# Supplementary material for: Deep learning shows declining groundwater levels in Germany until 2100 due to climate change
Source: Nat Commun. 2022 Mar 9;13:1221. doi: 10.1038/s41467-022-28770-2 (PMC8907324; doi:10.1038/s41467-022-28770-2)
Supplement: Supplementary file 1 — Supplementary Information [file 41467_2022_28770_MOESM1_ESM.pdf]

Deep learning shows declining groundwater levels in Germany until 2100 due to climate change (Supplementary Material)

Wunsch, A.*, Liesch, T., Broda, S.

*Corresponding Author

Contact: andreas.wunsch@kit.edu

Associated GitHub Respository:

GitHub AndreasWunsch/Long-Term-GWL-Simulations

DOI 10.5281/zenodo.4683901

Published Dataset:

DOI 10.5281/zenodo.4683879

Additional Supporting Information:

DOI 10.5281/zenodo.5645467

Content Overview:

- Table S1 lists all hydrographs including additional information such as identifiers and coordinates. We acknowledge support form the local authorities and point to the copyright statements published with the dataset (badge above).
- Table S2 lists the accuracy of the models in the past as well as the optimized hyperparameters
- Figures S1 to S2 show the simulation results for RCP2.6 and RCP4.5
- Tables S3 to S5 describe the boxplots of Figures 1 and 2 of the main text in numbers including confidence bounds
- Figures S3 to S8 show the input data analysis for RCP2.6 (S3, S4), RCP4.5 (S5, S6) and RCP8.5 (S7, S8)
- Figures S9-126 show the evaluation graph for the test period (2012-2016), the performance under extreme climatic conditions and the SHAP summary plot for each site

Additional Supporting Information: Under the link given above, additional supporting information is available. This includes time series plots for every site, every scenario and every according climate model, as well as heatmaps for the same, similar to Figure 3 of the main manuscript.

List of Tables

S1 List of all wells 6
S2 Model Errors and Hyperparameters 8
S3 RCP8.5 Boxplot Tables 12
S4 RCP4.5 Boxplot Tables 13
S5 RCP2.6 Boxplot Tables 14

List of Figures

S1 RCP2.6 Results (Maps) 10
S2 RCP4.5 Results (Maps) 11
S3 RCP2.6 Input Data Trend Analysis: Temperature 15
S4 RCP2.6 Input Data Trend Analysis: Precipitation 16
S5 RCP4.5 Input Data Trend Analysis: Temperature 17
S6 RCP4.5 Input Data Trend Analysis: Precipitation 18
S7 RCP8.5 Input Data Trend Analysis: Temperature 19
S8 RCP8.5 Input Data Trend Analysis: Precipitation 20
S9 Evaluation of BB_27381010 Model 21
S10 Evaluation of BB_28390113 Model 22
S11 Evaluation of BB_29519030 Model 23
S12 Evaluation of BB_30400591 Model 24
S13 Evaluation of BB_31400780 Model 25
S14 Evaluation of BB_31491979 Model 26
S15 Evaluation of BB_32455305 Model 27
S16 Evaluation of BB_33437070 Model 28
S17 Evaluation of BB_33437090 Model 29
S18 Evaluation of BB_33437106 Model 30
S19 Evaluation of BB_33452451 Model 31
S20 Evaluation of BB_33470960 Model 32
S21 Evaluation of BB_34426110 Model 33
S22 Evaluation of BB_34522461 Model 34
S23 Evaluation of BB_37451908 Model 35
S24 Evaluation of BB_39441476 Model 36
S25 Evaluation of BB_39496056 Model 37
S26 Evaluation of BB_40500136 Model 38
S27 Evaluation of BB_42458092 Model 39
S28 Evaluation of BW_100-813-7 Model 40
S29 Evaluation of BW_103-763-0 Model 41
S30 Evaluation of BW_107-666-2 Model 42
S31 Evaluation of BW_110-619-8 Model 43

S32 Evaluation of BW_112-211-1 Model 44
S33 Evaluation of BW_124-068-9 Model 45
S34 Evaluation of BW_131-115-0 Model 46
S35 Evaluation of BW_145-772-0 Model 47
S36 Evaluation of BW_15-568-2 Model 48
S37 Evaluation of BW_16-706-8 Model 49
S38 Evaluation of BW_177-770-1 Model 50
S39 Evaluation of BW_194-069-9 Model 51
S40 Evaluation of BY_11119 Model 52
S41 Evaluation of BY_13126 Model 53
S42 Evaluation of BY_15120 Model 54
S43 Evaluation of BY_22008 Model 55
S44 Evaluation of BY_24153 Model 56
S45 Evaluation of BY_25155 Model 57
S46 Evaluation of BY_3108 Model 58
S47 Evaluation of BY_5158 Model 59
S48 Evaluation of BY_5162 Model 60
S49 Evaluation of BY_7126 Model 61
S50 Evaluation of BY_8252 Model 62
S51 Evaluation of BY_83614 Model 63
S52 Evaluation of BY_9275 Model 64
S53 Evaluation of HE_10319 Model 65
S54 Evaluation of HE_11781 Model 66
S55 Evaluation of HE_12117 Model 67
S56 Evaluation of HE_14293 Model 68
S57 Evaluation of HE_14297 Model 69
S58 Evaluation of HE_6253 Model 70
S59 Evaluation of HE_6645 Model 71
S60 Evaluation of HE_7824 Model 72
S61 Evaluation of MV_16450200 Model 73
S62 Evaluation of MV_17390002 Model 74
S63 Evaluation of NI_100000728 Model 75
S64 Evaluation of NI_100000842 Model 76
S65 Evaluation of NI_100000926 Model 77
S66 Evaluation of NI_129300060 Model 78
S67 Evaluation of NI_200000620 Model 79
S68 Evaluation of NI_200000788 Model 80
S69 Evaluation of NI_200001068 Model 81
S70 Evaluation of NI_200001722 Model 82
S71 Evaluation of NI_200002153 Model 83
S72 Evaluation of NI_40000175 Model 84
S73 Evaluation of NI_40000233 Model 85
S74 Evaluation of NI_400080190 Model 86
S75 Evaluation of NI_400081660 Model 87

S76 Evaluation of NI_40501911 Model 88
S77 Evaluation of NI_405160331 Model 89
S78 Evaluation of NI_500000592 Model 90
S79 Evaluation of NI_600041871 Model 91
S80 Evaluation of NI_9700020 Model 92
S81 Evaluation of NI_9700080 Model 93
S82 Evaluation of NI_9700159 Model 94
S83 Evaluation of NI_9840391 Model 95
S84 Evaluation of NI_9840901 Model 96
S85 Evaluation of NI_9853172 Model 97
S86 Evaluation of NW_100140142 Model 98
S87 Evaluation of NW_100140762 Model 99
S88 Evaluation of NW_110040028 Model 100
S89 Evaluation of NW_110040041 Model 101
S90 Evaluation of NW_110060143 Model 102
S91 Evaluation of NW_110240017 Model 103
S92 Evaluation of NW_129660176 Model 104
S93 Evaluation of NW_129660206 Model 105
S94 Evaluation of NW_60090169 Model 106
S95 Evaluation of NW_60240258 Model 107
S96 Evaluation of NW_80000186 Model 108
S97 Evaluation of NW_80300376 Model 109
S98 Evaluation of NW_91163705 Model 110
S99 Evaluation of NW_91174909 Model 111
S100 Evaluation of RP_2373131200 Model 112
S101 Evaluation of RP_2378140100 Model 113
S102 Evaluation of RP_2587150500 Model 114
S103 Evaluation of SH_10L53126001 Model 115
S104 Evaluation of SH_10L54010004 Model 116
S105 Evaluation of SH_10L55005005 Model 117
S106 Evaluation of SH_10L55038005 Model 118
S107 Evaluation of SH_10L56048003 Model 119
S108 Evaluation of SH_10L57066002 Model 120
S109 Evaluation of SH_10L62060004 Model 121
S110 Evaluation of SN_46440927 Model 122
S111 Evaluation of SN_46460564 Model 123
S112 Evaluation of SN_47500596 Model 124
S113 Evaluation of SN_48390509 Model 125
S114 Evaluation of SN_49430964 Model 126
S115 Evaluation of SN_49484004 Model 127
S116 Evaluation of SN_49531740 Model 128
S117 Evaluation of SN_52410759 Model 129
S118 Evaluation of ST_31340028 Model 130
S119 Evaluation of ST_33340002 Model 131

S120 Evaluation of ST_34376608 Model 132
S121 Evaluation of ST_36340007 Model 133
S122 Evaluation of ST_39320023 Model 134
S123 Evaluation of ST_40415442 Model 135
S124 Evaluation of ST_41300015 Model 136
S125 Evaluation of ST_43435116 Model 137
S126 Evaluation of ST_44339213 Model 138

Table S1: List of all wells included in the published and used dataset. ID refers to ID in the respective data web service.

ID	Name	X_Coord (UTM 32N)	Y_Coord (UTM 32N)	Aquifer Type[1]	Ground Surf. [m asl]	Depth to GW [m]
BB_27381010	Lockstaedt OP	701838	5900152	p	49.7	1.32
BB_28390113	Beveringen OP	716212	5893813	p	69.7	0.45
BB_29519030	Schw.,Krzg.Teichm.-Seelenb.str	853218	5892330	p	6	2.63
BB_30400591	Stolpe, Birkenallee	731260	5874653	p	43.7	3.39
BB_31400780	Wusterhausen, Bahnlinie	732280	5863903	p	33.2	1.11
BB_31491979	Amalienhof-Falkenberg, KSP Nr. 6 GeDO	833480	5863149	p	1.7	0.48
BB_32455305	Hohenbruch, Weg n.Teerofen	781331	5857671	p	37.2	1.87
BB_33437070	Bredow, Siedlung Glien	768609	5836914	p	31.6	1.97
BB_33437090	Perwenitz, Luchstraße, OP	769303	5838525	p	32.35	1.67
BB_33437106	Nauen, gegenue.G.-Arco-Str. 148	764414	5838901	p	31.2	1.97
BB_33452451	Hennigsdorf,1,3km v.Trappen-A.	782655	5838050	p	32.15	0.93
BB_33470960	Lindow_Bernau	809679	5844936	p	66	1.39
BB_34426110	Bagow, Bollmannsruh	749731	5823802	p	31	2.31
BB_34522461	Sachsendorf, KSP Nr. 71	872109	5832083	p	12	2.64
BB_37451908	Juetchendorf,Str.Groeben-Gr.Beu.	785736	5800205	p	35.98	1.58
BB_39441476	Felgentreu, ca. 2 km oestl.	777374	5779401	p	51.35	1.74
BB_39496056	Kuschkow	839963	5779849	p	46.23	1.83
BB_40500136	Byhlen	853890	5762850	p	54.5	2.36
BB_42458092	Mahdel	788491	5735916	p	79.97	1.65
BW_100-813-7	GIENGEN TAUBENTAL	590920	5388935	k	497.28	35.32
BW_103-763-0	Sontheimer Wirtshäusle,STEINHEIM	578522	5391582	k	520.7	15.69
BW_107-666-2	GWM KB 3 A Weiler, Blaubeuren	557027	5360033	p	527.43	6.27
BW_110-619-8	GWM 7N SATTENBEUREN	546728	5320090	p	586.98	4.42
BW_112-211-1	3342 RASTATT STW-KA	443158	5414341	p	112.51	1.32
BW_124-068-9	3492 A KENZINGEN 2	405812	5338896	p	173.46	3.42
BW_131-115-0	GWM 3709, Ohlsbach	424415	5364473	p	162.06	1.82
BW_145-772-0	GWM 13-79 ALLMISHOFEN	578383	5294371	p	670.87	5.43
BW_15-568-2	GWM 129 ALTHEIM	532967	5331483	p	535.79	4.47
BW_16-706-8	GWM B2 Stäffelen, Niederstetten-Neuweiler	566991	5471838	f	325.56	12.78
BW_177-770-1	SBR 13, Aitrach	581180	5309055	p	612.57	18.51
BW_194-069-9	GWM B 4 Merdingen	401903	5319041	p	194.26	3.76
BY_11119	BADANHAUSEN 8B	679209	5432380	p	366.43	1.62
BY_13126	EBENHOFEN 758	620742	5296428	p	714.92	11.49
BY_15120	IHRLERSTEIN TIEF K1	707357	5426437	k	480.03	94.03
BY_22008	PFAFFENHAUSEN 82A	563680	5550758	p	179.7	2.91
BY_24153	SPEINSHART Q3	705931	5516765	k	416.22	1.42
BY_25155	IGLING 957	635606	5326788	p	592.7	12.73
BY_3108	MEINHEIM 429	634866	5432872	p	413.05	2.33
BY_5158	NEUSES	657993	5557607	p	273.22	0.59
BY_5162	Leitenbach 2	634999	5533371	p	240.54	4.73
BY_7126	ARBING 336A	799009	5401742	p	308.75	3.78
BY_8252	THIERHAUPTEN-S. D 36	640590	5371410	p	445.61	2.91
BY_83614	NBS-H_W KB 11_1	544375	5561463	f	233.42	37.16
BY_9275	GERLENHOFEN B3	576324	5355565	p	479.96	1.69
HE_10319	LETTGENBRUNN	532272	5555656	f	380.83	11.58
HE_11781	NAUHEIM	463126	5534254	p	89.03	1.12
HE_12117	DIEBURG	487910	5528018	p	142.12	2.76
HE_14293	KAILBACH	508261	5485664	p	354.5	8.56
HE_14297	SCHOELLENBACH	505323	5490972	p	294.52	5.26
HE_6253	NETRA	576396	5661004	f	312.7	8.86
HE_6645	KOMBACH	468878	5636195	p	259.71	8.23
HE_7824	LEIHGESTERN	476165	5597300	f	188.66	2.49
MV_16450200	Güttin	778860	6034537	p	11.34	4.22
MV_17390002	Klein Müritz	715409	6016858	p	3.8	1.97
NI_100000728	Ehra-Lessien I	622547	5824077	p	63.42	2.07
NI_100000842	Ehmen II	614668	5805451	f	76.5	1.62
NI_100000926	Sehlde	586584	5767546	f	117	1.82
NI_129300060	Rühen_RA 14 09	630065	5818918	p	57.68	0.94

[1] p: porous, f: fractured, k:karstic

ID	Name	X_Coord (UTM 32N)	Y_Coord (UTM 32N)	Aquifer Type[1]	Ground Surf. [m asl]	Depth to GW [m]
NI_200000620	Walsen	463773	5840294	p	37.22	2.18
NI_200000788	Wietzen	504336	5839574	p	64.54	1.97
NI_200001068	Rodewald MB I	534027	5835353	p	26.3	1.67
NI_200001722	Martfeld	503025	5858185	p	13.41	1.97
NI_200002153	Donstorf	470640	5835536	p	36.43	1.62
NI_40000175	Suttorf 261_7R	534210	5819061	p	40.92	3.14
NI_40000233	Fuhrberg-Ahrensnestgehege I	559587	5822985	p	41.75	2.25
NI_400080190	Wiepenkathen UE 19 FI	528647	5937096	p	4.3	0.80
NI_400081660	Huvenhoopsmoor UE 166	508134	5915473	p	8.1	2.66
NI_40501911	Wieste I	409615	5851908	p	31.63	4.20
NI_405160331	Klein Wohnste UE 33 FI	536583	5913023	p	36.7	2.17
NI_500000592	UWO 113_1 Uphusen	496767	5875283	p	6.83	2.10
NI_600041871	Wrestedt F1	606074	5862647	p	47.39	1.59
NI_9700020	Beverbruch 3_6	440656	5870819	p	15.59	0.31
NI_9700080	Feldkamp	449994	5801631	p	46.72	1.88
NI_9700159	Langwege	440052	5830351	p	29.01	1.59
NI_9840391	Tjücher Wilde I	388288	5932921	p	3.01	2.34
NI_9840901	Neermoor I	397042	5907998	p	0.83	1.41
NI_9853172	Südgeorgsfehner Moor	419063	5902646	p	6.39	2.42
NW_100140142	WG 22 LEVKENSTAD	484414	5800070	p	53.34	2.25
NW_100140762	WG 70 TAPPENAU	492808	5802796	f	56.03	3.85
NW_110040028	IV-2 -LIENEN-	430293	5774838	p	73.43	1.24
NW_110040041	IV-4 -SCHWEGE-	425943	5768989	p	55.55	1.07
NW_110060143	VI-14 - HANDORF -	412382	5760509	p	52.12	1.64
NW_110240017	AH-1 WELBERGEN	381429	5784337	p	48.58	2.17
NW_129660176	Silbecke	428864	5666613	k	320.32	53.38
NW_129660206	Schönholthausen I	432093	5671301	k	324.93	47.89
NW_60090169	HS 67	383993	5725893	p	58.17	5.38
NW_60240258	AH-25 VREDEN GRMAST	350479	5764657	p	40.61	1.65
NW_80000186	OEDT Nr020	317961	5688134	p	37.64	5.64
NW_80300376	MÜLHAUSEN Nr00-91	316792	5692404	p	32.21	2.03
NW_91163705	Pöppelsche Eikeloh	458197	5718515	k	109.91	11.26
NW_91174909	Brilon LederkeOL748	467638	5692896	k	446.53	15.72
RP_2373131200	1341 I Woerth am Rhein	432791	5431646	p	137.460007	2.00
RP_2378140100	1057 Boebingen	445538	5460551	p	113.989998	1.08
RP_2587150500	6024 Westerburg, Wengenroth	428344	5600050	f	313.140015	4.62
SH_10L53126001	4384_TRAMM_NORD_F1	606775	5937188	p	50.78	4.07
SH_10L54010004	1386_WESTERBARGUM_II	496464	6061837	p	4.78	4.39
SH_10L55005005	4522_OSTERMARKELSDORF	640373	6037388	p	9.8	1.27
SH_10L55038005	4565_LANGENHAGEN_HELMSTEICH	615529	6009571	p	105.59	0.90
SH_10L56048003	3587_TORNESCH_LIETHER_DAMM_F1	546919	5951632	p	13.34	3.68
SH_10L57066002	6067_RASTORFER_BAHNHOF	584911	6013922	p	29.32	9.08
SH_10L62060004	4712_REINBEK_SILKERFELD_F1	584999	5931988	p	22.78	9.03
SN_46440927	Bucha	781034	5700917	p	142.43	1.66
SN_46460564	Walda	813164	5695042	p	109.22	1.90
SN_47500596	Bischheim	851569	5688389	p	222.98	5.14
SN_48390509	Gatzen	727700	5668631	p	142.74	2.36
SN_49430964	Arras	773575	5663318	p	264.37	2.46
SN_49484004	Dresden, Königstraße	832311	5667461	p	112.25	7.13
SN_49531740	Schönbach	890527	5670891	p	386.83	7.12
SN_52410759	Muelsen-St-Niclas	752926	5625203	f	328.46	5.34
ST_31340028	Arendsee Süd	668011	5860747	p	32.5	2.13
ST_33340002	Altmersleben-Butterhorst	665806	5839436	p	29.66	1.20
ST_34376608	Charlottenhof	698014	5830461	p	32.42	1.43
ST_36340007	Satuelle	661910	5800899	p	55.5	1.14
ST_39320023	Hornhausen - Güte OP	647351	5767443	p	79.76	0.80
ST_40415442	Möllensdorf	743052	5757819	p	103.14	2.63
ST_41300015	Ilsenburg	616332	5749114	f	221.55	6.00
ST_43435116	Axien	767008	5734326	p	74.63	2.54
ST_44339213	Lengefeld	657980	5708535	f	258.48	6.15

[1] p: porous, f: fractured, k:karstic

Table S2: Model Errors in the past (2012-2016) and optimized Hyperparameters

ID	NSE	R^2	RMSE	rRMSE	Bias	rBias	filters	dense size	seqlength	batchsize
BB_27381010	0.91	0.91	0.09	5.53	0	0.09	192	19	29	41
BB_28390113	0.81	0.88	0.12	9.86	0.08	6.34	114	224	28	36
BB_29519030	0.7	0.81	0.1	7.26	0.07	4.75	190	47	36	16
BB_30400591	0.91	0.91	0.09	4.17	0.02	0.71	149	29	51	40
BB_31400780	0.7	0.75	0.1	8.18	-0.04	-3.32	254	72	51	41
BB_31491979	0.88	0.89	0.08	7.75	0.03	3.02	145	3	52	16
BB_32455305	0.91	0.93	0.1	5.37	-0.01	-0.54	176	81	39	182
BB_33437070	0.85	0.92	0.08	5.78	-0.05	-3.91	222	34	50	157
BB_33437090	0.8	0.85	0.15	7.84	-0.06	-3.46	201	54	37	46
BB_33437106	0.79	0.89	0.15	8.76	-0.1	-5.71	243	24	51	154
BB_33452451	0.86	0.86	0.13	8.88	0.01	0.75	110	55	51	16
BB_33470960	0.75	0.78	0.13	7.69	-0.04	-2.02	192	256	34	16
BB_34426110	0.85	0.9	0.09	5.16	-0.05	-2.97	248	29	50	201
BB_34522461	0.86	0.84	0.11	6.76	-0.01	-0.81	185	20	51	50
BB_37451908	0.75	0.8	0.12	9.5	-0.03	-2.08	199	21	52	205
BB_39441476	0.72	0.75	0.14	8.7	-0.07	-4.46	226	100	51	55
BB_39496056	0.86	0.89	0.1	6.04	0.02	1.06	220	63	43	22
BB_40500136	0.82	0.82	0.08	5.4	0.01	0.38	29	81	51	16
BB_42458092	0.79	0.88	0.14	8.05	-0.05	-3.1	226	17	51	143
BW_100-813-7	0.82	0.85	1.32	6.55	-0.59	-2.95	104	153	29	18
BW_103-763-0	0.86	0.84	2.54	8.78	0.65	2.25	164	32	28	16
BW_107-666-2	0.78	0.82	0.92	8.48	-0.41	-3.79	251	45	33	21
BW_110-619-8	0.8	0.87	0.22	7.65	-0.12	-4.06	198	94	37	18
BW_112-211-1	0.84	0.8	0.12	7.66	0.01	0.9	101	13	50	39
BW_124-068-9	0.78	0.79	0.18	6.43	-0.04	-1.55	201	53	36	16
BW_131-115-0	0.77	0.77	0.27	8.92	-0.01	-0.23	138	64	29	26
BW_145-772-0	0.86	0.87	0.37	6.46	-0.1	-1.69	179	76	52	34
BW_15-568-2	0.77	0.83	0.1	7.39	-0.01	-0.78	199	56	26	24
BW_16-706-8	0.86	0.86	0.3	7.63	0.07	1.8	90	154	28	25
BW_177-770-1	0.88	0.87	0.14	5.78	0.05	2.22	180	23	37	16
BW_194-069-9	0.85	0.85	0.09	5.43	-0.02	-1.19	225	27	51	18
BY_11119	0.77	0.75	0.2	8.67	-0.04	-1.57	225	85	34	47
BY_13126	0.85	0.87	0.31	6.14	-0.05	-1.05	255	25	51	16
BY_15120	0.78	0.77	1.41	7.34	0.72	3.78	156	58	52	16
BY_22008	0.81	0.8	0.23	6.4	-0.06	-1.71	51	7	30	36
BY_24153	0.81	0.82	0.11	9.69	0.03	2.2	178	242	9	17
BY_25155	0.79	0.82	0.19	7.95	-0.04	-1.47	202	45	45	24
BY_3108	0.81	0.82	0.26	8.96	-0.09	-3.12	237	43	38	111
BY_5158	0.76	0.76	0.2	9.78	-0.02	-1.14	178	198	20	16
BY_5162	0.87	0.87	0.14	6.92	0.02	0.82	239	31	29	22
BY_7126	0.85	0.89	0.15	6.4	-0.04	-1.6	83	130	52	16
BY_8252	0.78	0.81	0.19	9.06	0.11	5.3	236	169	52	16
BY_83614	0.8	0.78	0.62	8.36	0.05	0.73	132	40	51	16
BY_9275	0.87	0.87	0.06	6.5	0.02	1.95	187	256	42	16
HE_10319	0.82	0.83	2.03	9.71	-0.62	-2.98	254	80	17	24
HE_11781	0.84	0.86	0.18	9.8	0.02	0.91	85	107	28	37
HE_12117	0.86	0.93	0.11	5.64	-0.08	-3.92	180	54	52	96
HE_14293	0.78	0.75	0.25	7.98	0.07	2.05	248	103	30	29
HE_14297	0.77	0.75	0.24	5.58	-0.06	-1.48	144	255	46	16
HE_6253	0.79	0.75	0.48	6.78	0.13	1.86	225	59	43	40
HE_6645	0.93	0.91	0.12	3.64	-0.02	-0.7	220	63	43	22
HE_7824	0.8	0.83	0.24	6.45	-0.12	-3.06	41	12	51	52
MV_16450200	0.84	0.81	0.19	9.88	-0.02	-0.82	104	16	46	19
MV_17390002	0.8	0.79	0.24	10.67	-0.02	-0.71	249	217	51	18
NI_100000728	0.82	0.84	0.21	9.3	0.06	2.52	125	10	51	56
NI_100000842	0.77	0.76	0.17	10.14	0.04	2.44	255	4	42	52
NI_100000926	0.7	0.74	0.17	7.3	0.05	1.98	84	158	19	16
NI_129300060	0.87	0.88	0.19	8.22	0.05	2.28	169	122	51	80

ID	NSE	R^2	RMSE	rRMSE	Bias	rBias	filters	dense size	seqlength	batchsize
NI_200000620	0.82	0.87	0.22	8.24	0.14	5.39	197	93	52	46
NI_200000788	0.9	0.88	0.16	6.99	0.04	1.7	231	16	52	78
NI_200001068	0.83	0.83	0.13	8.19	0.04	2.63	218	70	50	76
NI_200001722	0.9	0.91	0.06	4.78	0	0.06	254	87	47	20
NI_200002153	0.76	0.85	0.19	9.86	0.13	6.41	144	248	12	156
NI_40000175	0.7	0.77	0.19	9.75	0.11	5.49	79	89	26	20
NI_40000233	0.88	0.88	0.09	4.99	0.05	2.53	238	100	39	19
NI_400080190	0.72	0.77	0.15	8.62	0.1	5.53	241	53	52	36
NI_400081660	0.75	0.76	0.15	9.63	0.06	4.24	138	224	11	157
NI_40501911	0.91	0.83	0.12	4.89	0.01	0.45	216	48	51	20
NI_405160331	0.8	0.83	0.21	7.75	0.12	4.59	197	27	50	26
NI_500000592	0.88	0.9	0.06	6.51	0.04	4.11	44	10	22	36
NI_600041871	0.79	0.8	0.12	7.59	-0.02	-1.5	217	97	42	18
NI_9700020	0.87	0.84	0.11	6.87	0.02	1.35	231	76	40	60
NI_9700080	0.84	0.84	0.21	8.03	0.11	4.07	195	31	23	29
NI_9700159	0.86	0.86	0.12	6.14	-0.01	-0.75	44	10	22	36
NI_9840391	0.9	0.89	0.08	6.58	0	0.38	217	246	19	21
NI_9840901	0.83	0.83	0.07	6.06	-0.01	-0.86	242	87	28	16
NI_9853172	0.87	0.89	0.1	7.73	0.02	1.53	203	52	13	63
NW_100140142	0.79	0.79	0.19	9.31	0.13	6.3	256	13	52	127
NW_100140762	0.83	0.81	0.16	7.1	0.02	1.05	205	51	36	49
NW_110040028	0.83	0.89	0.12	7.97	0.06	4.44	254	65	28	37
NW_110040041	0.86	0.87	0.12	7.08	0.03	1.95	149	98	25	16
NW_110060143	0.86	0.86	0.22	7.8	-0.01	-0.31	173	255	39	17
NW_110240017	0.84	0.83	0.11	5.47	0.03	1.33	223	58	42	27
NW_129660176	0.87	0.88	4	7.41	-0.29	-0.53	84	158	19	16
NW_129660206	0.81	0.8	1.45	6.95	0.28	1.34	34	132	52	16
NW_60090169	0.89	0.9	0.21	5.1	-0.03	-0.75	202	61	51	195
NW_60240258	0.77	0.81	0.15	7.85	0.07	3.46	205	51	36	49
NW_80000186	0.84	0.84	0.09	4.84	-0.01	-0.74	76	256	52	16
NW_80300376	0.83	0.83	0.1	5.47	0	-0.23	241	204	16	16
NW_91163705	0.82	0.77	1.18	8.57	0.11	0.82	237	43	38	111
NW_91174909	0.82	0.81	0.44	5.29	0.12	1.46	189	195	12	57
RP_2373131200	0.76	0.84	0.23	13.08	-0.13	-7.56	256	68	52	16
RP_2378140100	0.81	0.79	0.25	9.14	-0.04	-1.53	74	168	49	41
RP_2587150500	0.78	0.78	0.3	8.22	0.03	0.78	96	154	18	17
SH_10L53126001	0.84	0.66	0.18	6.34	0	0.13	82	107	52	65
SH_10L54010004	0.77	0.82	0.16	11.45	-0.06	-4.28	115	221	22	68
SH_10L55005005	0.85	0.88	0.21	11.46	0.04	2.05	243	2	44	27
SH_10L55038005	0.76	0.76	0.14	10.14	0.06	4.29	210	60	29	16
SH_10L56048003	0.78	0.75	0.13	7.28	0	0.12	236	10	26	21
SH_10L57066002	0.81	0.79	0.11	6.87	0.04	2.78	96	7	45	16
SH_10L62060004	0.88	0.89	0.1	7.1	0	0.17	163	248	23	16
SN_46440927	0.85	0.85	0.25	6.74	0.03	0.78	227	24	52	16
SN_46460564	0.71	0.79	0.15	8.31	-0.08	-4.32	244	154	50	78
SN_47500596	0.74	0.77	0.19	7.91	-0.07	-2.71	187	30	52	48
SN_48390509	0.8	0.71	0.22	7.1	0.03	0.82	252	75	51	37
SN_49430964	0.75	0.74	0.49	9.07	-0.04	-0.68	238	16	40	93
SN_49484004	0.73	0.76	0.32	8.19	0.09	2.37	135	34	31	16
SN_49531740	0.71	0.71	0.28	8.16	0.01	0.15	220	31	52	96
SN_52410759	0.82	0.84	0.28	6.16	0.05	1.14	217	70	43	35
ST_31340028	0.85	0.86	0.12	6.63	0.08	4.22	243	115	51	22
ST_33340002	0.79	0.86	0.12	7.37	-0.06	-3.7	41	34	29	39
ST_34376608	0.84	0.85	0.09	7.32	0.02	1.84	106	174	16	17
ST_36340007	0.73	0.73	0.14	6.6	-0.03	-1.57	245	56	42	20
ST_39320023	0.78	0.81	0.11	6.29	-0.03	-1.7	245	27	26	51
ST_40415442	0.83	0.84	0.14	6.38	0.06	2.85	182	17	50	33
ST_41300015	0.81	0.87	0.47	8.35	-0.25	-4.43	180	32	23	20
ST_43435116	0.72	0.75	0.23	7.69	-0.06	-2.04	232	41	48	16
ST_44339213	0.8	0.83	0.18	6.11	0.02	0.62	47	45	30	21

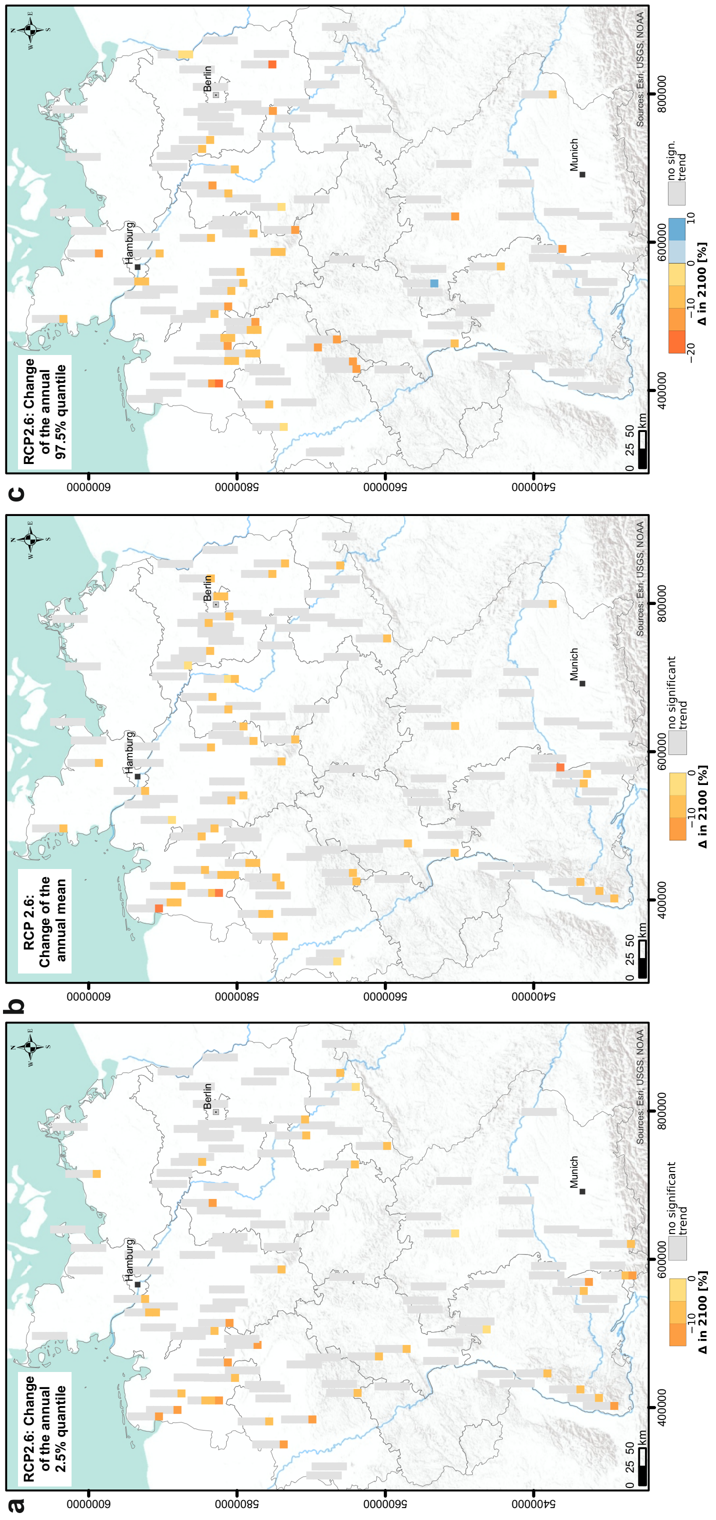

Figure S1: Change of groundwater level [%] in 2100 relative to 2014 (start of sim.) for each site and each climate projection under **RCP2.6**, based on a linear trend analysis: a) annual 2.5% quantile, b) the annual mean, c) annual 97.5% quantile.

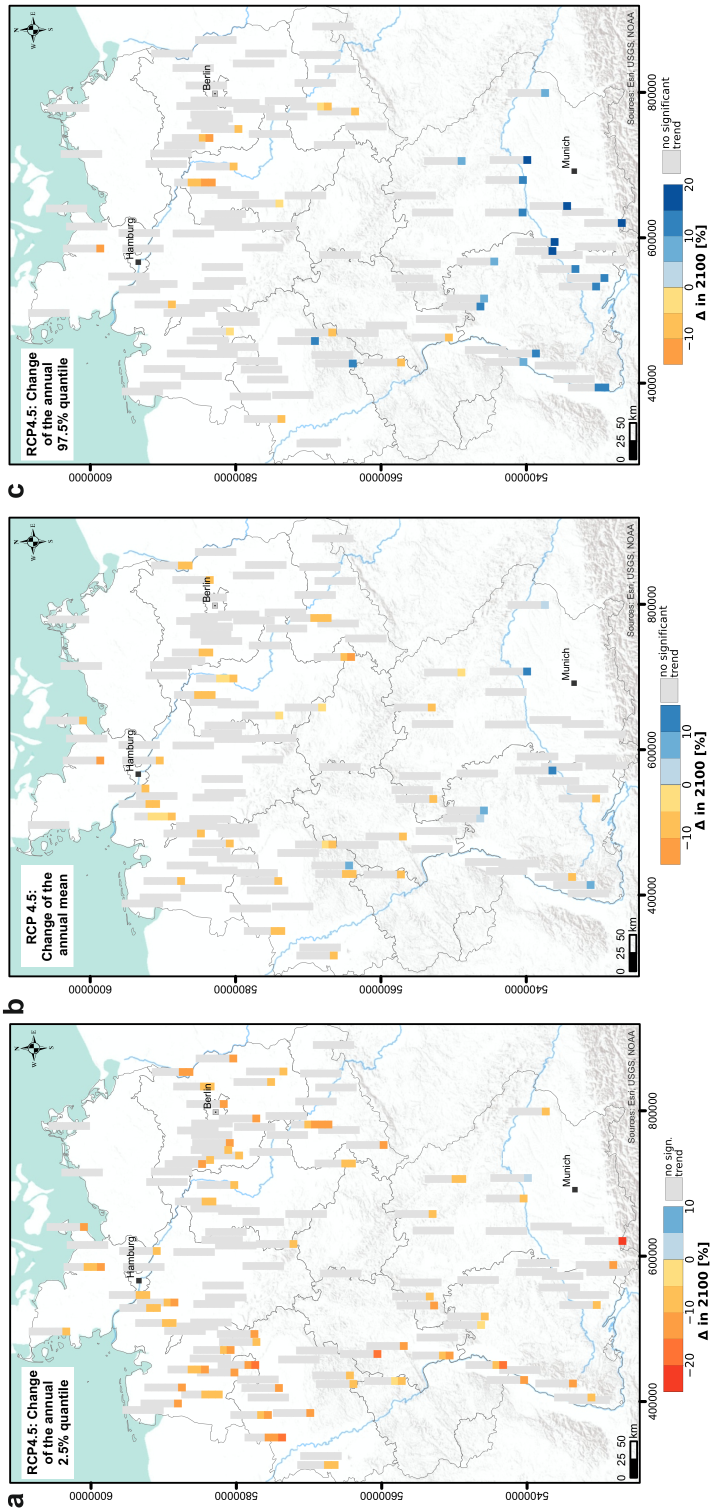

Figure S2: Change of groundwater level [%] in 2100 relative to 2014 (start of sim.) for each site and each climate projection under **RCP4.5**, based on a linear trend analysis: a) annual 2.5% quantile, b) the annual mean, c) annual 97.5% quantile.

Table S3: Table describing Boxplots in Figure 1 of the main text (**RCP8.5**). lb = lower bound / ub = upper bound of 95% confidence interval. Values relative [%] and absolute [m]:

relative

**Q97.5**	lb	**p1**	ub	lb	**p2**	ub	lb	**p3**	ub	lb	**p4**	ub	lb	**p5**	ub	lb	**p6**	ub
Max	8.9	**18.0**	26.6	4.8	**14.0**	27.9	1.7	**18.2**	35.8	4.0	**13.8**	25.3	2.1	**12.5**	23.3	7.5	**17.0**	27.9
Q75	-22.1	**-10.8**	-1.1	1.2	**10.5**	19.5	-14.5	**-7.7**	-0.5	-15.2	**-7.8**	-0.8	-11.0	**-6.3**	-0.7	3.8	**12.7**	21.7
Median	-27.4	**-15.4**	-4.3	0.7	**7.5**	12.7	-20.2	**-11.3**	-1.8	-21.7	**-11.9**	-2.2	-17.0	**-10.3**	-1.7	1.5	**9.3**	18.0
Q25	-35.0	**-21.1**	-8.7	-15.0	**-8.8**	-1.2	-25.0	**-14.8**	-2.9	-27.9	**-16.7**	-4.8	-23.7	**-13.2**	-4.5	-14.2	**-8.0**	-1.4
Min	-60.5	**-39.8**	-19.1	-24.1	**-13.8**	-3.4	-44.4	**-30.4**	-15.3	-46.8	**-30.2**	-13.3	-44.4	**-29.3**	-12.8	-24.0	**-16.7**	-9.8
Samples		**45**			**15**			**23**			**29**			**28**			**32**	

**mean**	lb	**p1**	ub	lb	**p2**	ub	lb	**p3**	ub	lb	**p4**	ub	lb	**p5**	ub	lb	**p6**	ub
Max	-9.8	**-5.0**	0.1	0.2	**7.2**	13.4	0.0	**10.0**	19.5	-10.5	**-5.7**	2.4	-8.0	**-4.2**	0.1	5.7	**14.9**	23.8
Q75	-23.8	**-13.9**	-4.4	-10.4	**-5.9**	-0.6	-12.8	**-7.7**	-1.8	-15.4	**-8.6**	-1.1	-11.4	**-6.8**	-1.0	1.0	**7.7**	13.7
Median	-29.8	**-17.6**	-6.2	-14.7	**-7.9**	-1.9	-16.6	**-10.4**	-3.2	-18.9	**-10.7**	-2.0	-13.5	**-7.7**	-2.4	-11.1	**-6.3**	-0.4
Q25	-36.5	**-22.3**	-8.8	-18.9	**-11.2**	-3.5	-20.6	**-11.8**	-5.2	-26.5	**-14.1**	-3.2	-19.0	**-11.3**	-3.8	-14.7	**-8.4**	-2.4
Min	-56.9	**-35.4**	-14.8	-24.6	**-15.4**	-6.0	-39.2	**-26.1**	-12.8	-39.6	**-23.2**	-11.9	-33.3	**-21.7**	-11.5	-20.6	**-11.7**	-4.9
Samples		**114**			**26**			**59**			**50**			**49**			**34**	

**Q2.5**	lb	**p1**	ub	lb	**p2**	ub	lb	**p3**	ub	lb	**p4**	ub	lb	**p5**	ub	lb	**p6**	ub
Max	□16.27	**-8.0**	-0.8	-8.2	**-4.4**	-0.1	-5.9	**-3.0**	-0.1	-12.3	**-7.9**	2.9	-8.4	**-4.2**	1.0	0.6	**6.5**	12.2
Q75	-43.5	**-28.3**	-13.1	-15.6	**-9.4**	-2.0	-15.2	**-8.8**	-1.9	-24.3	**-13.9**	-4.6	-14.6	**-7.9**	-1.1	-12.6	**-6.9**	-1.2
Median	-51.9	**-34.1**	-16.8	-19.4	**-11.0**	-3.5	-19.9	**-11.8**	-4.0	-30.5	**-19.0**	-8.2	-16.1	**-9.3**	-2.4	-14.7	**-8.7**	-1.8
Q25	-61.9	**-40.8**	-19.5	-24.2	**-14.5**	-5.7	-23.5	**-14.5**	-5.8	-36.4	**-24.3**	-11.5	-21.3	**-11.7**	-3.8	-18.9	**-10.9**	-2.7
Min	-118.8	**-78.7**	-45.3	-40.7	**-28.1**	-15.0	-40.6	**-26.7**	-14.6	-62.4	**-41.6**	-23.5	-37.6	**-25.7**	-13.4	-22.9	**-13.7**	-5.2
Samples		**118**			**70**			**61**			**94**			**60**			**34**	

absolute

**Q97.5**	lb	**p1**	ub	lb	**p2**	ub	lb	**p3**	ub	lb	**p4**	ub	lb	**p5**	ub	lb	**p6**	ub
Max	0.9	**2.2**	4.0	0.3	**2.5**	5.0	0.1	**0.5**	0.9	0.4	**1.2**	2.0	0.0	**0.2**	0.4	1.1	**4.0**	7.1
Q75	-0.3	**-0.2**	0.0	0.0	**0.2**	0.3	-0.2	**-0.1**	0.0	-0.2	**-0.1**	0.0	-0.2	**-0.1**	0.0	0.1	**0.3**	0.6
Median	-0.5	**-0.3**	-0.1	0.0	**0.1**	0.2	-0.3	**-0.2**	0.0	-0.3	**-0.2**	0.0	-0.3	**-0.2**	0.0	0.0	**0.2**	0.3
Q25	-0.8	**-0.5**	-0.1	-0.2	**-0.1**	0.0	-0.5	**-0.3**	-0.1	-0.7	**-0.3**	-0.1	-0.6	**-0.4**	-0.1	-0.1	**-0.1**	0.0
Min	-2.5	**-1.3**	-0.5	-0.8	**-0.4**	0.0	-1.0	**-0.6**	-0.2	-1.2	**-0.8**	-0.3	-1.4	**-0.8**	-0.4	-0.5	**-0.3**	-0.2
Samples		**45**			**15**			**23**			**29**			**28**			**32**	

**mean**	lb	**p1**	ub	lb	**p2**	ub	lb	**p3**	ub	lb	**p4**	ub	lb	**p5**	ub	lb	**p6**	ub
Max	-0.2	**-0.1**	0.0	0.0	**1.3**	2.4	0.0	**0.3**	0.5	-0.2	**-0.1**	0.1	-0.1	**-0.1**	0.0	1.6	**4.1**	6.6
Q75	-0.4	**-0.2**	-0.1	-0.1	**-0.1**	0.0	-0.2	**-0.1**	0.0	-0.3	**-0.1**	0.0	-0.2	**-0.1**	0.0	0.0	**0.2**	0.3
Median	-0.5	**-0.3**	-0.1	-0.3	**-0.2**	0.0	-0.3	**-0.2**	-0.1	-0.5	**-0.3**	-0.1	-0.3	**-0.2**	-0.1	-0.1	**-0.1**	0.0
Q25	-0.8	**-0.5**	-0.2	-0.4	**-0.2**	-0.1	-0.5	**-0.3**	-0.1	-0.7	**-0.4**	-0.1	-0.5	**-0.3**	-0.1	-0.2	**-0.2**	0.0
Min	-9.1	**-5.3**	-1.7	-2.3	**-1.1**	-0.3	-6.0	**-3.4**	-1.1	-7.2	**-3.7**	-0.7	-2.3	**-1.1**	-0.4	-0.6	**-0.4**	-0.2
Samples		**114**			**26**			**59**			**50**			**49**			**34**	

**Q2.5**	lb	**p1**	ub	lb	**p2**	ub	lb	**p3**	ub	lb	**p4**	ub	lb	**p5**	ub	lb	**p6**	ub
Max	-0.5	**-0.3**	0.0	-0.2	**-0.1**	0.0	-0.1	**-0.1**	0.0	-0.2	**-0.1**	0.0	-0.1	**-0.1**	0.0	0.1	**1.2**	2.1
Q75	-0.7	**-0.5**	-0.2	-0.3	**-0.2**	0.0	-0.3	**-0.2**	0.0	-0.4	**-0.2**	-0.1	-0.3	**-0.1**	0.0	-0.2	**-0.1**	0.0
Median	-1.0	**-0.6**	-0.3	-0.3	**-0.2**	-0.1	-0.3	**-0.2**	-0.1	-0.6	**-0.4**	-0.2	-0.3	**-0.2**	0.0	-0.3	**-0.1**	0.0
Q25	-1.4	**-0.9**	-0.4	-0.5	**-0.3**	-0.1	-0.5	**-0.4**	-0.1	-0.9	**-0.6**	-0.3	-0.5	**-0.3**	-0.1	-0.4	**-0.2**	-0.1
Min	-23.5	**-15.5**	-7.7	-6.8	**-4.0**	-1.1	-5.7	**-3.1**	-1.1	-13.7	**-9.1**	-4.5	-4.0	**-2.0**	-0.6	-5.7	**-3.5**	-1.2
Samples		**118**			**70**			**61**			**94**			**60**			**34**	

Table S4: Table describing **RCP4.5** Boxplots in Figure 2 of the main text. lb = lower bound / ub = upper bound of 95% confidence interval. Values relative [%] and absolute [m]:

relative

**Q97.5**	lb	**p1**	ub	lb	**p2**	ub	lb	**p3**	ub	lb	**p4**	ub	lb	**p5**	ub	lb	**p6**	ub
Max	-9.7	**-4.7**	0.0	-8.7	**-4.7**	-0.2	5.3	**14.4**	23.0	-9.3	**-5.0**	-0.1	-14.9	**-7.9**	-1.4	8.0	**19.7**	32.0
Q75	-9.8	**-4.9**	-0.1	-10.0	**-5.4**	-0.2	-2.6	**4.6**	11.4	-12.8	**-7.4**	-1.6	-14.9	**-7.9**	-1.4	2.7	**13.7**	26.6
Median	-16.7	**-8.9**	-0.3	-11.3	**-6.0**	-0.3	-10.4	**-5.3**	-0.2	-14.1	**-8.0**	-2.2	-14.9	**-7.9**	-1.4	2.0	**12.2**	22.1
Q25	-18.5	**-9.6**	-0.4	-12.4	**-6.4**	-0.5	-15.3	**-8.4**	-1.3	-18.9	**-11.0**	-2.8	-14.9	**-7.9**	-1.4	0.8	**9.7**	17.5
Min	-18.7	**-9.8**	-0.8	-13.6	**-6.8**	-0.6	-20.2	**-11.5**	-2.4	-24.3	**-13.9**	-3.7	-14.9	**-7.9**	-1.4	-14.9	**-7.3**	-1.2
Samples		**5**			**3**			**3**			**7**			**1**			**21**	

**mean**	lb	**p1**	ub	lb	**p2**	ub	lb	**p3**	ub	lb	**p4**	ub	lb	**p5**	ub	lb	**p6**	ub
Max	-5.7	**-3.0**	0.0	0.6	**6.1**	11.6	0.1	**6.0**	11.5	-8.2	**-4.4**	0.2	-10.7	**-5.7**	0.3	3.0	**11.2**	22.2
Q75	-8.9	**-4.4**	-0.1	-7.4	**-4.1**	-0.1	-5.1	**-2.5**	0.2	-10.0	**-5.5**	-0.6	-10.9	**-6.2**	-0.1	0.4	**5.0**	9.0
Median	-9.8	**-5.2**	-0.4	-10.2	**-5.2**	-0.7	-8.5	**-4.9**	-0.1	-11.6	**-6.4**	-1.0	-12.3	**-6.7**	-1.0	-3.0	**0.1**	3.3
Q25	-10.5	**-5.6**	-0.5	-10.6	**-5.4**	-0.9	-11.3	**-5.7**	-0.2	-13.1	**-7.2**	-1.5	-14.2	**-8.1**	-1.5	-7.1	**-3.8**	-0.3
Min	-11.1	**-6.1**	-0.6	-19.5	**-10.2**	-1.0	-17.0	**-8.0**	-1.0	-17.9	**-10.9**	-3.2	-20.1	**-9.6**	-2.4	-12.0	**-6.2**	-1.1
Samples		**6**			**6**			**5**			**20**			**6**			**10**	

**Q2.5**	lb	**p1**	ub	lb	**p2**	ub	lb	**p3**	ub	lb	**p4**	ub	lb	**p5**	ub	lb	**p6**	ub
Max	-	**-**	-	-10.2	**-5.0**	-0.1	-12.5	**-6.4**	0.0	-8.2	**-4.9**	0.1	-7.4	**-3.8**	0.0	-0.2	**5.6**	11.2
Q75	-	**-**	-	-14.4	**-7.8**	-0.1	-15.7	**-8.6**	-0.7	-15.5	**-8.6**	-1.2	-15.6	**-7.8**	-0.6	-0.2	**5.6**	11.2
Median	-	**-**	-	-16.8	**-8.5**	-0.8	-17.4	**-9.3**	-0.9	-18.7	**-10.3**	-2.1	-16.3	**-8.3**	-1.0	-0.2	**5.6**	11.2
Q25	-	**-**	-	-18.2	**-9.3**	-1.0	-18.4	**-10.3**	-1.1	-21.3	**-12.5**	-3.2	-17.9	**-10.0**	-1.5	-0.2	**5.6**	11.2
Min	-	**-**	-	-22.4	**-12.0**	-2.0	-23.9	**-13.2**	-2.3	-32.7	**-20.7**	-7.1	-20.5	**-11.5**	-4.4	-0.2	**5.6**	11.2
Samples		**0**			**10**			**13**			**50**			**15**			**1**	

absolute

**Q97.5**	lb	**p1**	ub	lb	**p2**	ub	lb	**p3**	ub	lb	**p4**	ub	lb	**p5**	ub	lb	**p6**	ub
Max	-0.2	**-0.1**	0.0	-0.2	**-0.1**	0.0	0.1	**0.2**	0.3	-0.2	**-0.1**	0.0	-0.2	**-0.1**	0.0	1.8	**5.7**	11.2
Q75	-0.2	**-0.1**	0.0	-0.2	**-0.1**	0.0	0.0	**0.1**	0.2	-0.2	**-0.1**	0.0	-0.2	**-0.1**	0.0	0.1	**1.0**	2.0
Median	-0.3	**-0.2**	0.0	-0.2	**-0.1**	0.0	-0.1	**-0.1**	0.0	-0.2	**-0.1**	0.0	-0.2	**-0.1**	0.0	0.1	**0.3**	0.6
Q25	-0.6	**-0.3**	0.0	-0.4	**-0.2**	0.0	-0.2	**-0.1**	0.0	-0.3	**-0.2**	-0.1	-0.2	**-0.1**	0.0	0.0	**0.2**	0.3
Min	-1.0	**-0.5**	0.0	-0.6	**-0.3**	0.0	-0.3	**-0.2**	0.0	-0.5	**-0.3**	-0.1	-0.2	**-0.1**	0.0	-0.2	**-0.1**	0.0
Samples		**5**			**3**			**3**			**7**			**1**			**21**	

**mean**	lb	**p1**	ub	lb	**p2**	ub	lb	**p3**	ub	lb	**p4**	ub	lb	**p5**	ub	lb	**p6**	ub
Max	-0.1	**0.0**	0.0	0.1	**1.1**	2.1	0.0	**0.1**	0.2	-0.1	**-0.1**	0.0	-0.1	**-0.1**	0.0	0.5	**3.1**	6.1
Q75	-0.2	**-0.1**	0.0	-0.1	**-0.1**	0.0	-0.1	**0.0**	0.0	-0.1	**-0.1**	0.0	-0.1	**-0.1**	0.0	0.0	**0.2**	0.3
Median	-0.2	**-0.1**	0.0	-0.1	**-0.1**	0.0	-0.1	**-0.1**	0.0	-0.2	**-0.1**	0.0	-0.2	**-0.1**	0.0	-0.1	**0.0**	0.1
Q25	-0.4	**-0.2**	0.0	-0.2	**-0.1**	0.0	-0.1	**-0.1**	0.0	-0.4	**-0.2**	0.0	-0.4	**-0.2**	0.0	-0.2	**-0.1**	0.0
Min	-5.5	**-3.0**	-0.3	-0.5	**-0.3**	0.0	-0.3	**-0.1**	0.0	-6.5	**-4.1**	-1.4	-0.5	**-0.2**	0.0	-0.3	**-0.2**	0.0
Samples		**6**			**6**			**5**			**20**			**6**			**10**	

**Q2.5**	lb	**p1**	ub	lb	**p2**	ub	lb	**p3**	ub	lb	**p4**	ub	lb	**p5**	ub	lb	**p6**	ub
Max	-	**-**	-	-0.2	**-0.1**	0.0	-0.1	**-0.1**	0.0	-0.1	**-0.1**	0.0	-0.1	**-0.1**	0.0	0.0	**1.0**	2.0
Q75	-	**-**	-	-0.2	**-0.1**	0.0	-0.2	**-0.1**	0.0	-0.3	**-0.2**	0.0	-0.2	**-0.1**	0.0	0.0	**1.0**	2.0
Median	-	**-**	-	-0.2	**-0.1**	0.0	-0.3	**-0.2**	0.0	-0.4	**-0.2**	0.0	-0.3	**-0.2**	0.0	0.0	**1.0**	2.0
Q25	-	**-**	-	-0.3	**-0.2**	0.0	-0.4	**-0.2**	0.0	-0.5	**-0.3**	-0.1	-0.4	**-0.2**	0.0	0.0	**1.0**	2.0
Min	-	**-**	-	-0.6	**-0.3**	0.0	-0.8	**-0.5**	-0.1	-8.6	**-5.1**	-1.2	-0.7	**-0.4**	-0.1	0.0	**1.0**	2.0
Samples		**0**			**10**			**13**			**50**			**15**			**1**	

Table S5: Table describing **RCP2.6** Boxplots in Figure 2 of the main text. lb = lower bound / ub = upper bound of 95% confidence interval. Values relative [%] and absolute [m]:

relative

**Q97.5**	lb	**p1**	ub	lb	**p2**	ub	lb	**p3**	ub	lb	**p4**	ub	lb	**p5**	ub
Max	-7.8	**-4.1**	0.1	0.3	**6.1**	13.1	-9.1	**-4.8**	1.7	-12.1	**-6.5**	-0.1	-10.7	**-5.8**	0.2
Q75	-10.9	**-6.0**	-0.5	0.3	**6.1**	13.1	-12.4	**-5.6**	0.2	-15.9	**-8.7**	-1.1	-14.2	**-7.8**	-1.0
Median	-15.2	**-8.1**	-1.1	0.3	**6.1**	13.1	-14.5	**-6.6**	-0.3	-21.3	**-11.7**	-1.9	-18.0	**-9.1**	-1.2
Q25	-18.6	**-10.0**	-1.9	0.3	**6.1**	13.1	-17.1	**-8.8**	-0.9	-24.2	**-12.8**	-2.5	-21.4	**-11.8**	-2.0
Min	-24.7	**-15.4**	-6.9	0.3	**6.1**	13.1	-21.6	**-12.8**	-2.5	-25.9	**-14.6**	-3.6	-28.3	**-17.2**	-5.7
Samples		**24**			**1**			**4**			**8**			**12**	

**mean**	lb	**p1**	ub	lb	**p2**	ub	lb	**p3**	ub	lb	**p4**	ub	lb	**p5**	ub
Max	-8.6	**-5.0**	0.5	-8.6	**-4.4**	0.0	-14.7	**-7.6**	-0.5	-	**-**	-	-6.7	**-3.8**	0.1
Q75	-12.0	**-6.2**	-0.4	-9.9	**-5.0**	-0.1	-15.2	**-7.9**	-0.9	-	**-**	-	-11.3	**-6.2**	-0.7
Median	-12.8	**-6.7**	-0.8	-11.8	**-5.8**	-0.2	-15.7	**-8.3**	-1.3	-	**-**	-	-12.1	**-7.1**	-1.4
Q25	-13.8	**-7.4**	-1.1	-12.5	**-6.6**	-0.6	-18.2	**-10.1**	-2.3	-	**-**	-	-14.1	**-8.2**	-2.3
Min	-17.8	**-8.8**	-2.5	-14.4	**-7.7**	-0.7	-20.6	**-11.9**	-3.2	-	**-**	-	-19.9	**-11.4**	-3.5
Samples		**26**			**7**			**3**			**0**			**24**	

**Q2.5**	lb	**p1**	ub	lb	**p2**	ub	lb	**p3**	ub	lb	**p4**	ub	lb	**p5**	ub
Max	-8.4	**-4.5**	-0.1	-15.4	**-7.5**	-0.3	-17.7	**-9.3**	-0.4	-13.9	**-7.4**	-0.1	-6.2	**-3.2**	-0.1
Q75	-11.6	**-6.0**	-0.3	-16.2	**-8.0**	-0.6	-17.8	**-9.6**	-0.8	-16.2	**-8.3**	-0.7	-12.2	**-6.0**	-0.4
Median	-16.8	**-8.3**	-0.6	-16.9	**-8.5**	-1.0	-17.9	**-10.0**	-1.2	-17.3	**-8.7**	-0.9	-16.0	**-9.2**	-0.7
Q25	-19.0	**-11.1**	-1.5	-17.6	**-9.0**	-1.4	-17.9	**-10.4**	-1.7	-18.4	**-9.1**	-1.0	-19.4	**-10.4**	-1.5
Min	-25.2	**-13.6**	-3.2	-18.3	**-9.6**	-1.7	-18.0	**-10.7**	-2.1	-20.3	**-10.0**	-1.1	-23.1	**-12.9**	-3.7
Samples		**12**			**2**			**2**			**4**			**21**	

absolute

**Q97.5**	lb	**p1**	ub	lb	**p2**	ub	lb	**p3**	ub	lb	**p4**	ub	lb	**p5**	ub
Max	-0.1	**0.0**	0.0	0.0	**0.5**	1.0	-0.1	**0.0**	0.0	-0.1	**-0.1**	0.0	-0.2	**-0.1**	0.0
Q75	-0.2	**-0.1**	0.0	0.0	**0.5**	1.0	-0.2	**-0.1**	0.0	-0.3	**-0.2**	0.0	-0.2	**-0.1**	0.0
Median	-0.2	**-0.1**	0.0	0.0	**0.5**	1.0	-0.4	**-0.2**	0.0	-1.1	**-0.7**	-0.1	-0.3	**-0.2**	0.0
Q25	-0.3	**-0.2**	0.0	0.0	**0.5**	1.0	-1.4	**-0.8**	-0.1	-2.7	**-1.5**	-0.2	-0.4	**-0.2**	0.0
Min	-0.5	**-0.3**	-0.1	0.0	**0.5**	1.0	-3.8	**-2.2**	-0.4	-14.0	**-7.7**	-1.4	-0.6	**-0.3**	-0.1
Samples		**24**			**1**			**4**			**8**			**12**	

**mean**	lb	**p1**	ub	lb	**p2**	ub	lb	**p3**	ub	lb	**p4**	ub	lb	**p5**	ub
Max	-0.1	**-0.1**	0.0	-0.1	**-0.1**	0.0	-0.1	**-0.1**	0.0	-	**-**	-	-0.1	**-0.1**	0.0
Q75	-0.2	**-0.1**	0.0	-0.1	**-0.1**	0.0	-0.9	**-0.5**	-0.1	-	**-**	-	-0.2	**-0.1**	0.0
Median	-0.2	**-0.1**	0.0	-0.2	**-0.1**	0.0	-1.7	**-0.9**	-0.1	-	**-**	-	-0.2	**-0.1**	0.0
Q25	-0.2	**-0.1**	0.0	-0.2	**-0.1**	0.0	-3.7	**-2.1**	-0.5	-	**-**	-	-0.3	**-0.2**	0.0
Min	-0.6	**-0.3**	0.0	-0.2	**-0.1**	0.0	-5.7	**-3.3**	-0.9	-	**-**	-	-6.6	**-3.7**	-1.2
Samples		**26**			**7**			**3**			**0**			**24**	

**Q2.5**	lb	**p1**	ub	lb	**p2**	ub	lb	**p3**	ub	lb	**p4**	ub	lb	**p5**	ub
Max	-0.2	**-0.1**	0.0	-0.3	**-0.2**	0.0	-0.2	**-0.1**	0.0	-0.3	**-0.1**	0.0	-0.2	**-0.1**	0.0
Q75	-0.2	**-0.1**	0.0	-0.3	**-0.2**	0.0	-0.6	**-0.3**	0.0	-0.3	**-0.2**	0.0	-0.2	**-0.1**	0.0
Median	-0.3	**-0.2**	0.0	-0.3	**-0.2**	0.0	-1.0	**-0.5**	0.0	-0.6	**-0.3**	0.0	-0.3	**-0.2**	0.0
Q25	-0.4	**-0.2**	0.0	-0.3	**-0.2**	0.0	-1.5	**-0.8**	0.0	-0.8	**-0.4**	0.0	-0.4	**-0.2**	0.0
Min	-0.5	**-0.3**	-0.1	-0.3	**-0.2**	0.0	-1.9	**-1.0**	0.0	-0.9	**-0.4**	0.0	-5.6	**-3.3**	-0.8
Samples		**12**			**2**			**2**			**4**			**21**	

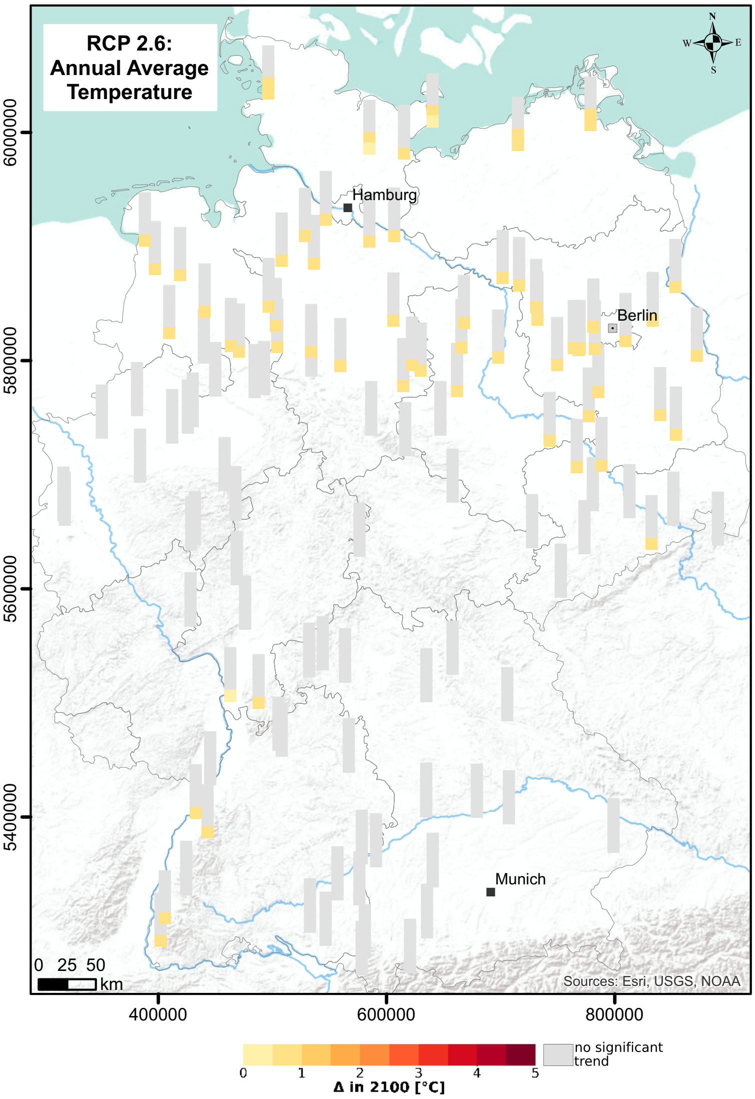

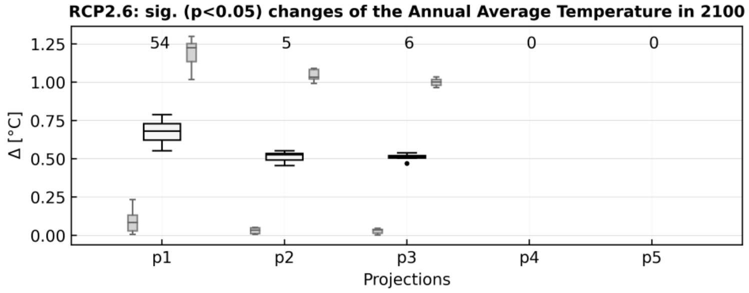

Figure S3: Input data trend analysis for the annual average temperature under **RCP2.6**. Single squares depict results of a single projection, ordered by the strength and sign of the change. Changes are considered significant for $p < 0.05$.

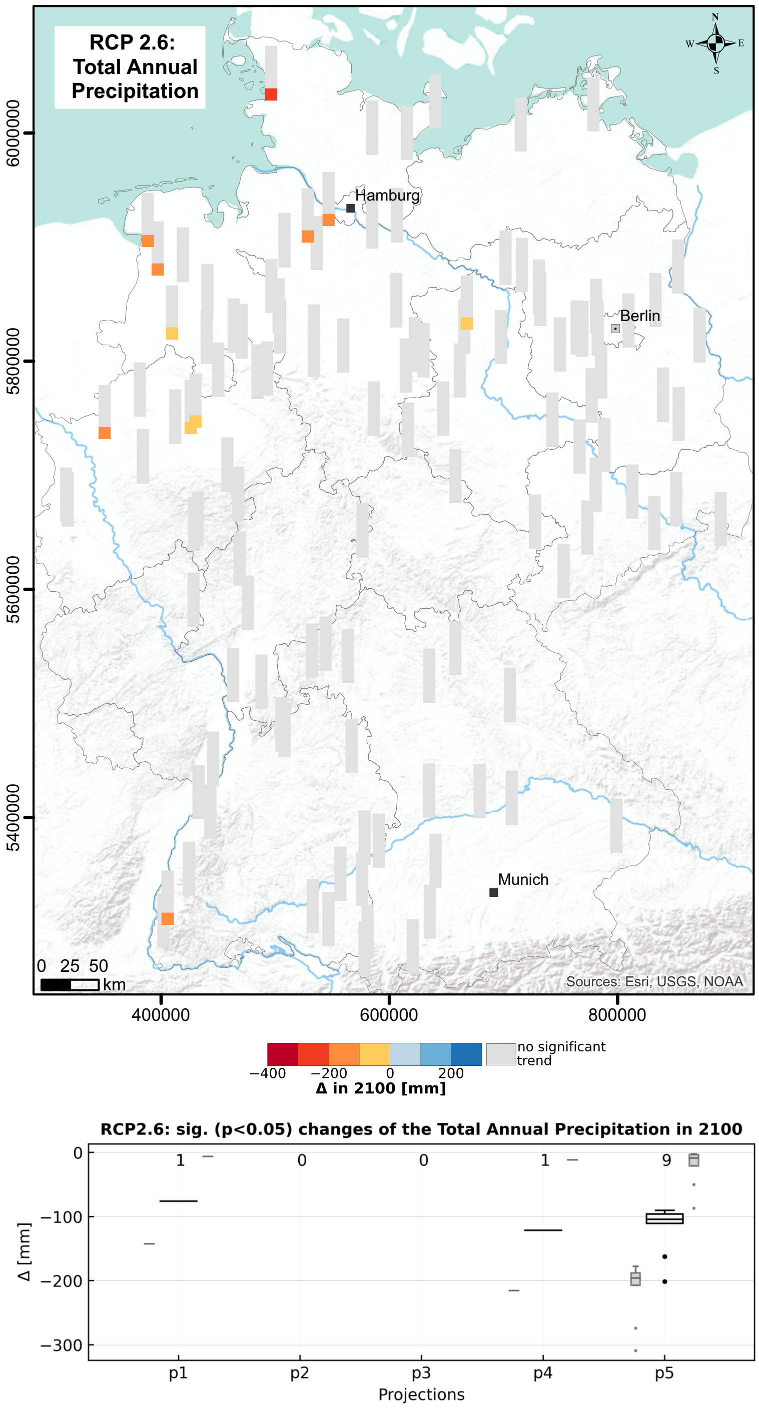

Figure S4: Input data trend analysis for the total annual precipitation under **RCP2.6**. Single squares depict results of a single projection, ordered by the strength and sign of the change. Changes are considered significant for p $<$ 0.05.

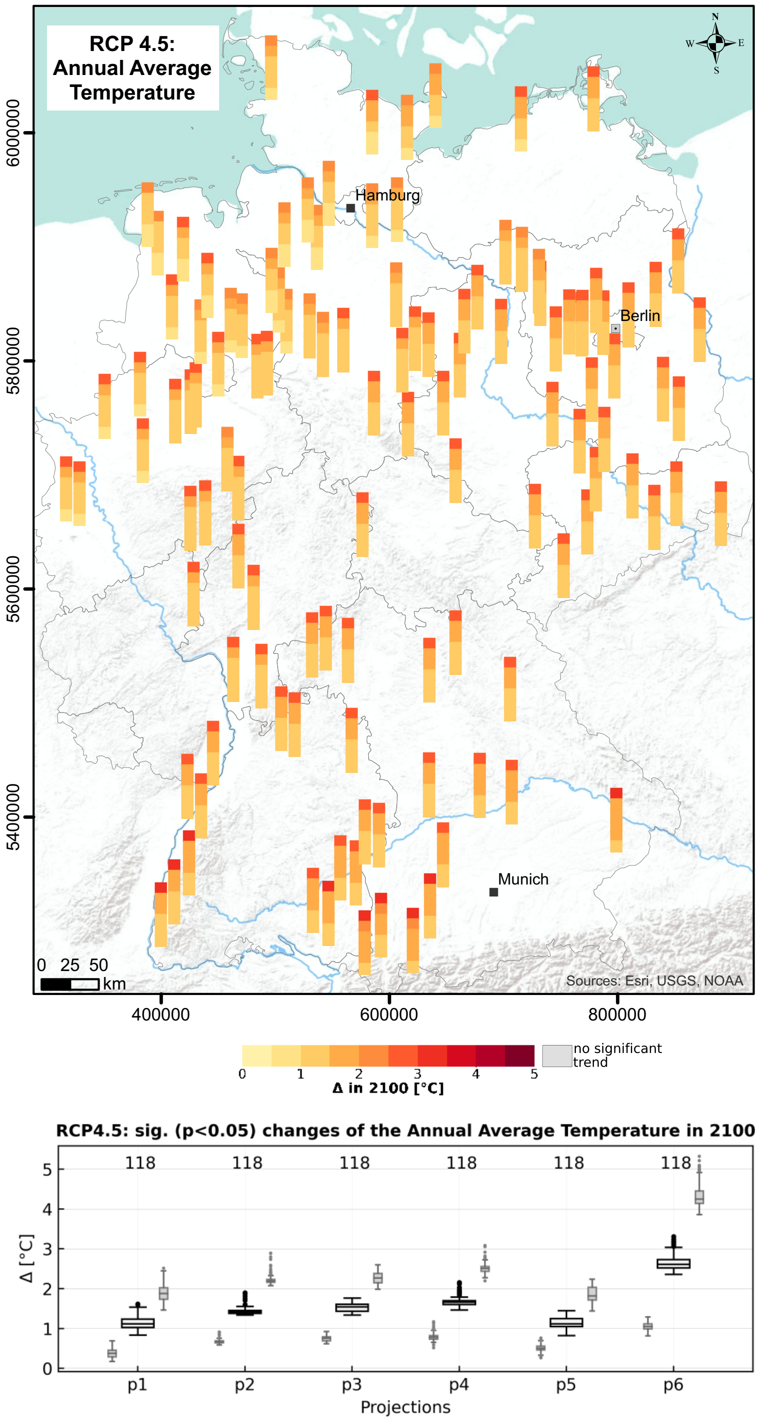

Figure S5: Input data trend analysis for the annual average temperature under **RCP4.5**. Single squares depict results of a single projection, ordered by the strength and sign of the change. Changes are considered significant for $p < 0.05$.

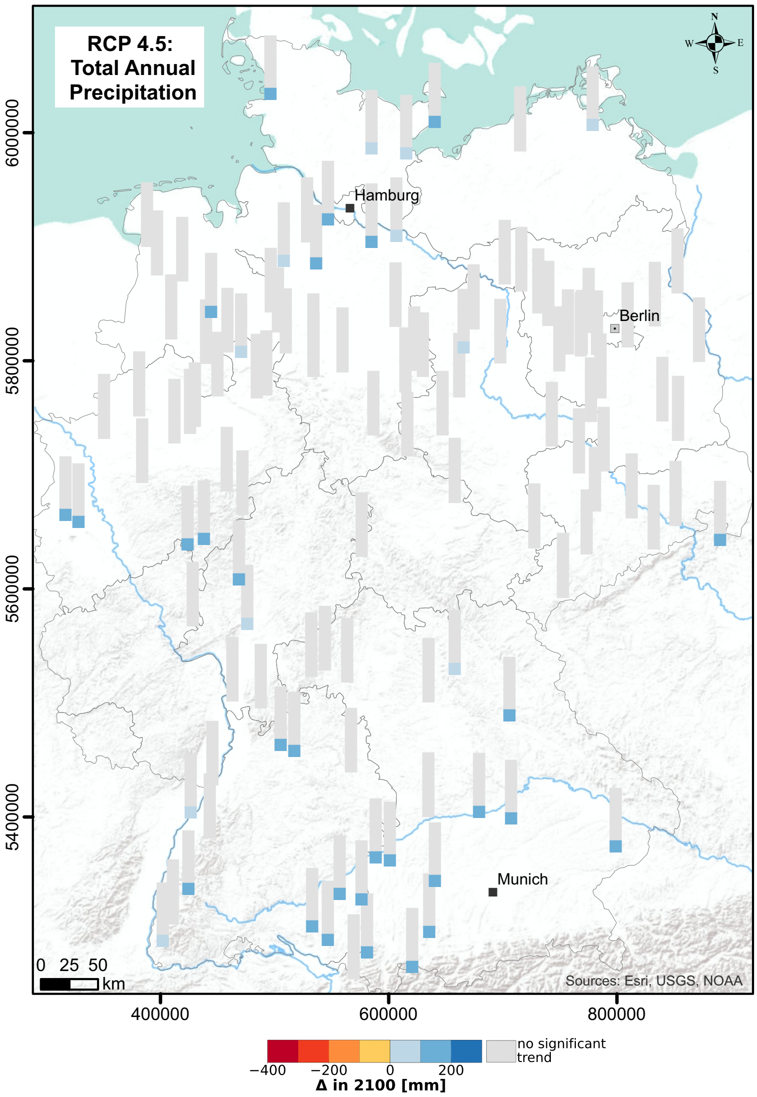

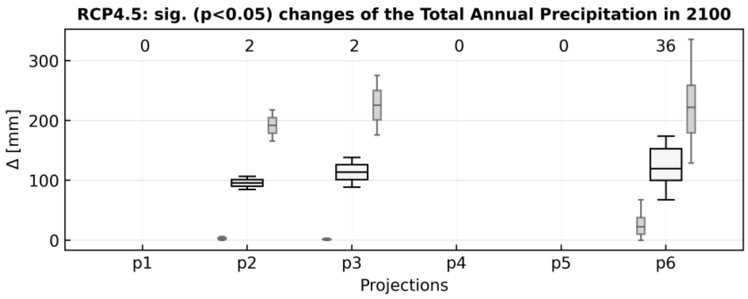

Figure S6: Input data trend analysis for the total annual precipitation under **RCP4.5**. Single squares depict results of a single projection, ordered by the strength and sign of the change. Changes are considered significant for $p < 0.05$.

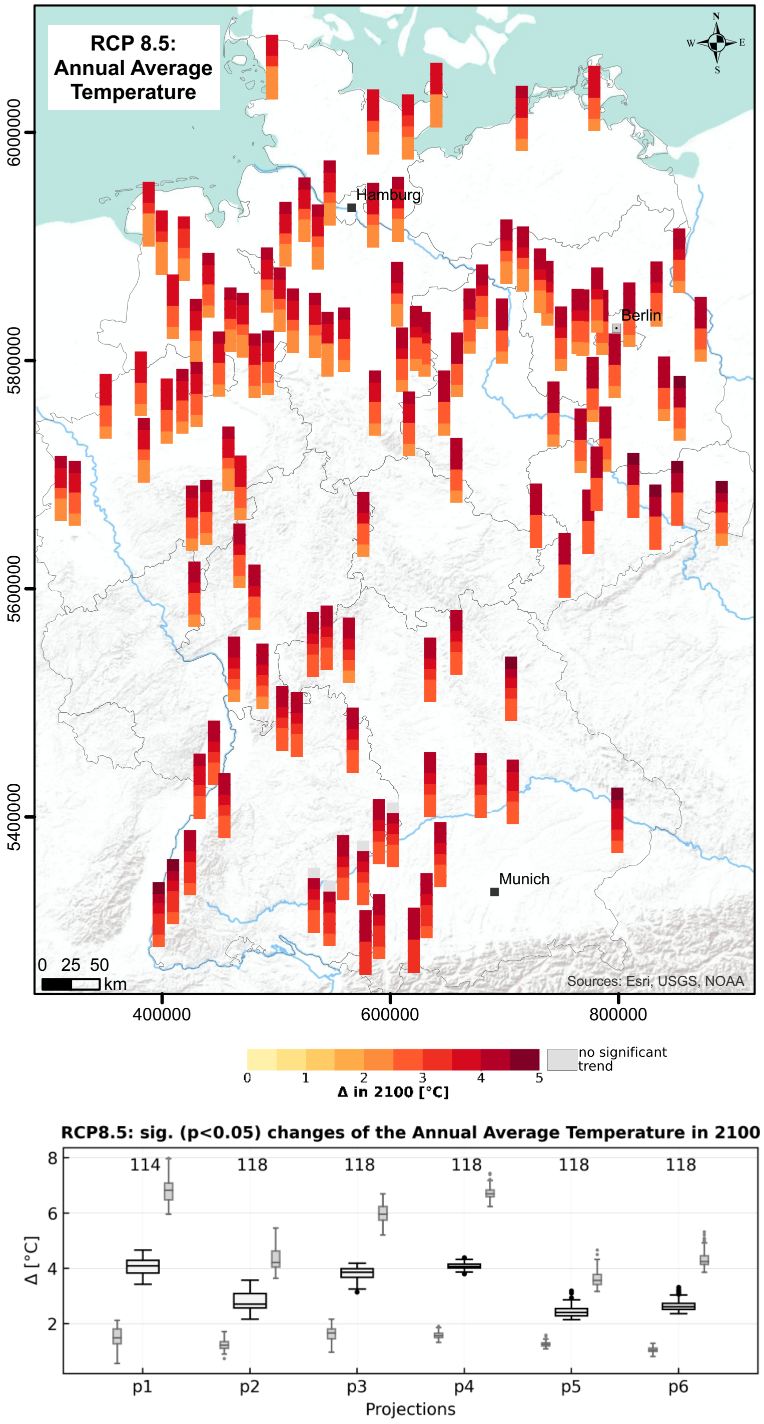

Figure S7: Input data trend analysis for the annual average temperature under **RCP8.5**. Single squares depict results of a single projection, ordered by the strength and sign of the change. Changes are considered significant for $p < 0.05$.

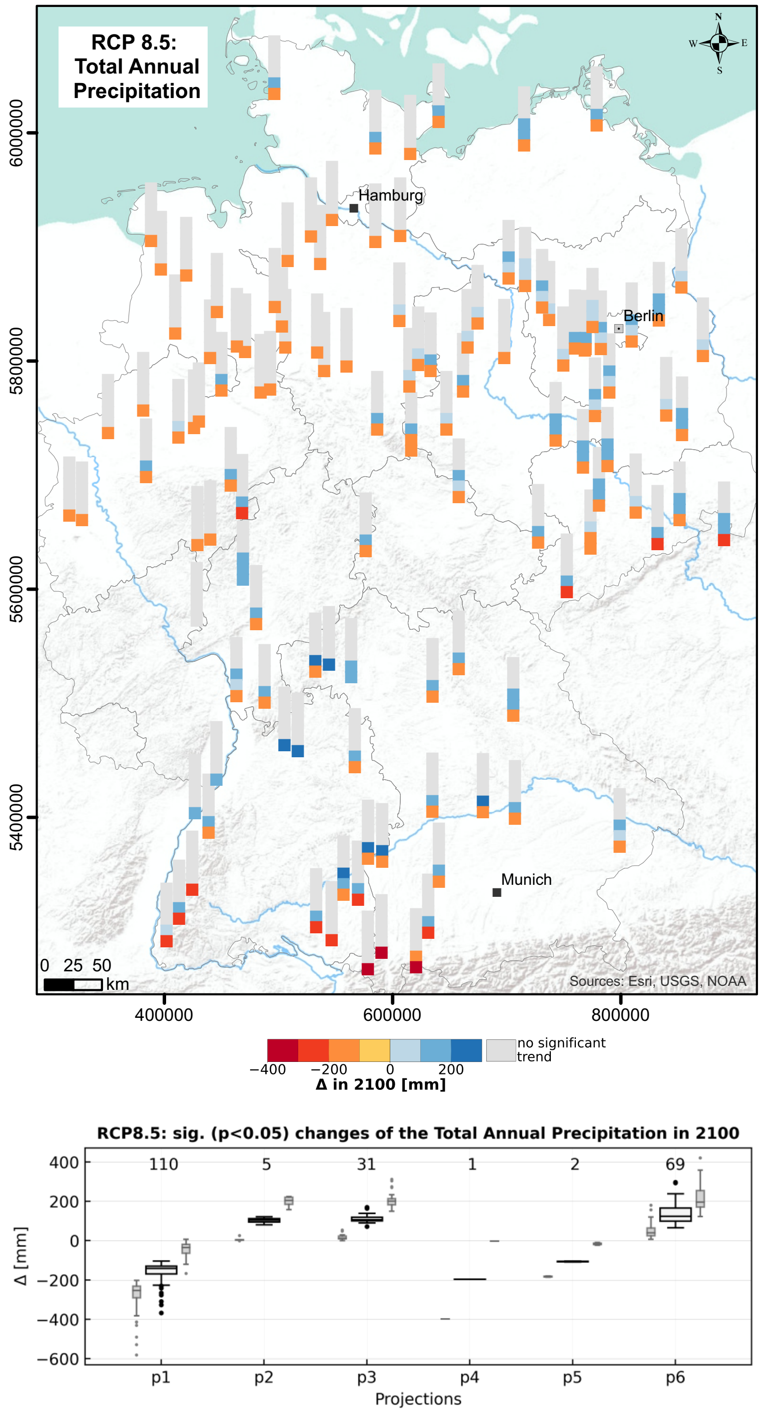

Figure S8: Input data trend analysis for the total annual precipitation under **RCP8.5**. Single squares depict results of a single projection, ordered by the strength and sign of the change. Changes are considered significant for $p < 0.05$.

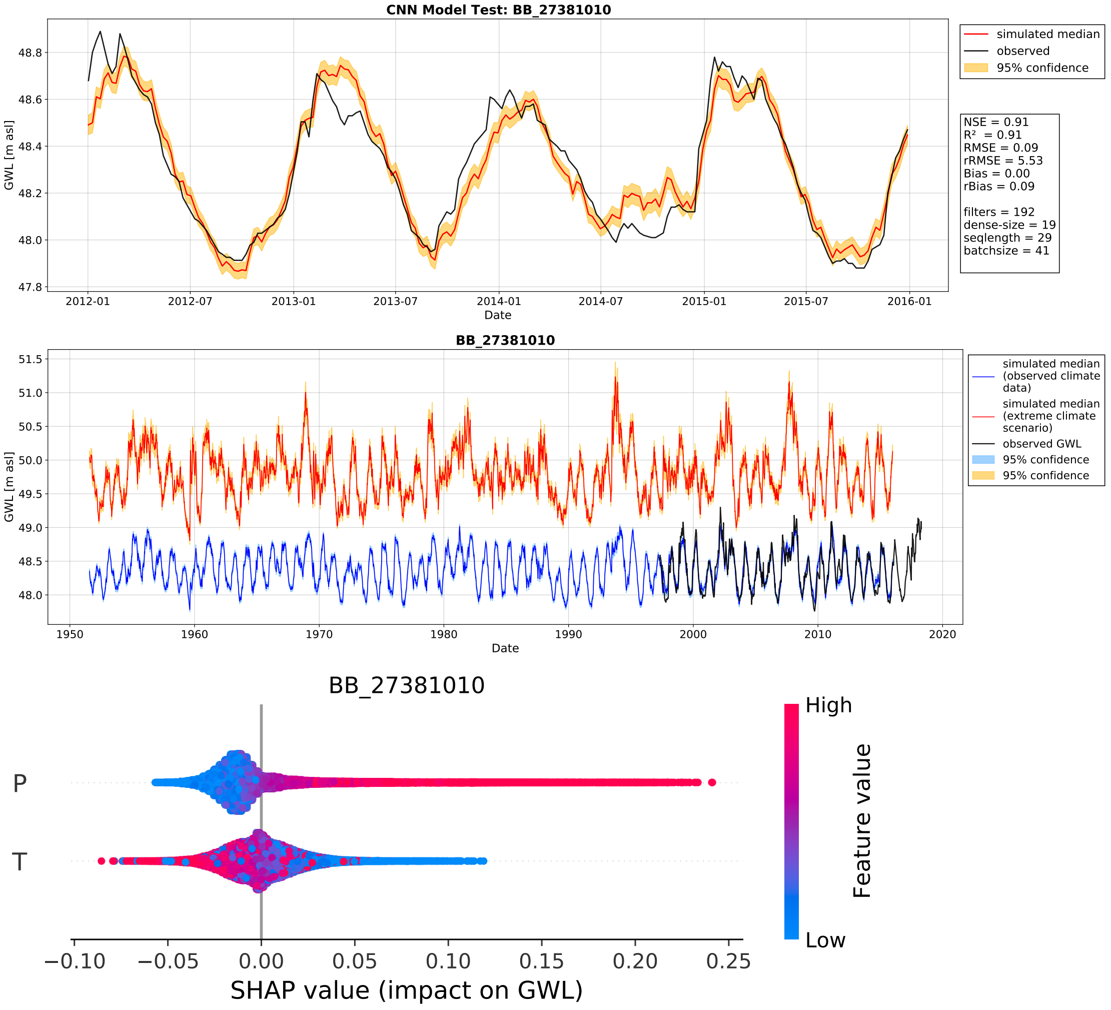

Figure S9: Evaluation of BB_27381010 Model Performance in the past (upper), under extreme climate conditions (middle) and SHAP Summary plot (lower)

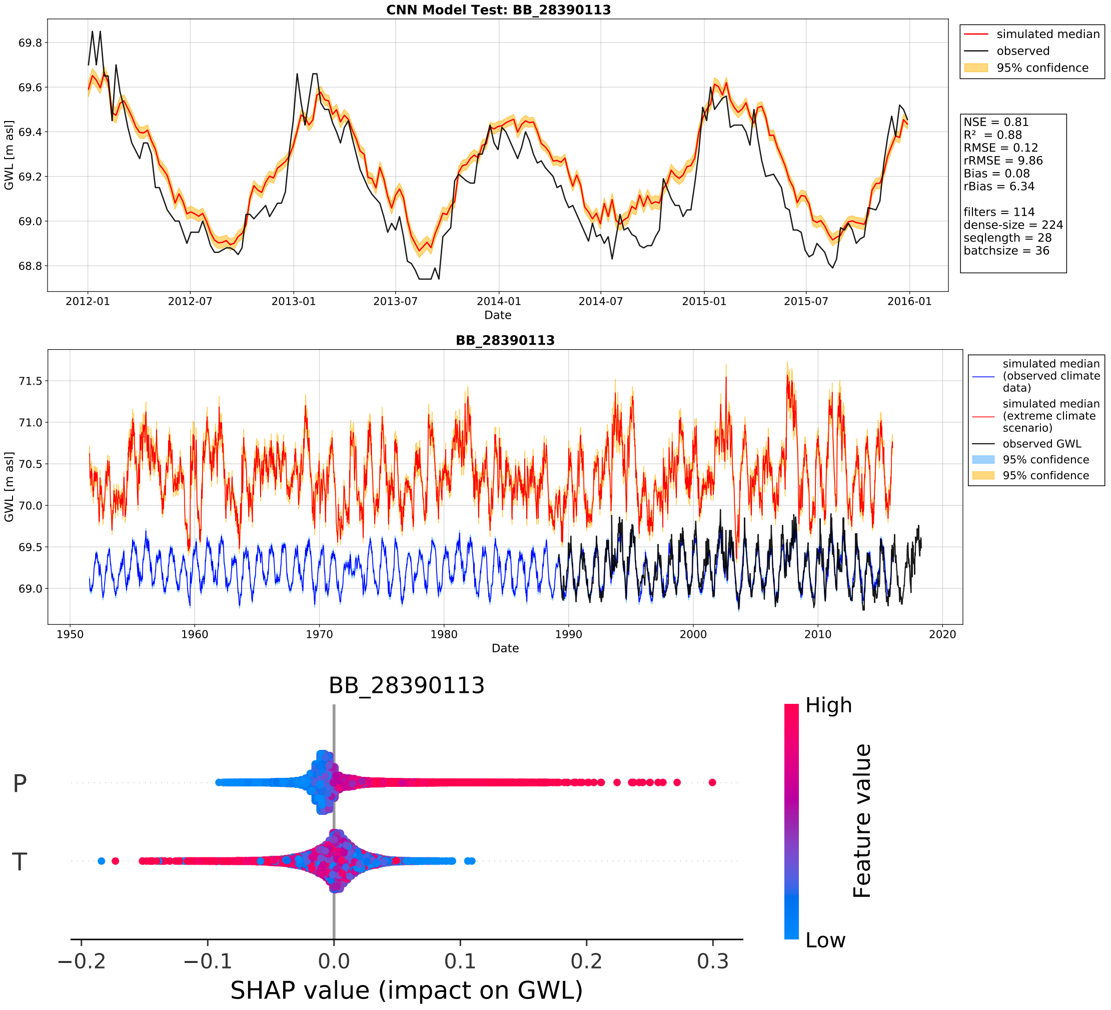

Figure S10: Evaluation of BB_28390113 Model Performance in the past (upper), under extreme climate conditions (middle) and SHAP Summary plot (lower)

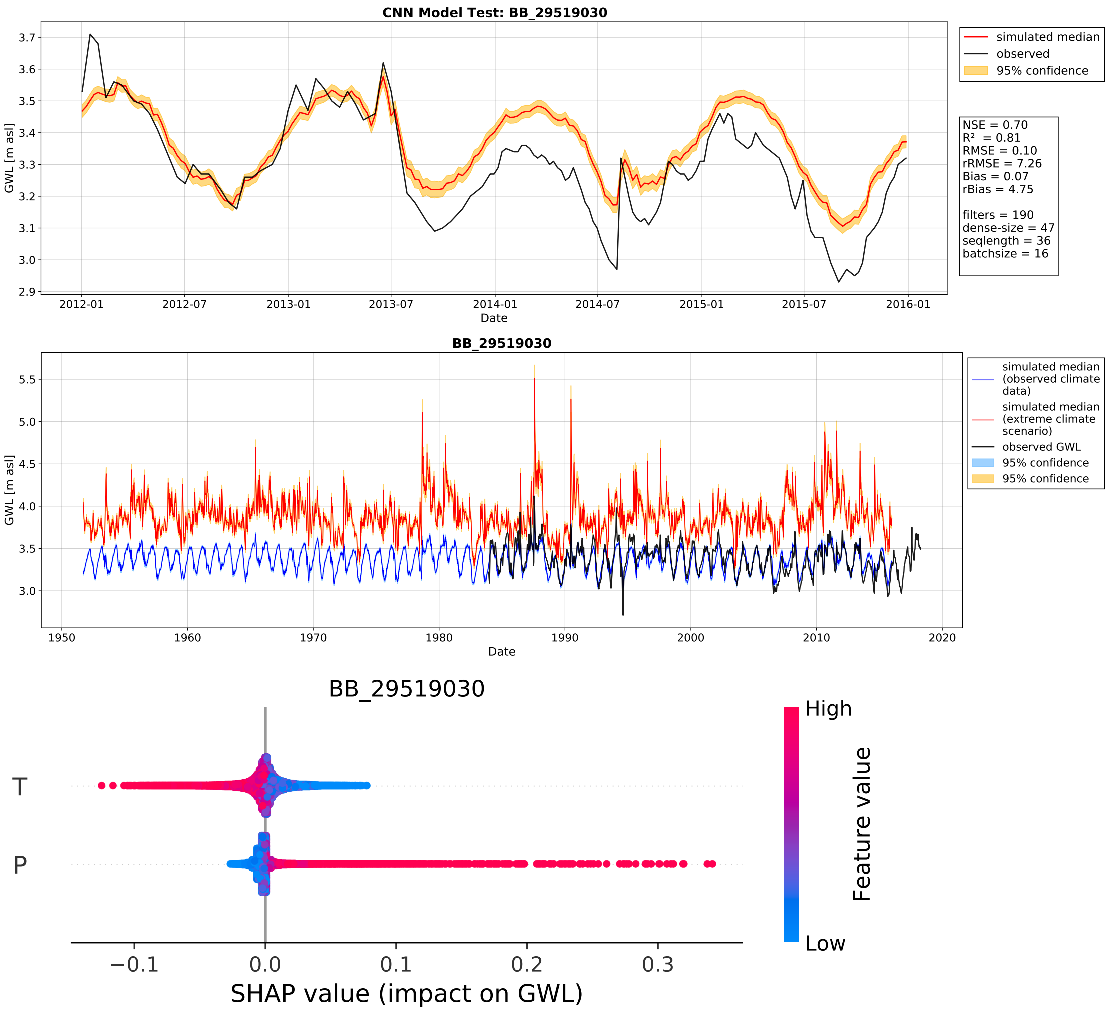

Figure S11: Evaluation of BB_29519030 Model Performance in the past (upper), under extreme climate conditions (middle) and SHAP Summary plot (lower)

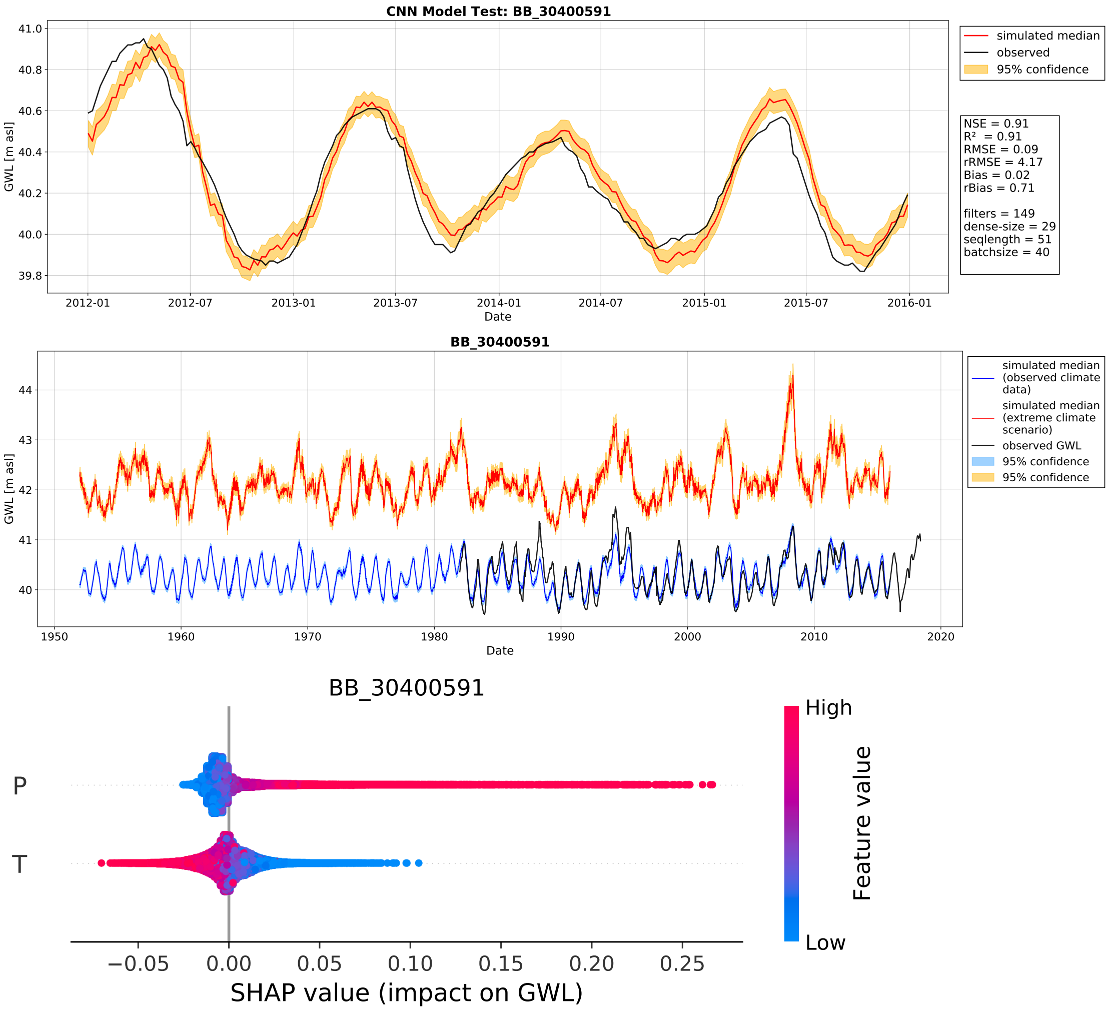

Figure S12: Evaluation of BB_30400591 Model Performance in the past (upper), under extreme climate conditions (middle) and SHAP Summary plot (lower)

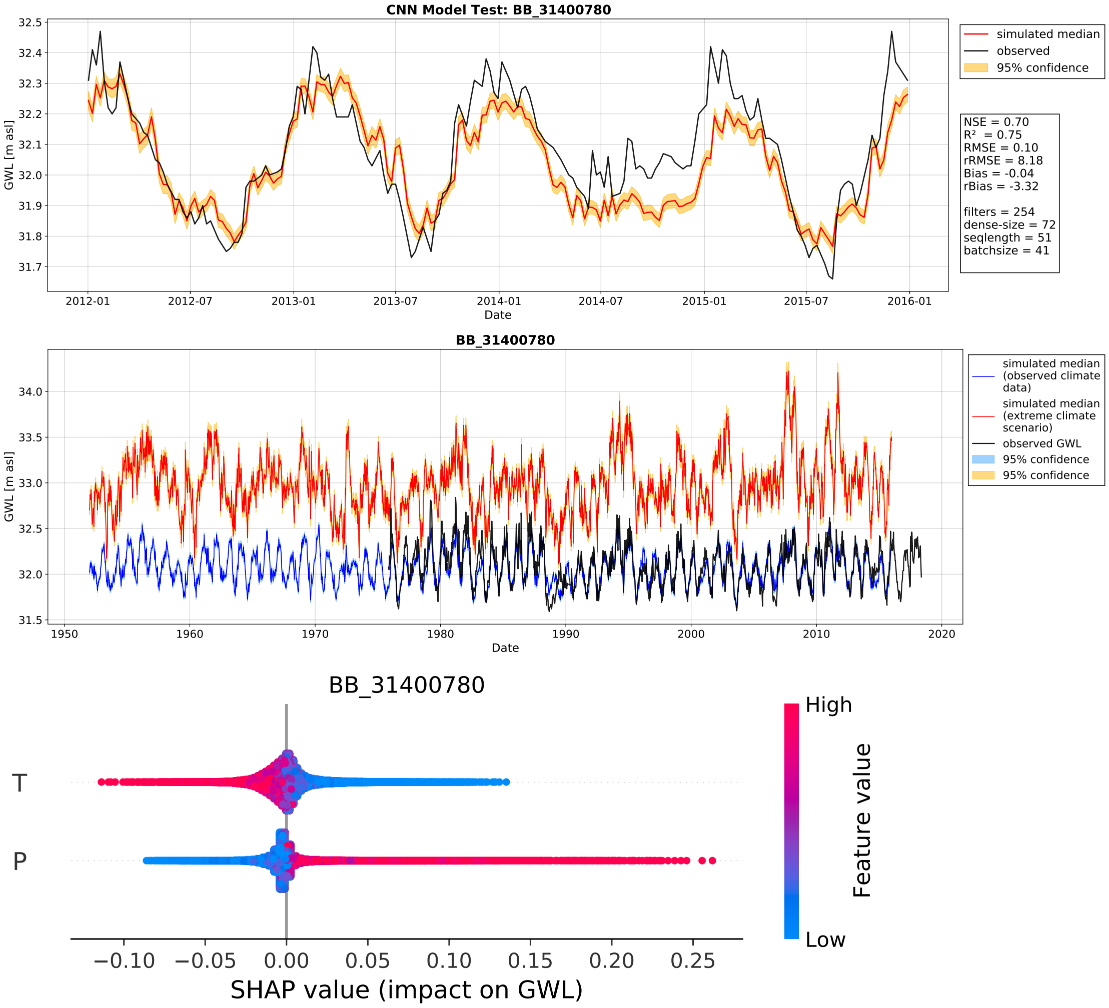

Figure S13: Evaluation of BB_31400780 Model Performance in the past (upper), under extreme climate conditions (middle) and SHAP Summary plot (lower)

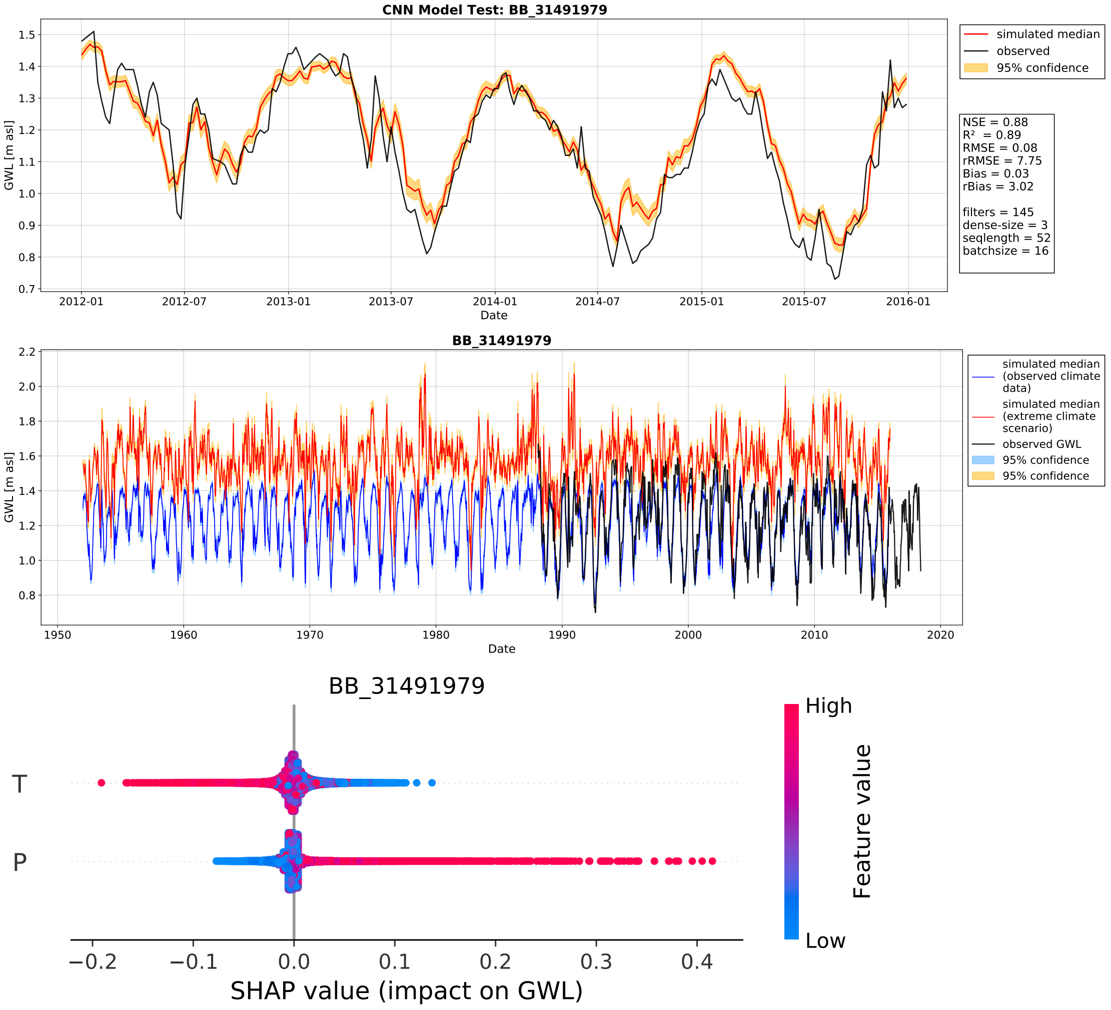

Figure S14: Evaluation of BB_31491979 Model Performance in the past (upper), under extreme climate conditions (middle) and SHAP Summary plot (lower)

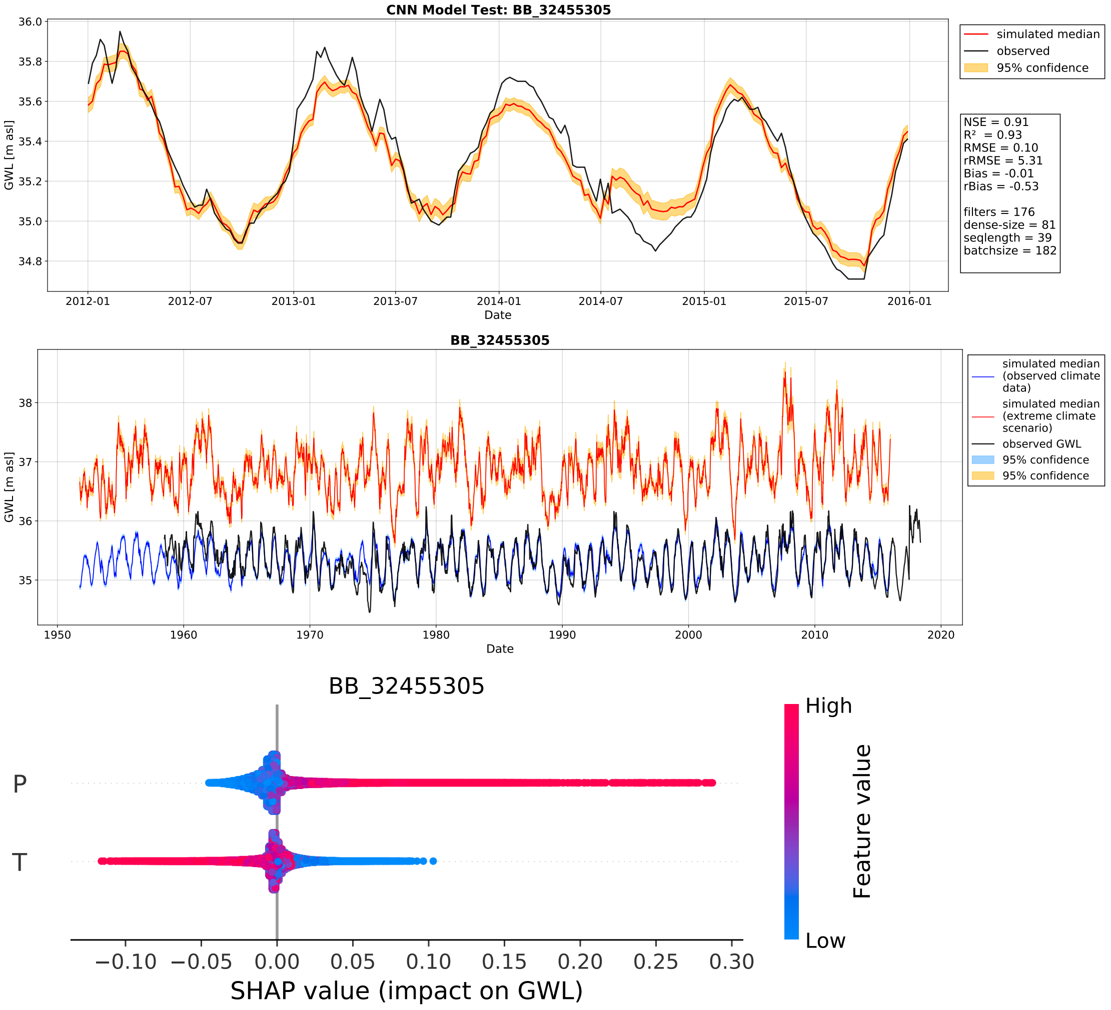

Figure S15: Evaluation of BB_32455305 Model Performance in the past (upper), under extreme climate conditions (middle) and SHAP Summary plot (lower)

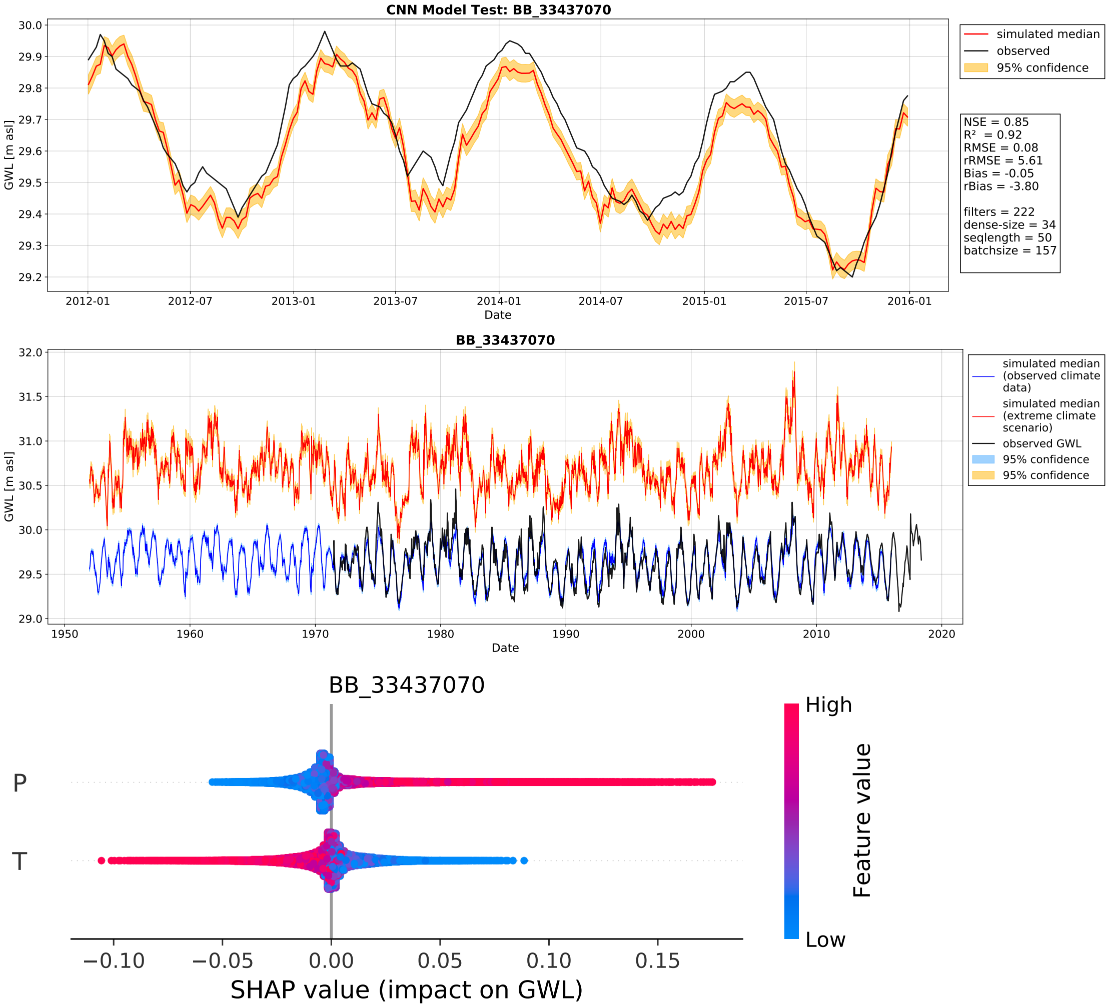

Figure S16: Evaluation of BB_33437070 Model Performance in the past (upper), under extreme climate conditions (middle) and SHAP Summary plot (lower)

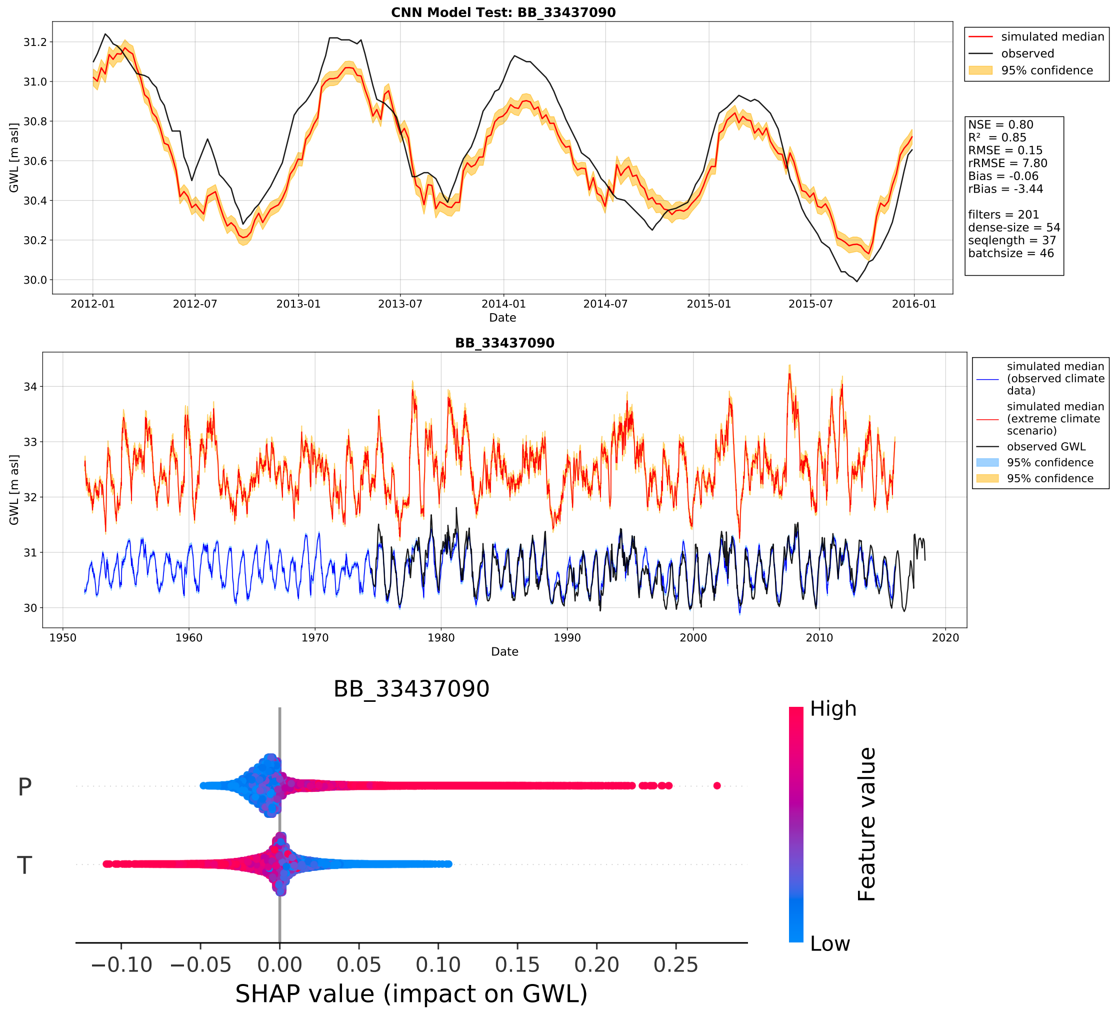

Figure S17: Evaluation of BB_33437090 Model Performance in the past (upper), under extreme climate conditions (middle) and SHAP Summary plot (lower)

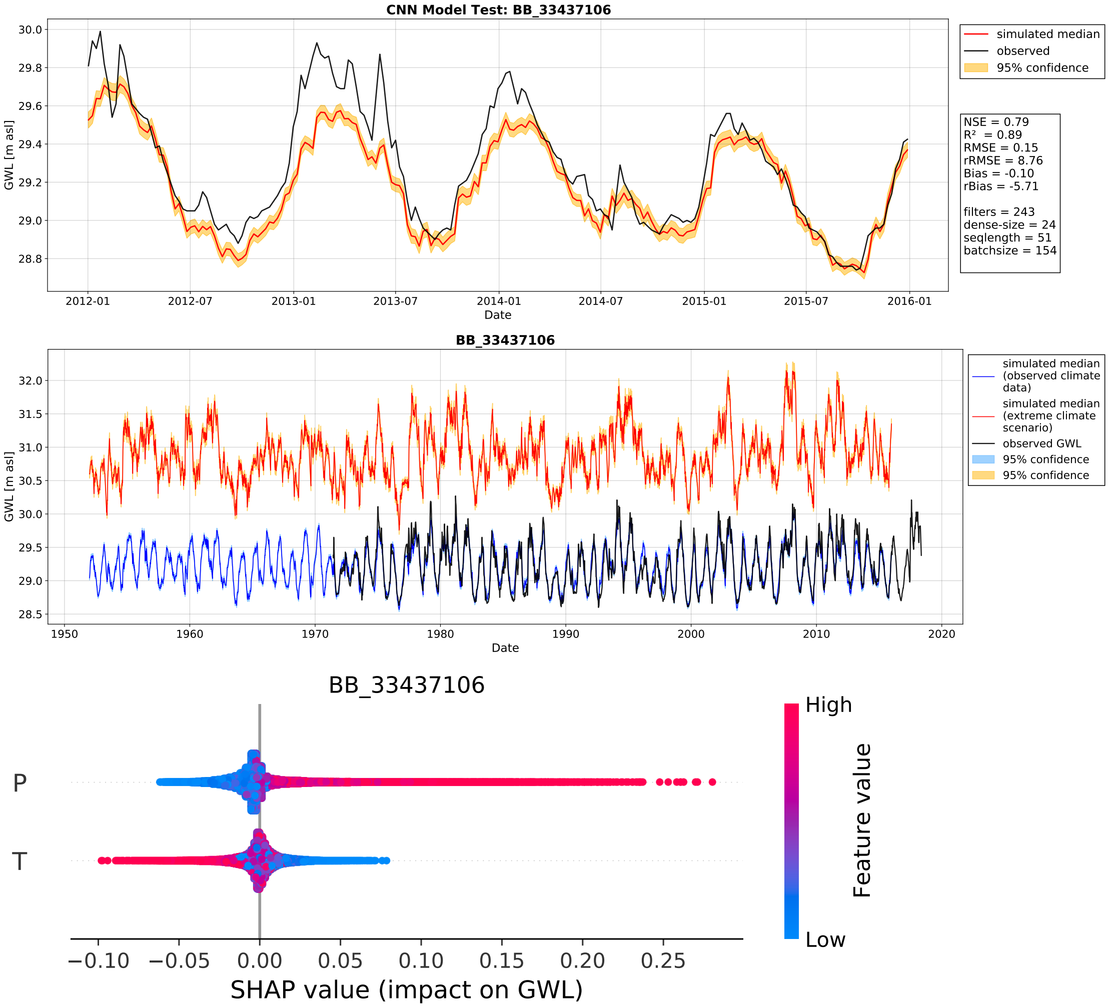

Figure S18: Evaluation of BB_33437106 Model Performance in the past (upper), under extreme climate conditions (middle) and SHAP Summary plot (lower)

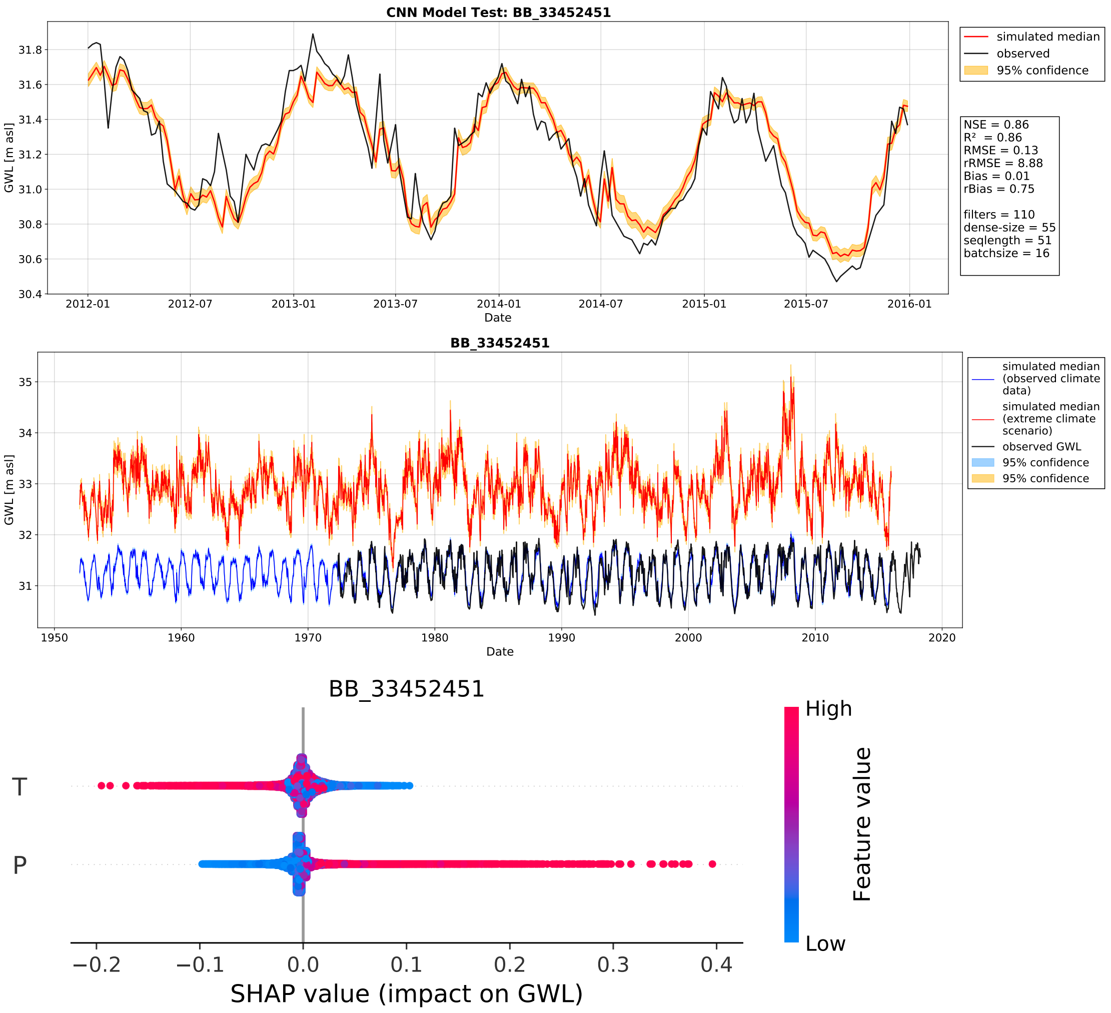

Figure S19: Evaluation of BB_33452451 Model Performance in the past (upper), under extreme climate conditions (middle) and SHAP Summary plot (lower)

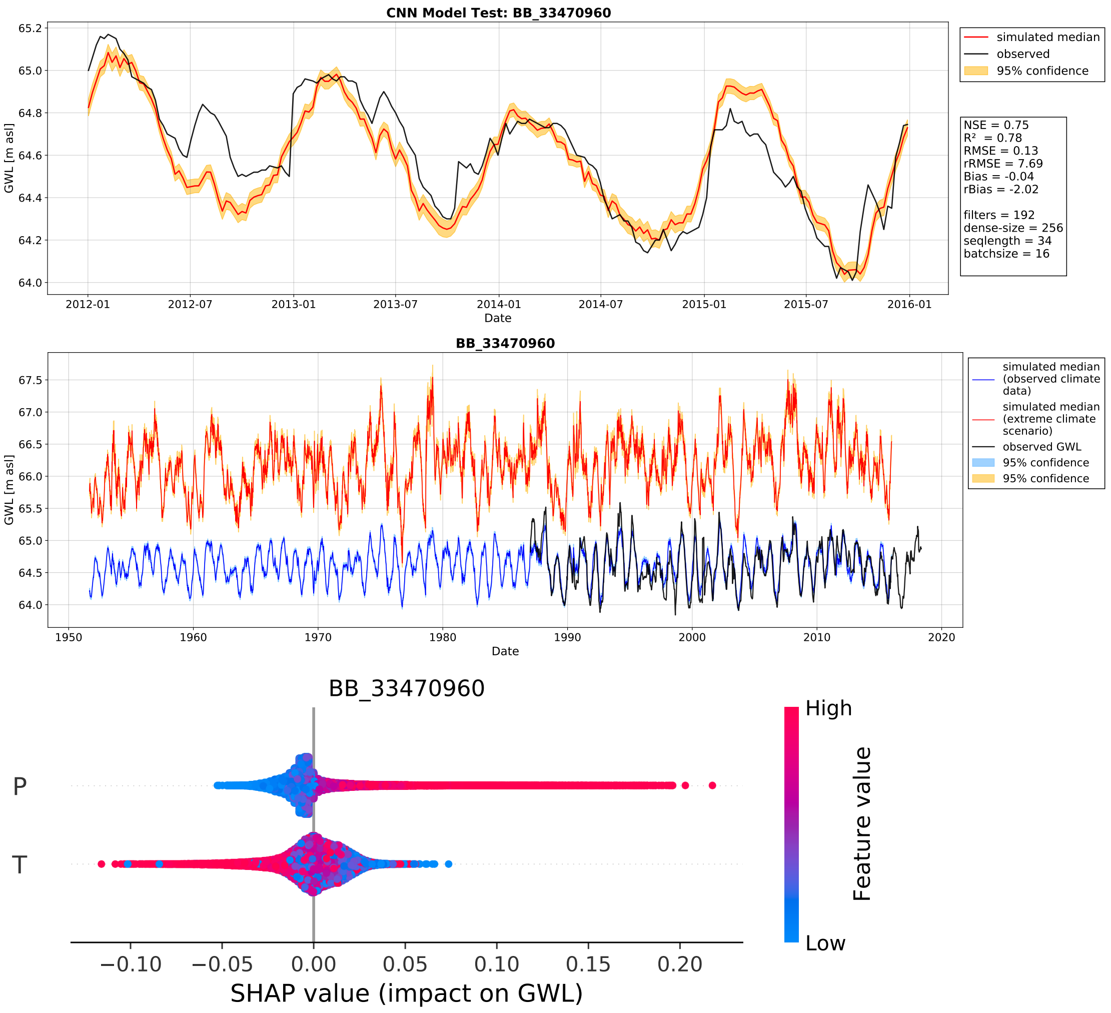

Figure S20: Evaluation of BB_33470960 Model Performance in the past (upper), under extreme climate conditions (middle) and SHAP Summary plot (lower)

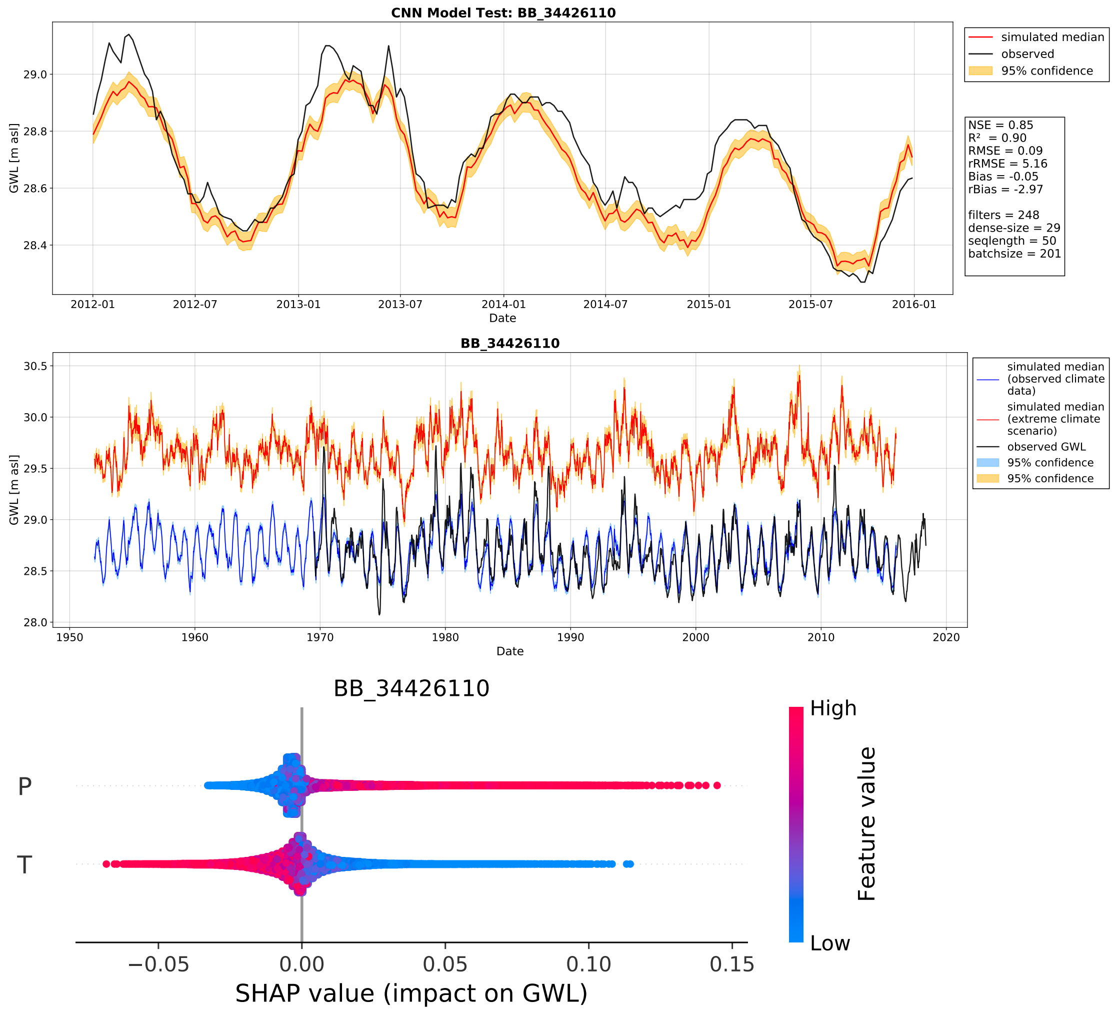

Figure S21: Evaluation of BB_34426110 Model Performance in the past (upper), under extreme climate conditions (middle) and SHAP Summary plot (lower)

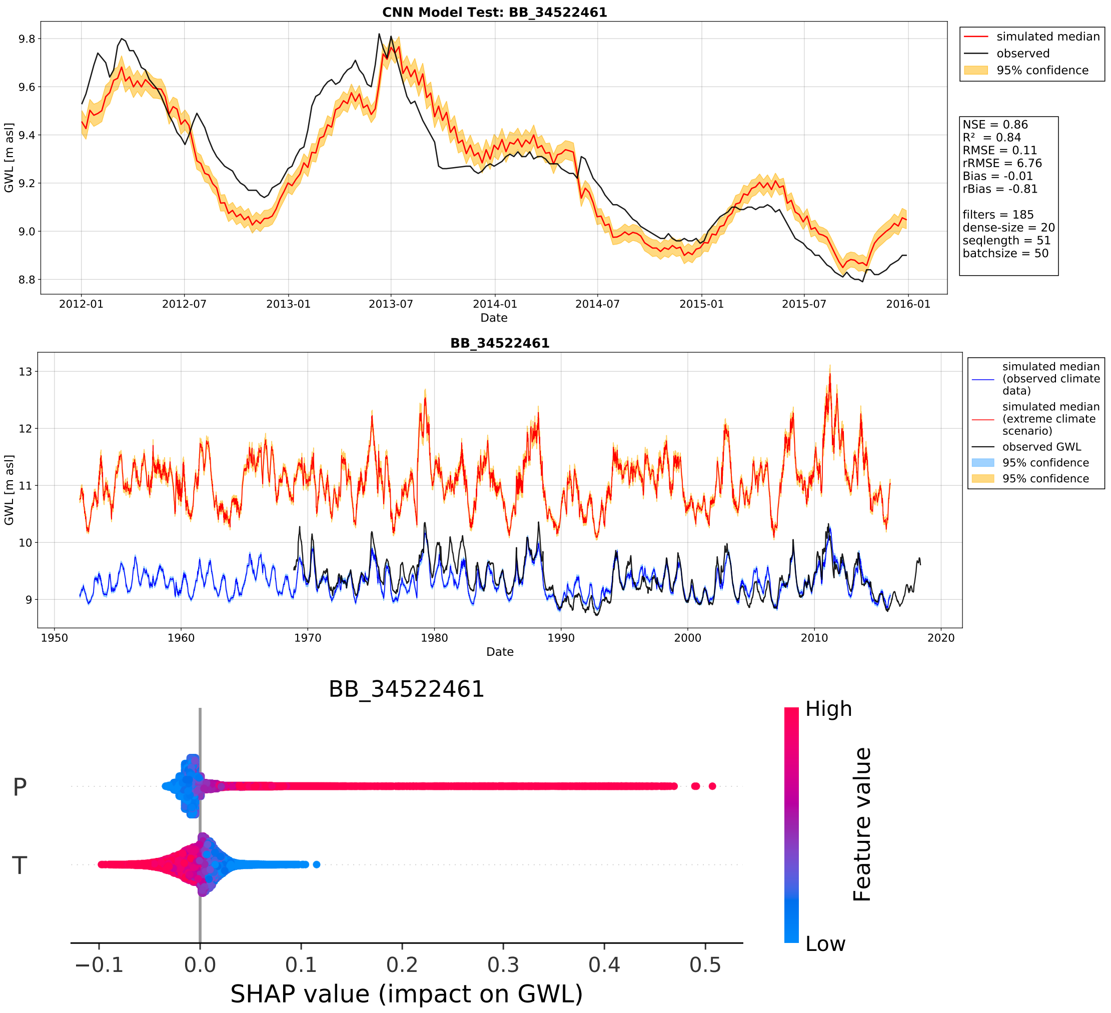

Figure S22: Evaluation of BB_34522461 Model Performance in the past (upper), under extreme climate conditions (middle) and SHAP Summary plot (lower)

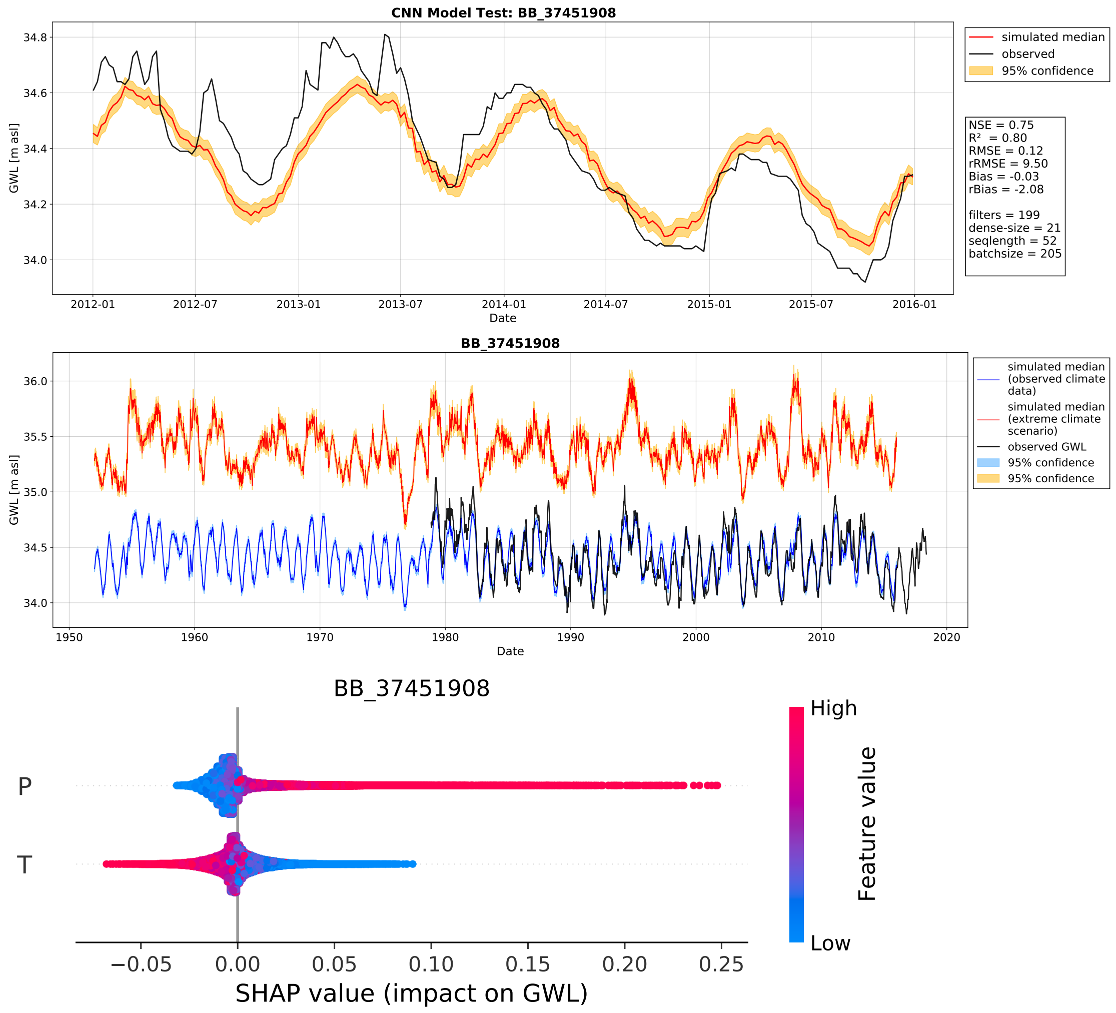

Figure S23: Evaluation of BB_37451908 Model Performance in the past (upper), under extreme climate conditions (middle) and SHAP Summary plot (lower)

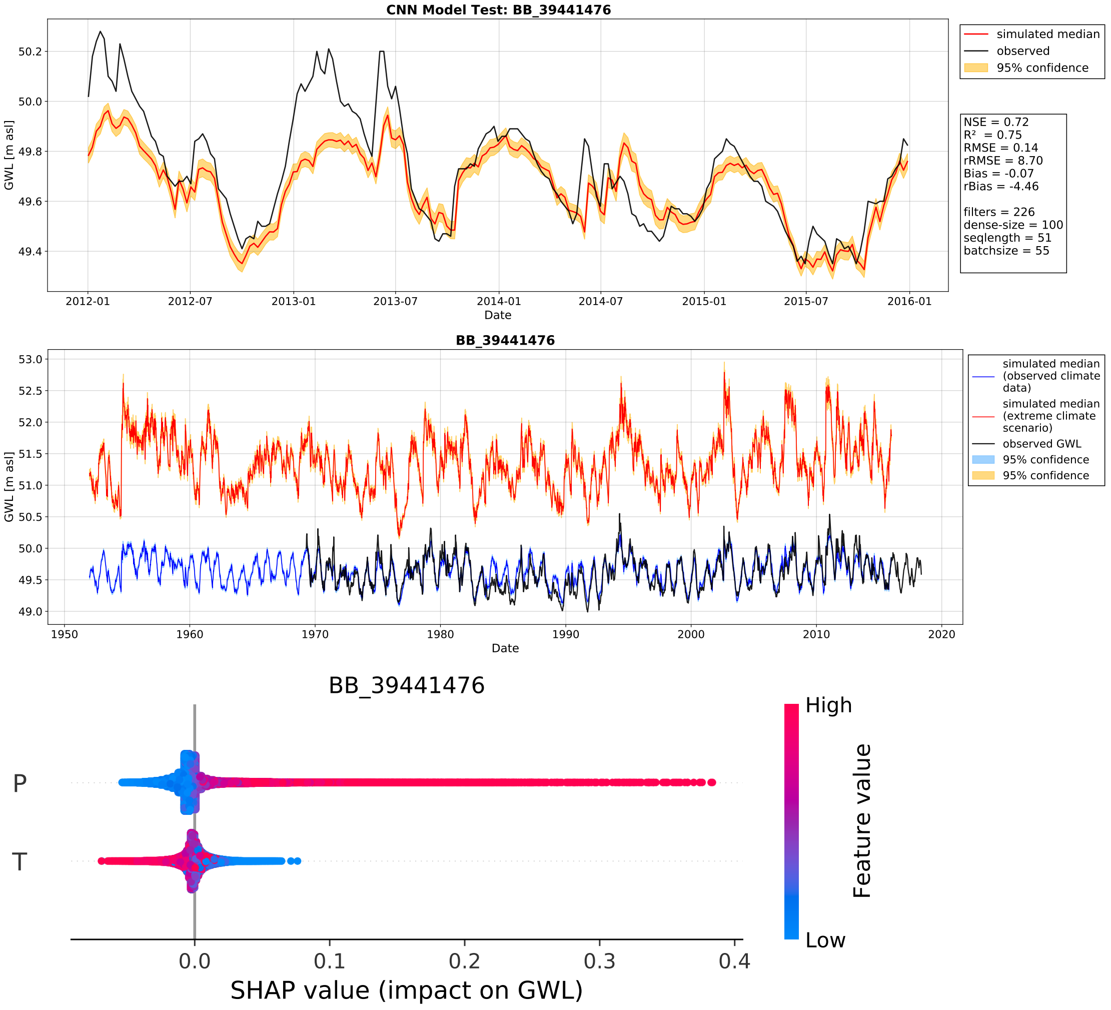

Figure S24: Evaluation of BB_39441476 Model Performance in the past (upper), under extreme climate conditions (middle) and SHAP Summary plot (lower)

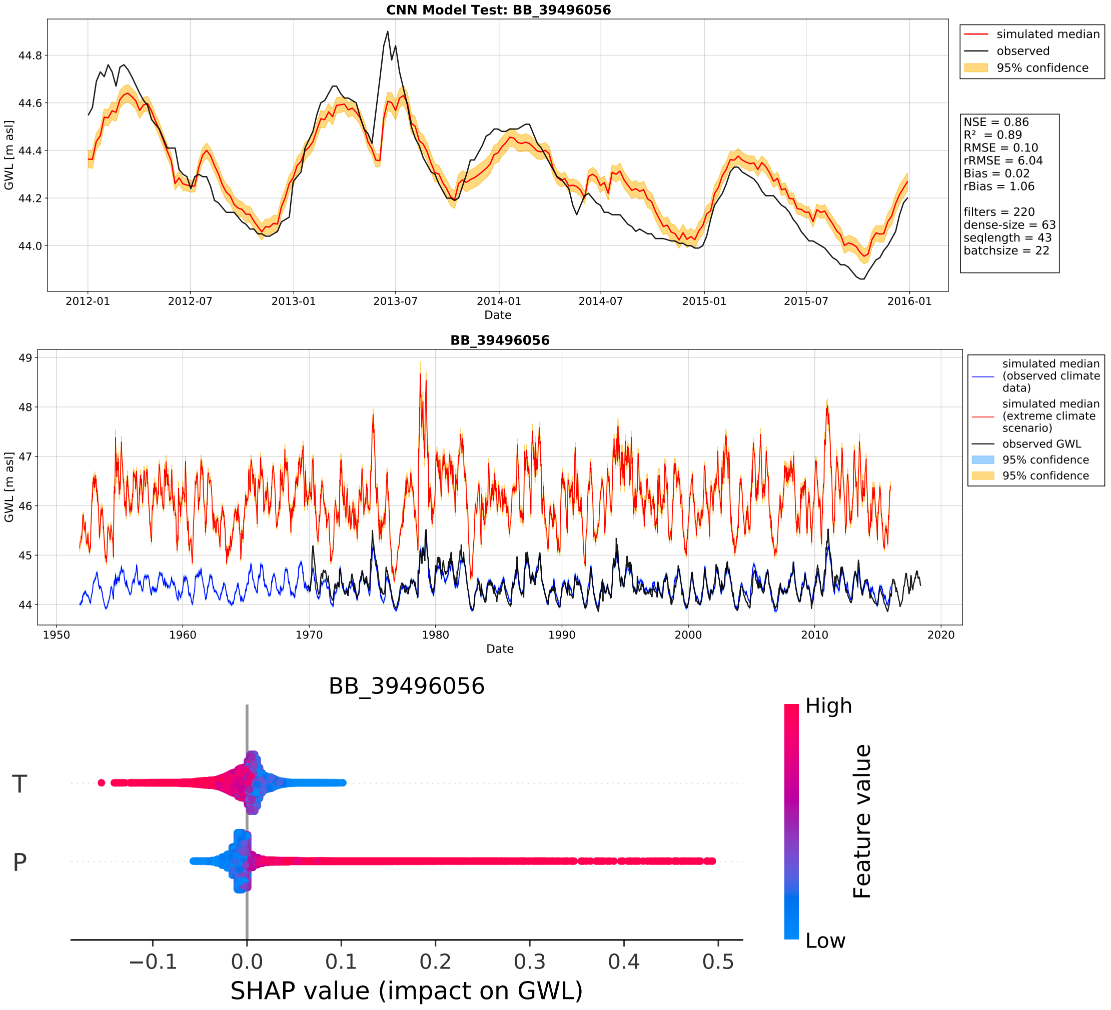

Figure S25: Evaluation of BB_39496056 Model Performance in the past (upper), under extreme climate conditions (middle) and SHAP Summary plot (lower)

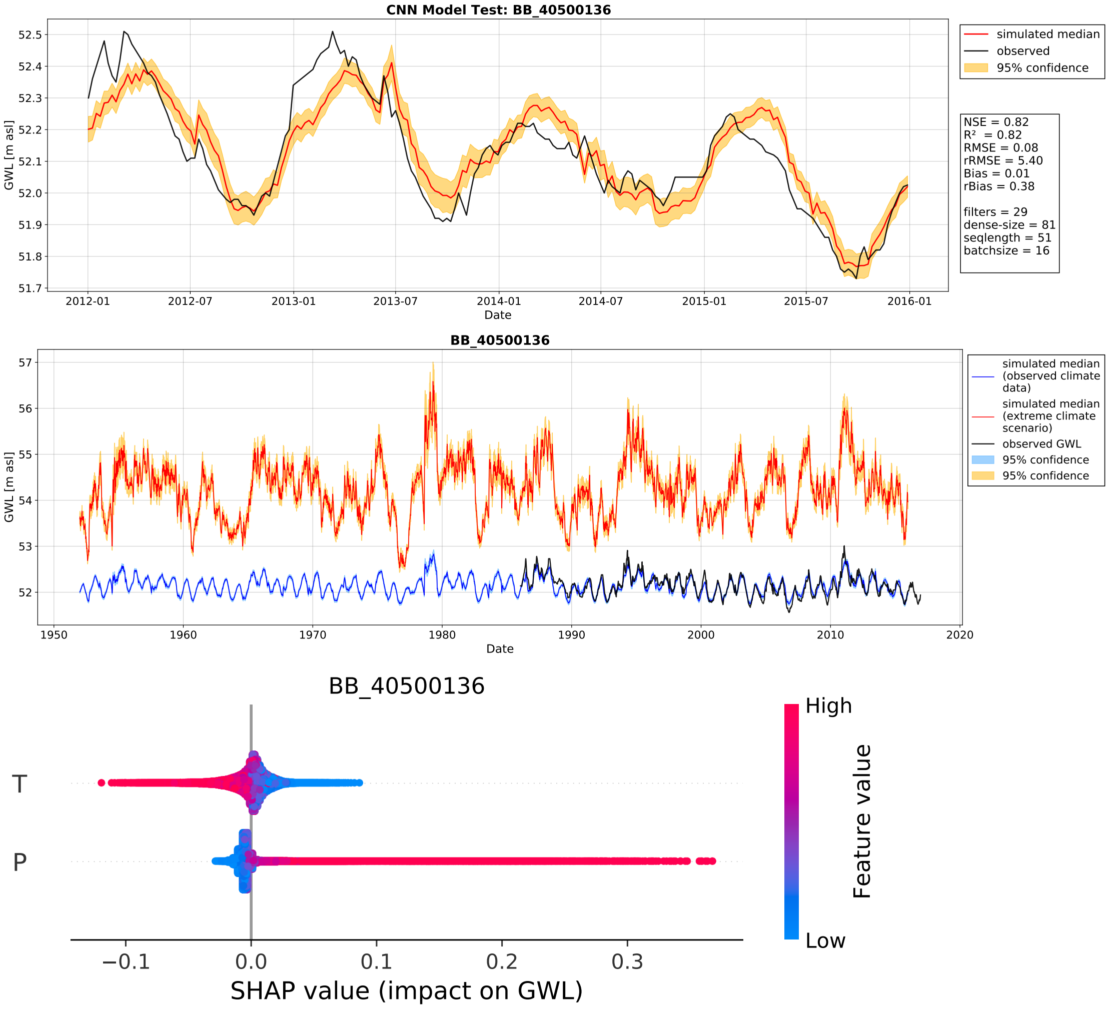

Figure S26: Evaluation of BB_40500136 Model Performance in the past (upper), under extreme climate conditions (middle) and SHAP Summary plot (lower)

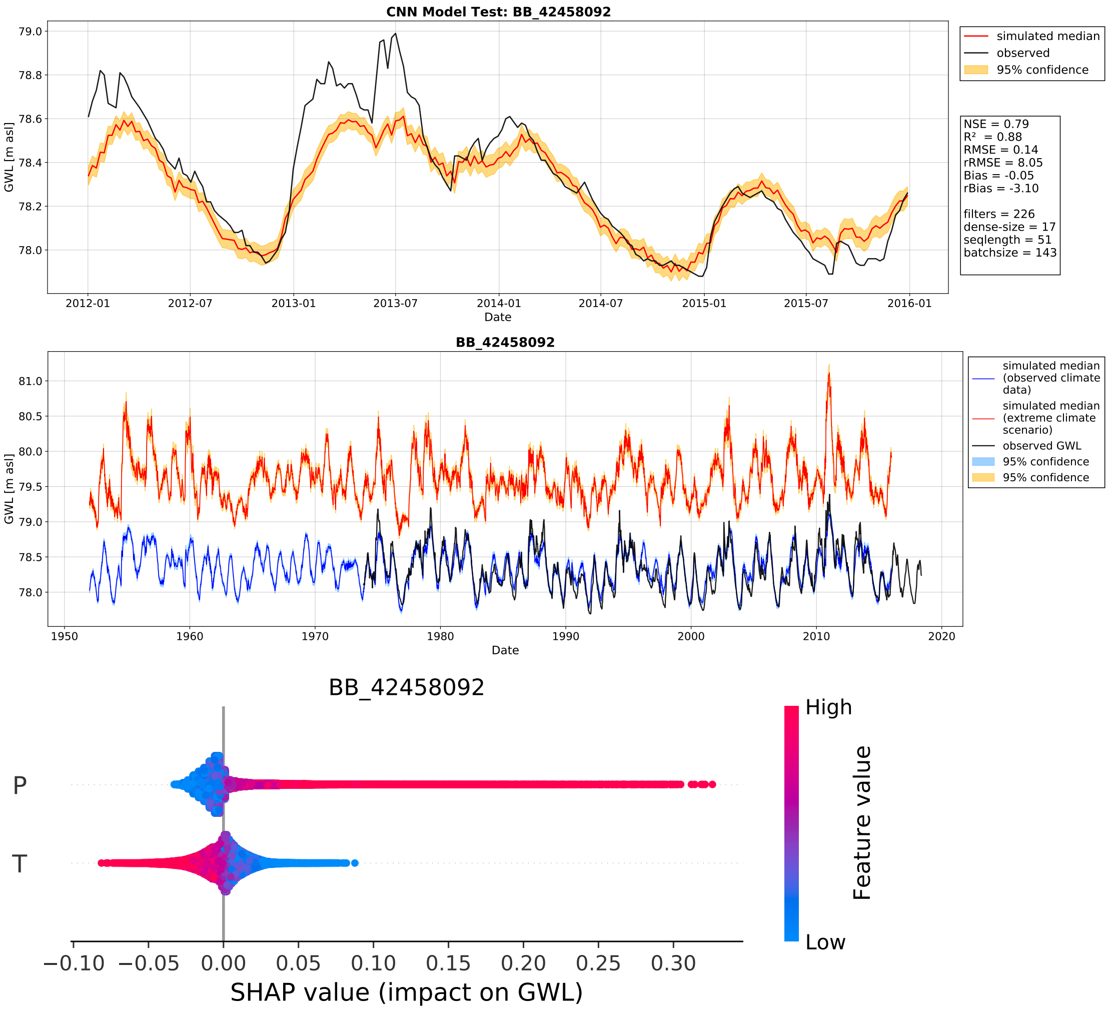

Figure S27: Evaluation of BB_42458092 Model Performance in the past (upper), under extreme climate conditions (middle) and SHAP Summary plot (lower)

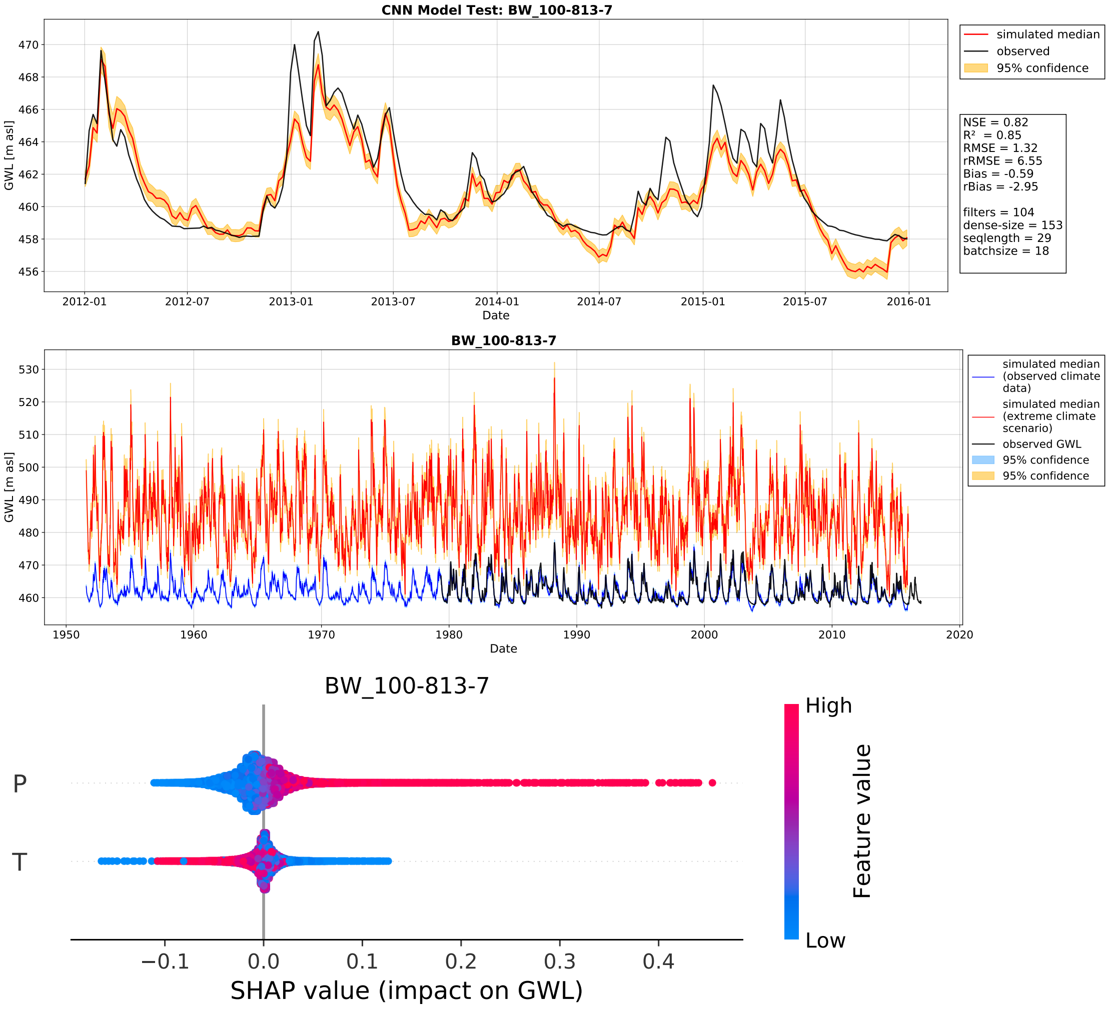

Figure S28: Evaluation of BW_100-813-7 Model Performance in the past (upper), under extreme climate conditions (middle) and SHAP Summary plot (lower)

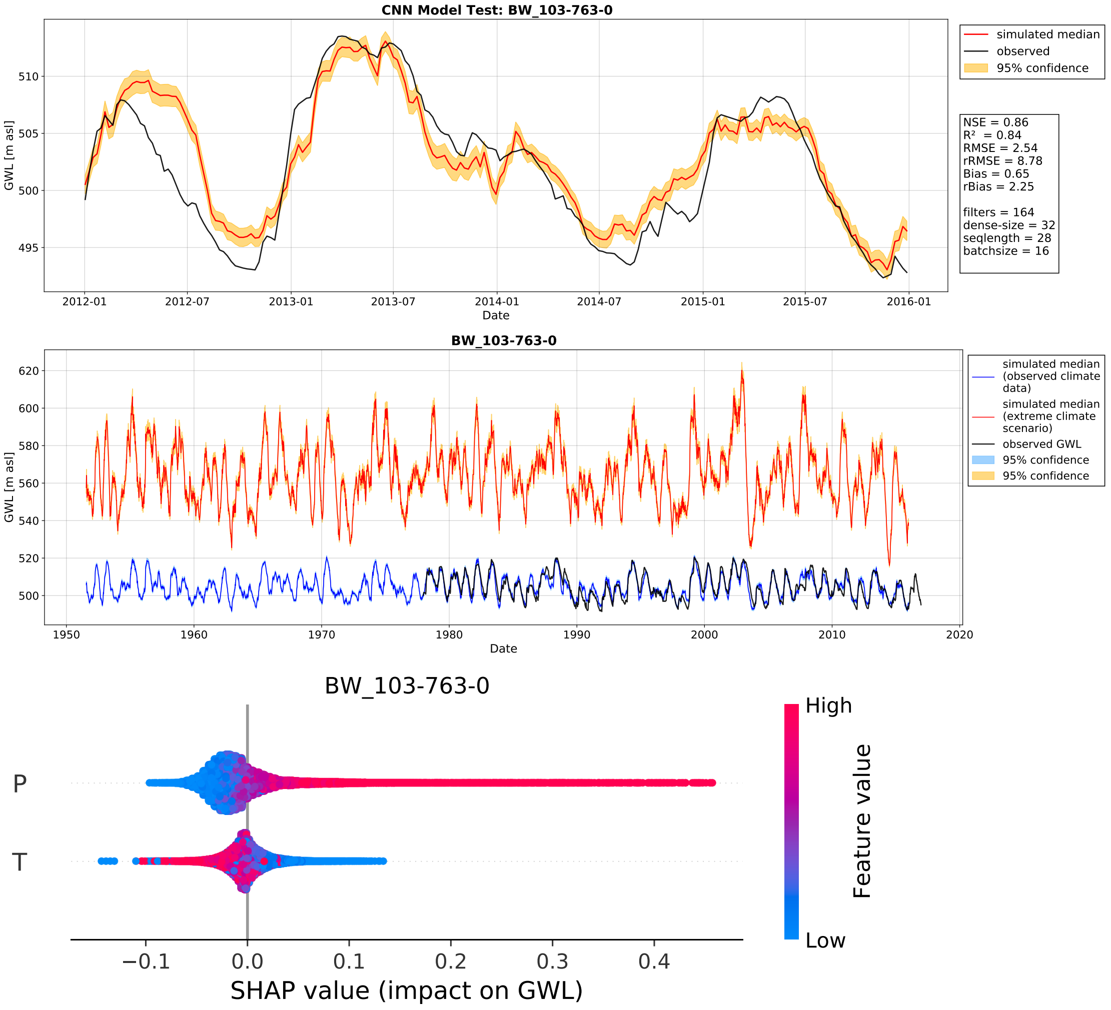

Figure S29: Evaluation of BW_103-763-0 Model Performance in the past (upper), under extreme climate conditions (middle) and SHAP Summary plot (lower)

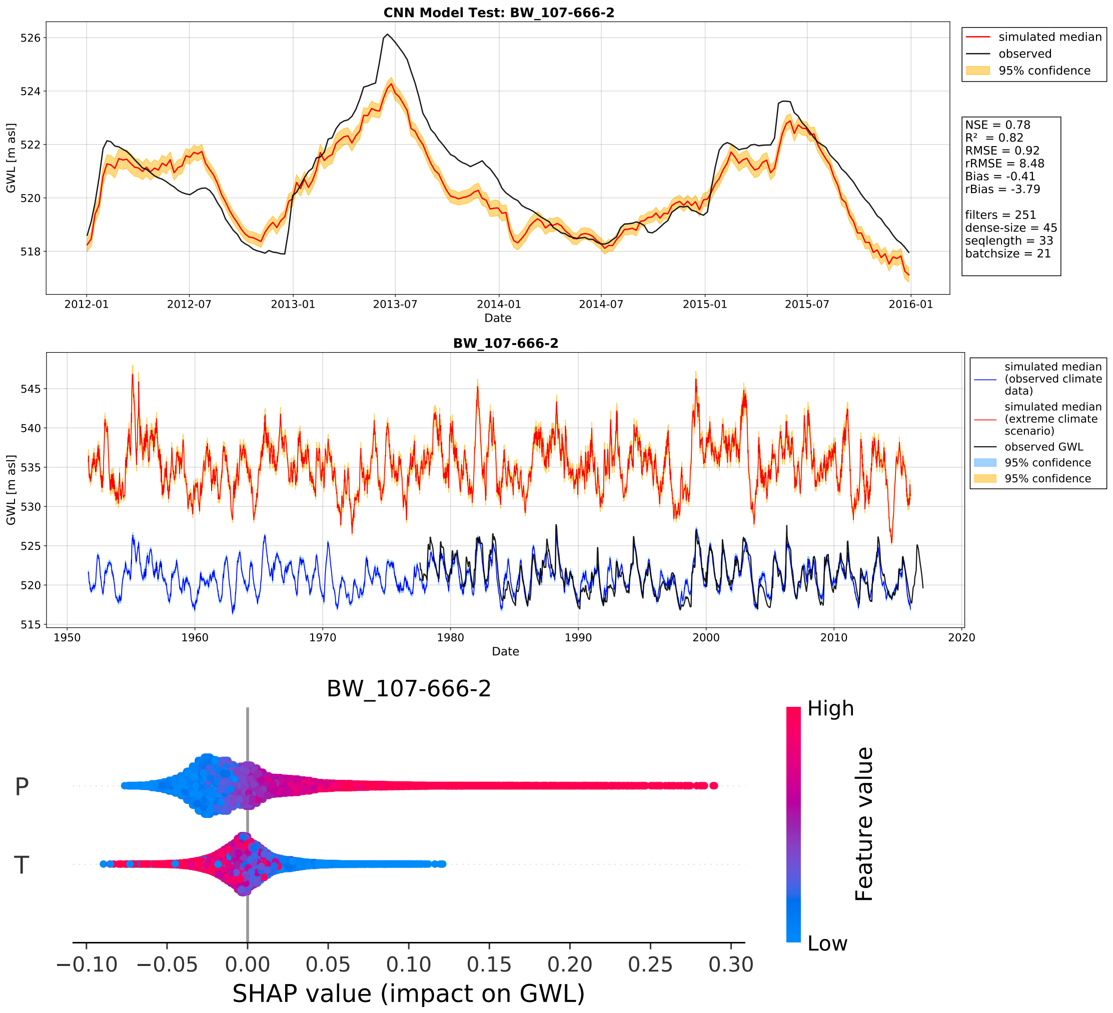

Figure S30: Evaluation of BW_107-666-2 Model Performance in the past (upper), under extreme climate conditions (middle) and SHAP Summary plot (lower)

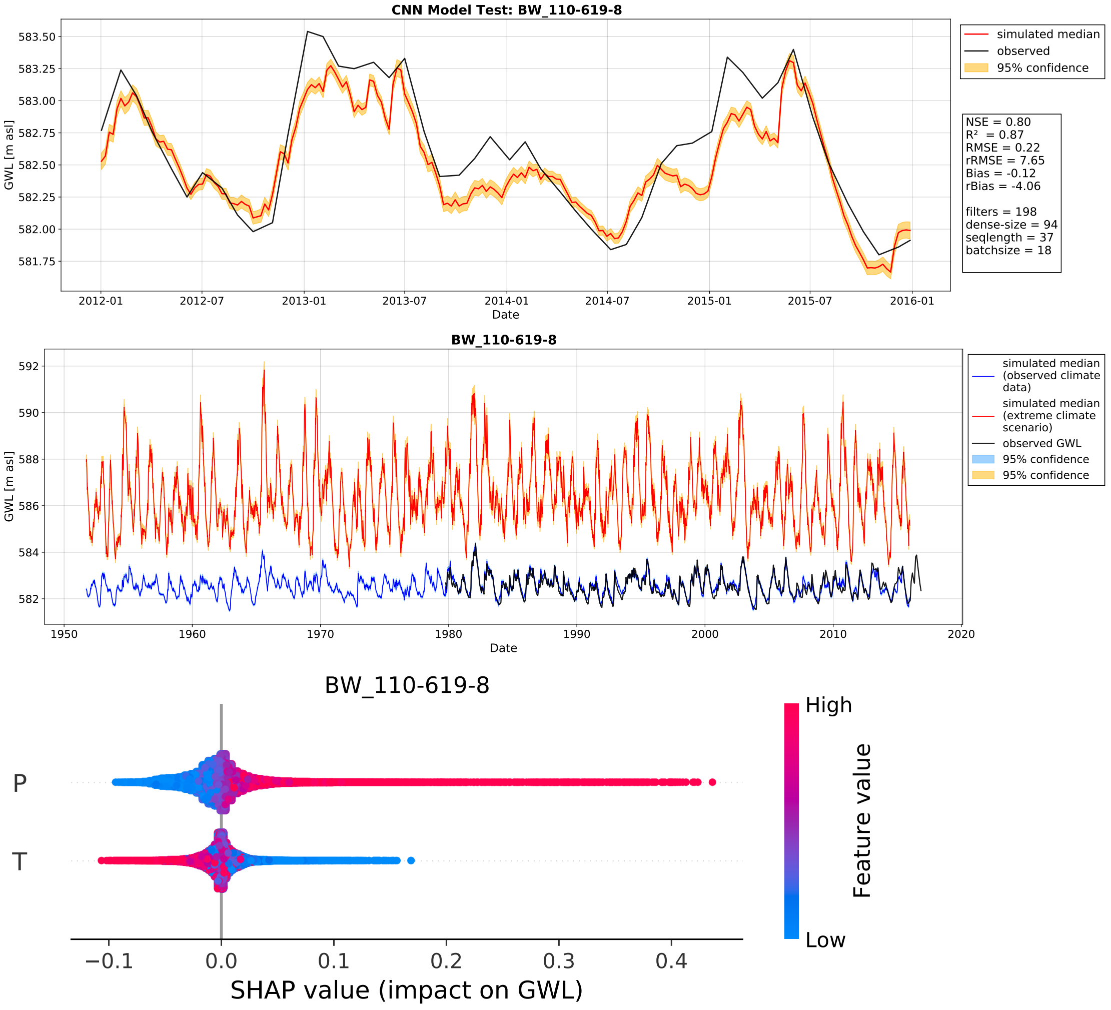

Figure S31: Evaluation of BW_110-619-8 Model Performance in the past (upper), under extreme climate conditions (middle) and SHAP Summary plot (lower)

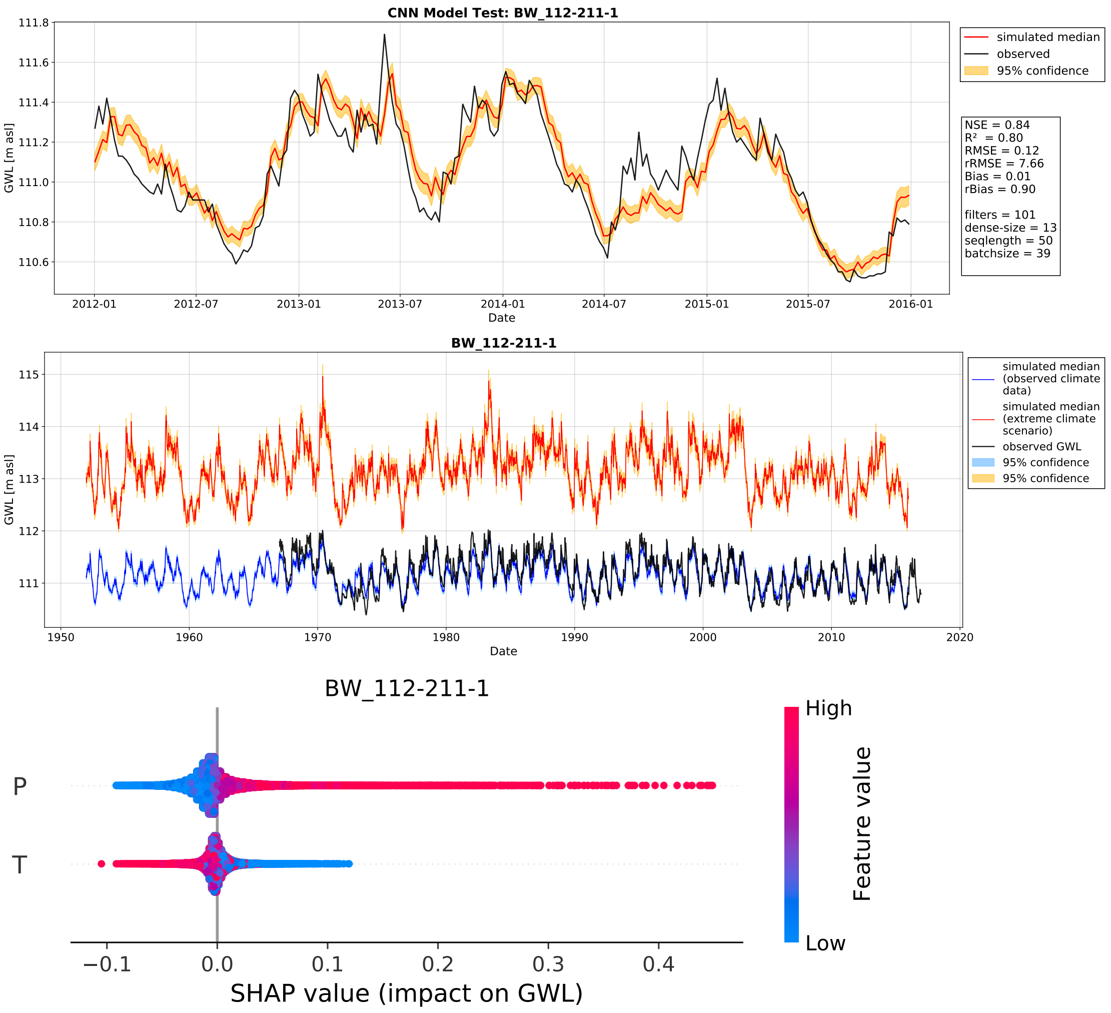

Figure S32: Evaluation of BW_112-211-1 Model Performance in the past (upper), under extreme climate conditions (middle) and SHAP Summary plot (lower)

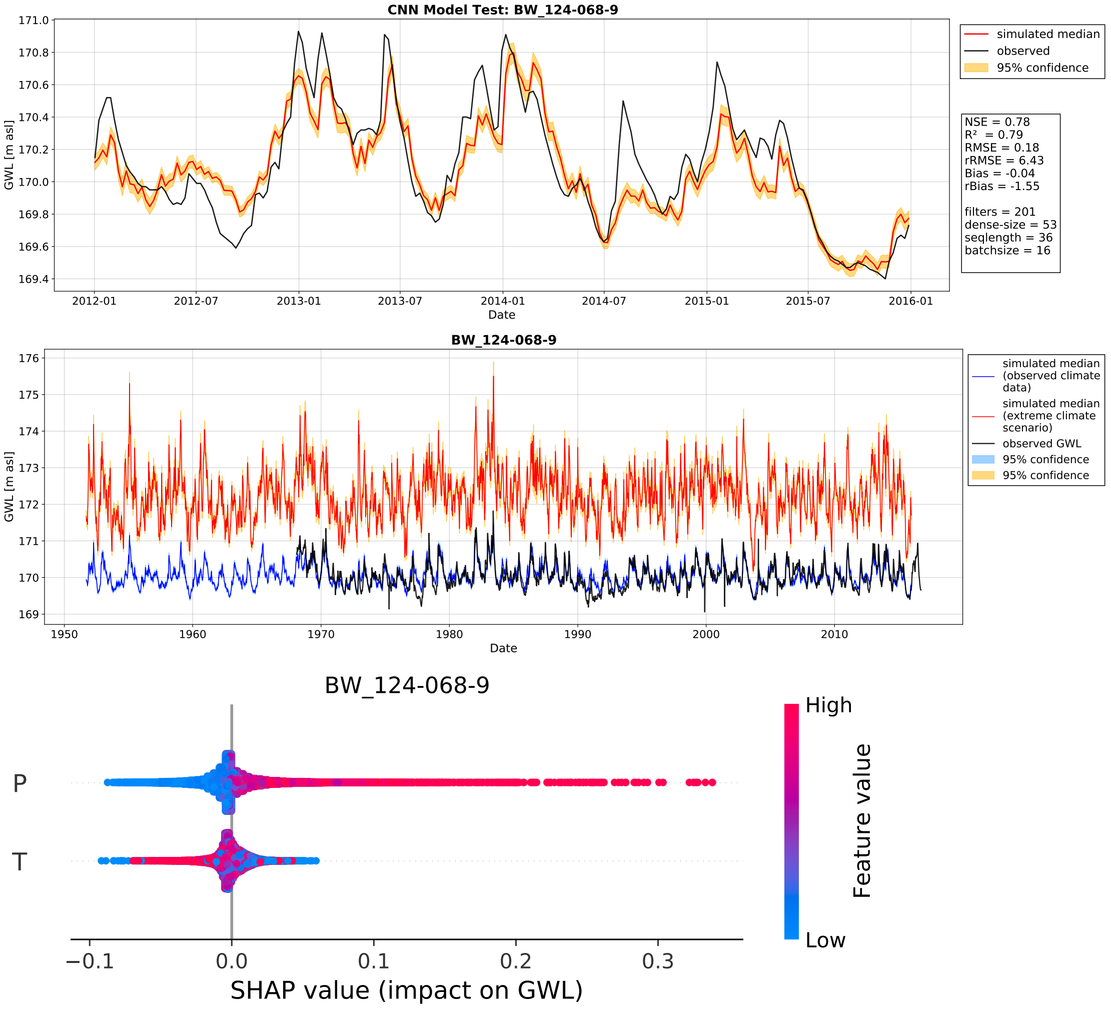

Figure S33: Evaluation of BW_124-068-9 Model Performance in the past (upper), under extreme climate conditions (middle) and SHAP Summary plot (lower)

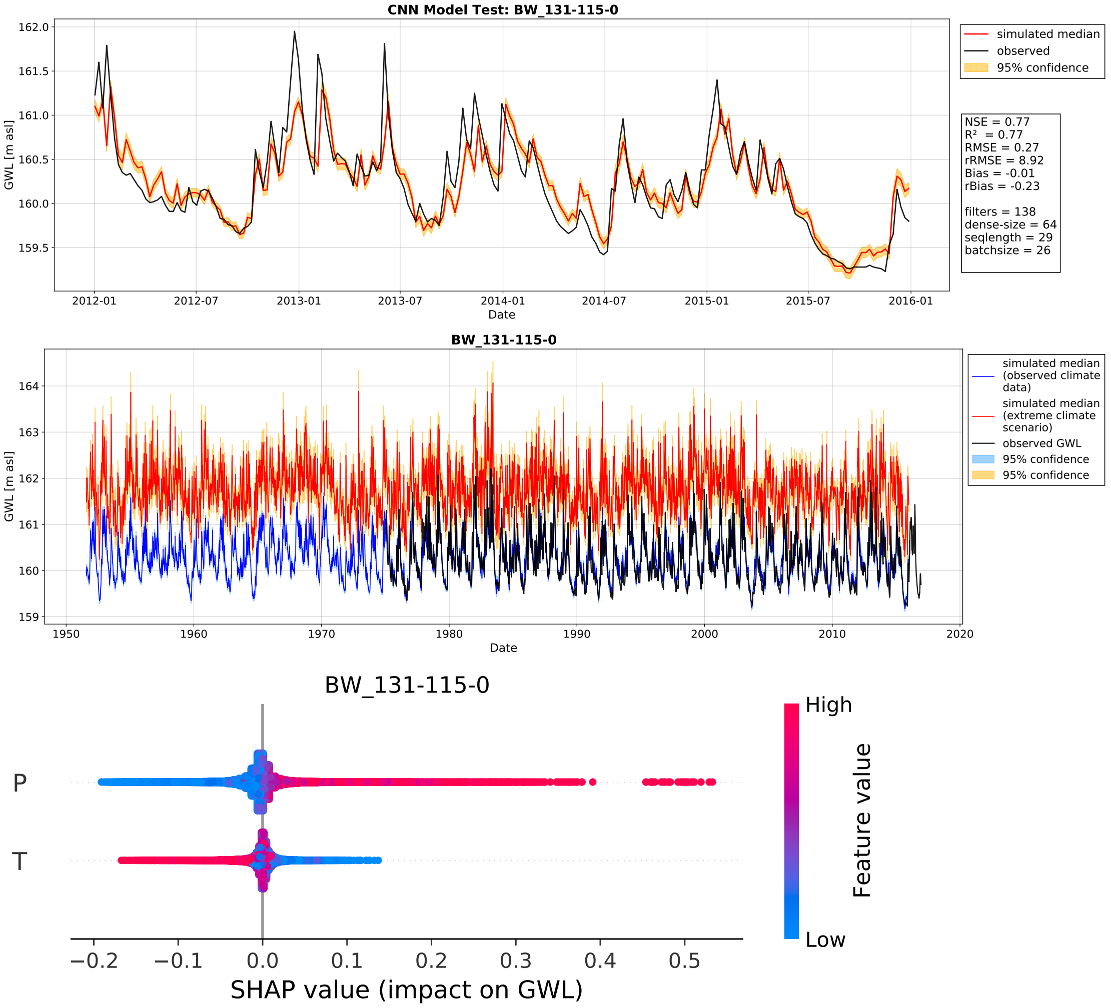

Figure S34: Evaluation of BW_131-115-0 Model Performance in the past (upper), under extreme climate conditions (middle) and SHAP Summary plot (lower)

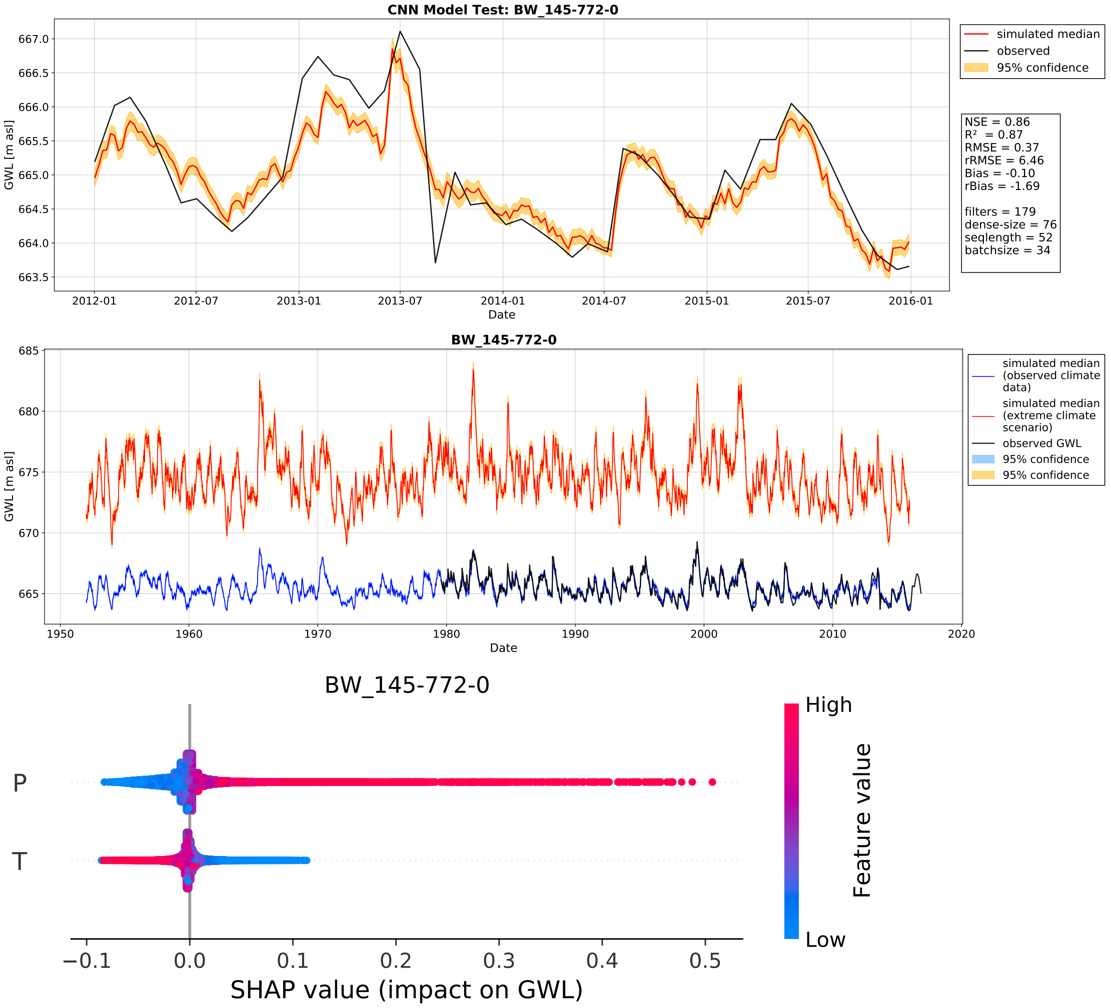

Figure S35: Evaluation of BW_145-772-0 Model Performance in the past (upper), under extreme climate conditions (middle) and SHAP Summary plot (lower)

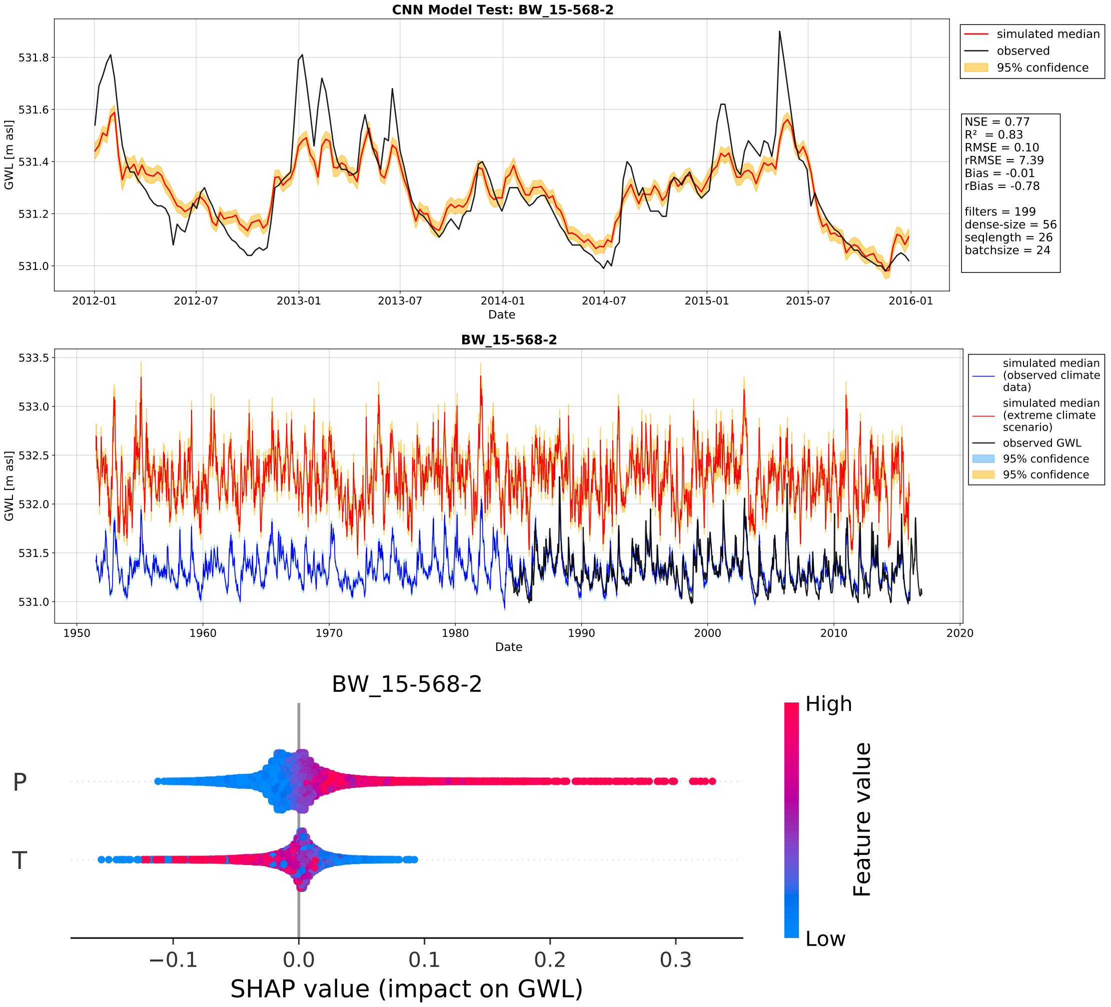

Figure S36: Evaluation of BW_15-568-2 Model Performance in the past (upper), under extreme climate conditions (middle) and SHAP Summary plot (lower)

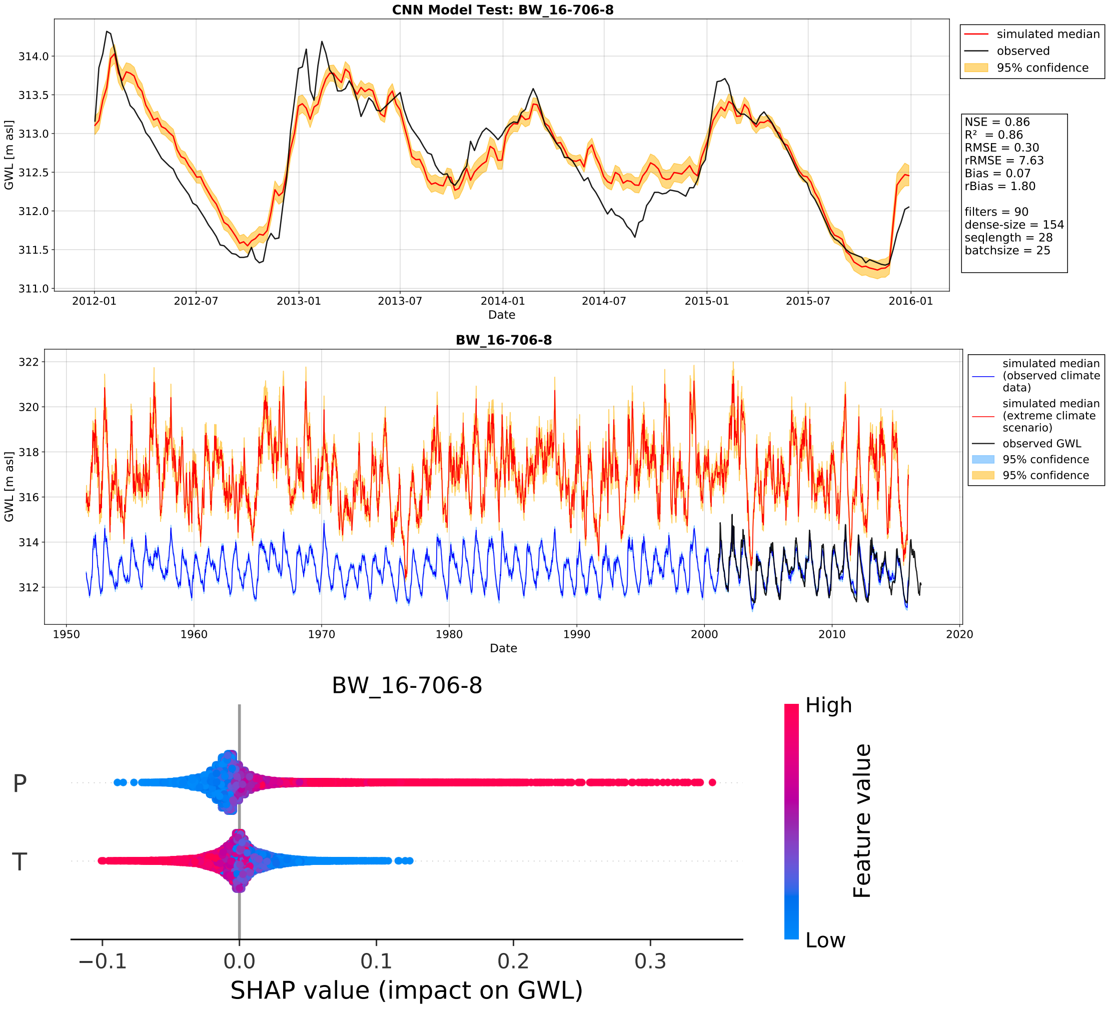

Figure S37: Evaluation of BW_16-706-8 Model Performance in the past (upper), under extreme climate conditions (middle) and SHAP Summary plot (lower)

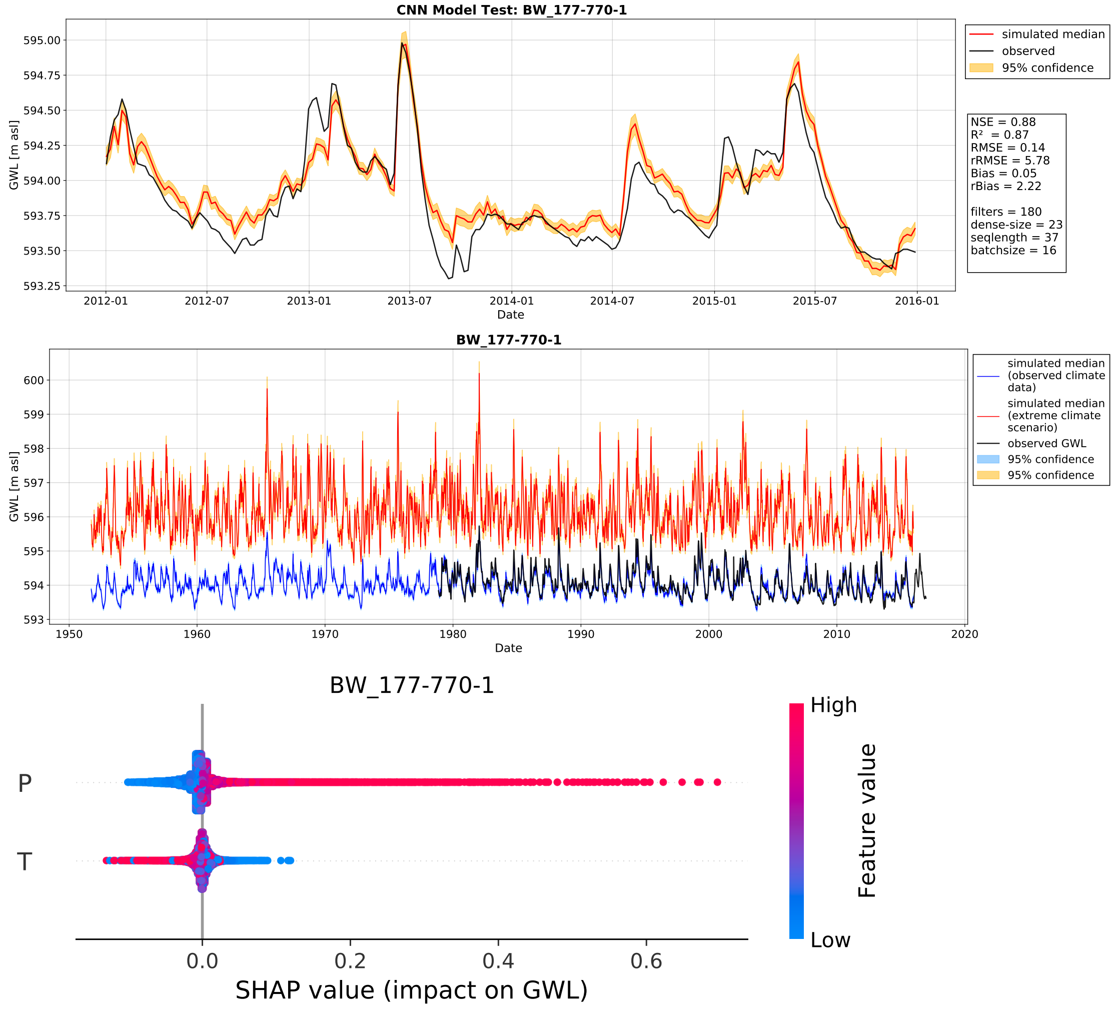

Figure S38: Evaluation of BW_177-770-1 Model Performance in the past (upper), under extreme climate conditions (middle) and SHAP Summary plot (lower)

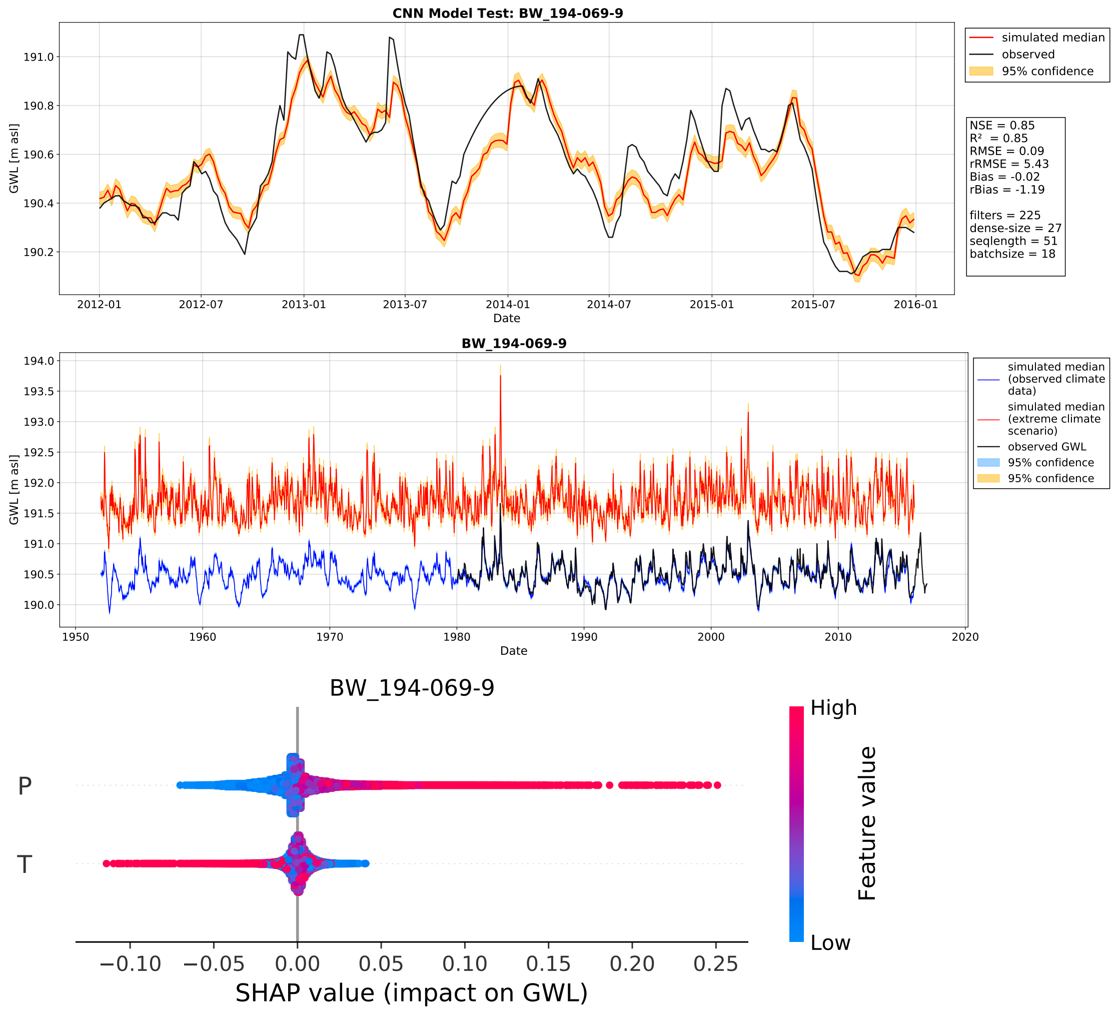

Figure S39: Evaluation of BW_194-069-9 Model Performance in the past (upper), under extreme climate conditions (middle) and SHAP Summary plot (lower)

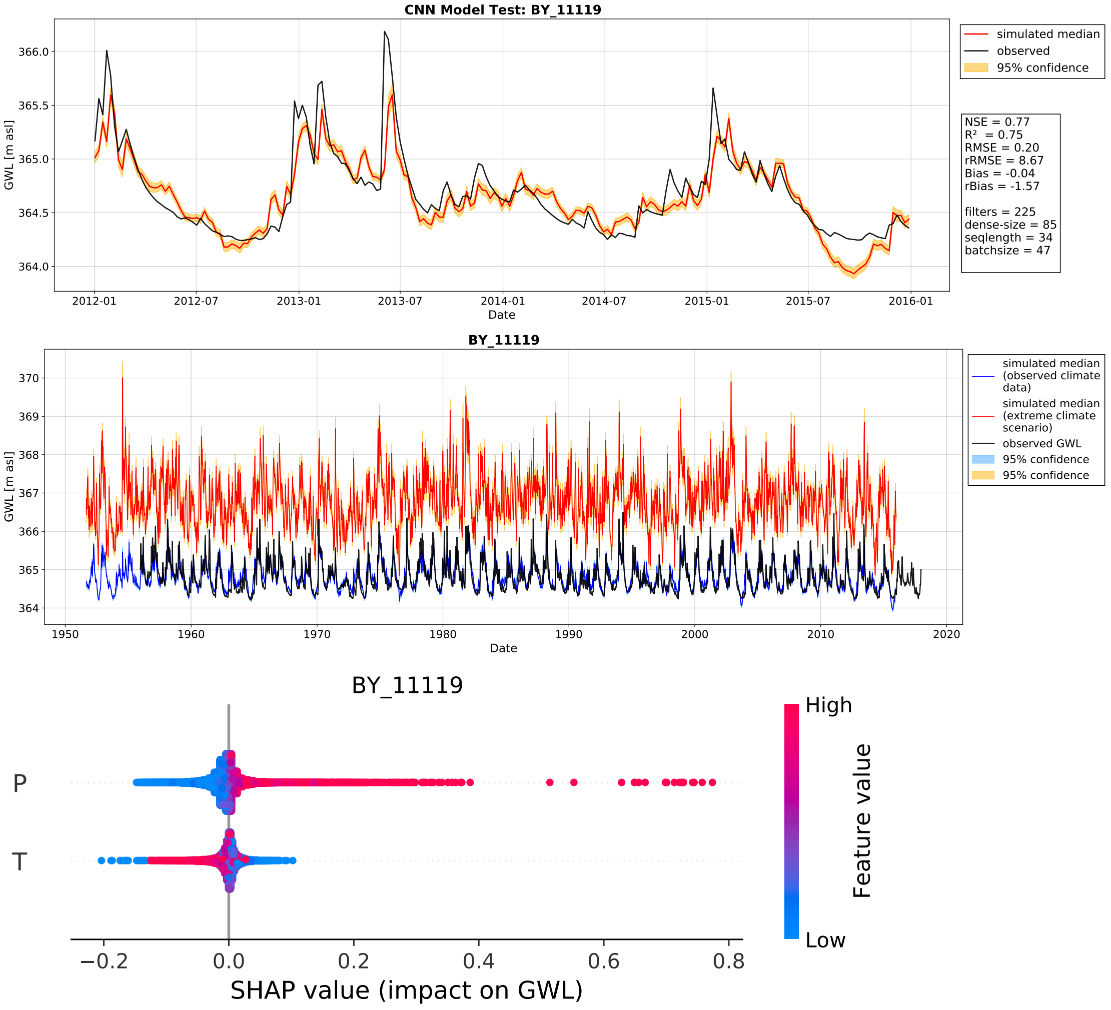

Figure S40: Evaluation of BY_11119 Model Performance in the past (upper), under extreme climate conditions (middle) and SHAP Summary plot (lower)

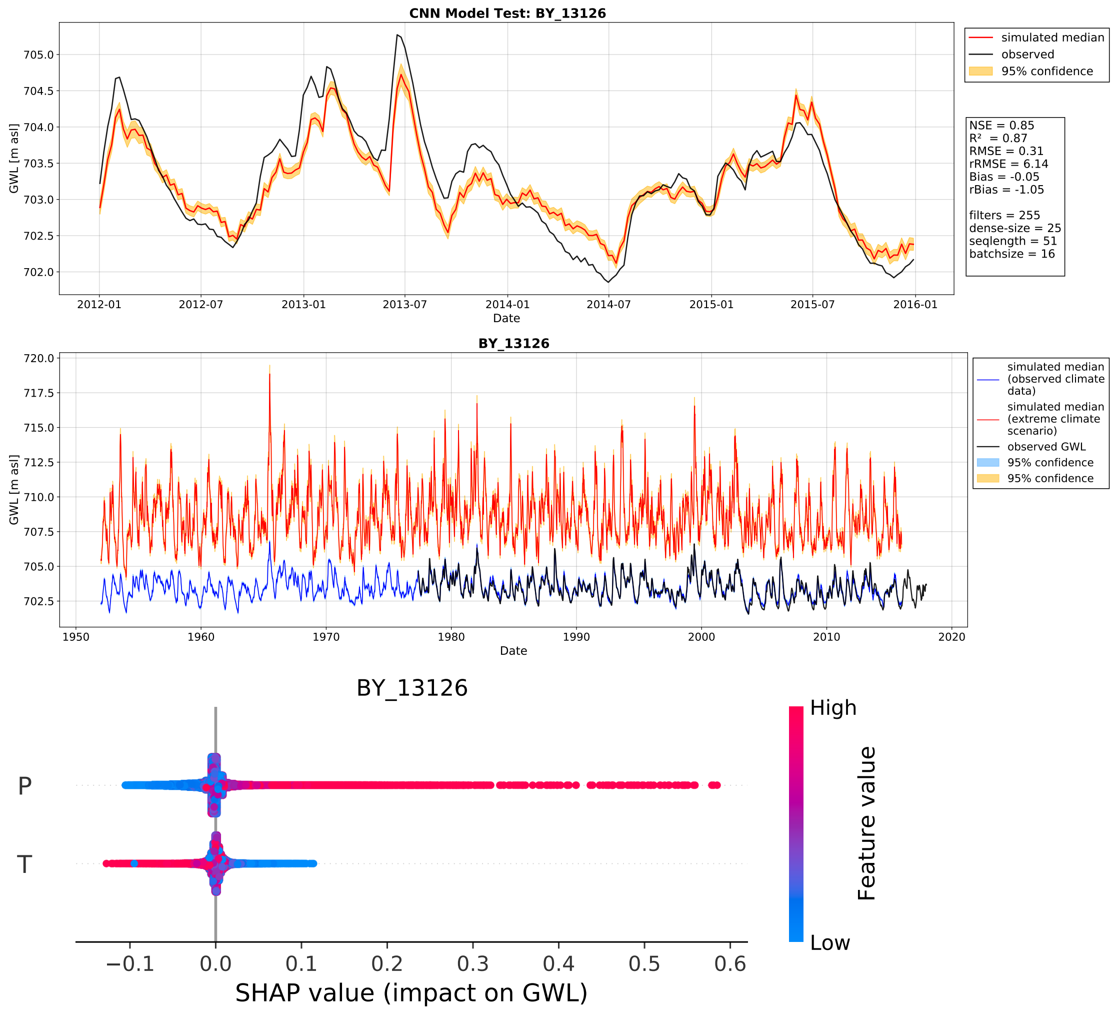

Figure S41: Evaluation of BY_13126 Model Performance in the past (upper), under extreme climate conditions (middle) and SHAP Summary plot (lower)

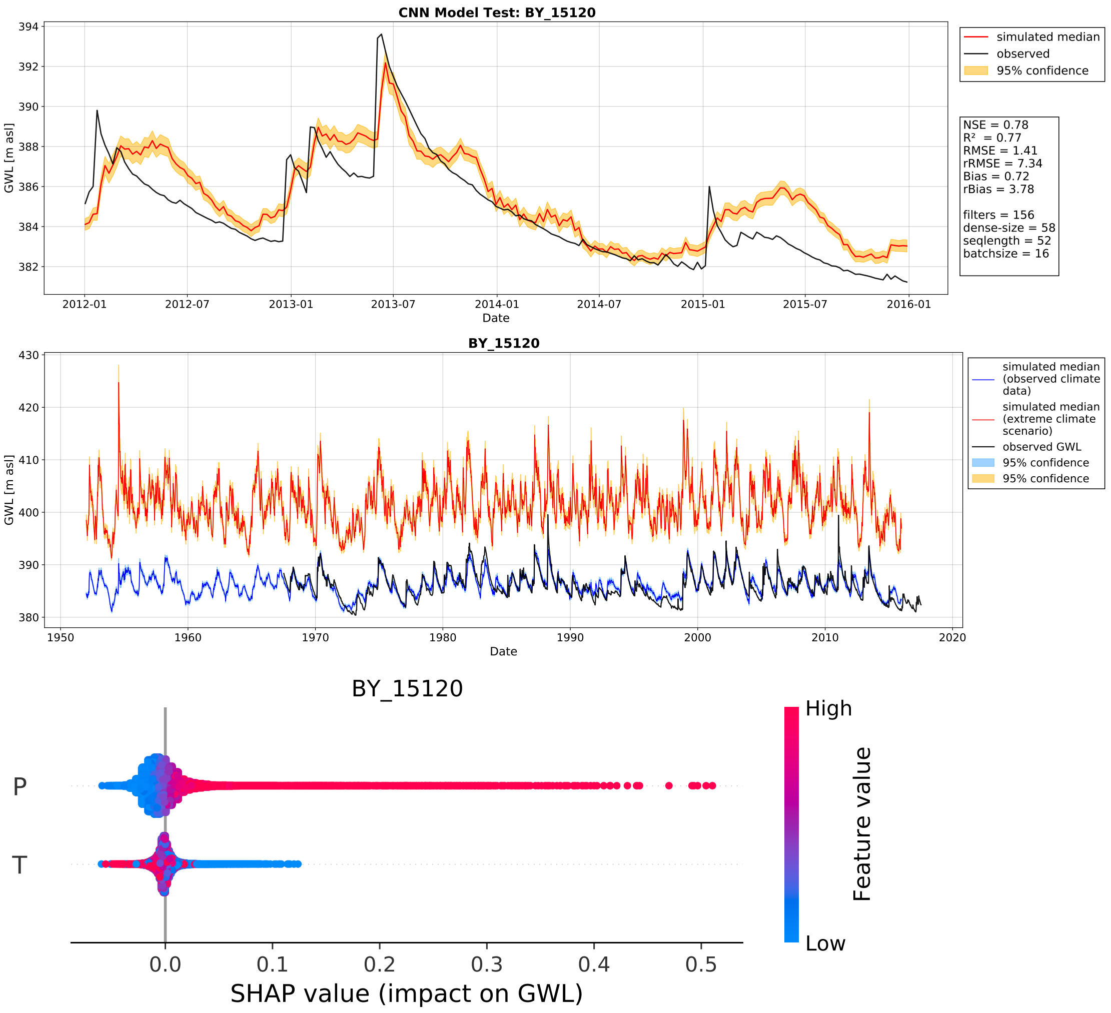

Figure S42: Evaluation of BY_15120 Model Performance in the past (upper), under extreme climate conditions (middle) and SHAP Summary plot (lower)

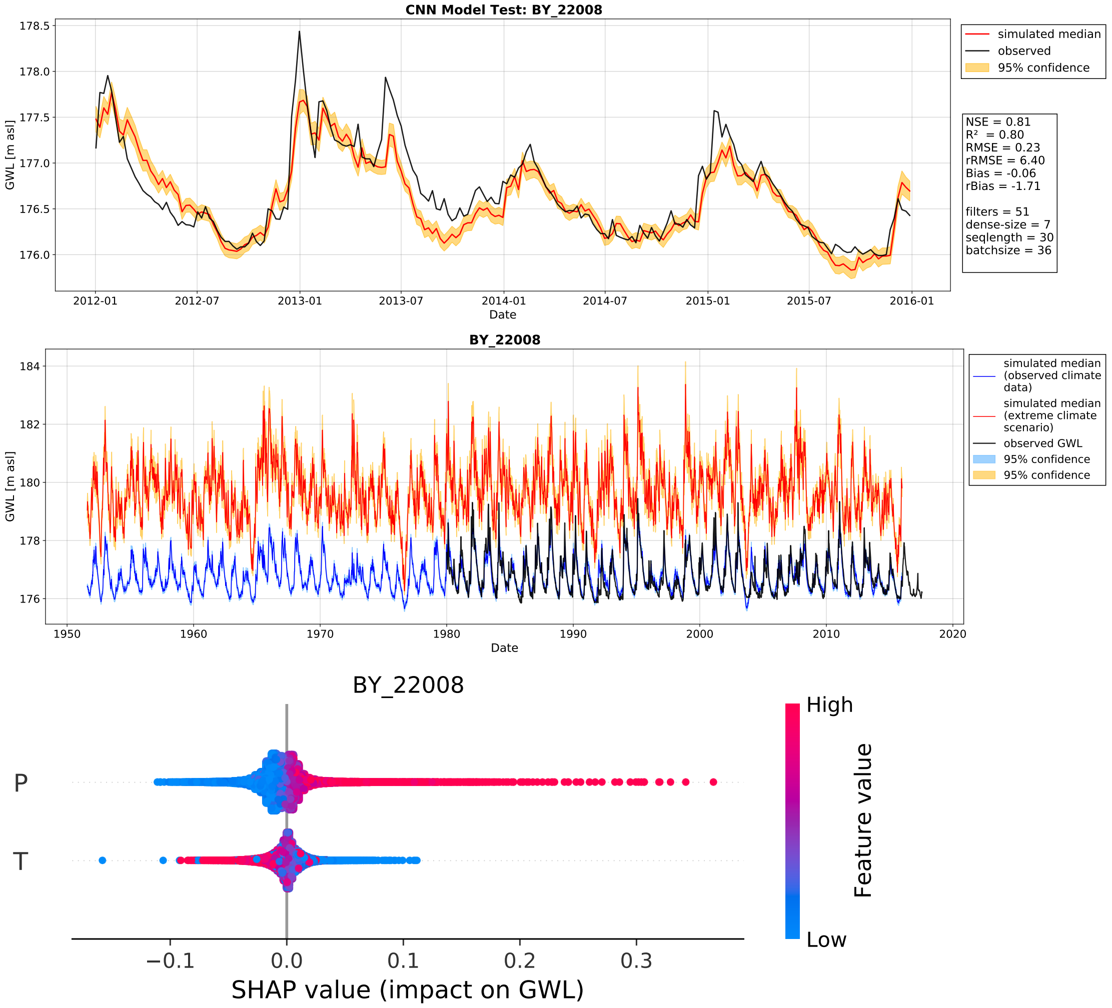

Figure S43: Evaluation of BY_22008 Model Performance in the past (upper), under extreme climate conditions (middle) and SHAP Summary plot (lower)

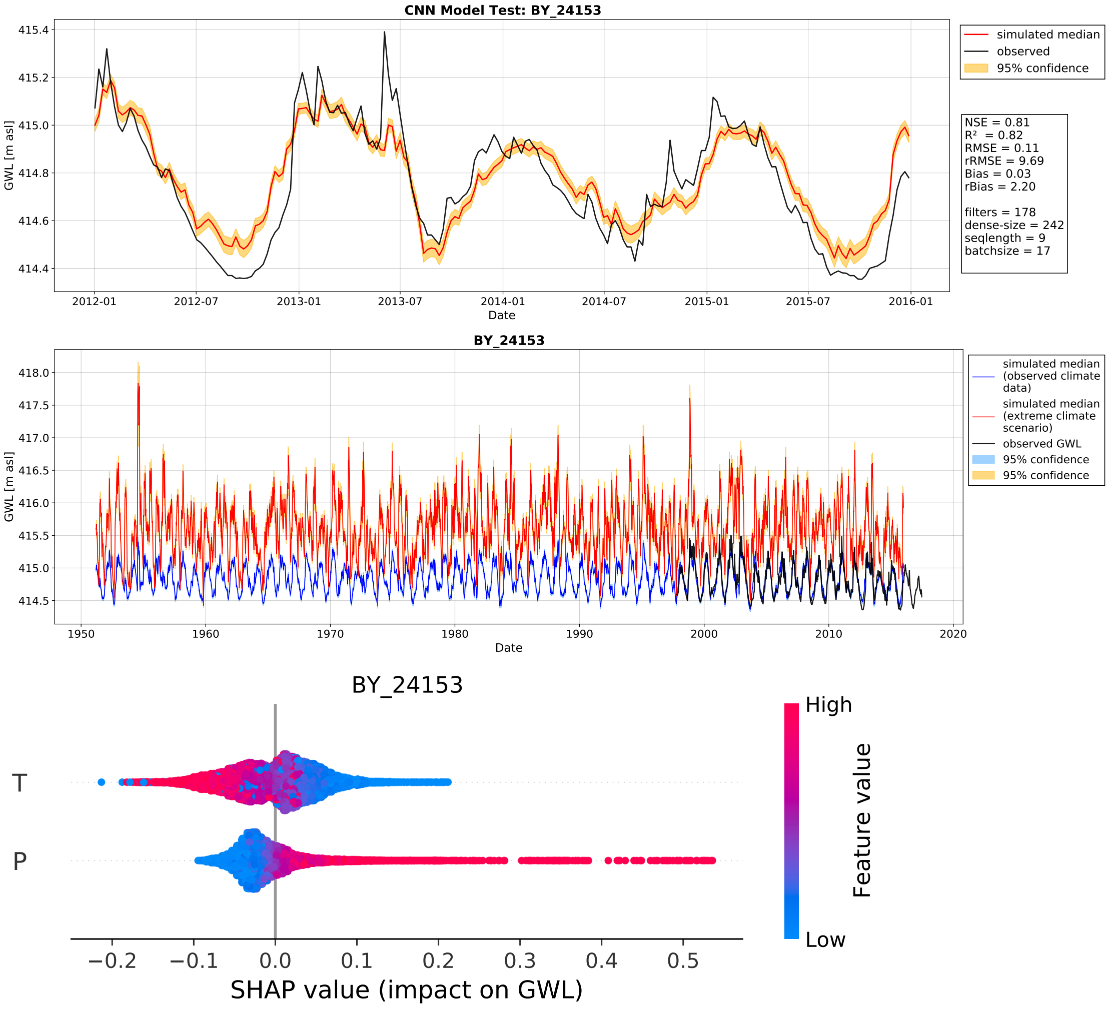

Figure S44: Evaluation of BY_24153 Model Performance in the past (upper), under extreme climate conditions (middle) and SHAP Summary plot (lower)

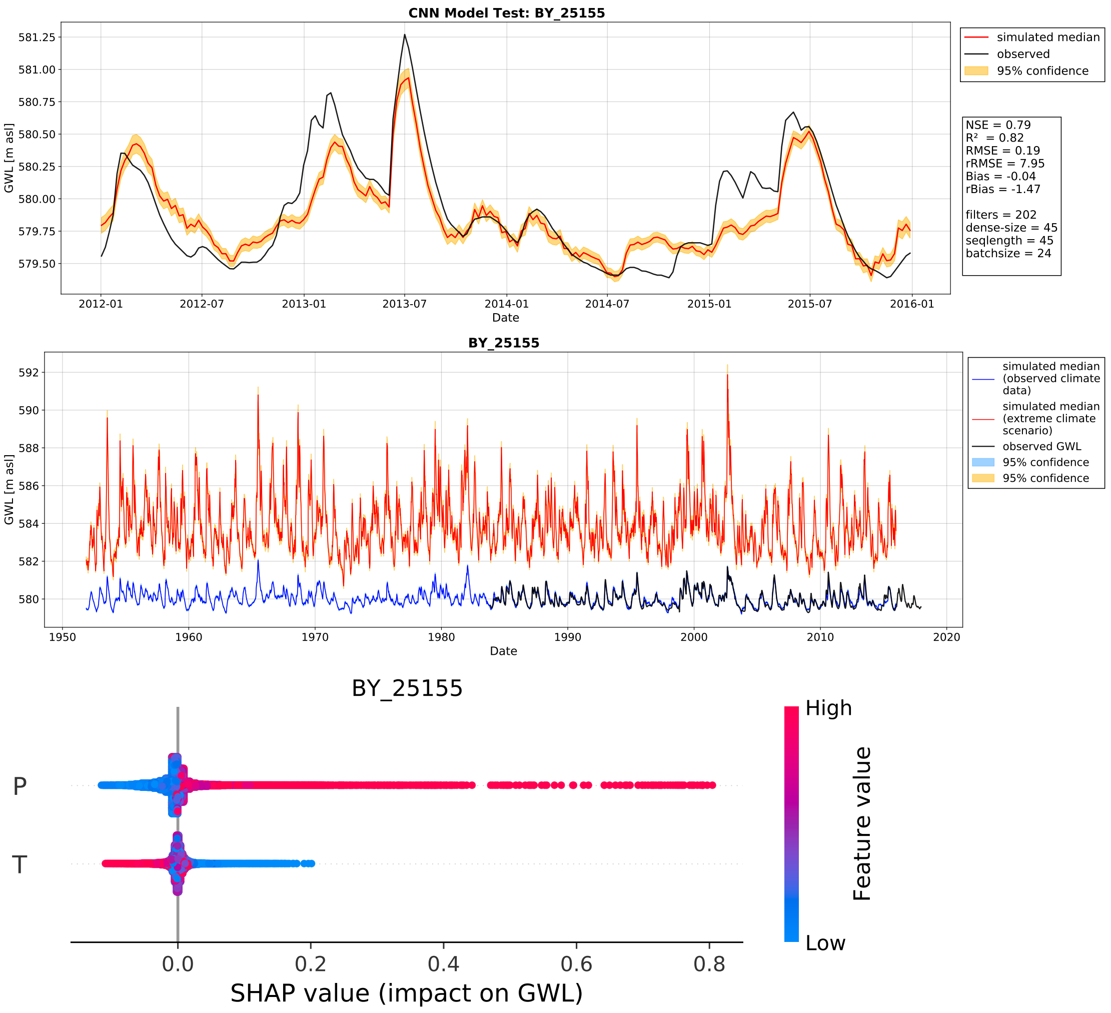

Figure S45: Evaluation of BY_25155 Model Performance in the past (upper), under extreme climate conditions (middle) and SHAP Summary plot (lower)

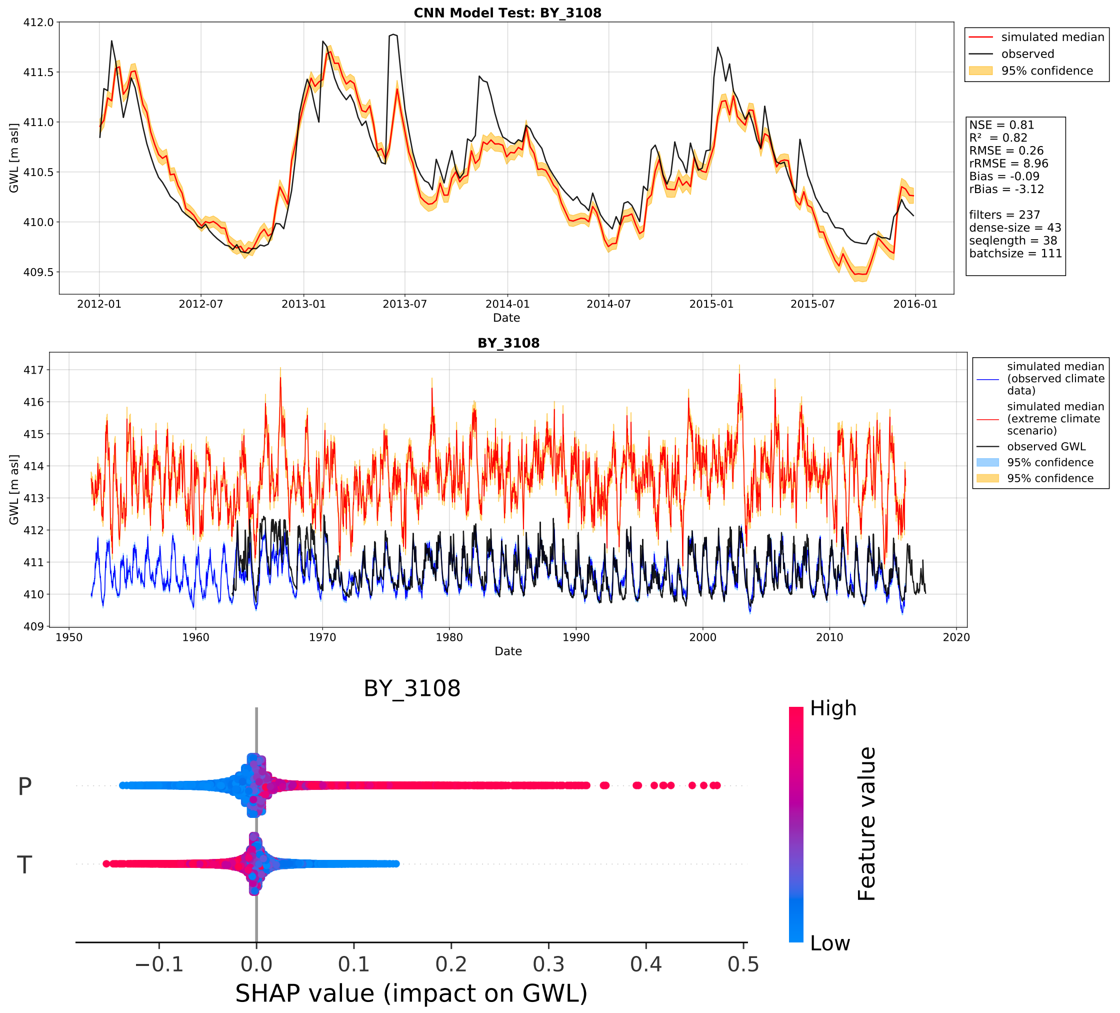

Figure S46: Evaluation of BY_3108 Model Performance in the past (upper), under extreme climate conditions (middle) and SHAP Summary plot (lower)

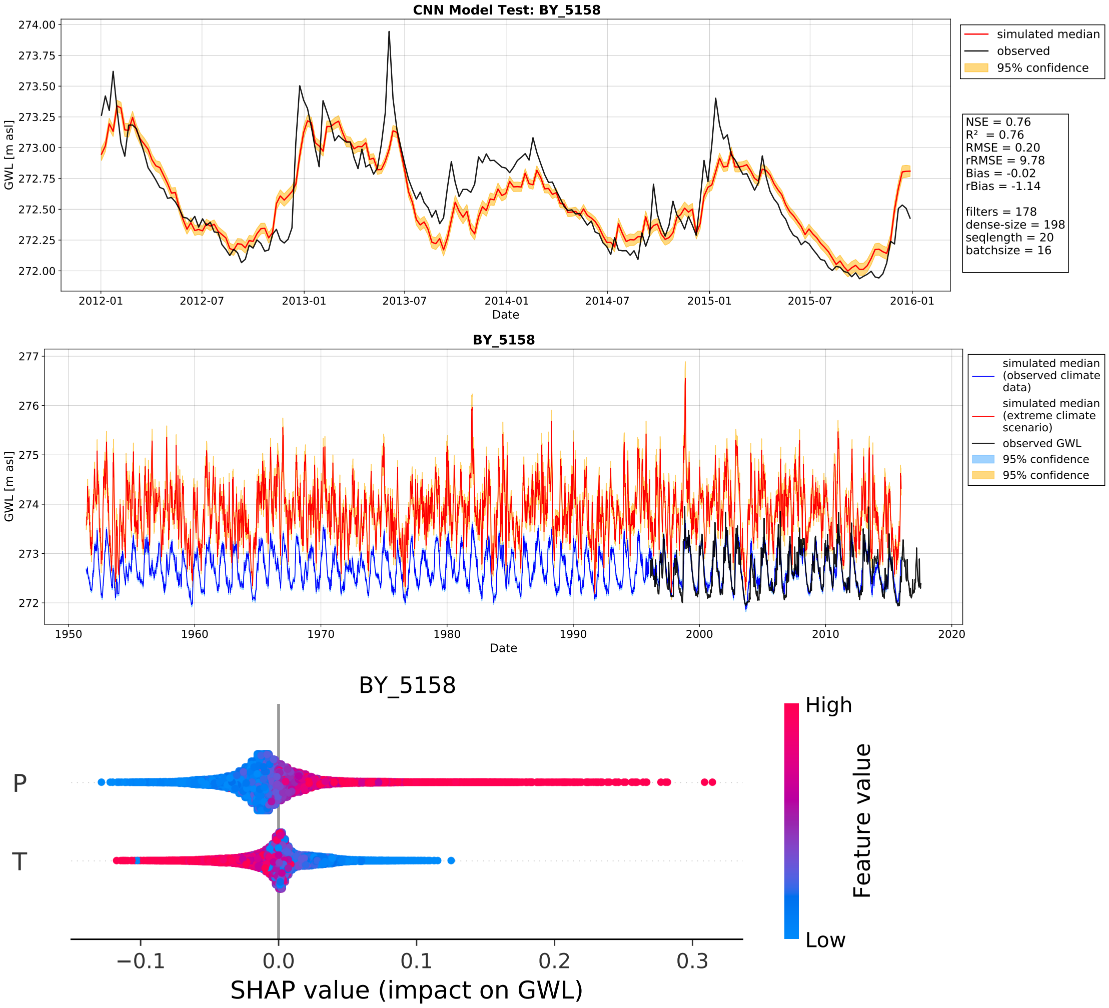

Figure S47: Evaluation of BY_5158 Model Performance in the past (upper), under extreme climate conditions (middle) and SHAP Summary plot (lower)

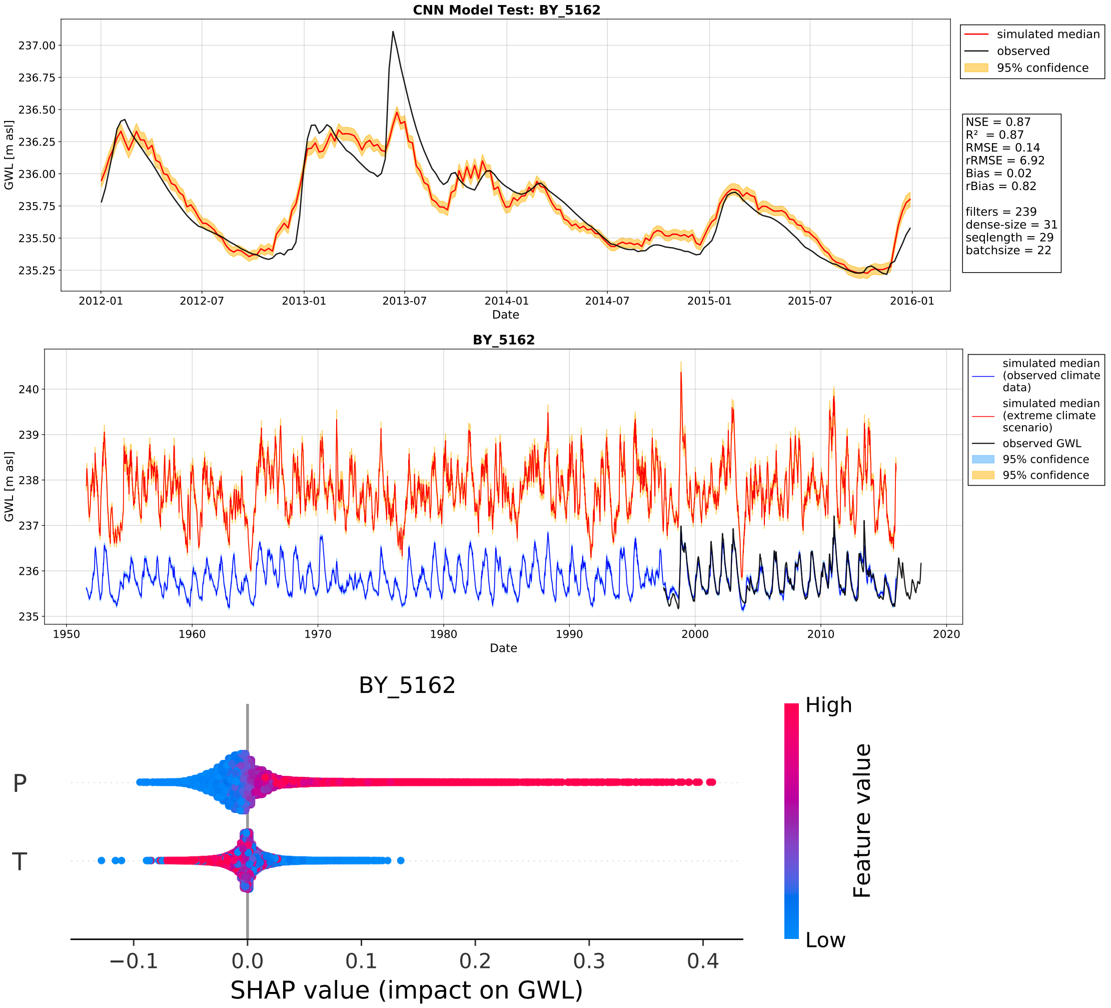

Figure S48: Evaluation of BY_5162 Model Performance in the past (upper), under extreme climate conditions (middle) and SHAP Summary plot (lower)

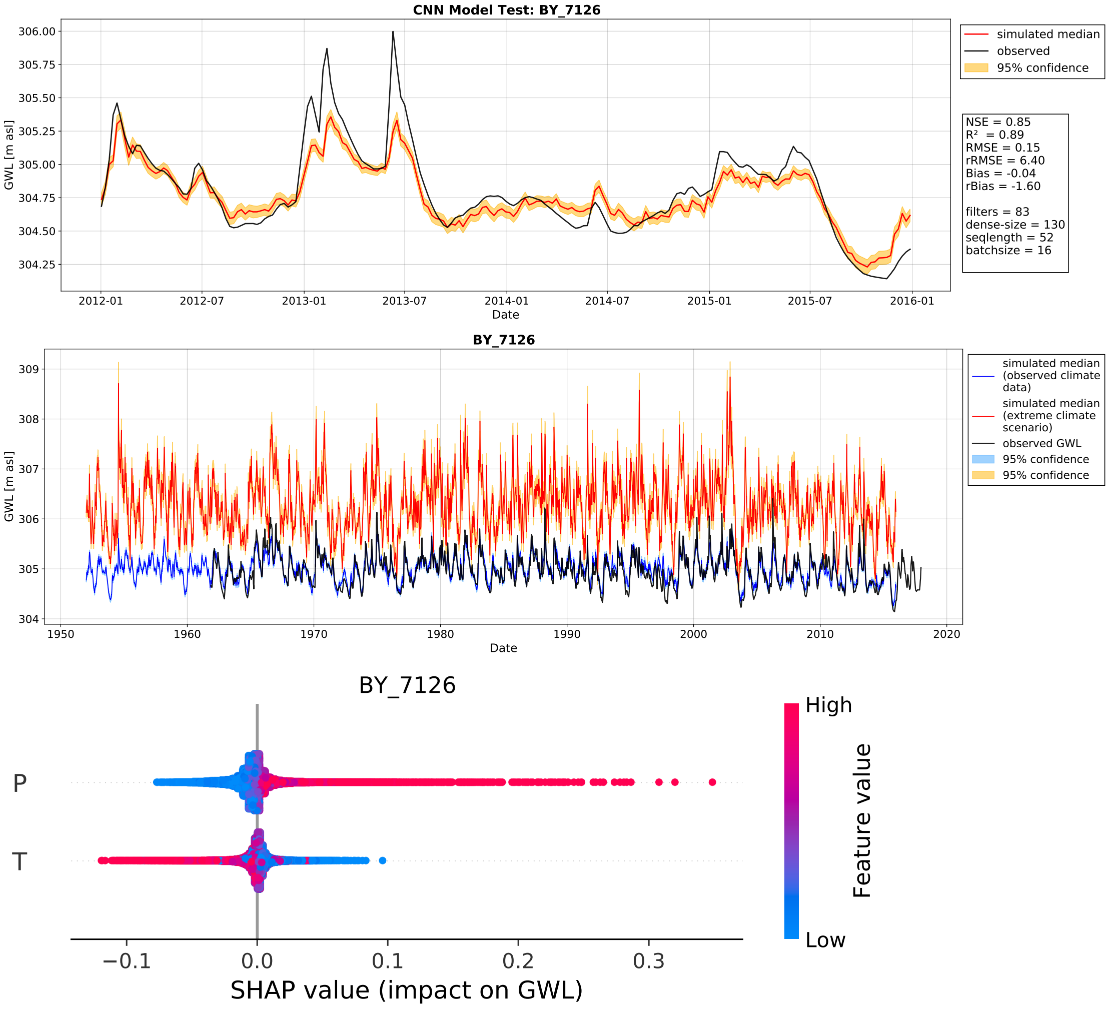

Figure S49: Evaluation of BY_7126 Model Performance in the past (upper), under extreme climate conditions (middle) and SHAP Summary plot (lower)

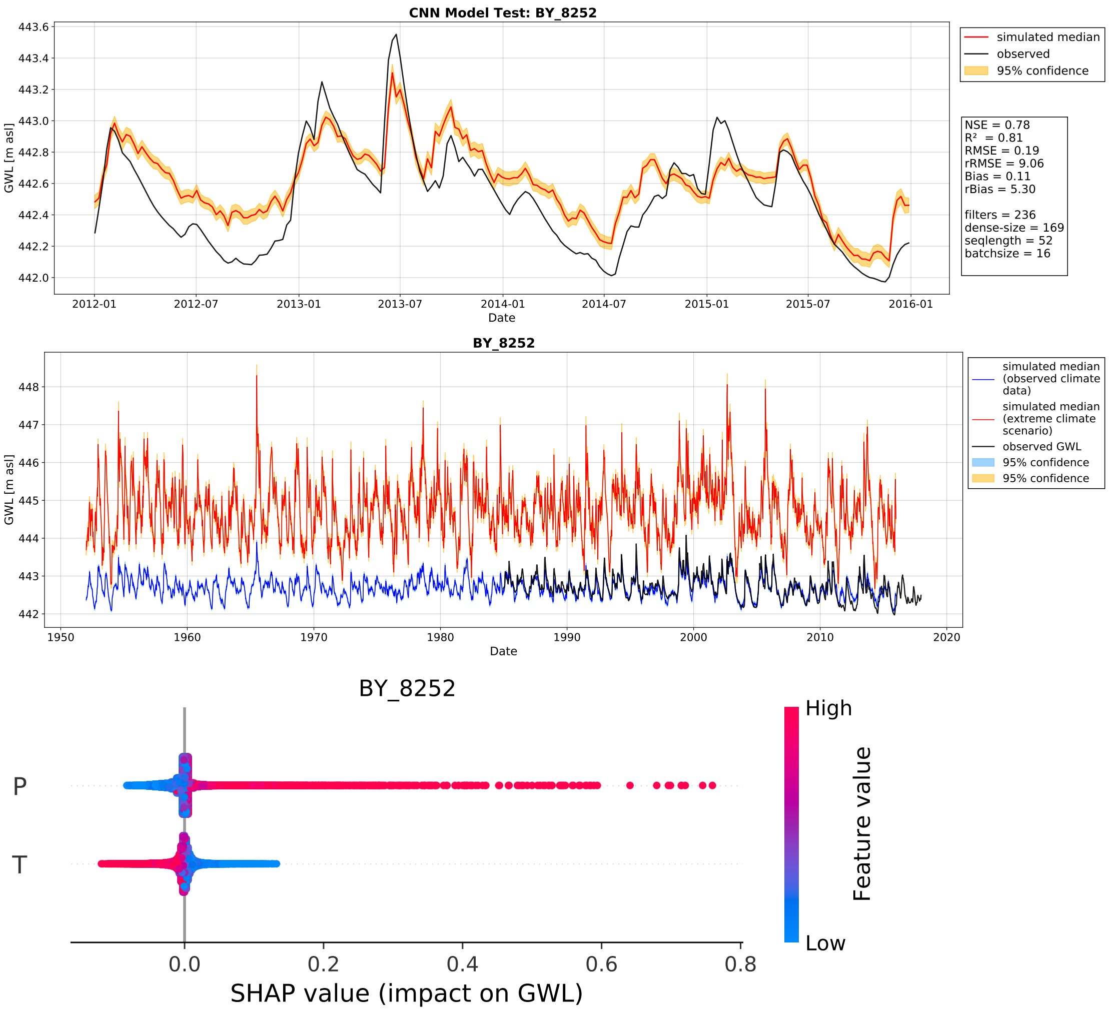

Figure S50: Evaluation of BY_8252 Model Performance in the past (upper), under extreme climate conditions (middle) and SHAP Summary plot (lower)

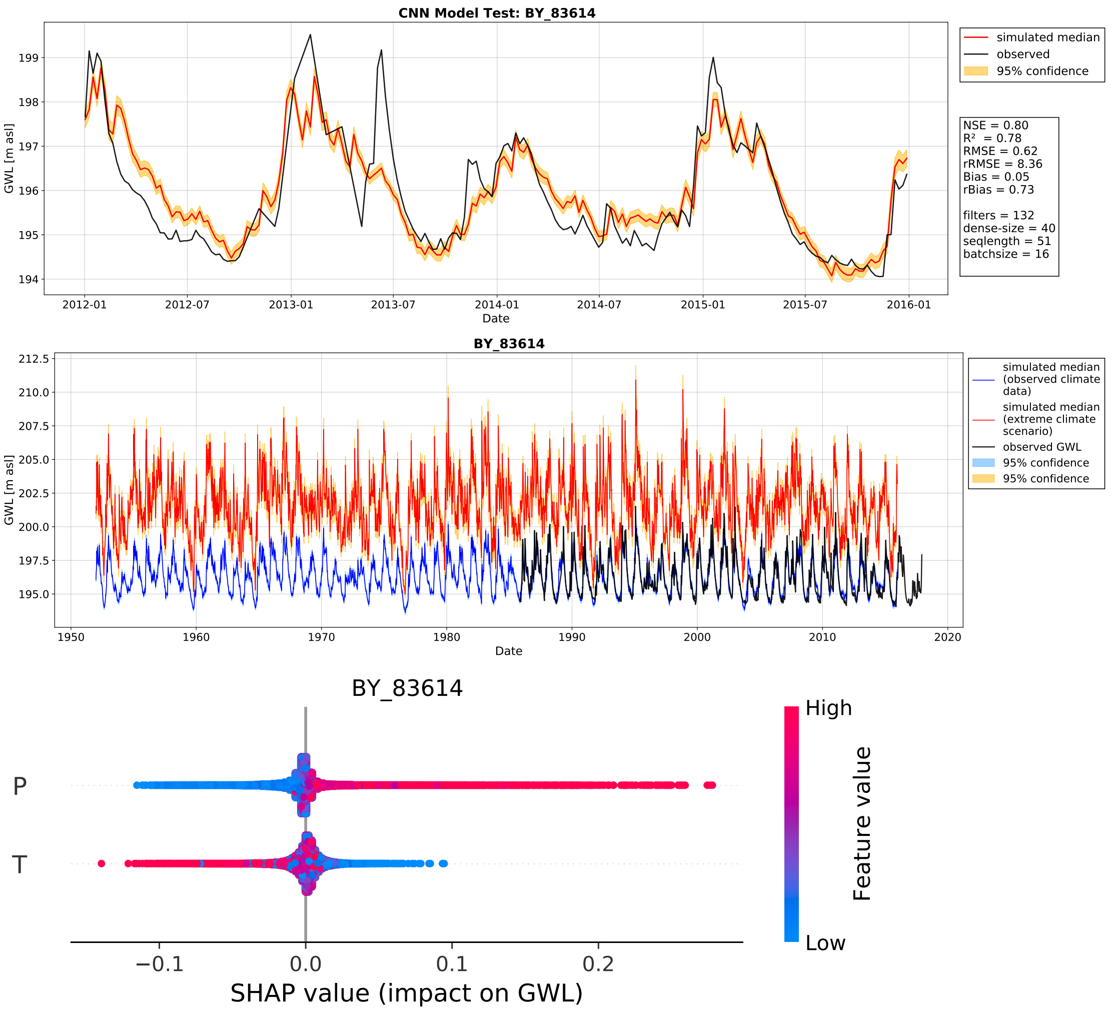

Figure S51: Evaluation of BY_83614 Model Performance in the past (upper), under extreme climate conditions (middle) and SHAP Summary plot (lower)

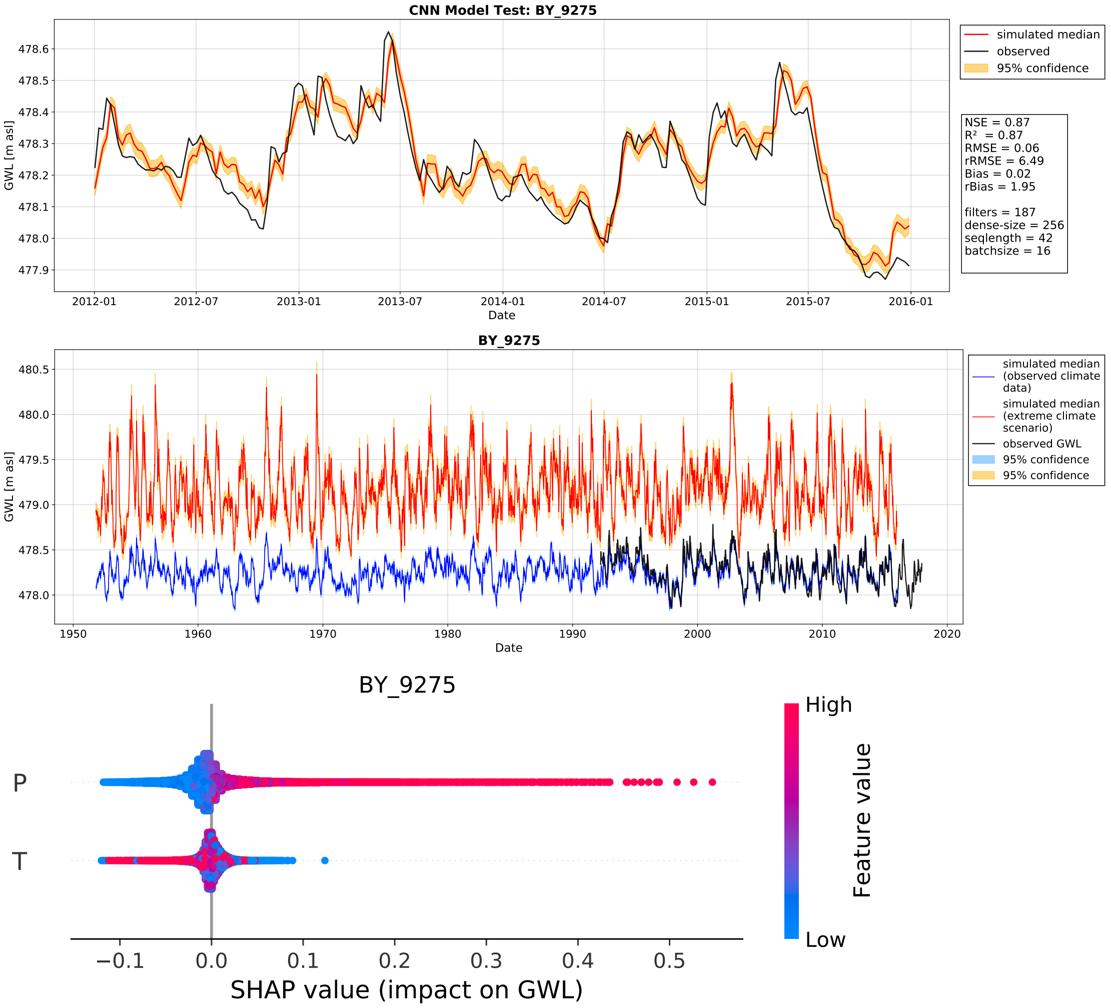

Figure S52: Evaluation of BY_9275 Model Performance in the past (upper), under extreme climate conditions (middle) and SHAP Summary plot (lower)

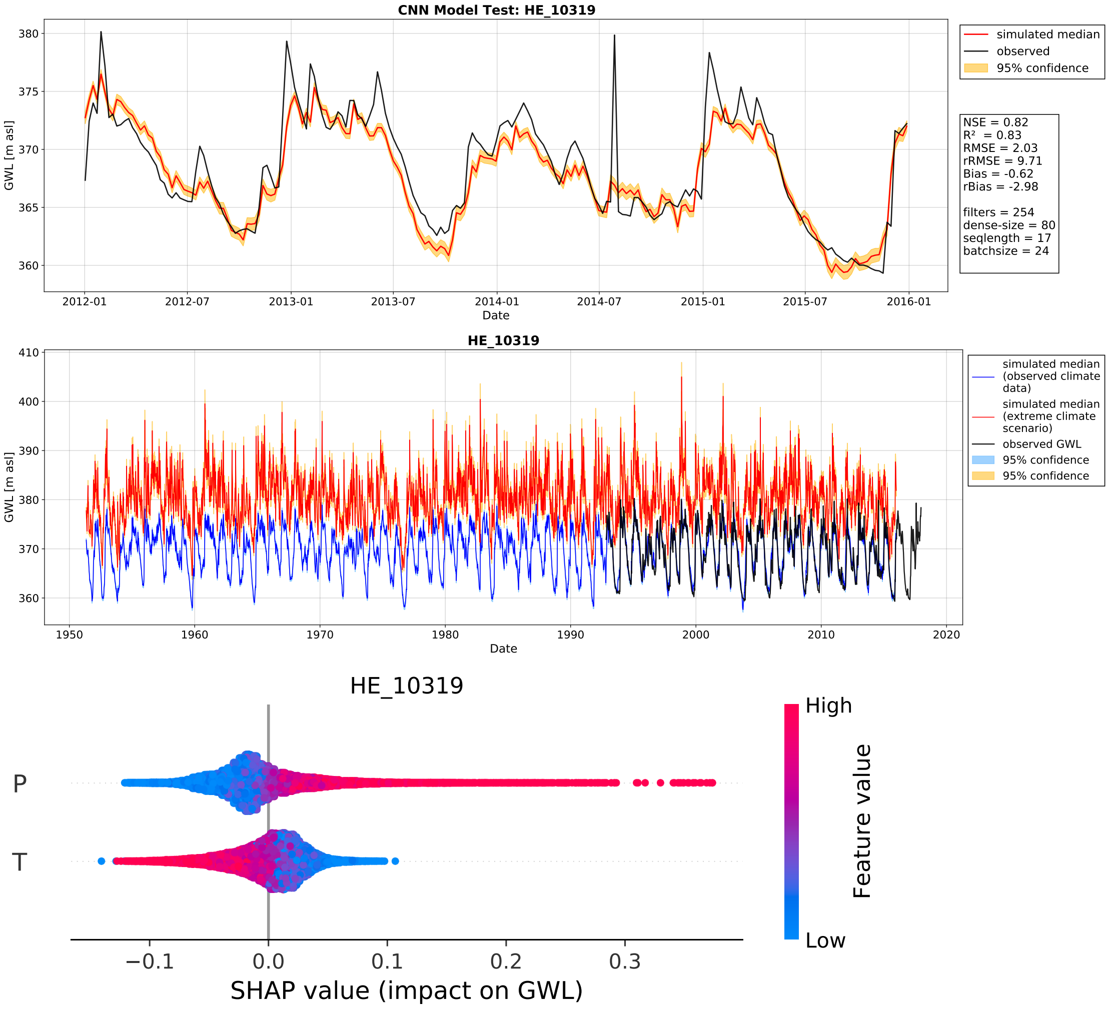

Figure S53: Evaluation of HE_10319 Model Performance in the past (upper), under extreme climate conditions (middle) and SHAP Summary plot (lower)

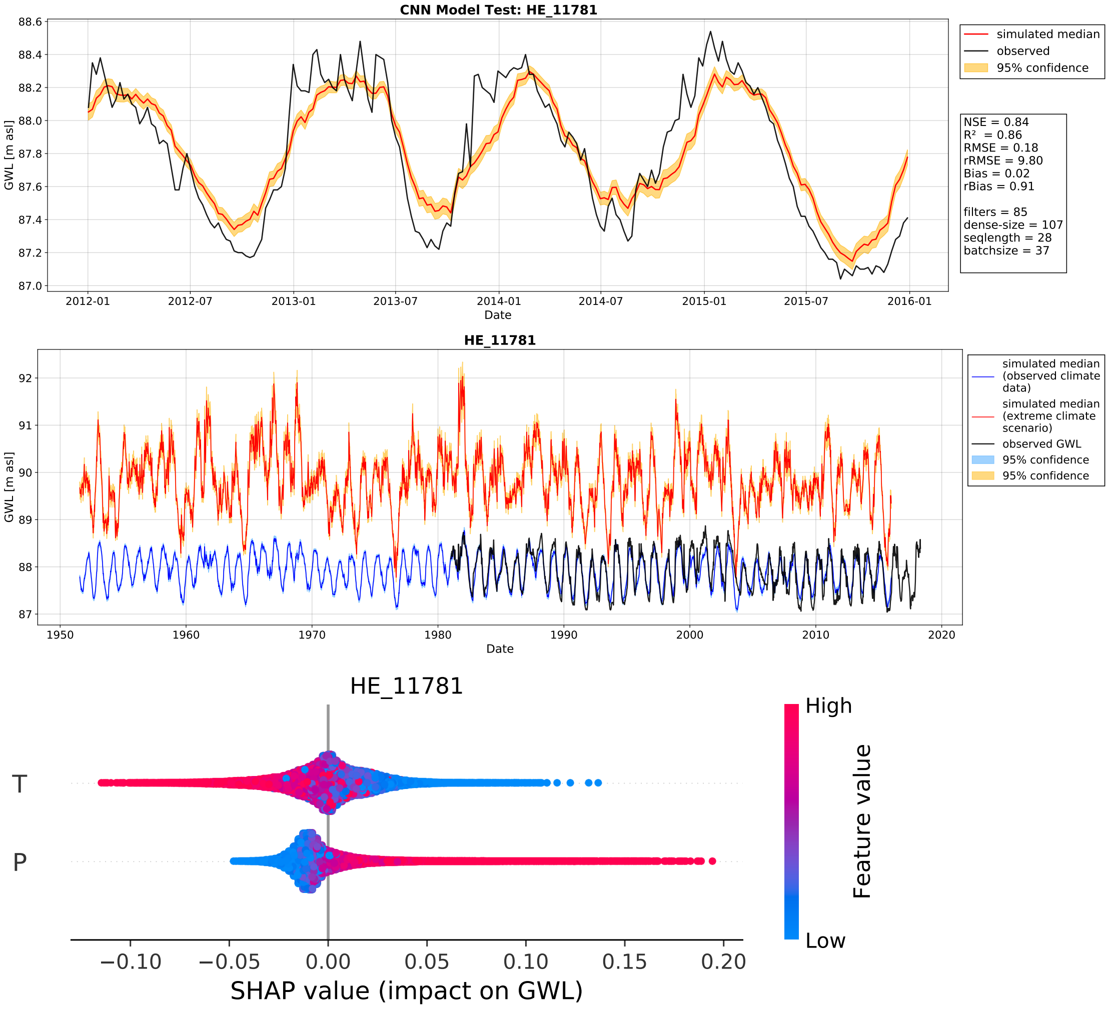

Figure S54: Evaluation of HE_11781 Model Performance in the past (upper), under extreme climate conditions (middle) and SHAP Summary plot (lower)

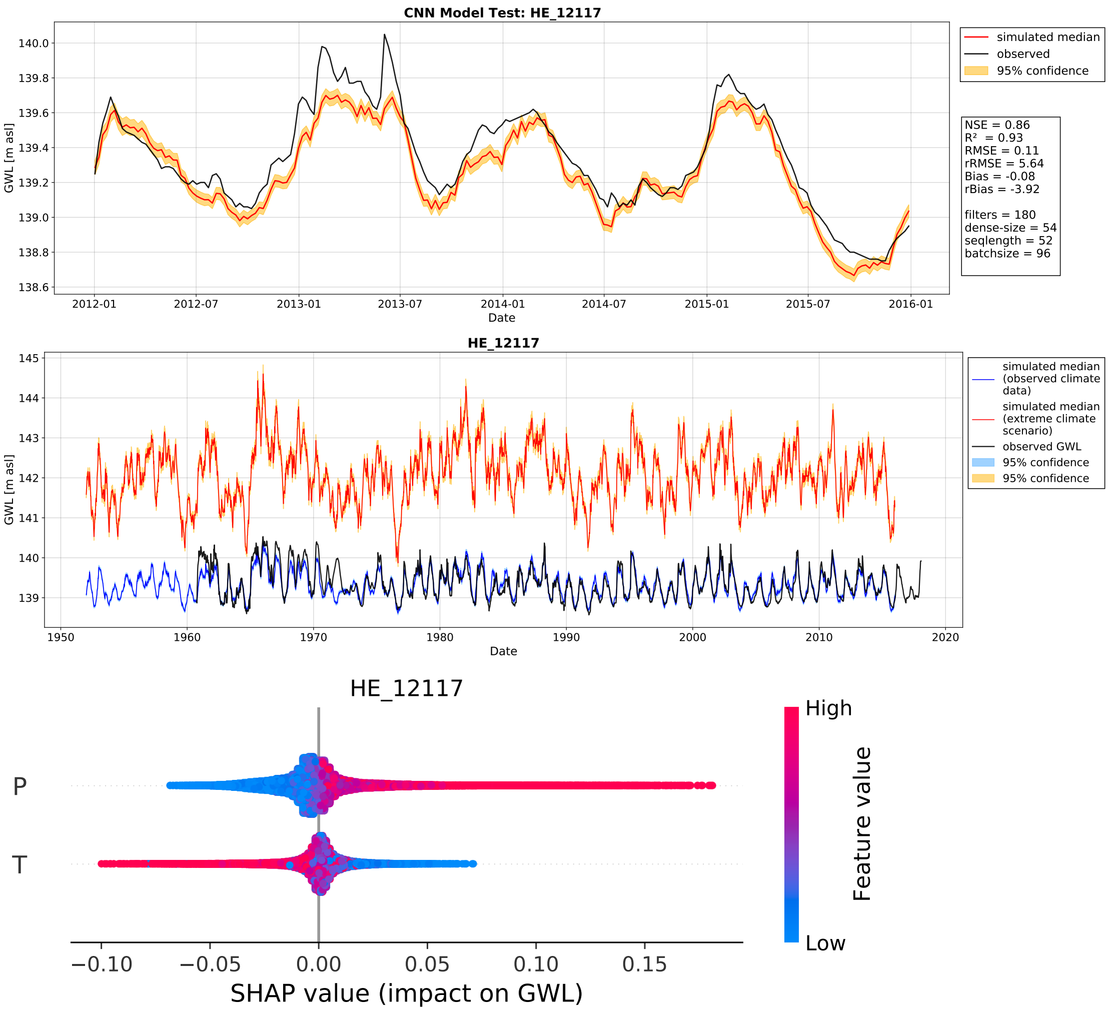

Figure S55: Evaluation of HE_12117 Model Performance in the past (upper), under extreme climate conditions (middle) and SHAP Summary plot (lower)

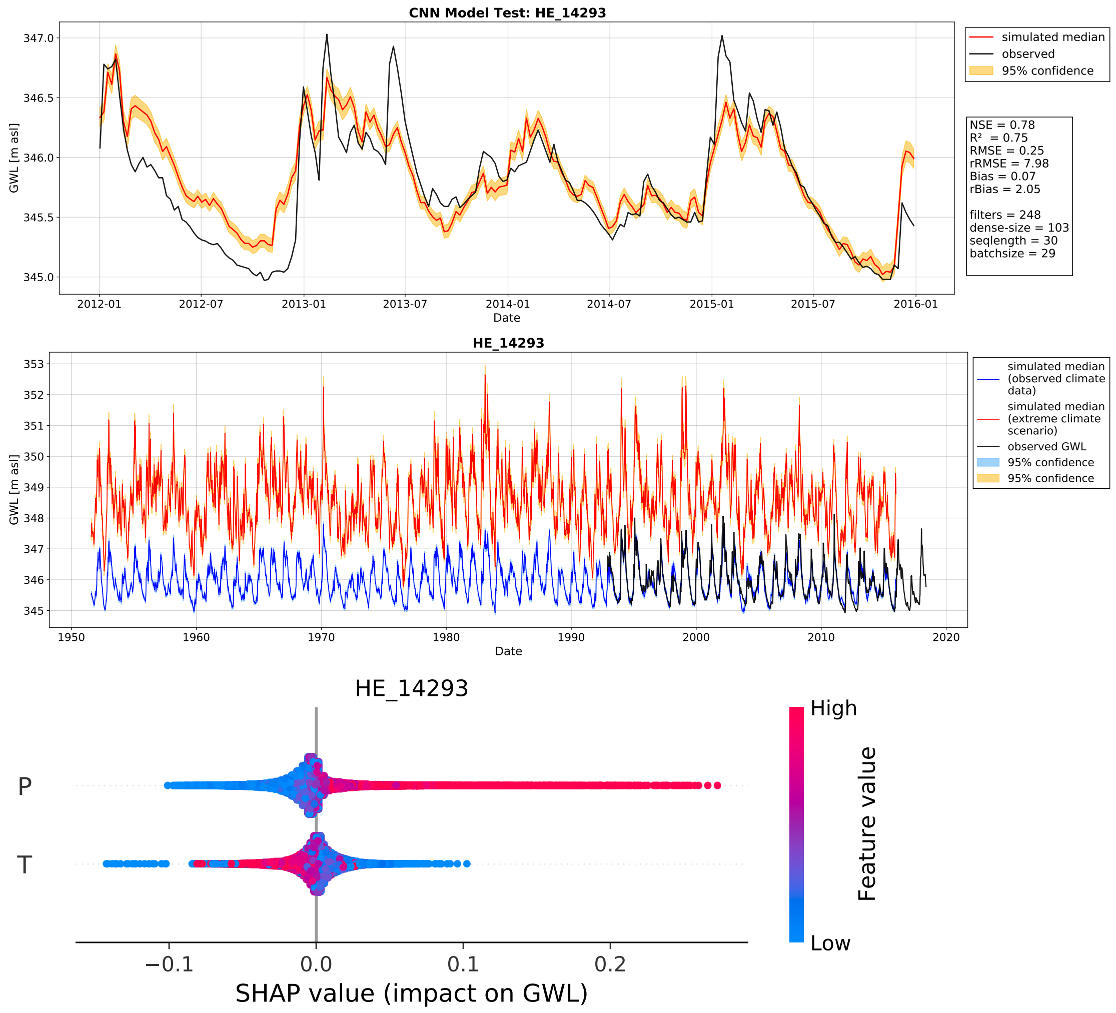

Figure S56: Evaluation of HE_14293 Model Performance in the past (upper), under extreme climate conditions (middle) and SHAP Summary plot (lower)

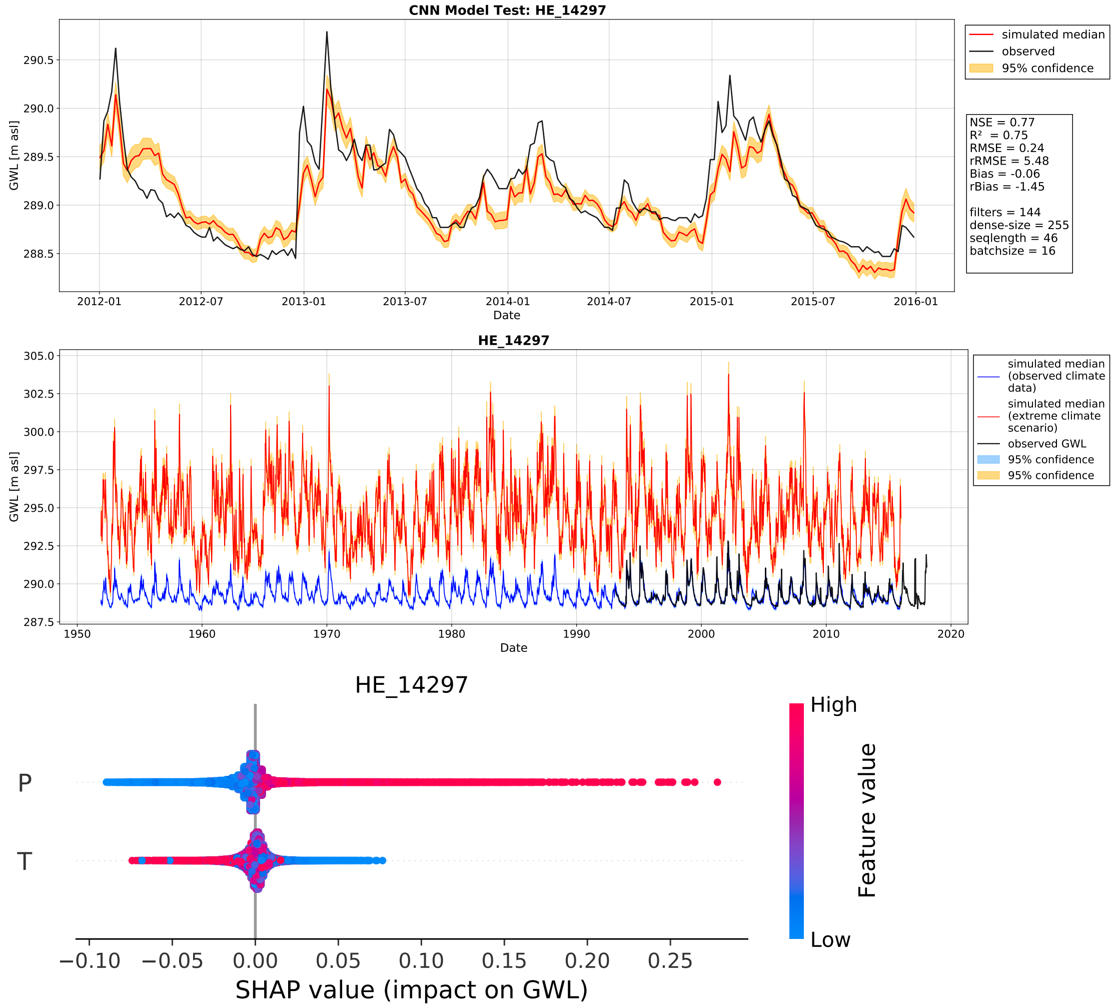

Figure S57: Evaluation of HE_14297 Model Performance in the past (upper), under extreme climate conditions (middle) and SHAP Summary plot (lower)

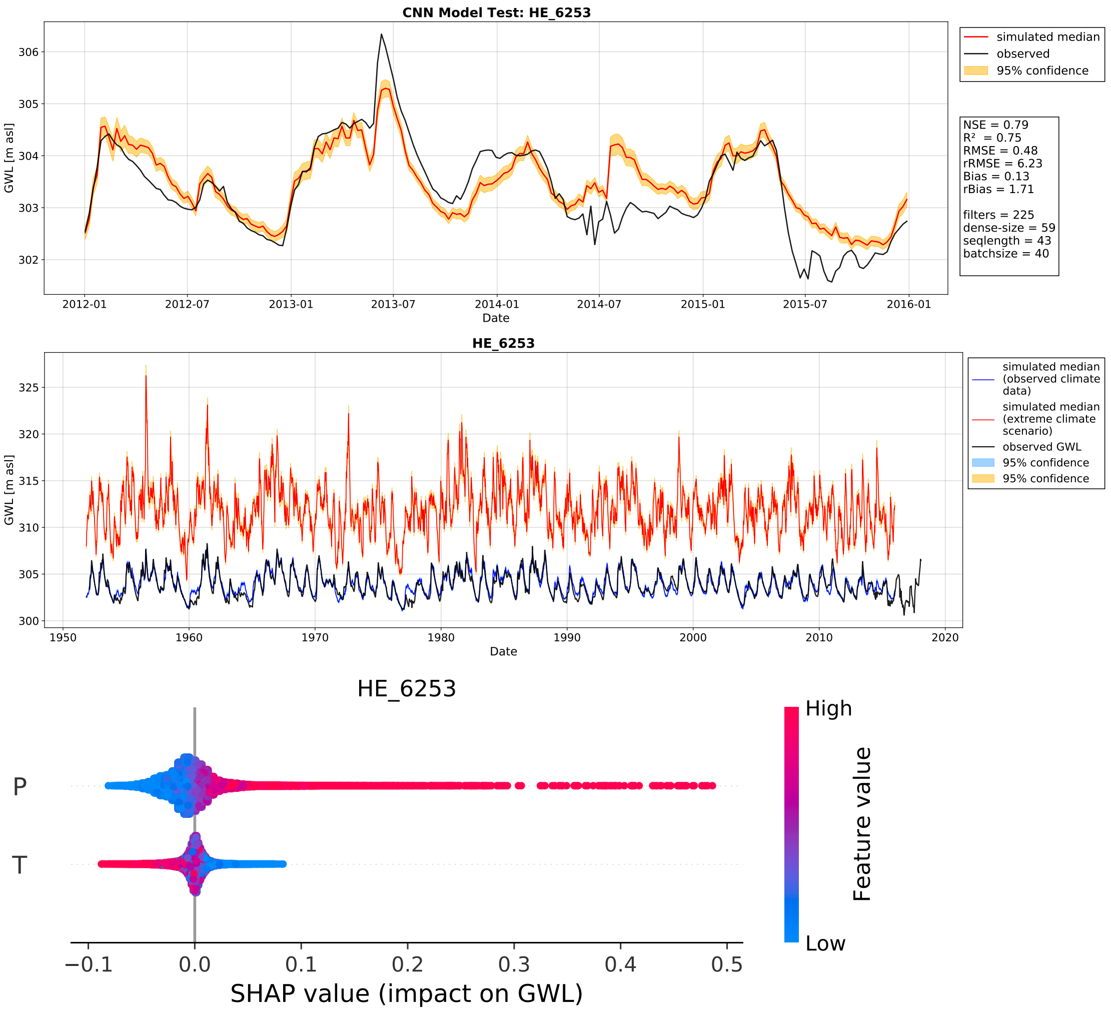

Figure S58: Evaluation of HE_6253 Model Performance in the past (upper), under extreme climate conditions (middle) and SHAP Summary plot (lower)

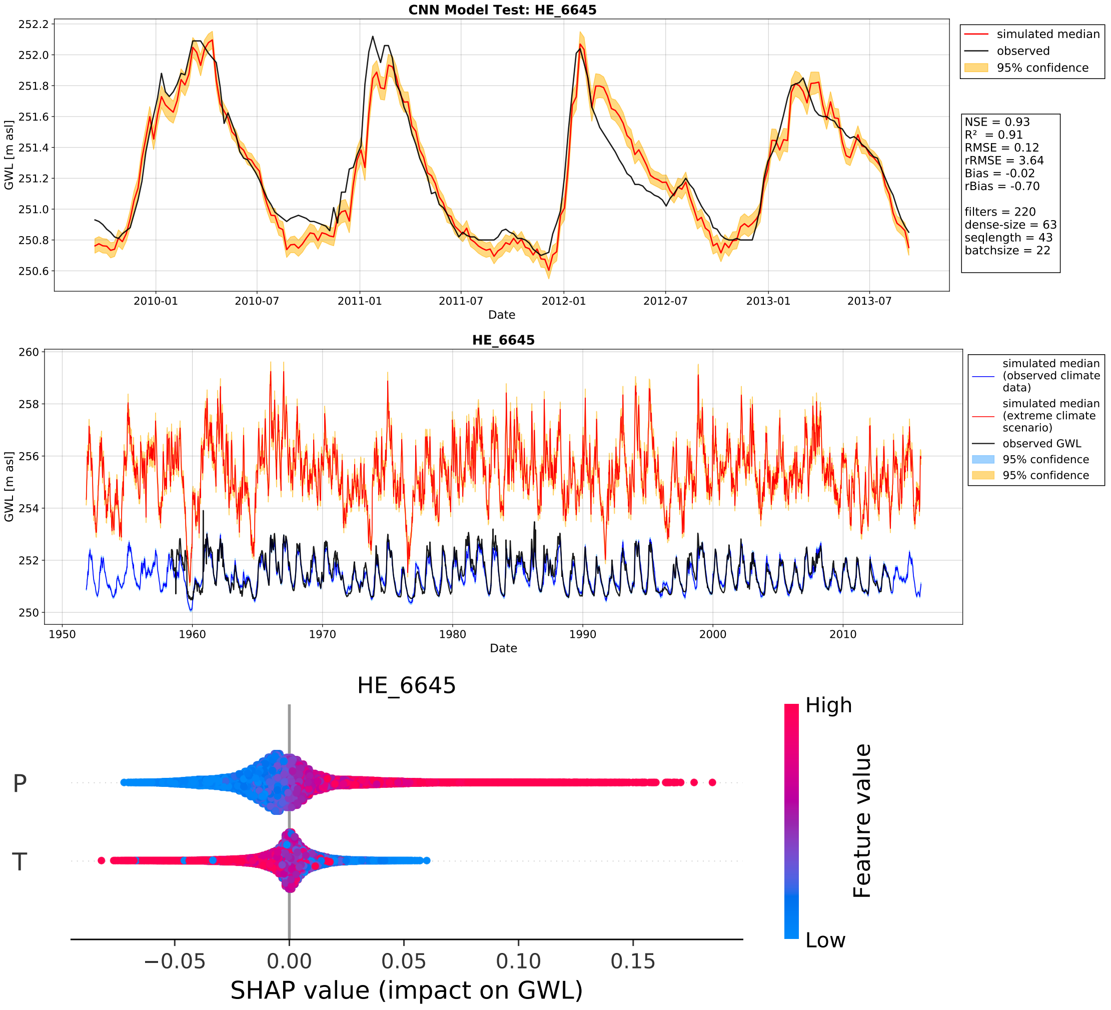

Figure S59: Evaluation of HE_6645 Model Performance in the past (upper), under extreme climate conditions (middle) and SHAP Summary plot (lower)

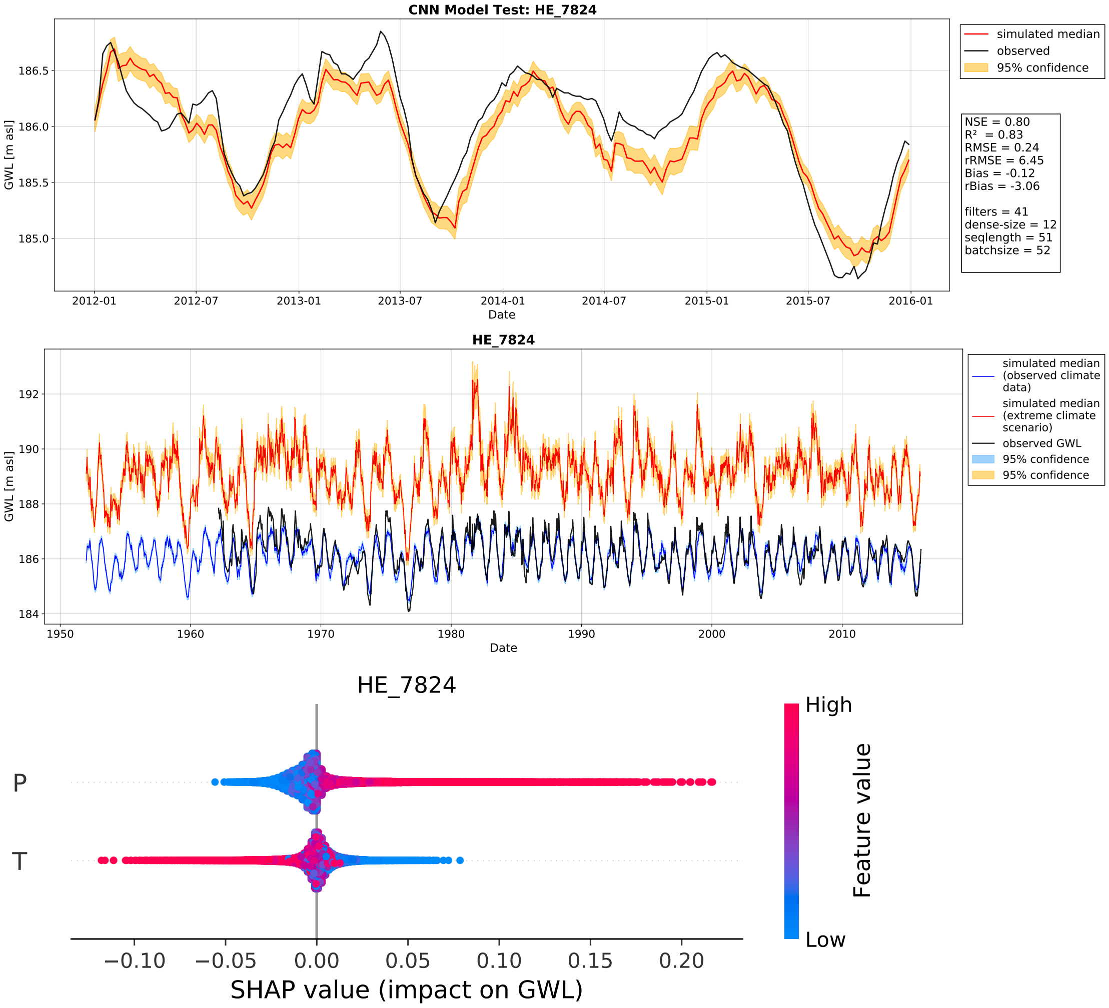

Figure S60: Evaluation of HE_7824 Model Performance in the past (upper), under extreme climate conditions (middle) and SHAP Summary plot (lower)

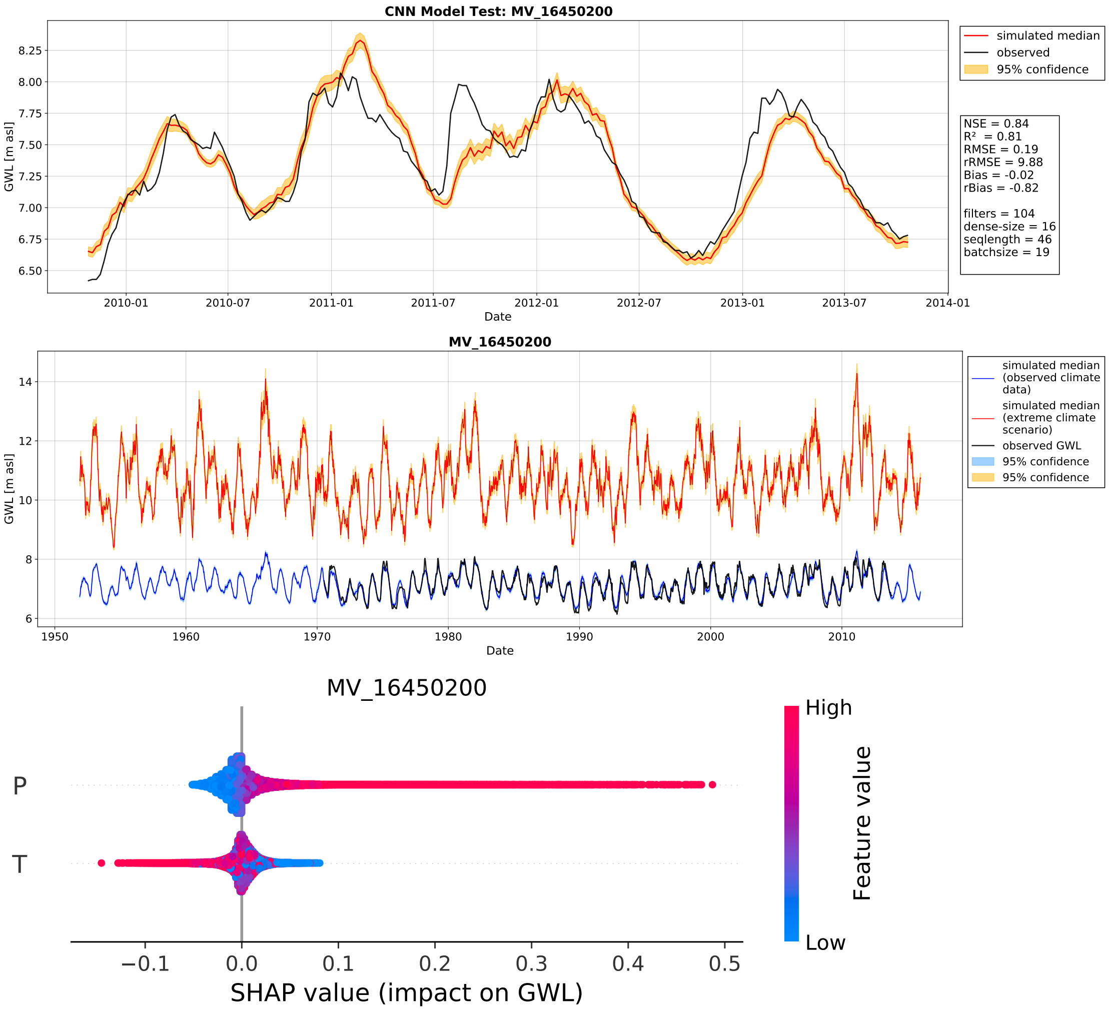

Figure S61: Evaluation of MV_16450200 Model Performance in the past (upper), under extreme climate conditions (middle) and SHAP Summary plot (lower)

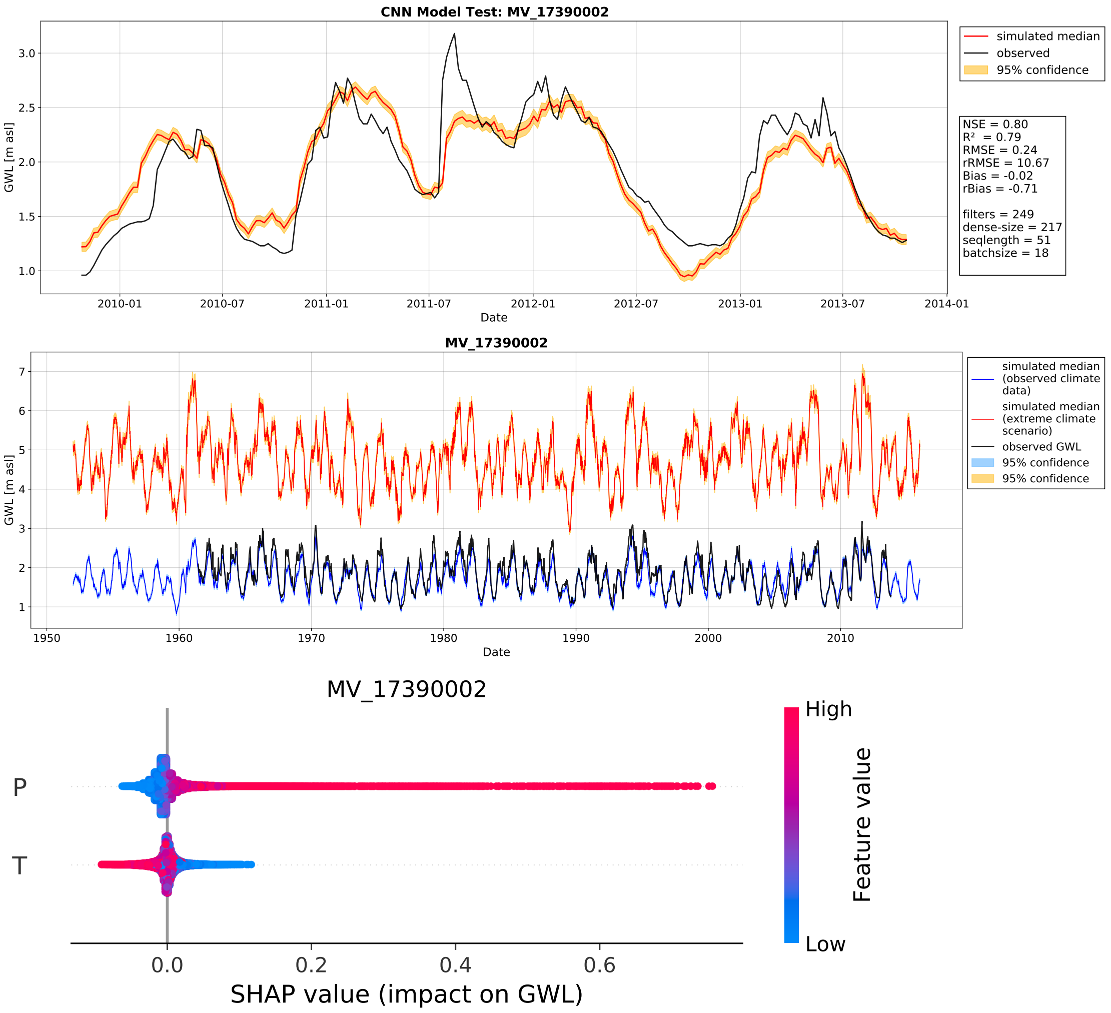

Figure S62: Evaluation of MV_17390002 Model Performance in the past (upper), under extreme climate conditions (middle) and SHAP Summary plot (lower)

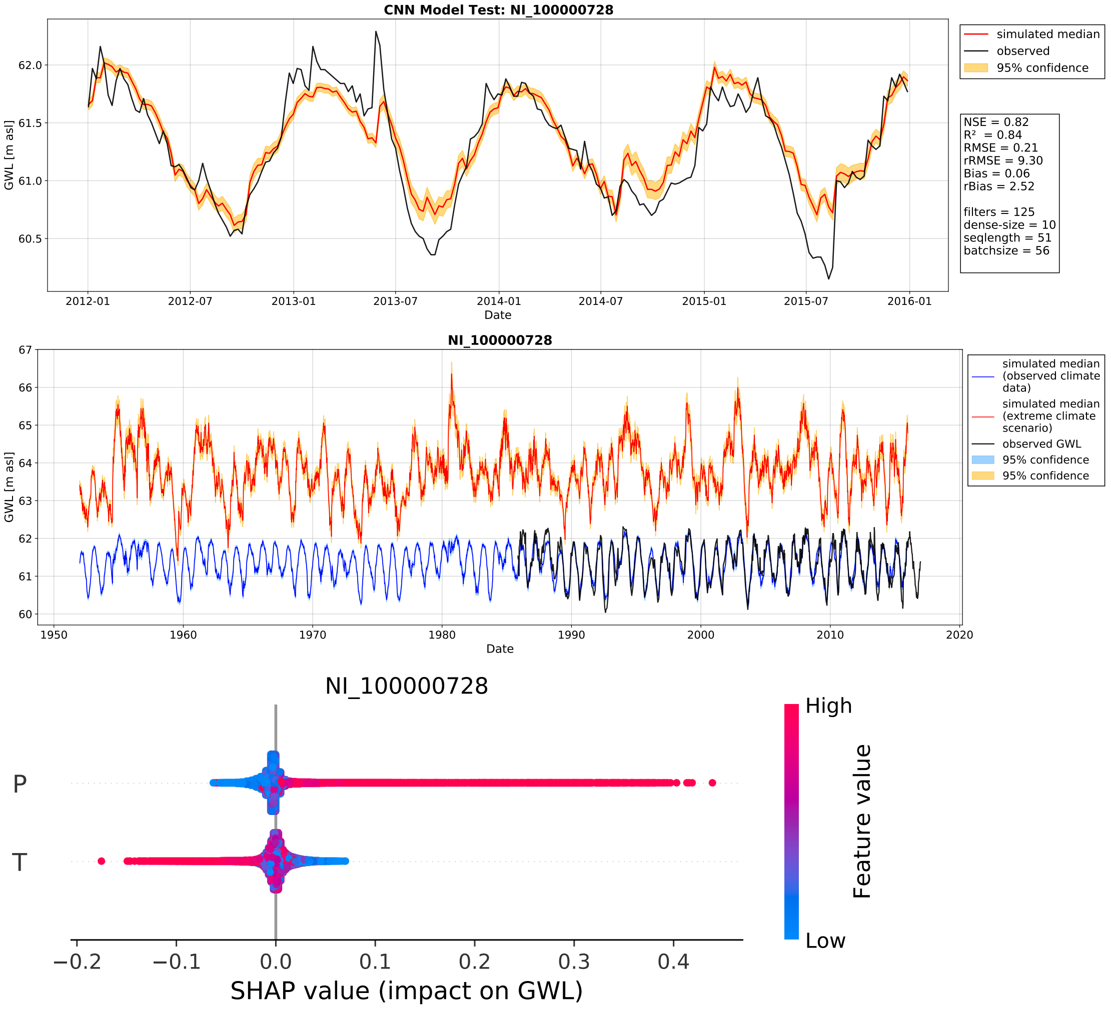

Figure S63: Evaluation of NI_100000728 Model Performance in the past (upper), under extreme climate conditions (middle) and SHAP Summary plot (lower)

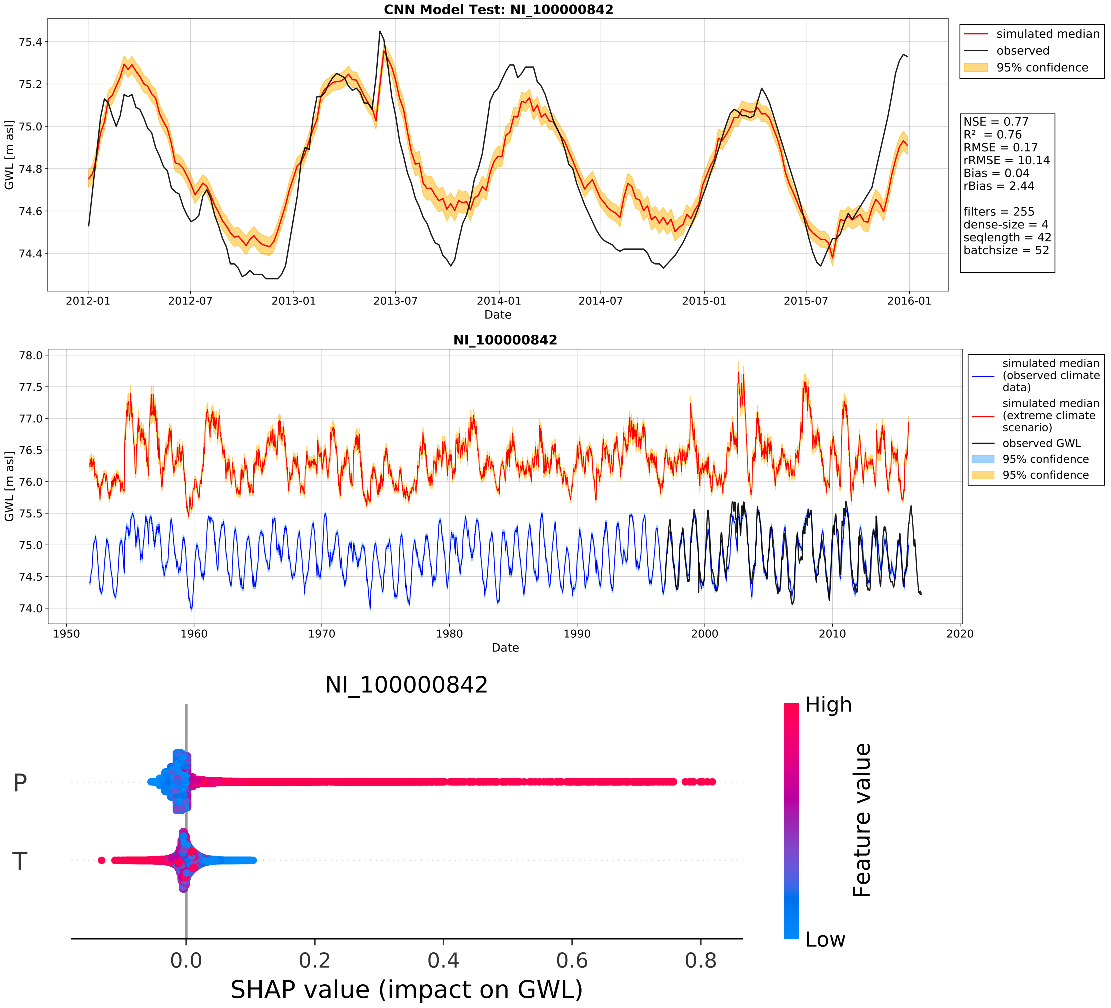

Figure S64: Evaluation of NI_100000842 Model Performance in the past (upper), under extreme climate conditions (middle) and SHAP Summary plot (lower)

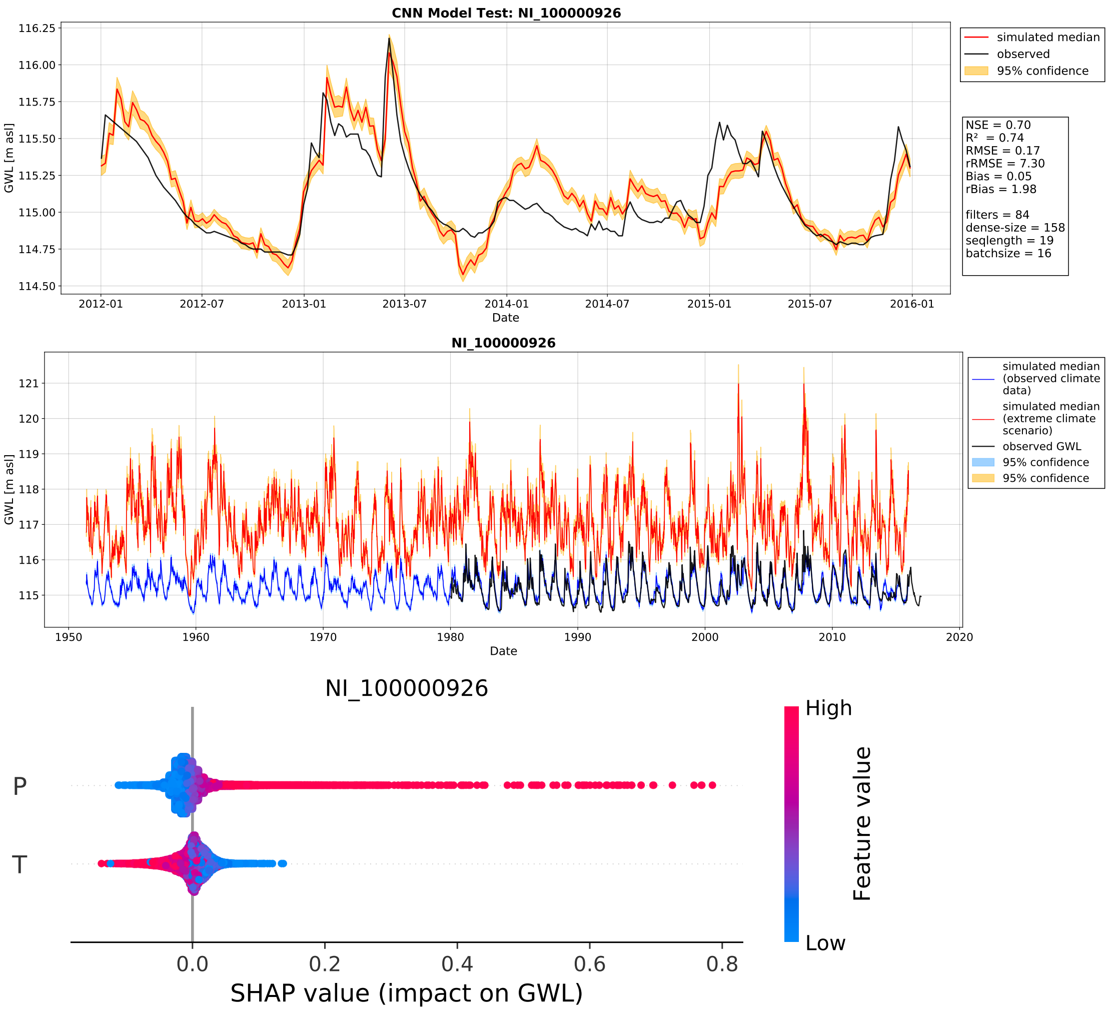

Figure S65: Evaluation of NI_100000926 Model Performance in the past (upper), under extreme climate conditions (middle) and SHAP Summary plot (lower)

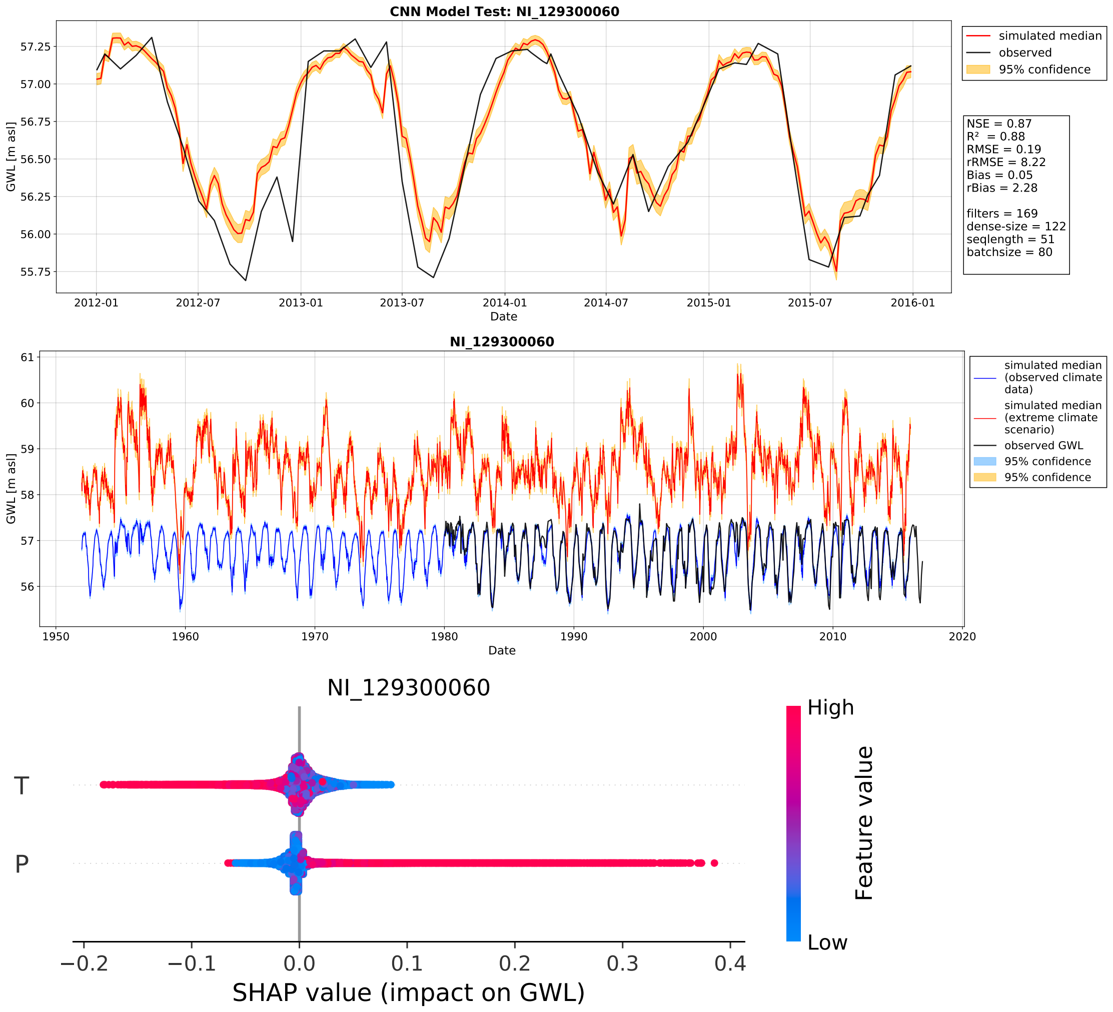

Figure S66: Evaluation of NI_129300060 Model Performance in the past (upper), under extreme climate conditions (middle) and SHAP Summary plot (lower)

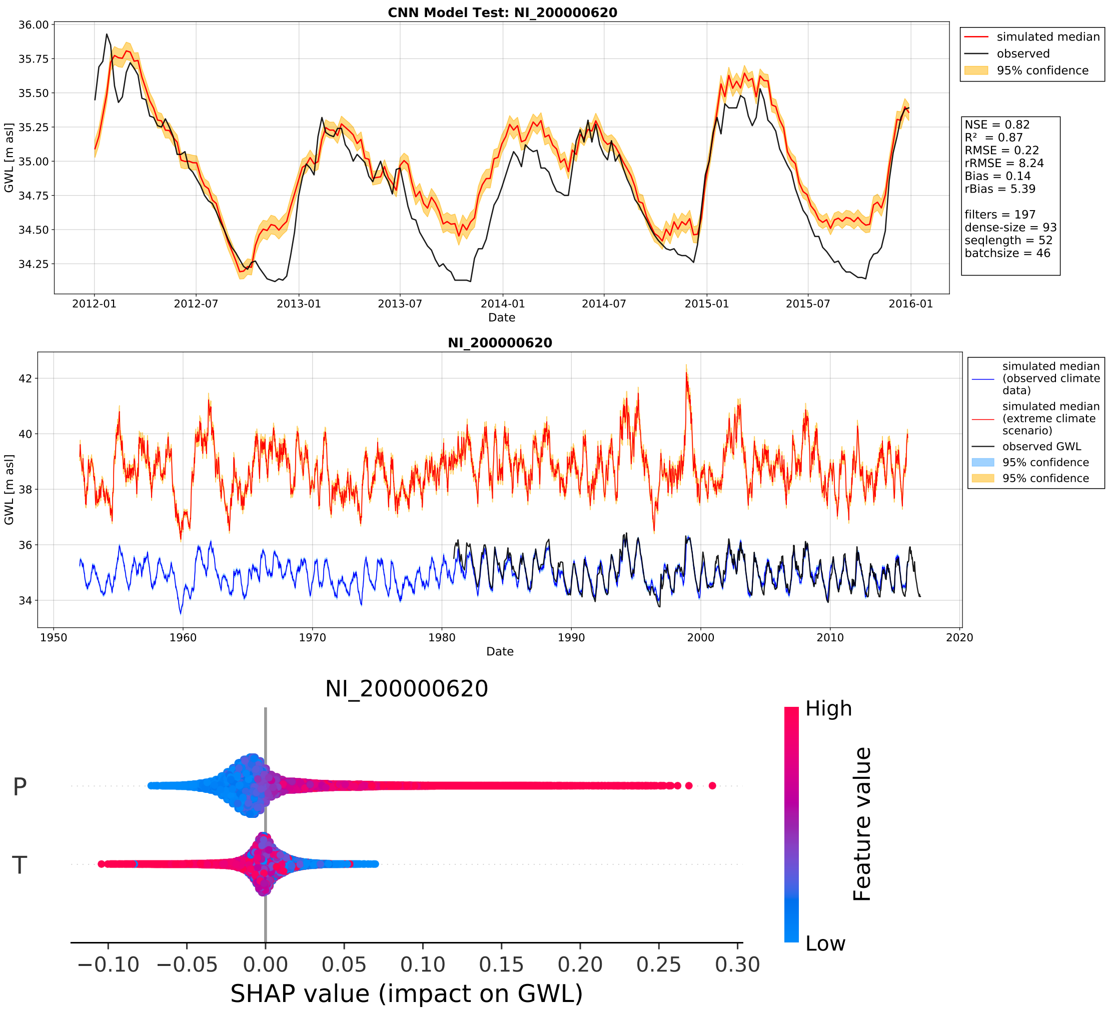

Figure S67: Evaluation of NI_200000620 Model Performance in the past (upper), under extreme climate conditions (middle) and SHAP Summary plot (lower)

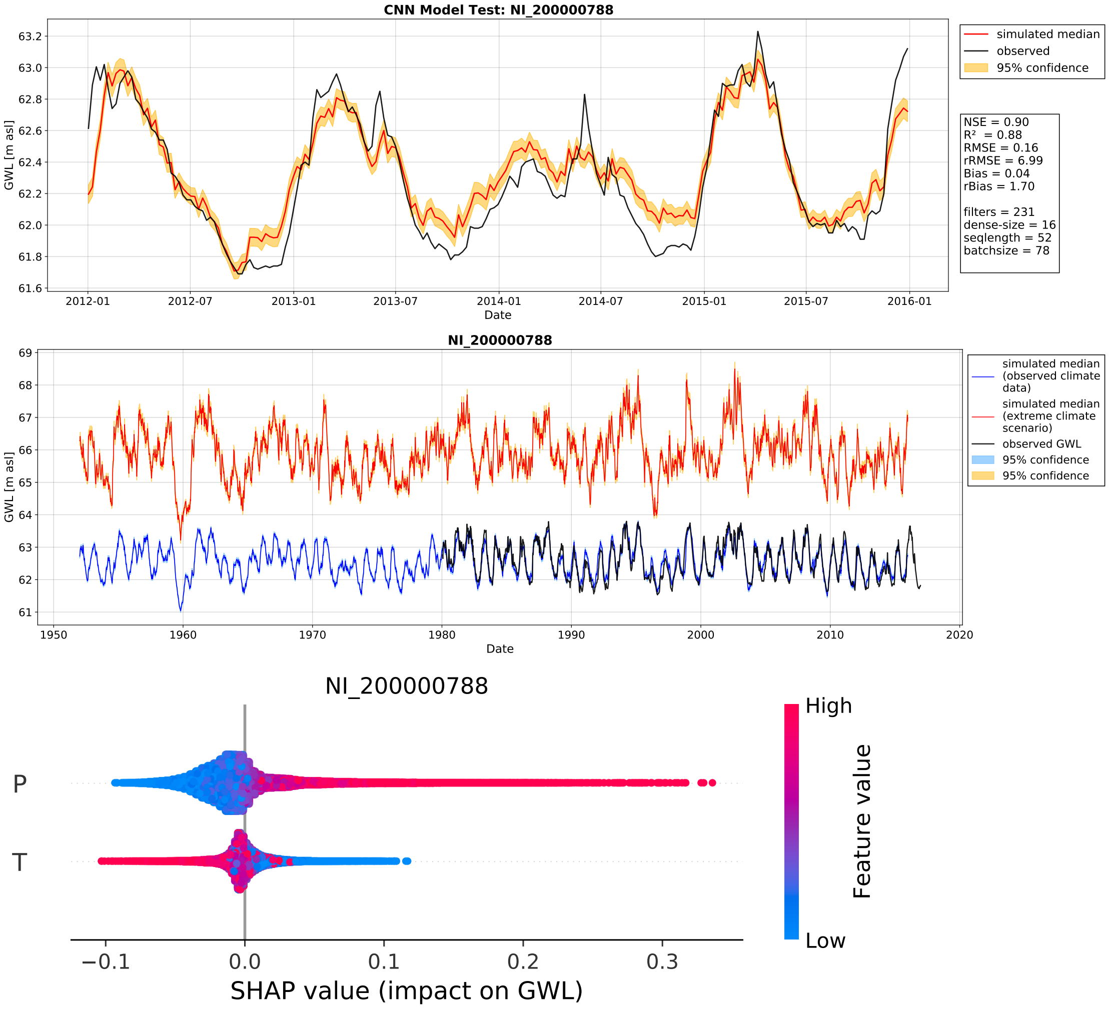

Figure S68: Evaluation of NI_200000788 Model Performance in the past (upper), under extreme climate conditions (middle) and SHAP Summary plot (lower)

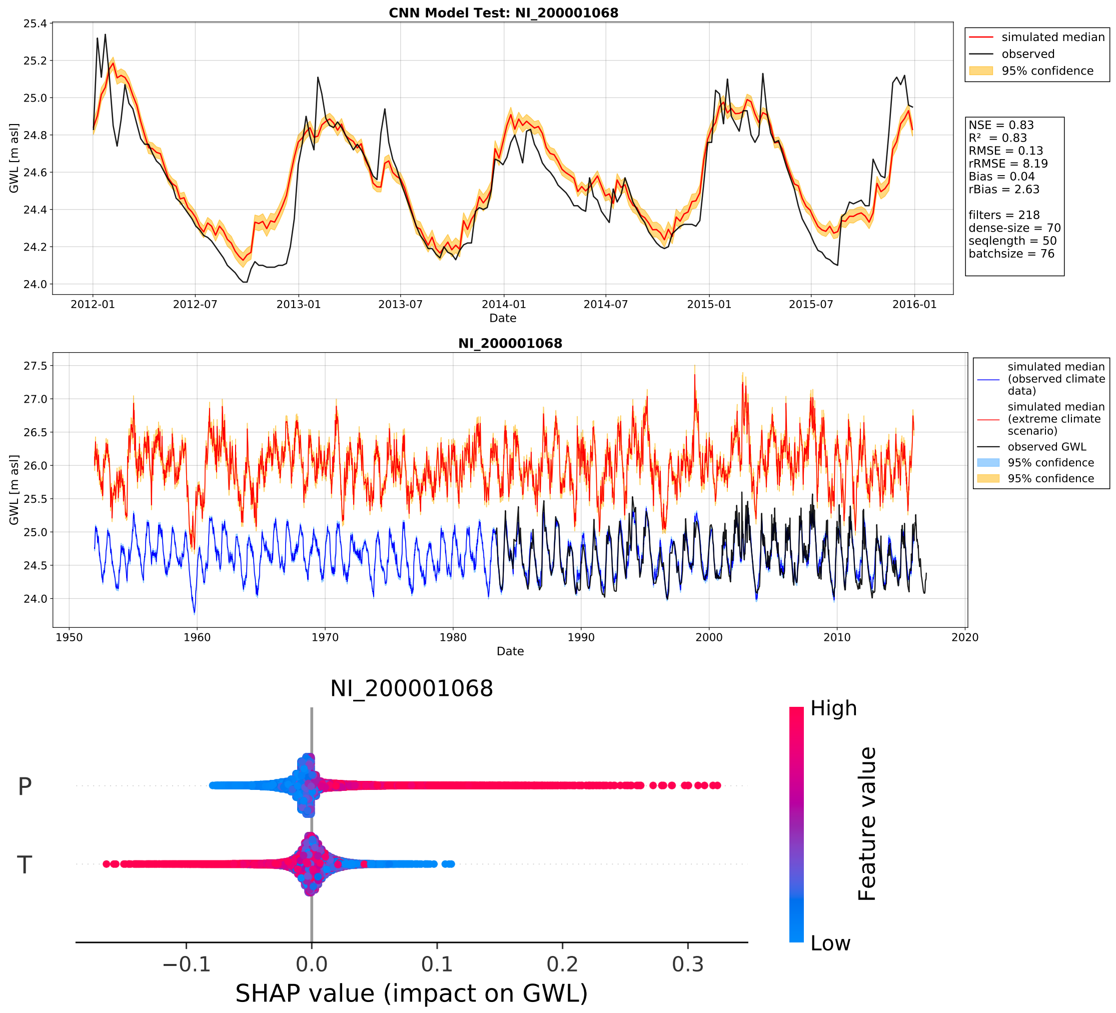

Figure S69: Evaluation of NI_200001068 Model Performance in the past (upper), under extreme climate conditions (middle) and SHAP Summary plot (lower)

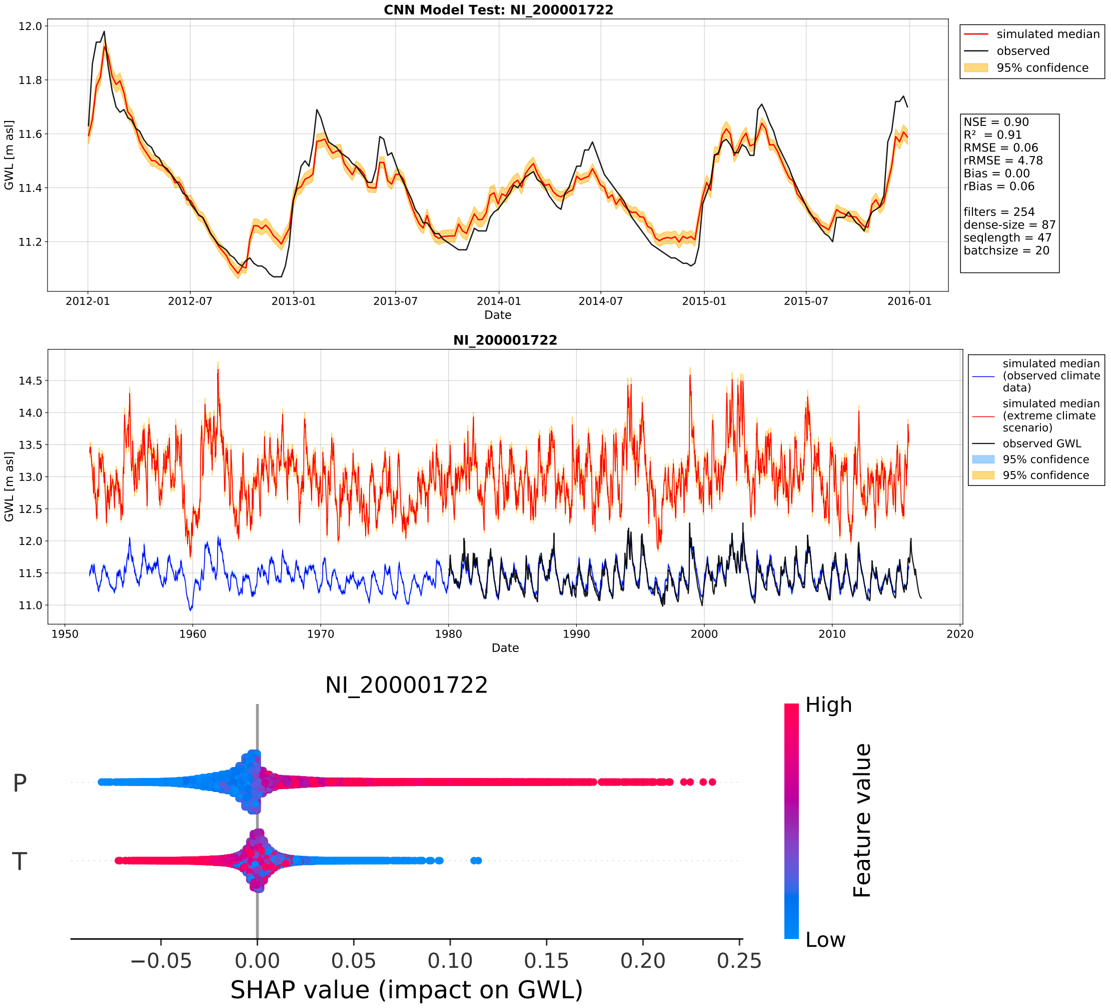

Figure S70: Evaluation of NI_200001722 Model Performance in the past (upper), under extreme climate conditions (middle) and SHAP Summary plot (lower)

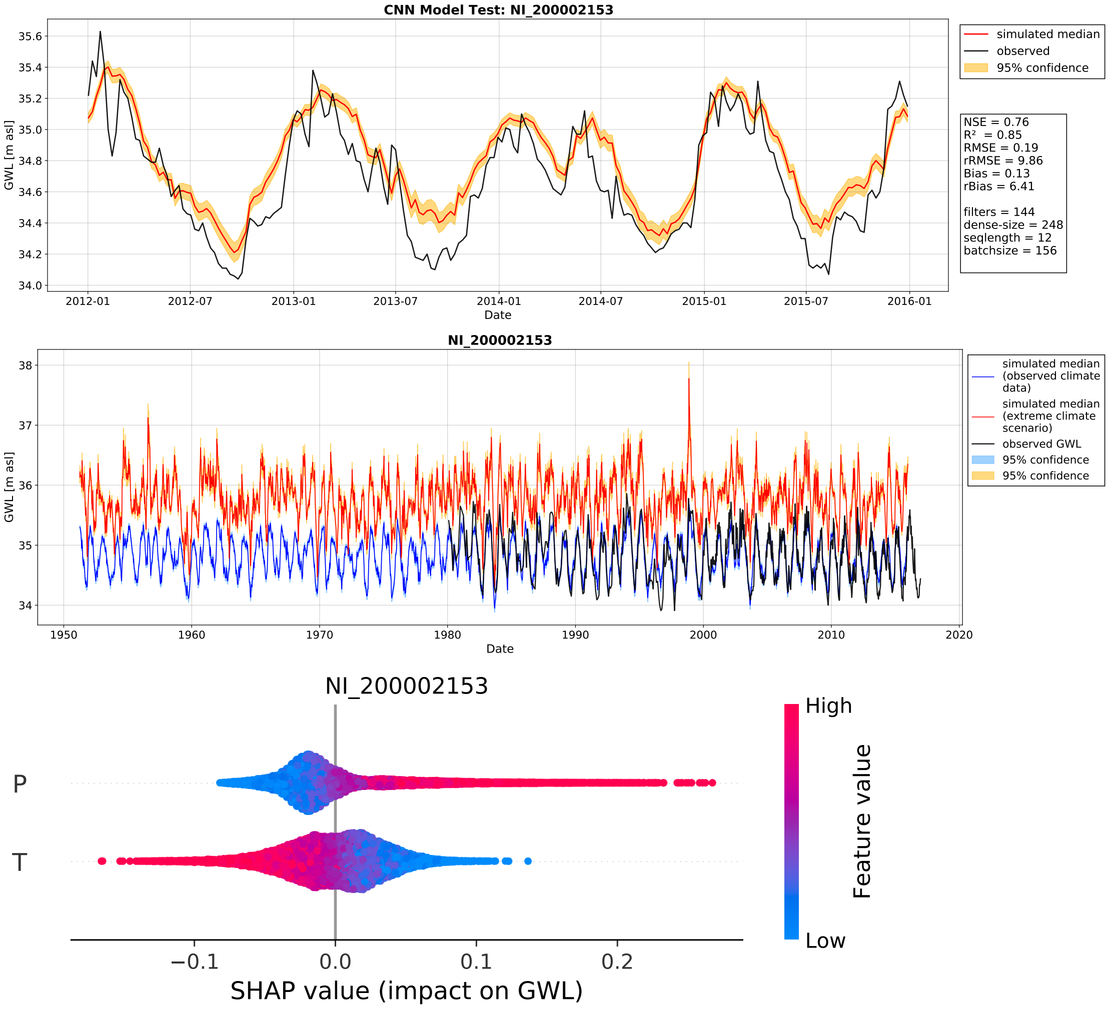

Figure S71: Evaluation of NI_200002153 Model Performance in the past (upper), under extreme climate conditions (middle) and SHAP Summary plot (lower)

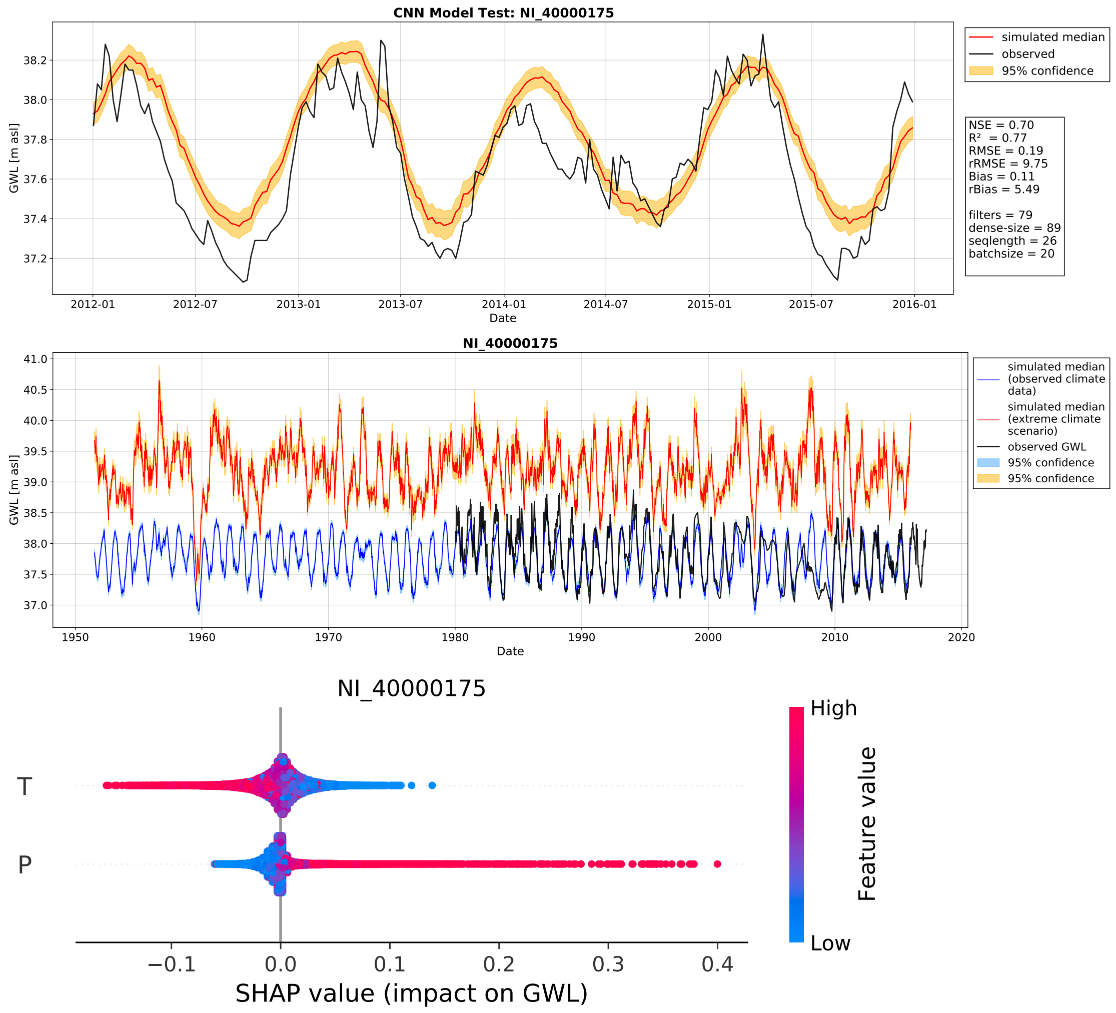

Figure S72: Evaluation of NI_40000175 Model Performance in the past (upper), under extreme climate conditions (middle) and SHAP Summary plot (lower)

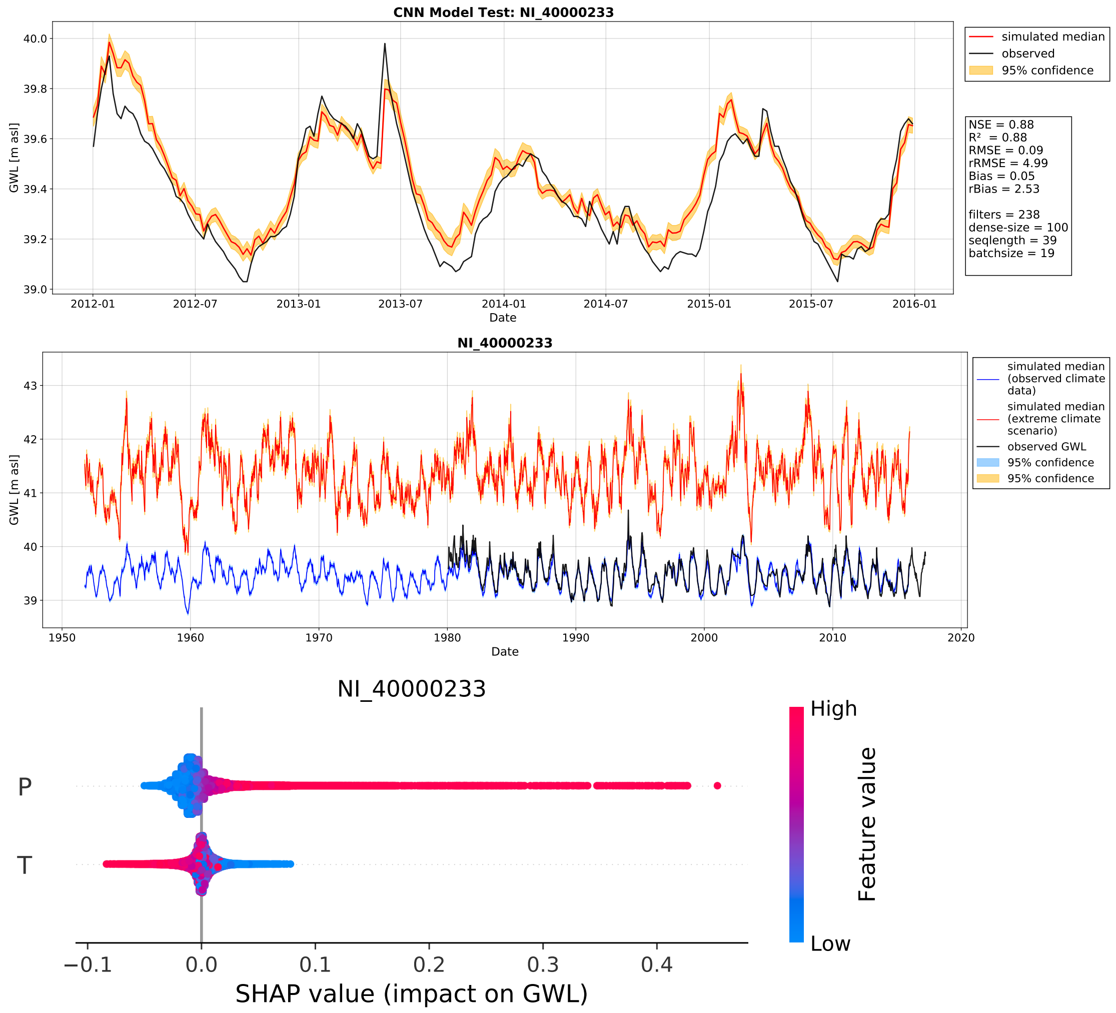

Figure S73: Evaluation of NI_40000233 Model Performance in the past (upper), under extreme climate conditions (middle) and SHAP Summary plot (lower)

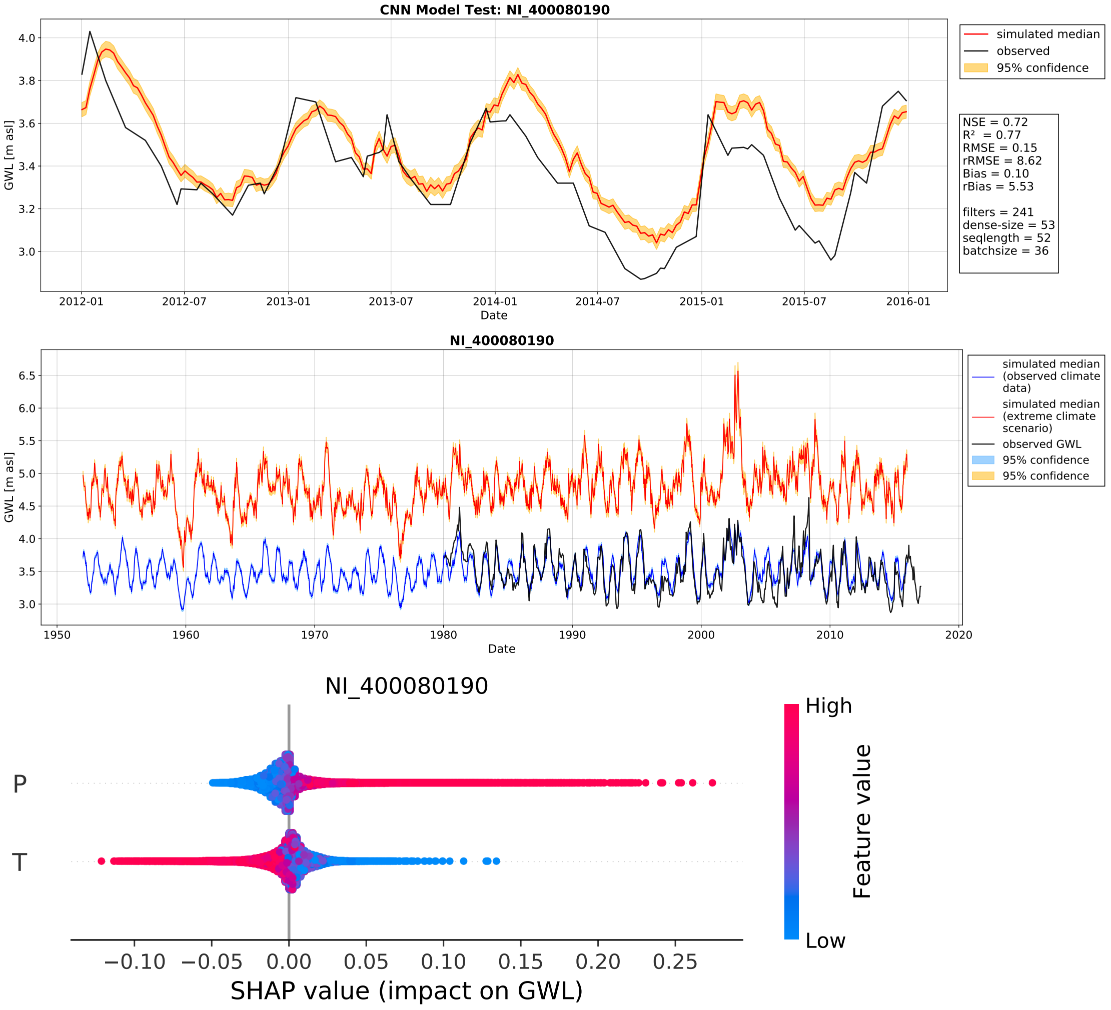

Figure S74: Evaluation of NI_400080190 Model Performance in the past (upper), under extreme climate conditions (middle) and SHAP Summary plot (lower)

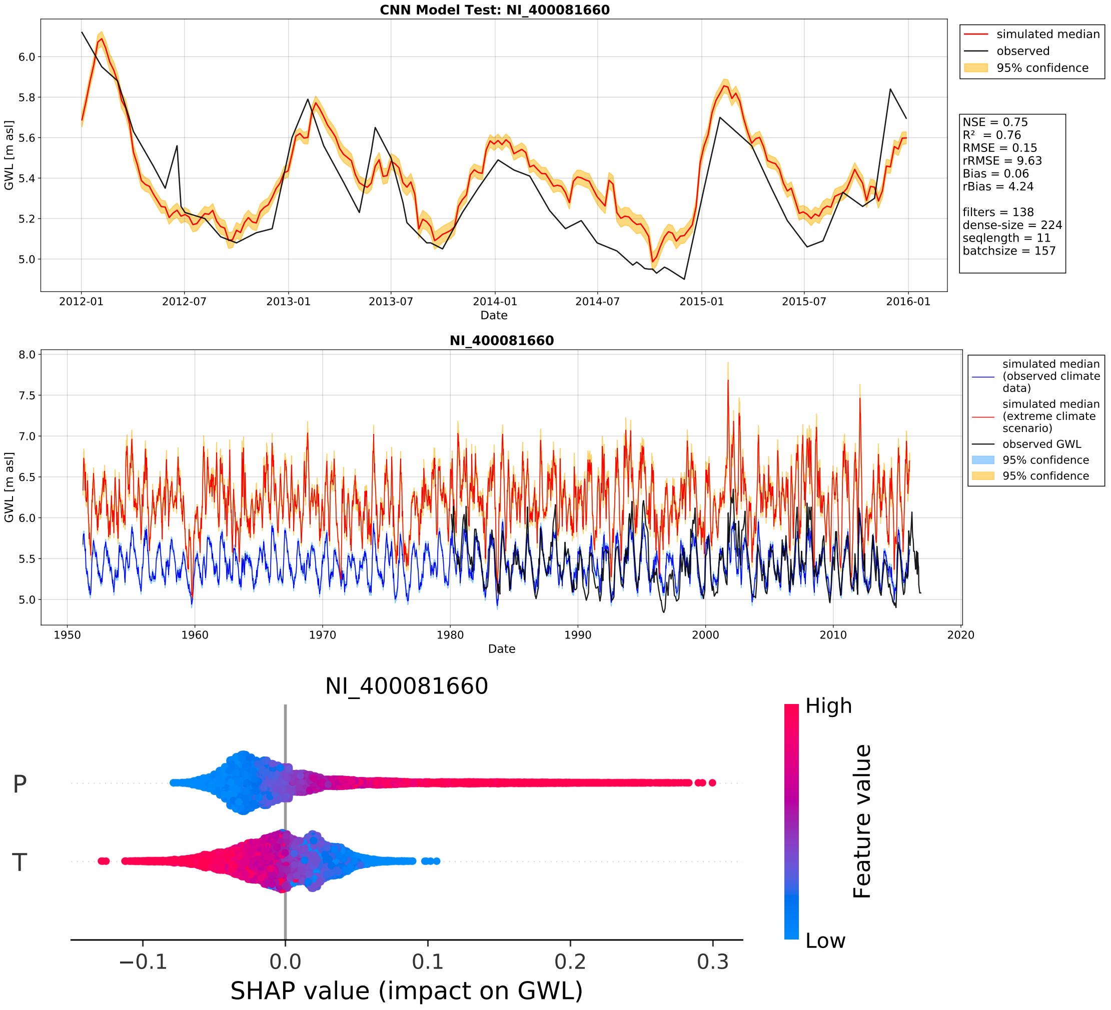

Figure S75: Evaluation of NI_400081660 Model Performance in the past (upper), under extreme climate conditions (middle) and SHAP Summary plot (lower)

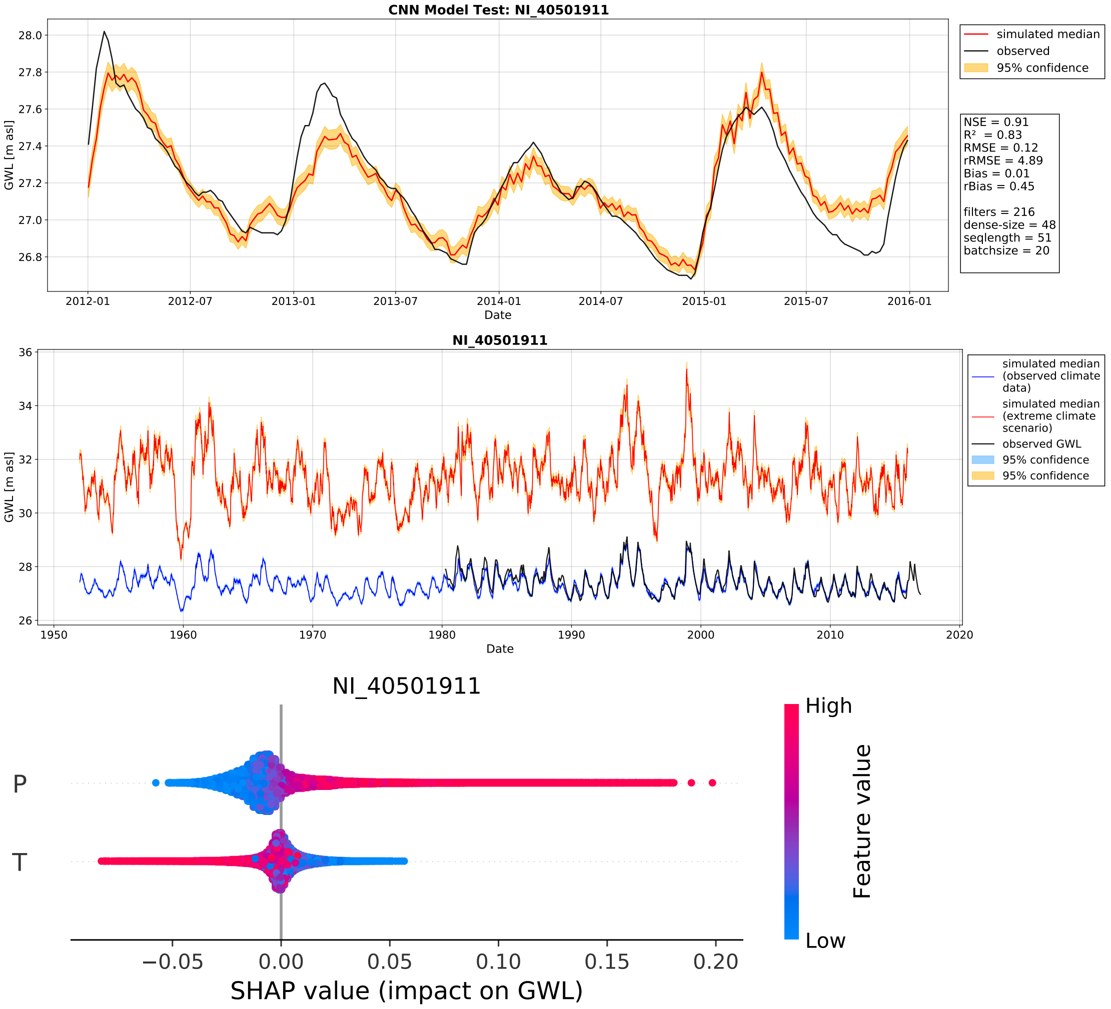

Figure S76: Evaluation of NI_40501911 Model Performance in the past (upper), under extreme climate conditions (middle) and SHAP Summary plot (lower)

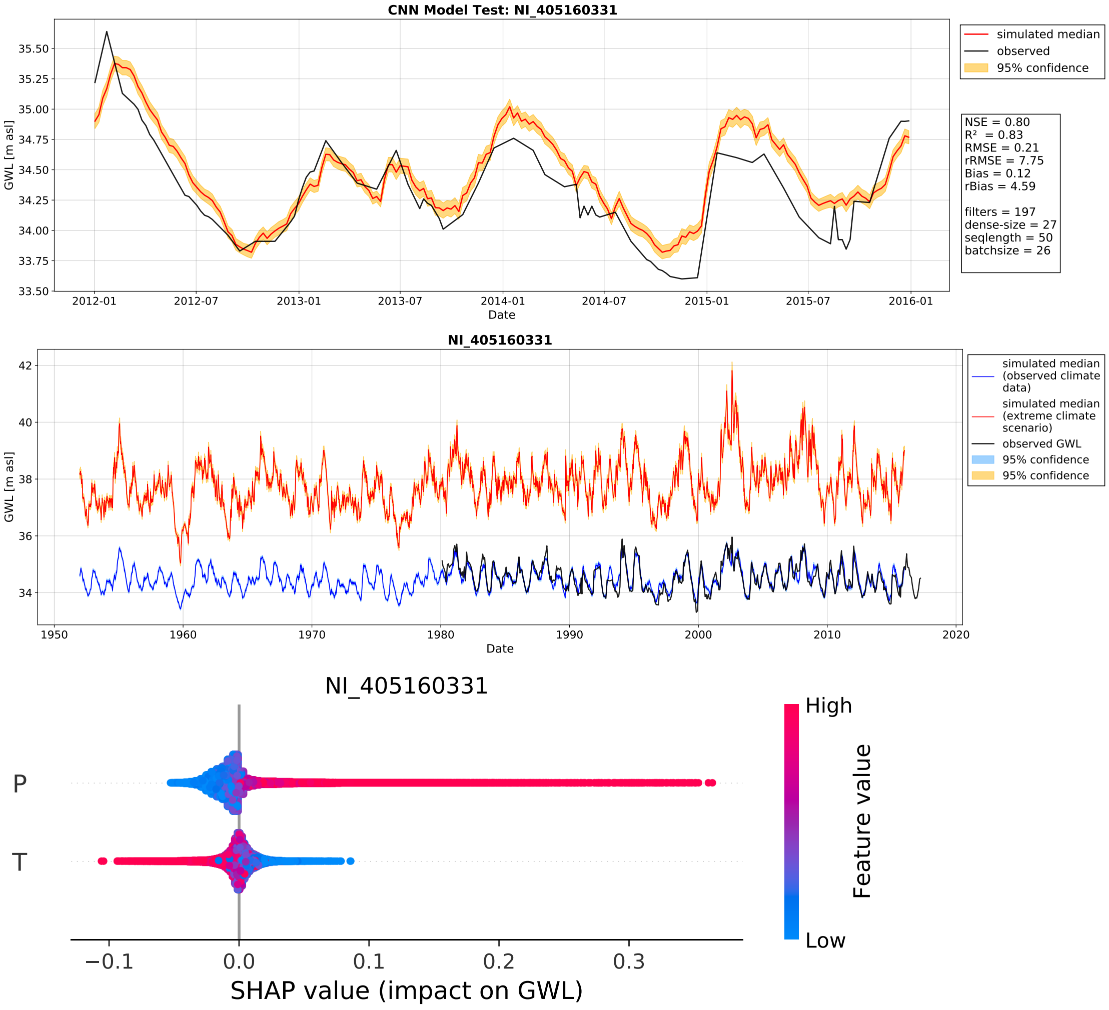

Figure S77: Evaluation of NI_405160331 Model Performance in the past (upper), under extreme climate conditions (middle) and SHAP Summary plot (lower)

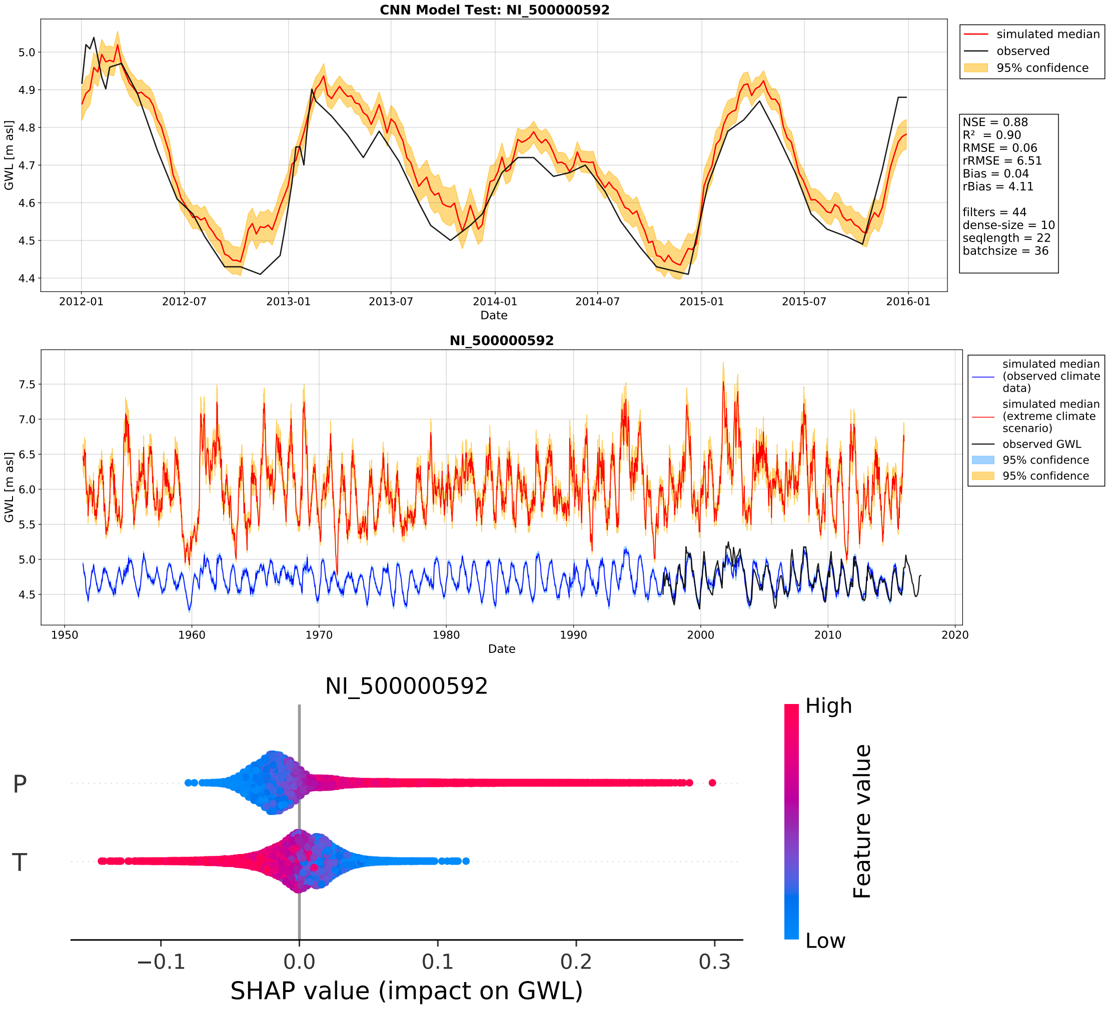

Figure S78: Evaluation of NI_500000592 Model Performance in the past (upper), under extreme climate conditions (middle) and SHAP Summary plot (lower)

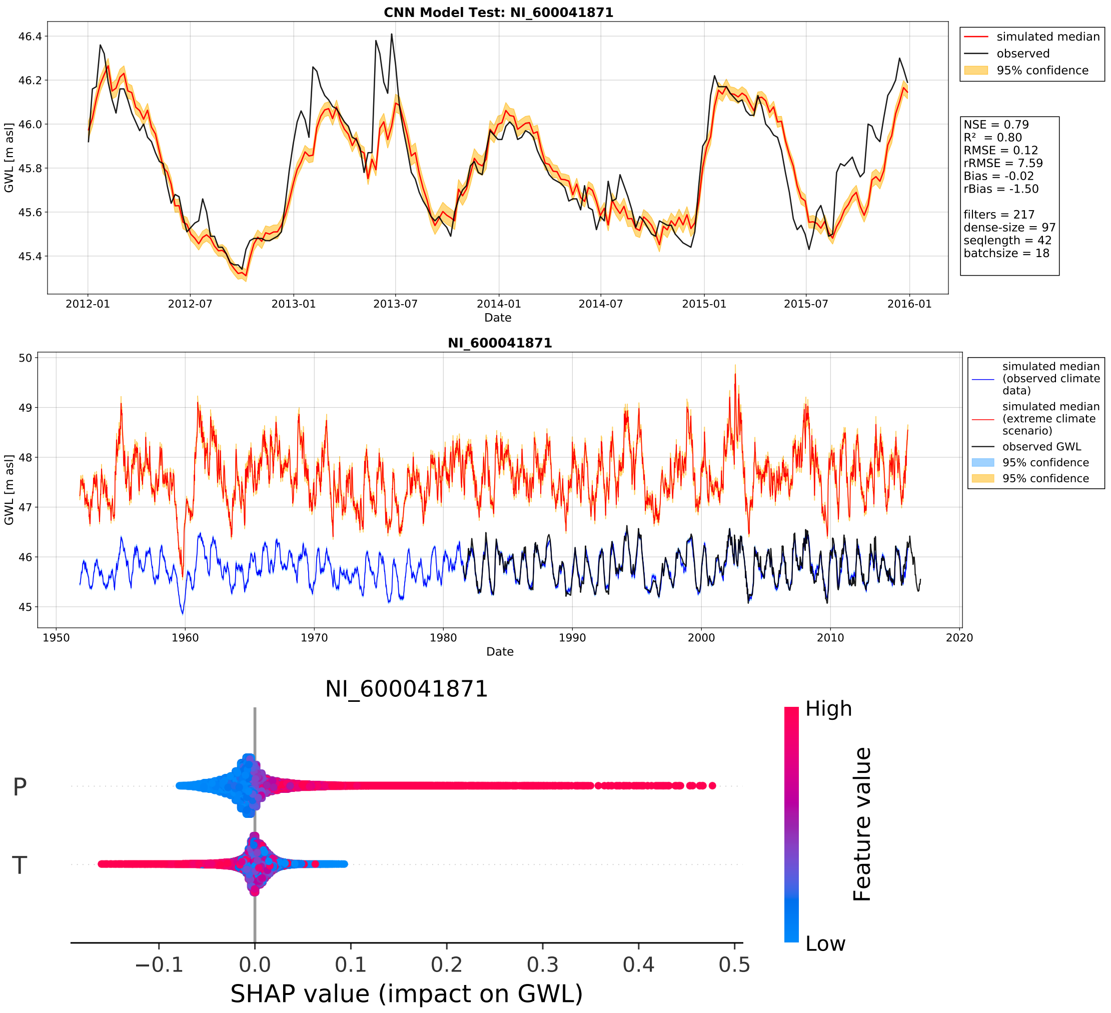

Figure S79: Evaluation of NI_600041871 Model Performance in the past (upper), under extreme climate conditions (middle) and SHAP Summary plot (lower)

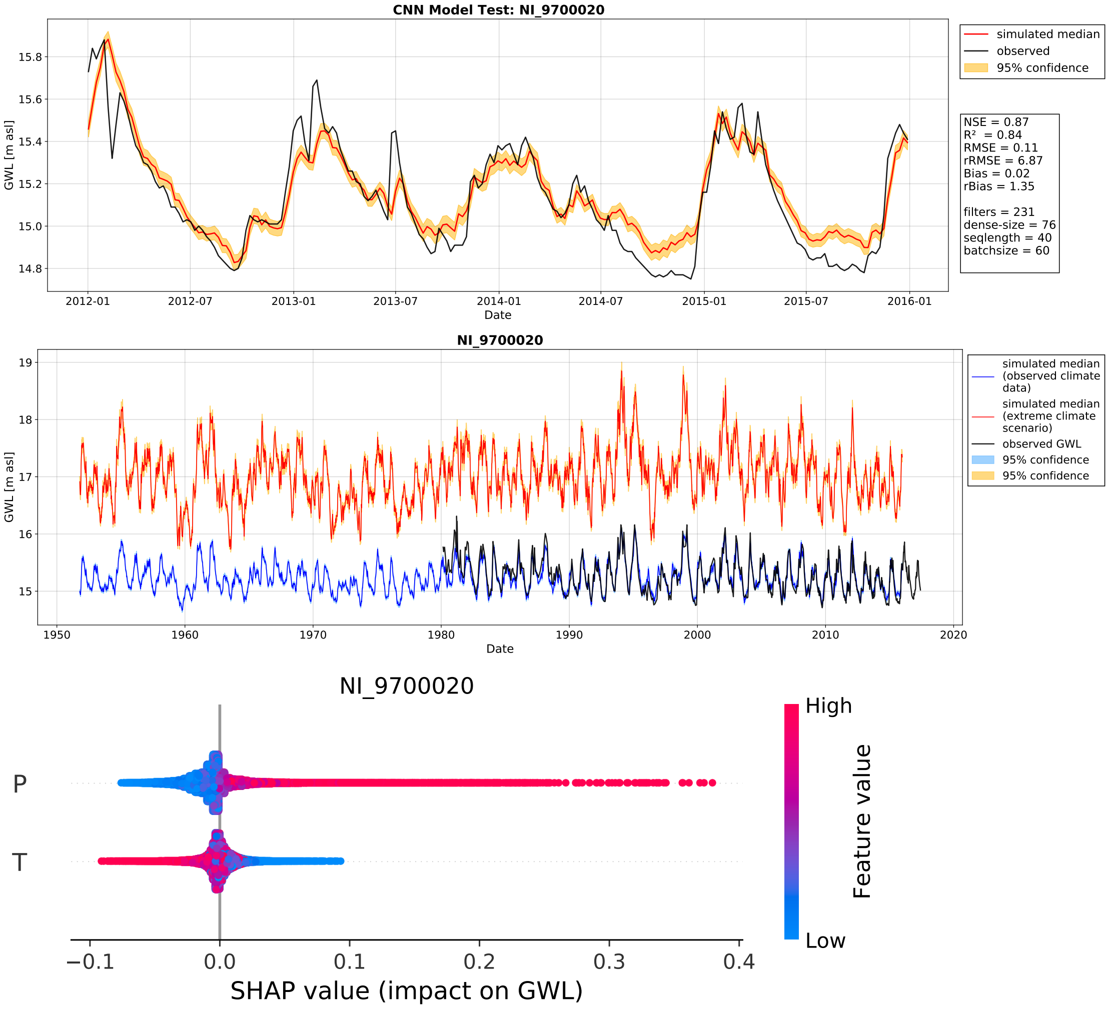

Figure S80: Evaluation of NI_9700020 Model Performance in the past (upper), under extreme climate conditions (middle) and SHAP Summary plot (lower)

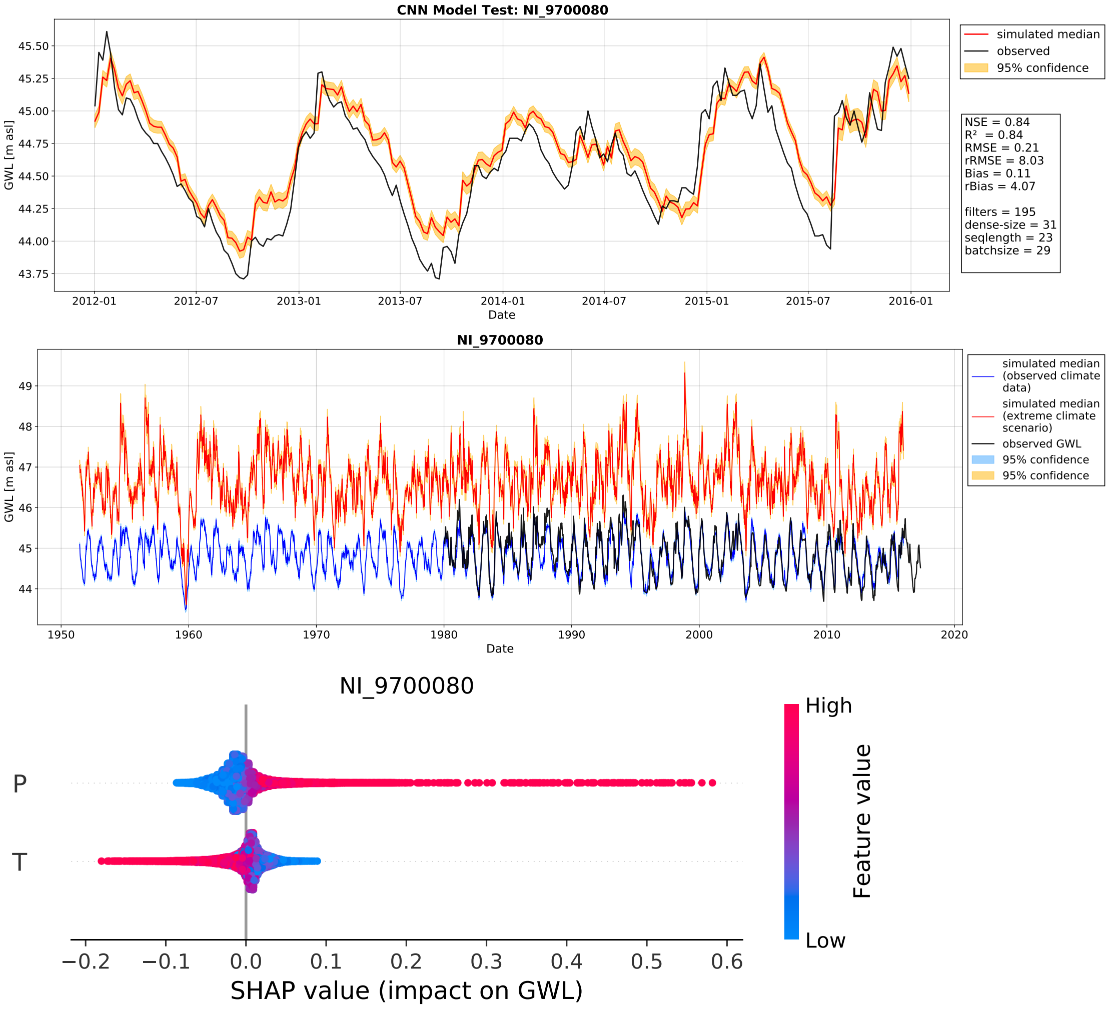

Figure S81: Evaluation of NI_9700080 Model Performance in the past (upper), under extreme climate conditions (middle) and SHAP Summary plot (lower)

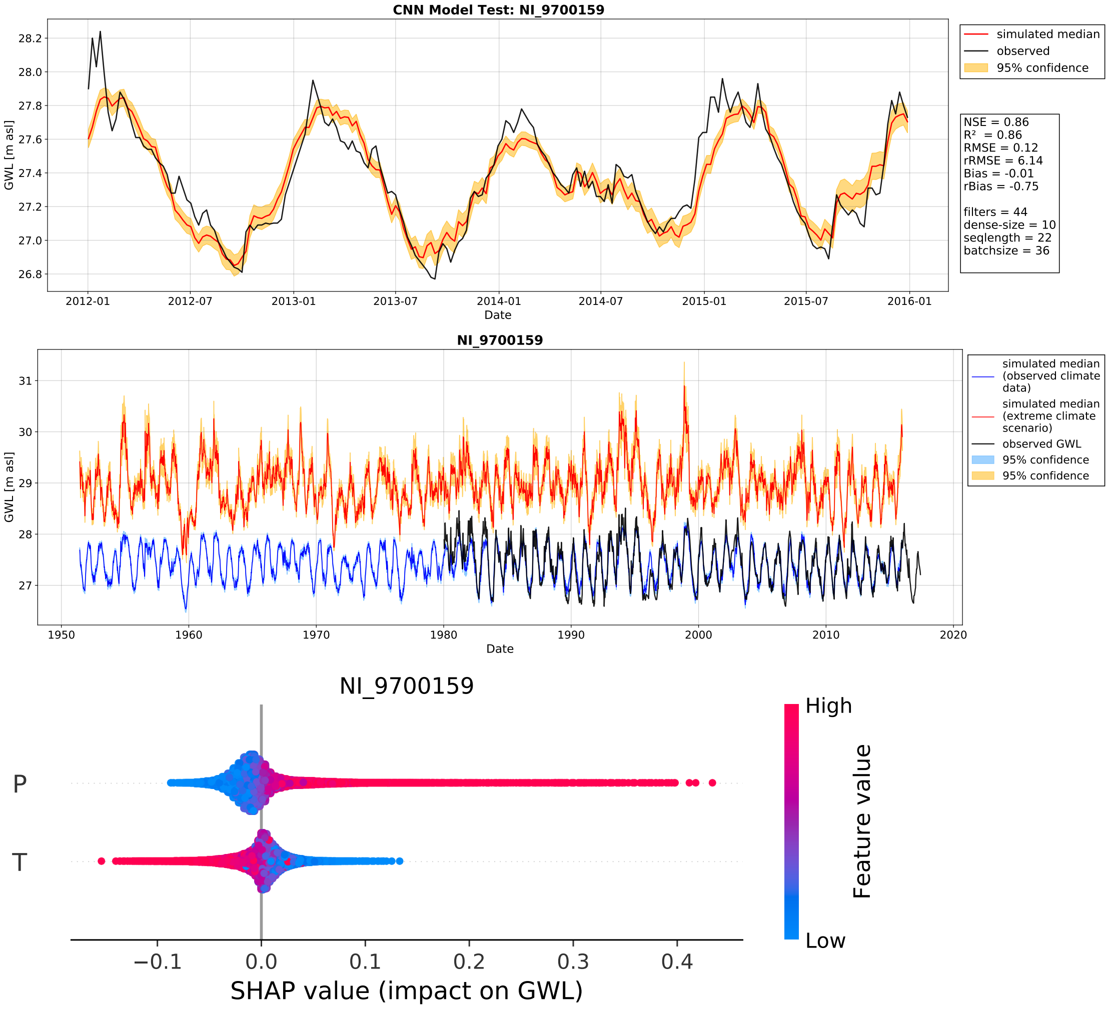

Figure S82: Evaluation of NI_9700159 Model Performance in the past (upper), under extreme climate conditions (middle) and SHAP Summary plot (lower)

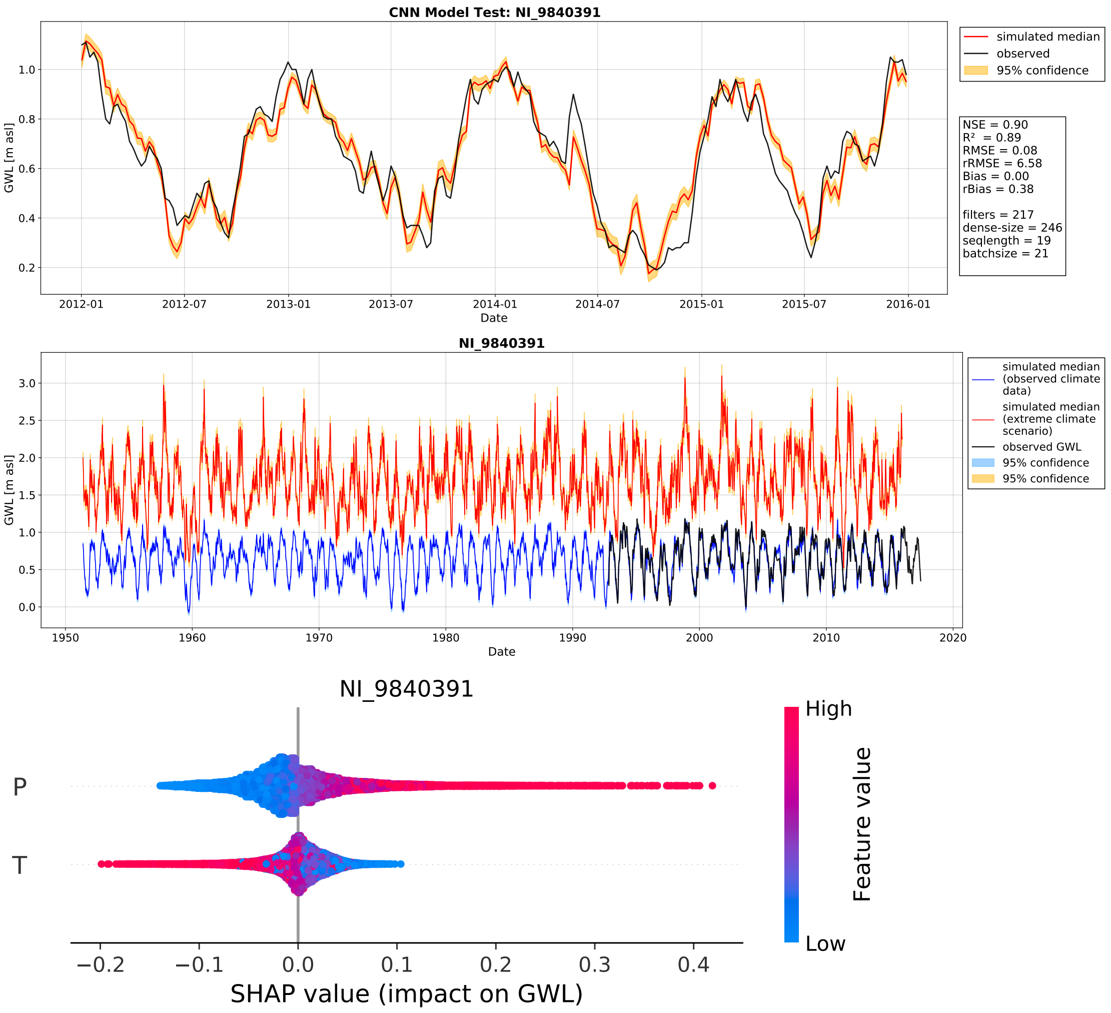

Figure S83: Evaluation of NI_9840391 Model Performance in the past (upper), under extreme climate conditions (middle) and SHAP Summary plot (lower)

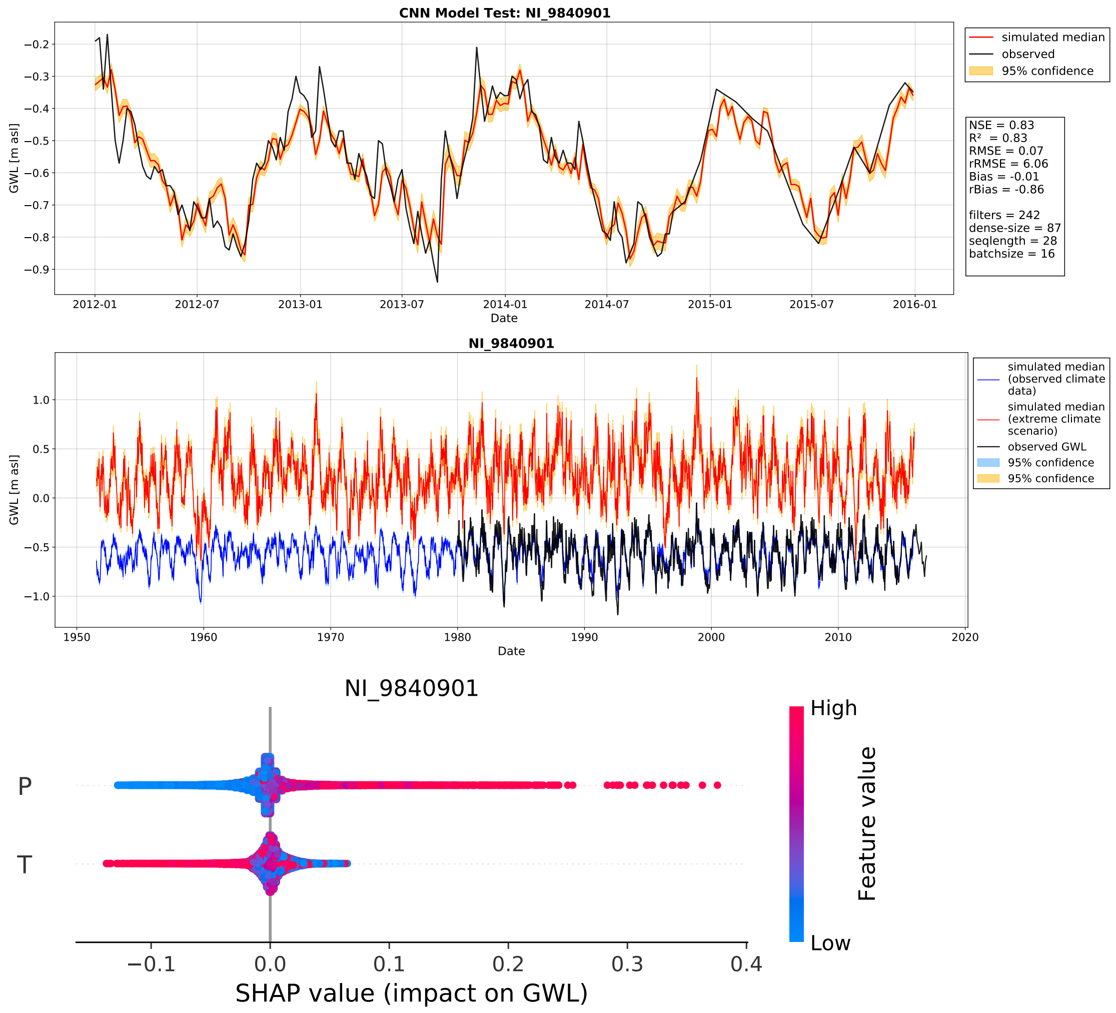

Figure S84: Evaluation of NI_9840901 Model Performance in the past (upper), under extreme climate conditions (middle) and SHAP Summary plot (lower)

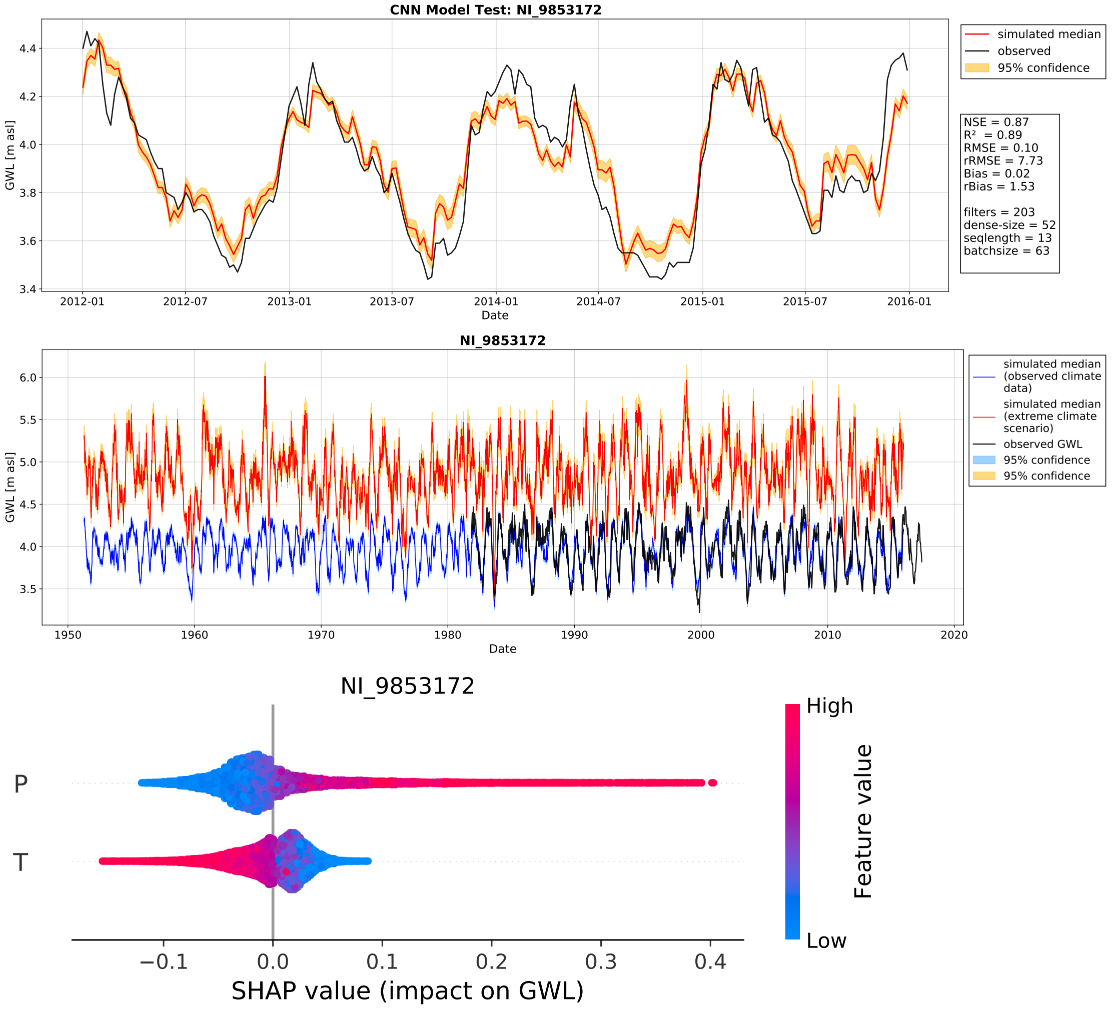

Figure S85: Evaluation of NI_9853172 Model Performance in the past (upper), under extreme climate conditions (middle) and SHAP Summary plot (lower)

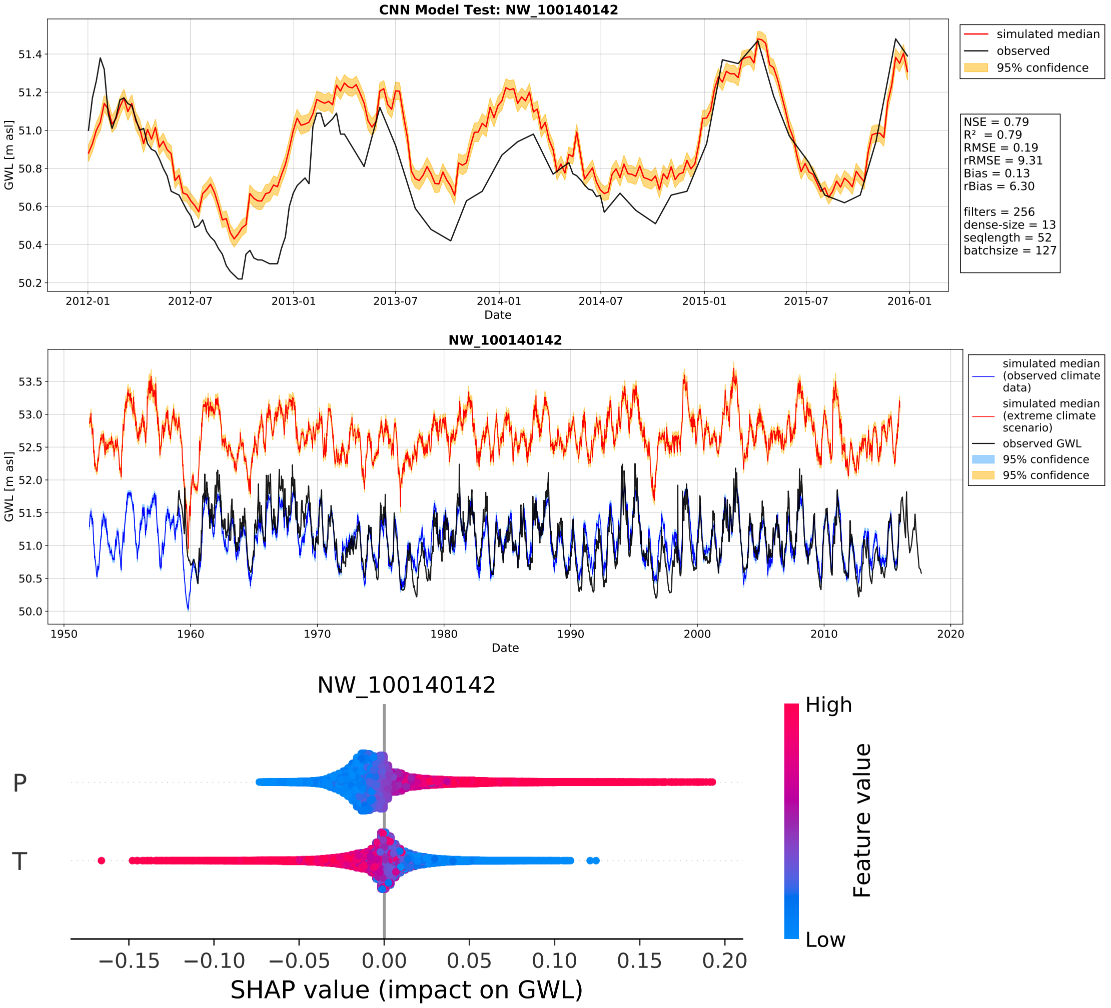

Figure S86: Evaluation of NW_100140142 Model Performance in the past (upper), under extreme climate conditions (middle) and SHAP Summary plot (lower)

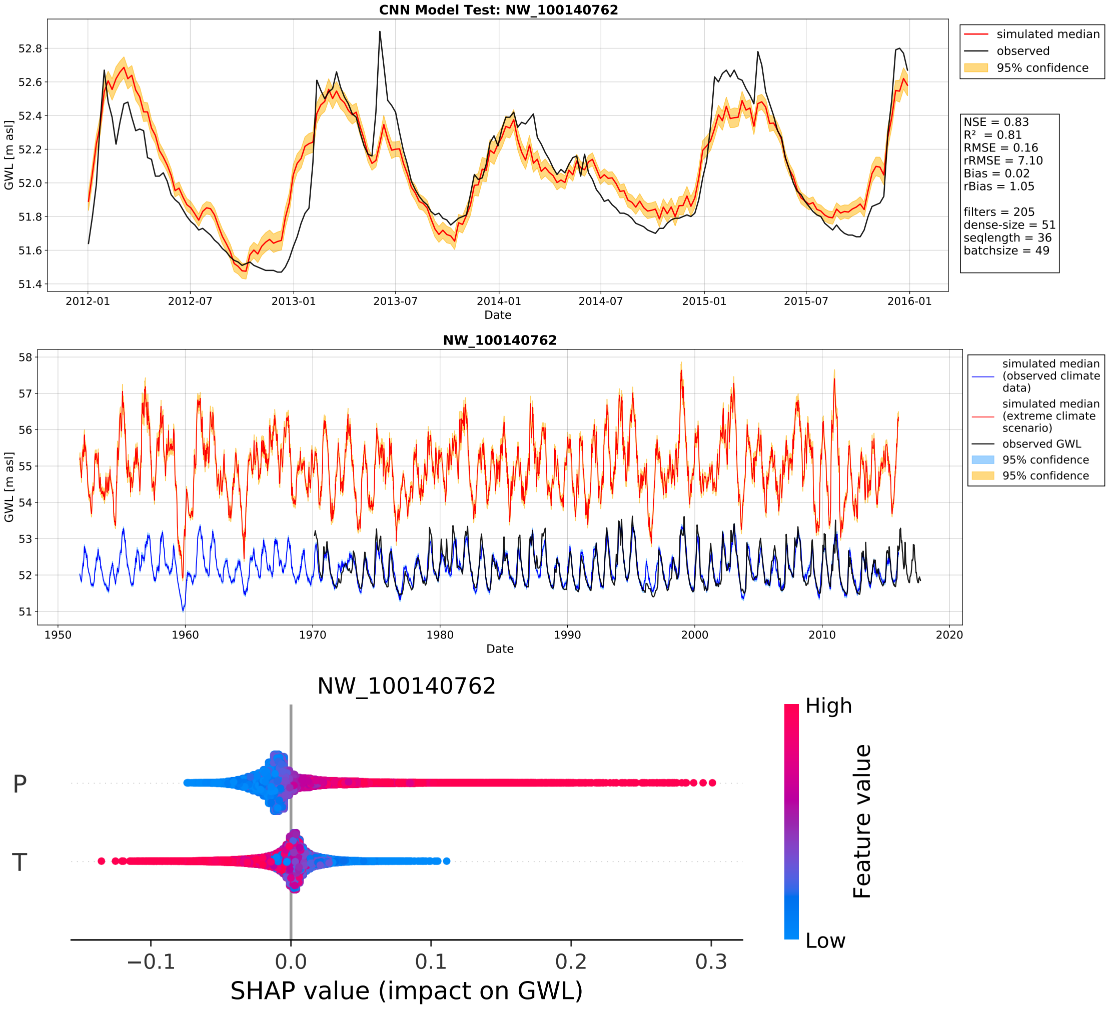

Figure S87: Evaluation of NW_100140762 Model Performance in the past (upper), under extreme climate conditions (middle) and SHAP Summary plot (lower)

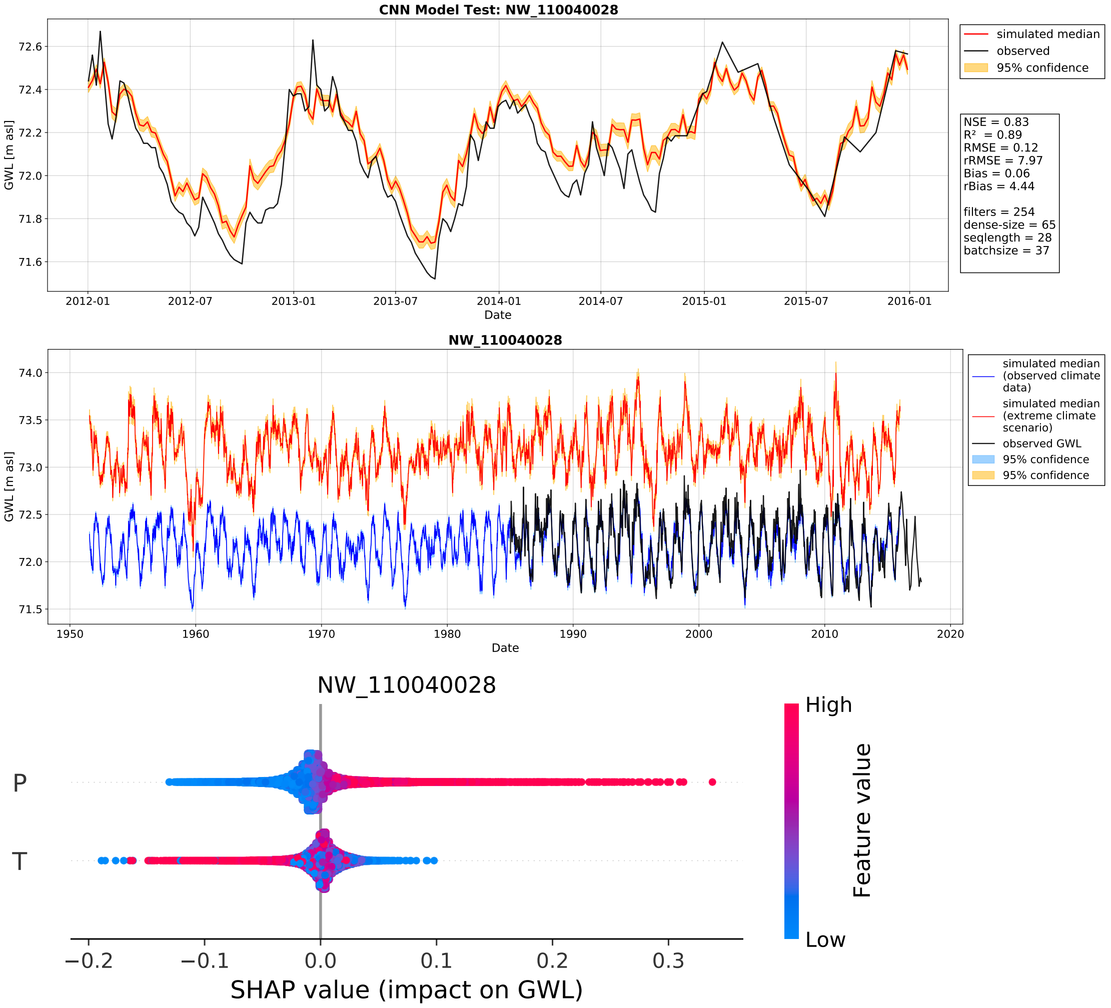

Figure S88: Evaluation of NW_110040028 Model Performance in the past (upper), under extreme climate conditions (middle) and SHAP Summary plot (lower)

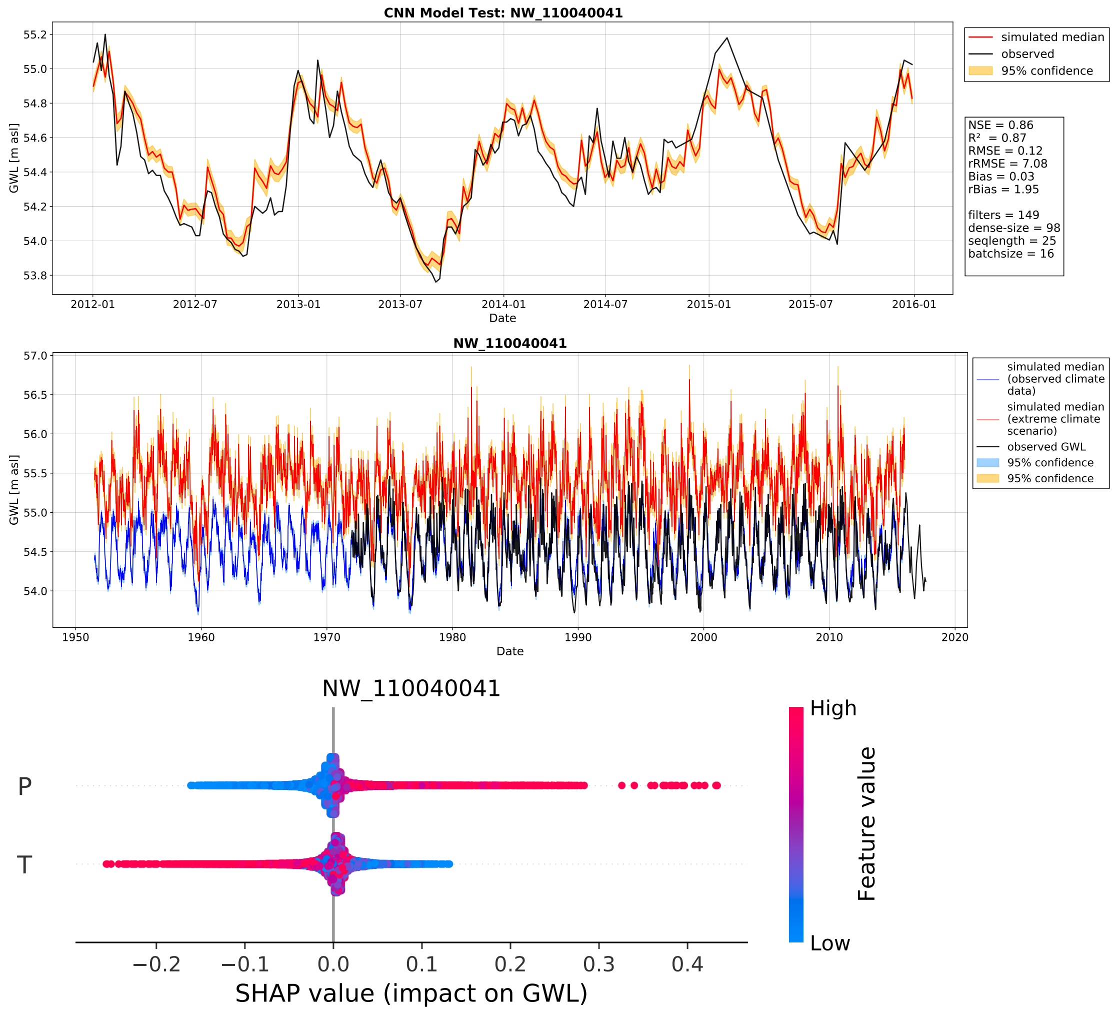

Figure S89: Evaluation of NW_110040041 Model Performance in the past (upper), under extreme climate conditions (middle) and SHAP Summary plot (lower)

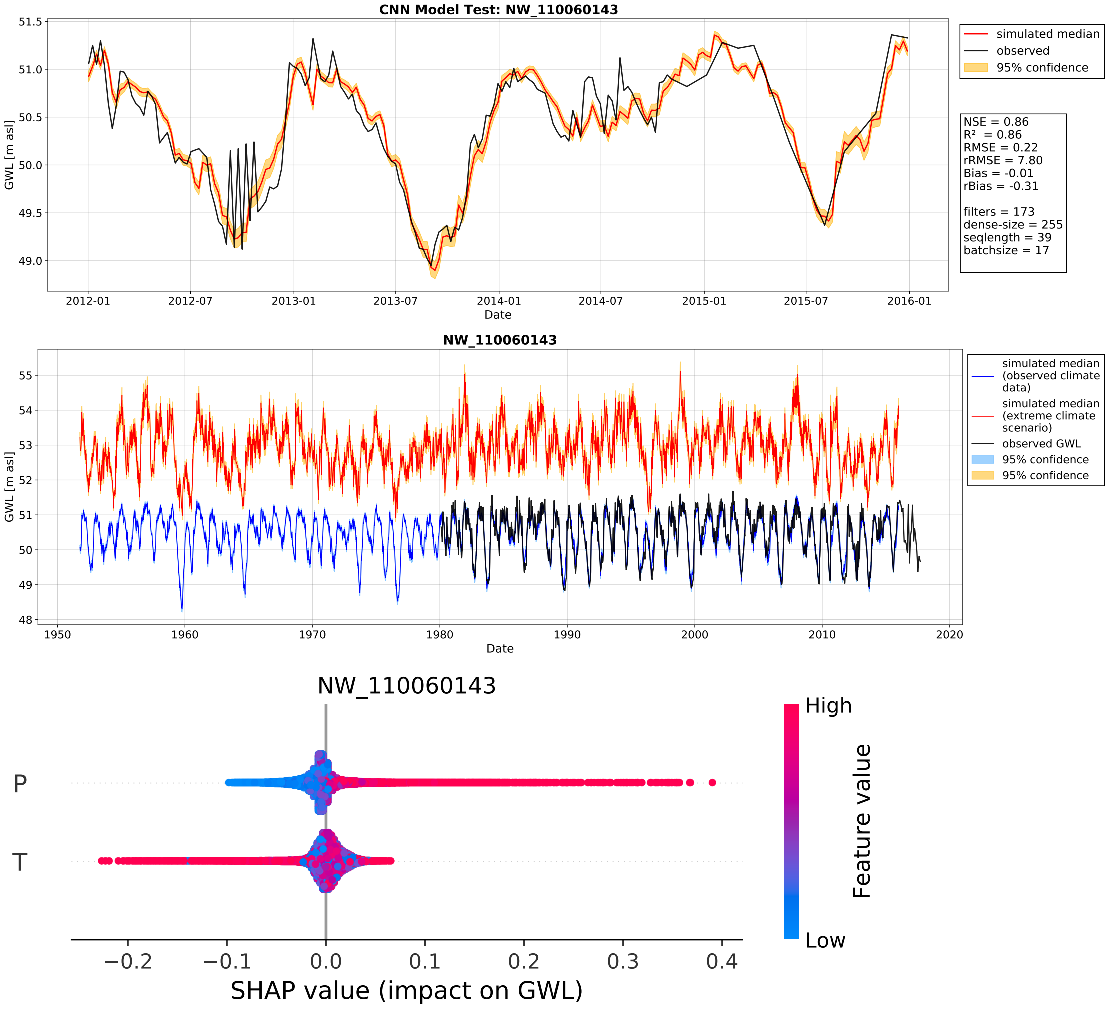

Figure S90: Evaluation of NW_110060143 Model Performance in the past (upper), under extreme climate conditions (middle) and SHAP Summary plot (lower)

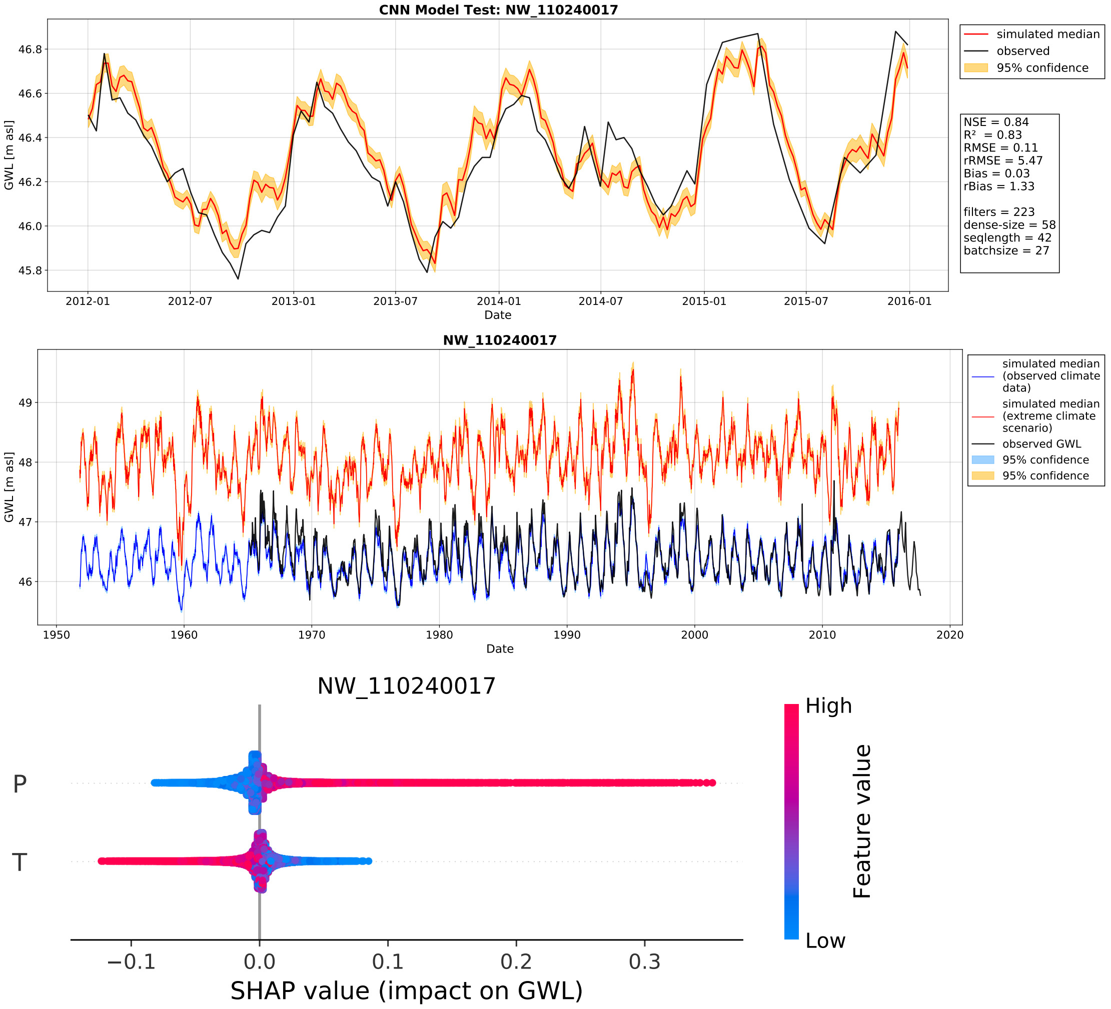

Figure S91: Evaluation of NW_110240017 Model Performance in the past (upper), under extreme climate conditions (middle) and SHAP Summary plot (lower)

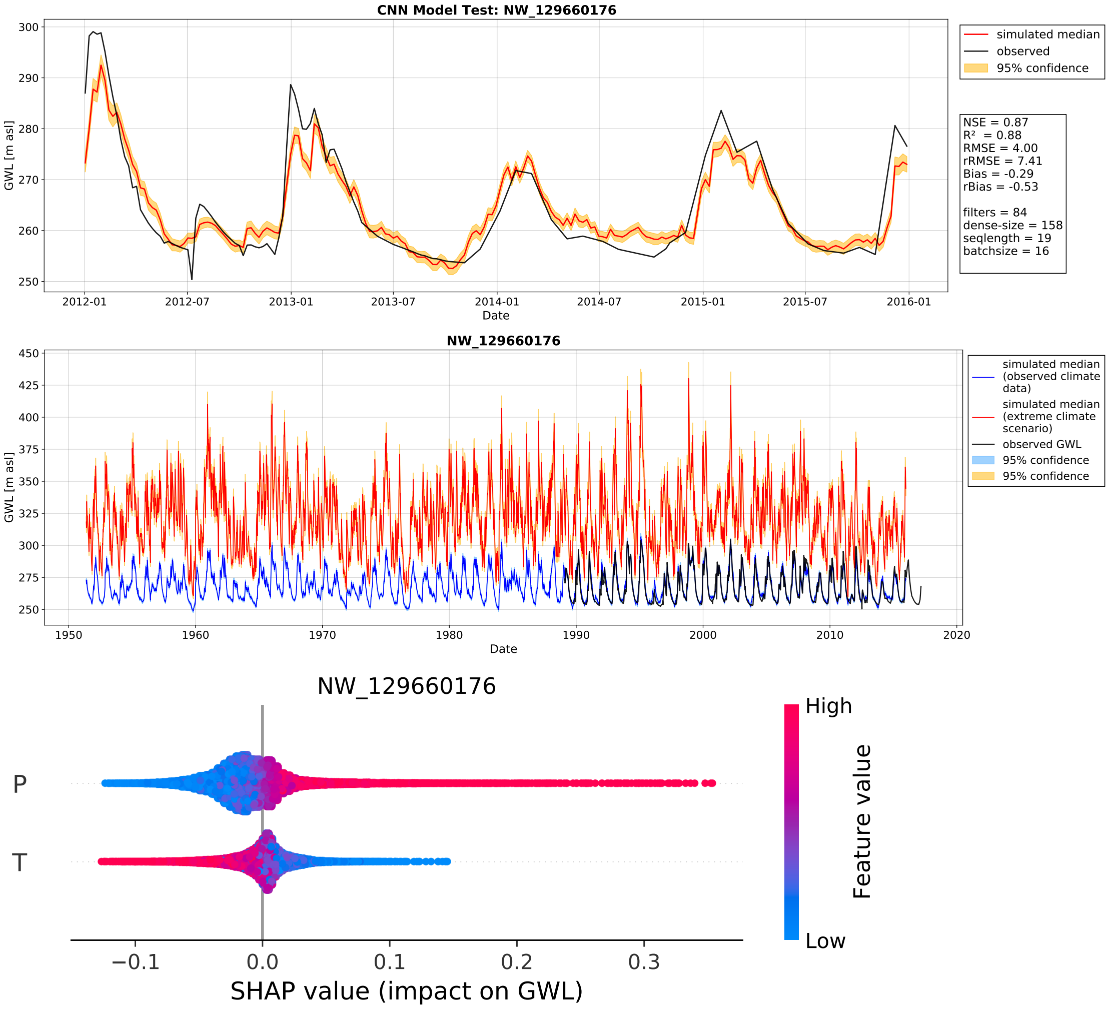

Figure S92: Evaluation of NW_129660176 Model Performance in the past (upper), under extreme climate conditions (middle) and SHAP Summary plot (lower)

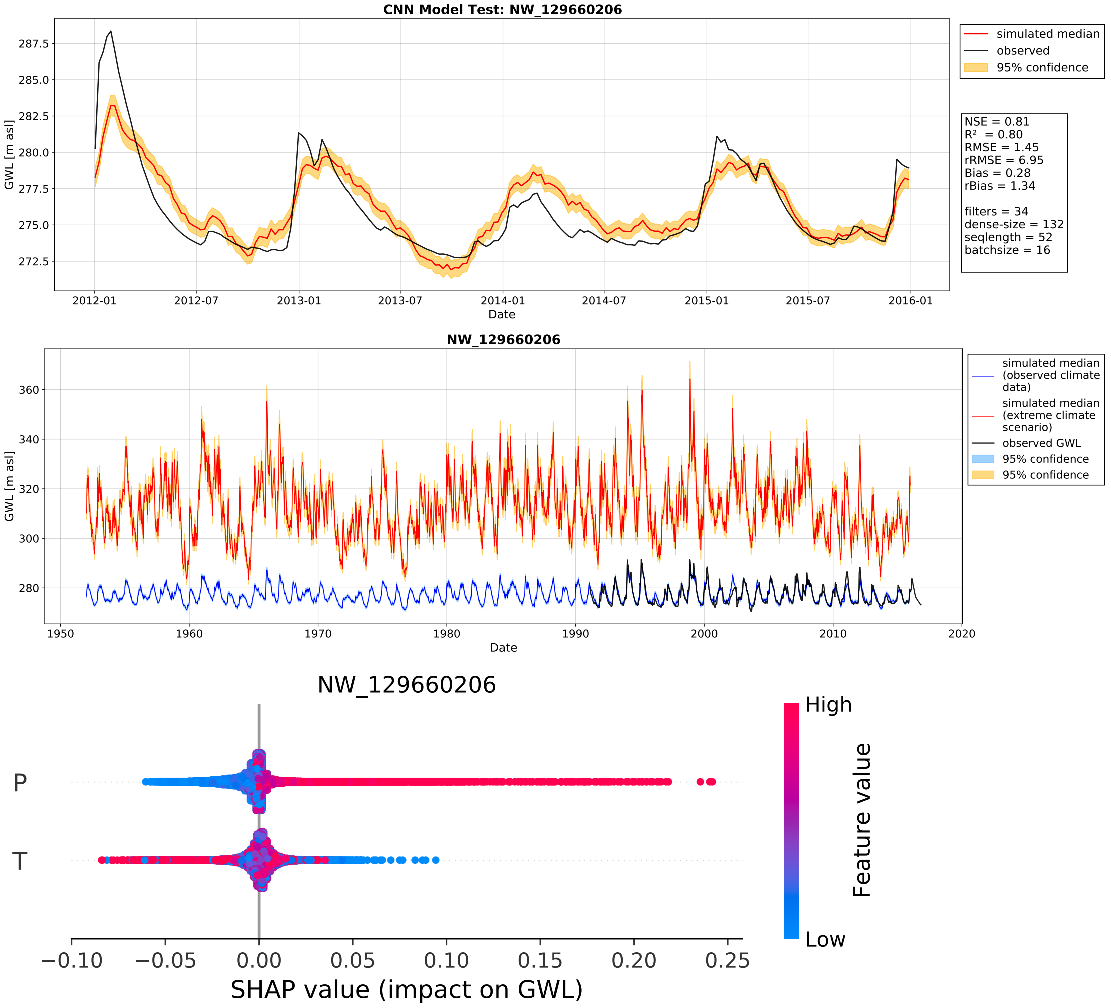

Figure S93: Evaluation of NW_129660206 Model Performance in the past (upper), under extreme climate conditions (middle) and SHAP Summary plot (lower)

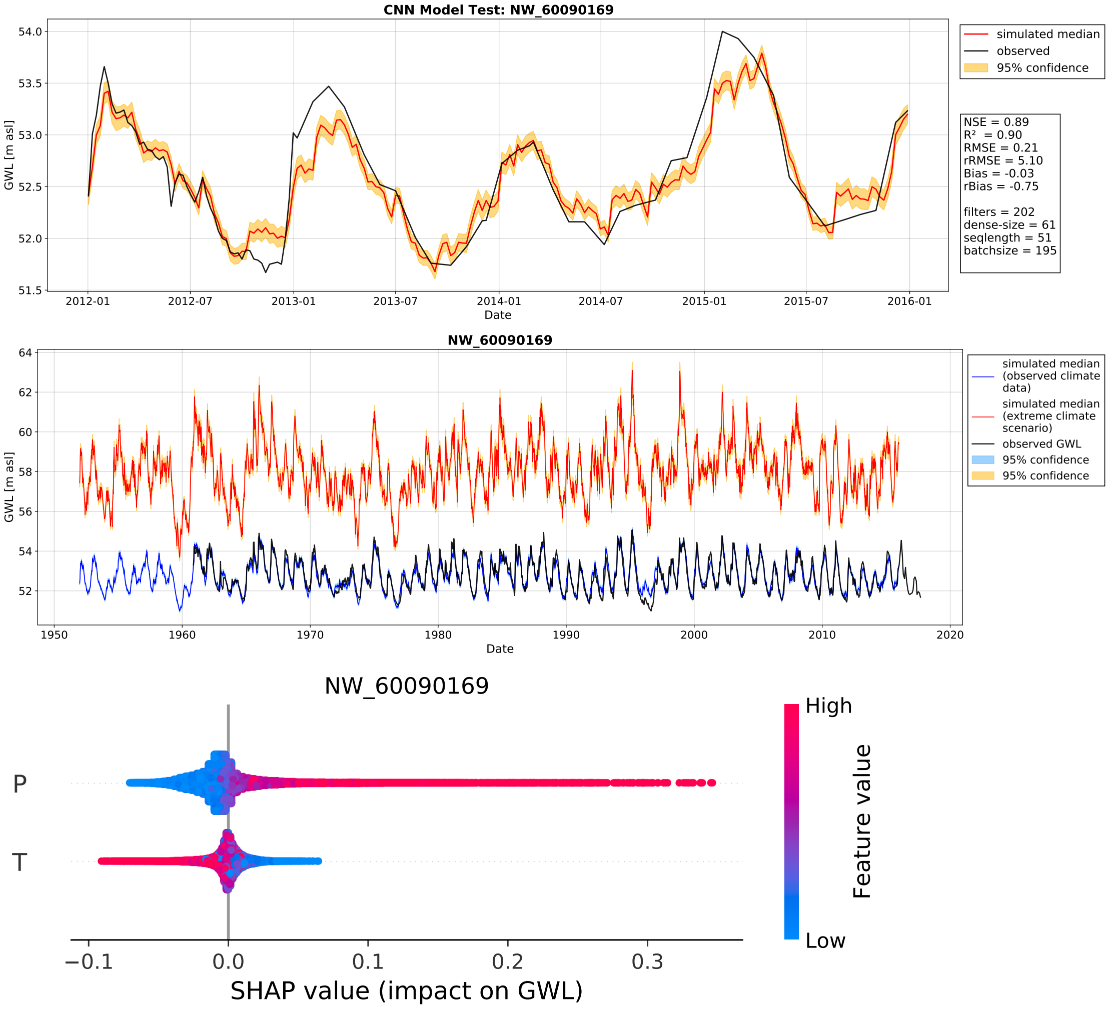

Figure S94: Evaluation of NW_60090169 Model Performance in the past (upper), under extreme climate conditions (middle) and SHAP Summary plot (lower)

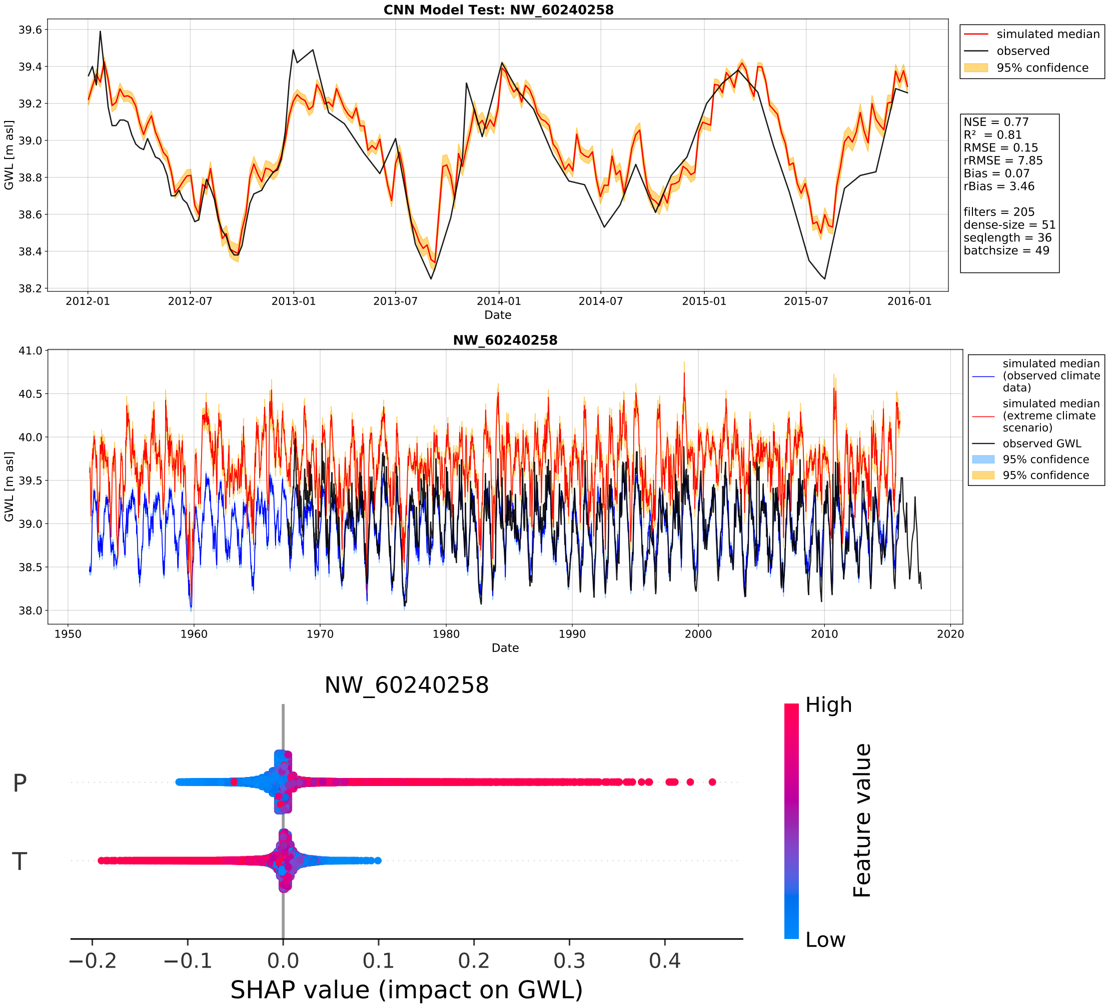

Figure S95: Evaluation of NW_60240258 Model Performance in the past (upper), under extreme climate conditions (middle) and SHAP Summary plot (lower)

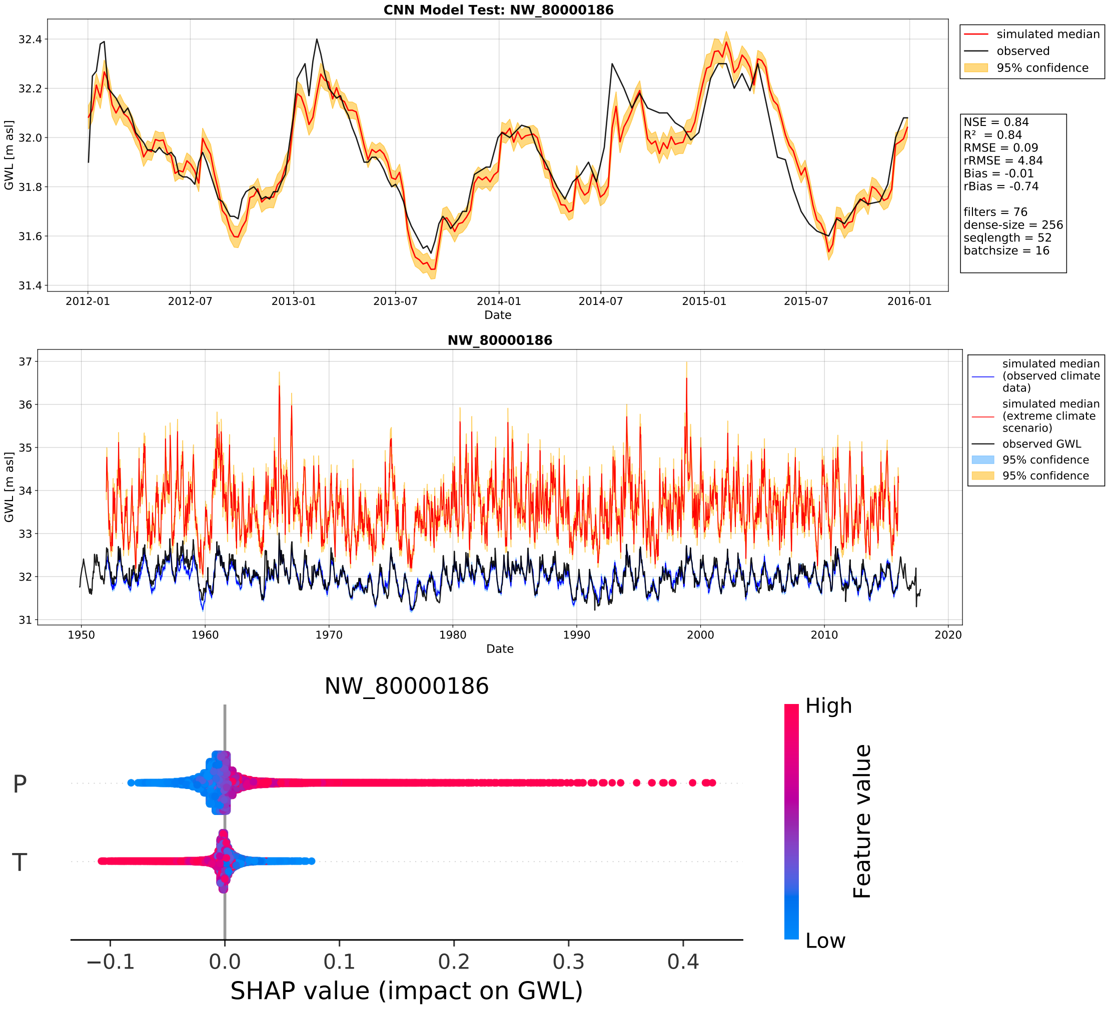

Figure S96: Evaluation of NW_80000186 Model Performance in the past (upper), under extreme climate conditions (middle) and SHAP Summary plot (lower)

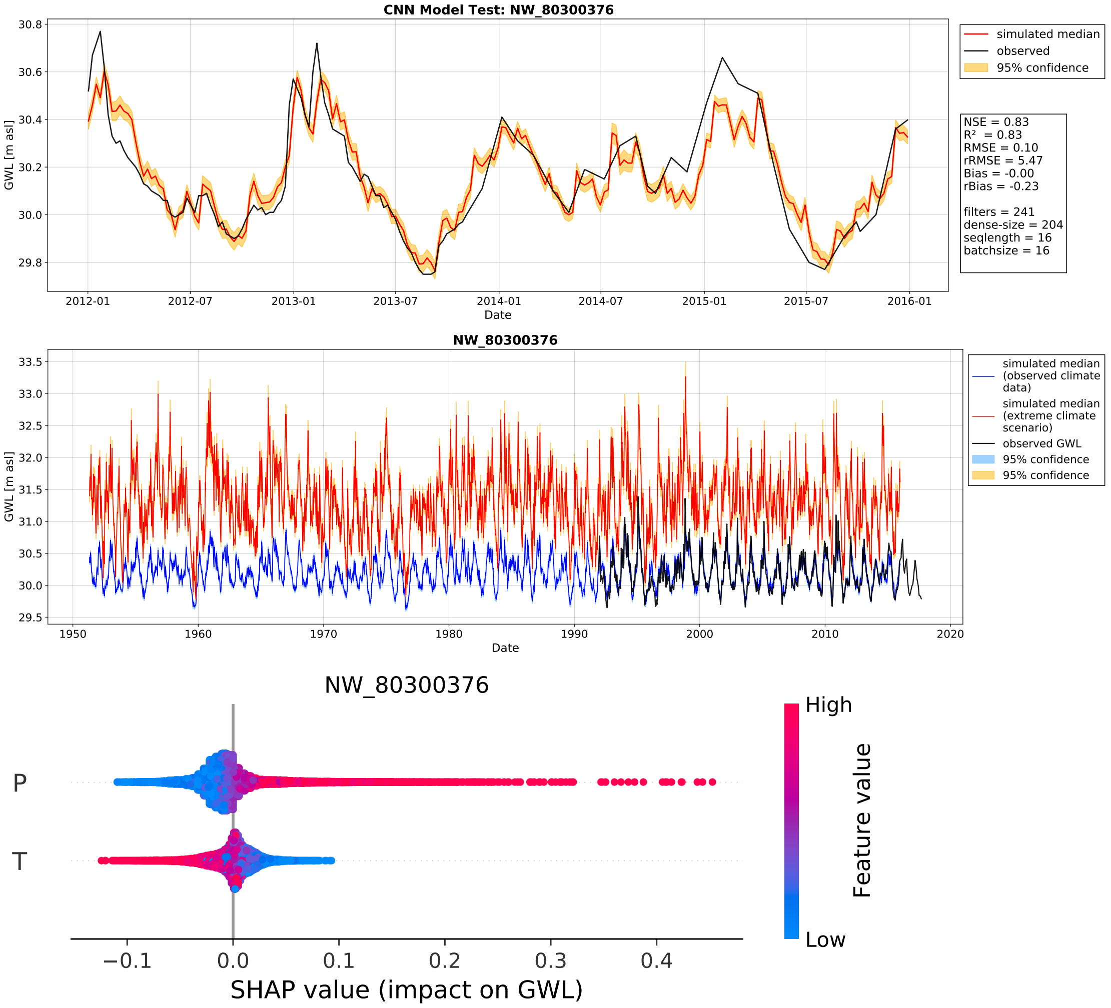

Figure S97: Evaluation of NW_80300376 Model Performance in the past (upper), under extreme climate conditions (middle) and SHAP Summary plot (lower)

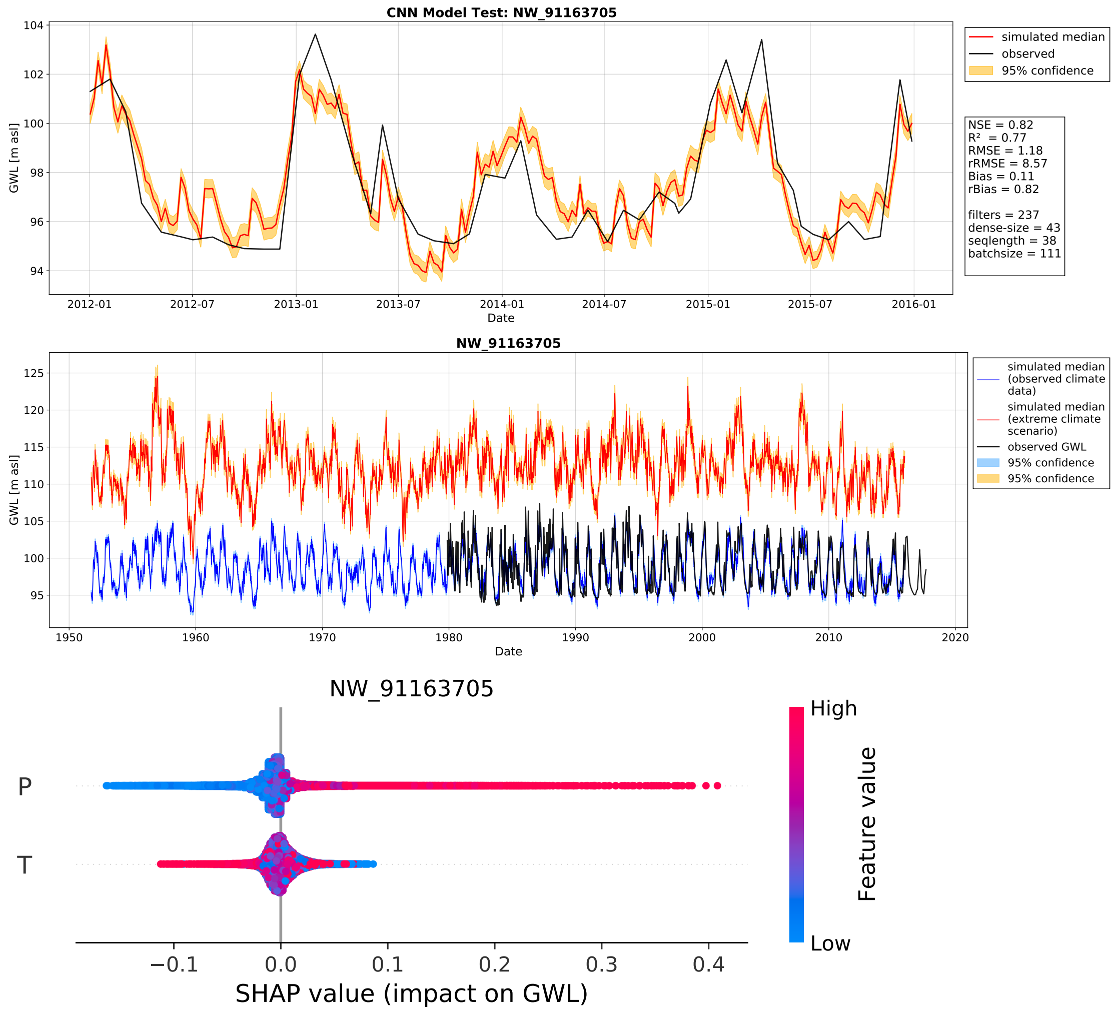

Figure S98: Evaluation of NW_91163705 Model Performance in the past (upper), under extreme climate conditions (middle) and SHAP Summary plot (lower)

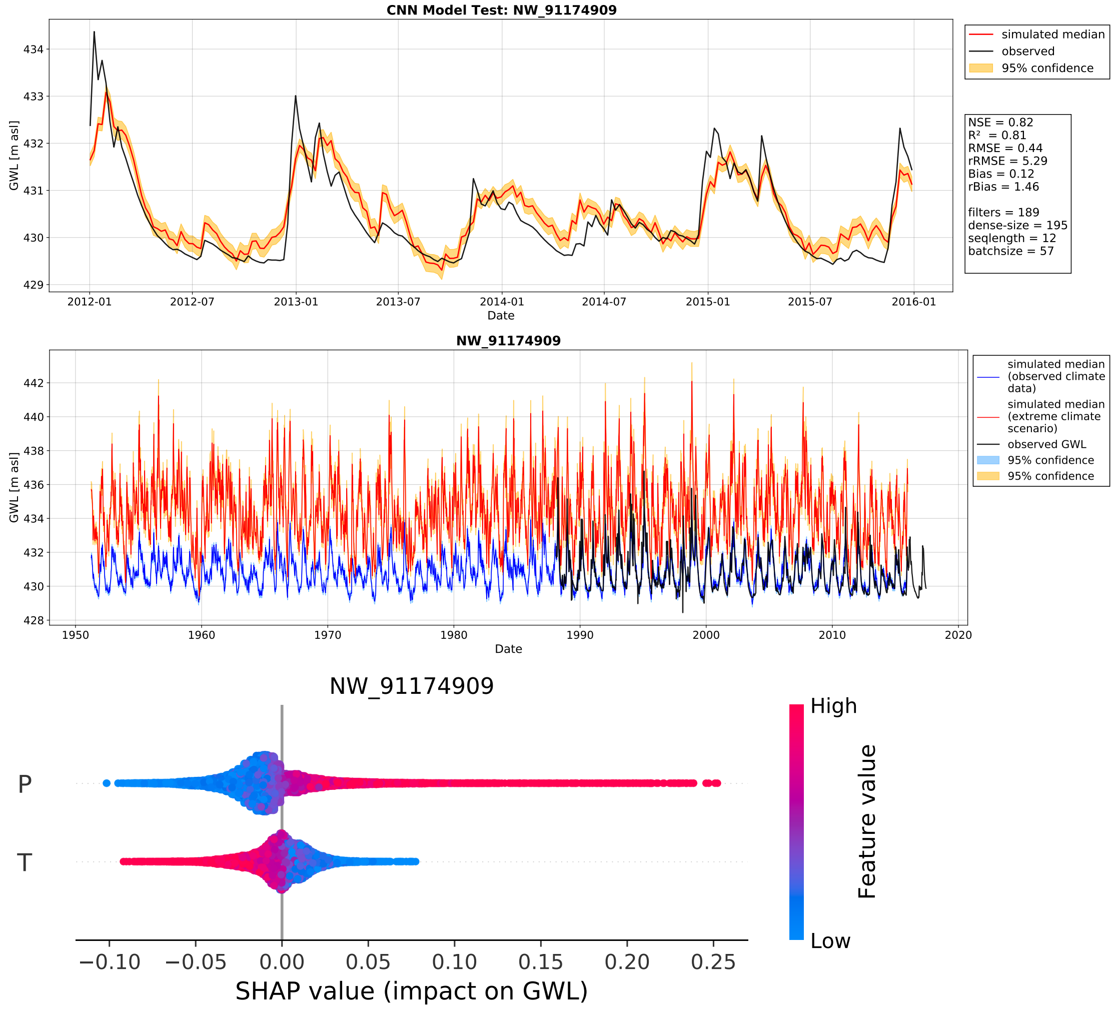

Figure S99: Evaluation of NW_91174909 Model Performance in the past (upper), under extreme climate conditions (middle) and SHAP Summary plot (lower)

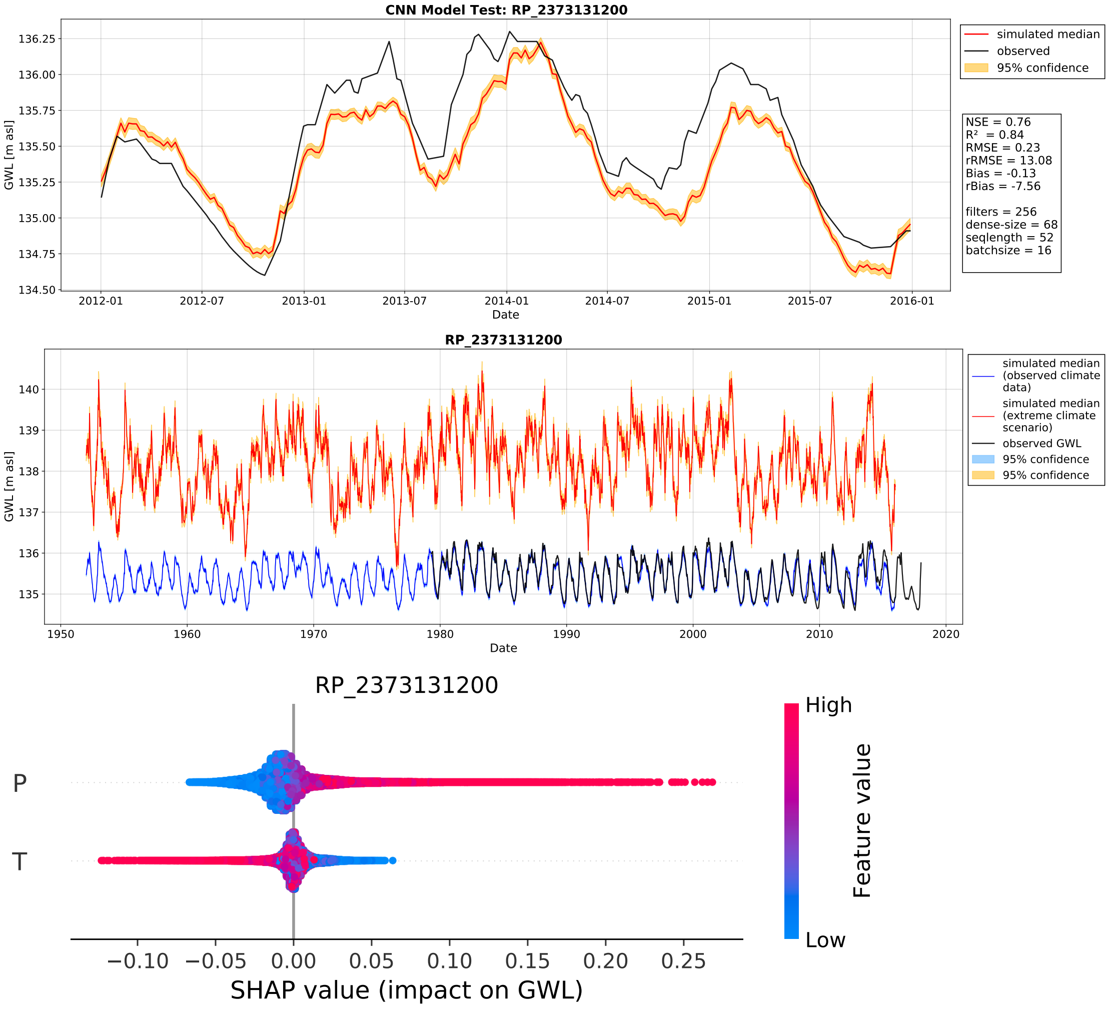

Figure S100: Evaluation of RP_2373131200 Model Performance in the past (upper), under extreme climate conditions (middle) and SHAP Summary plot (lower)

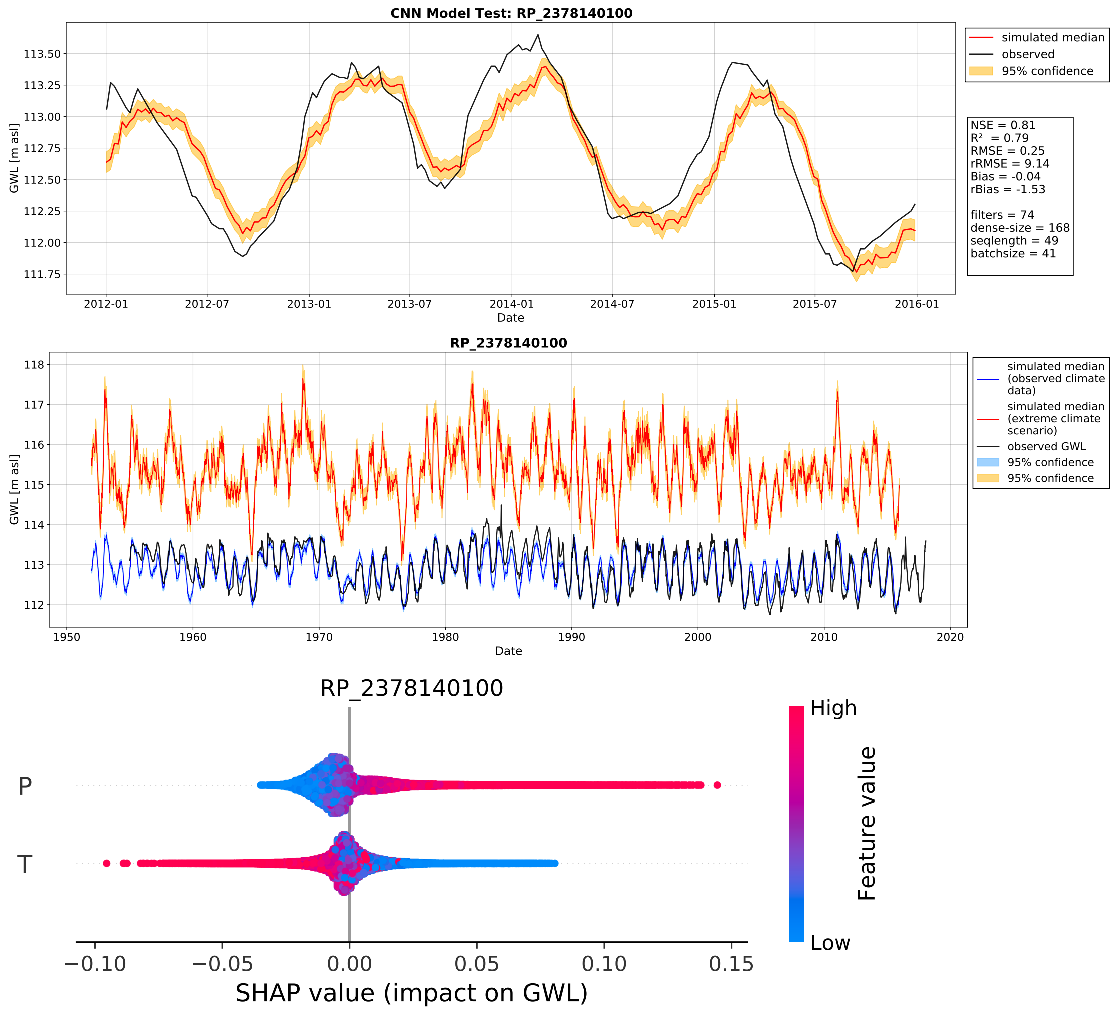

Figure S101: Evaluation of RP_2378140100 Model Performance in the past (upper), under extreme climate conditions (middle) and SHAP Summary plot (lower)

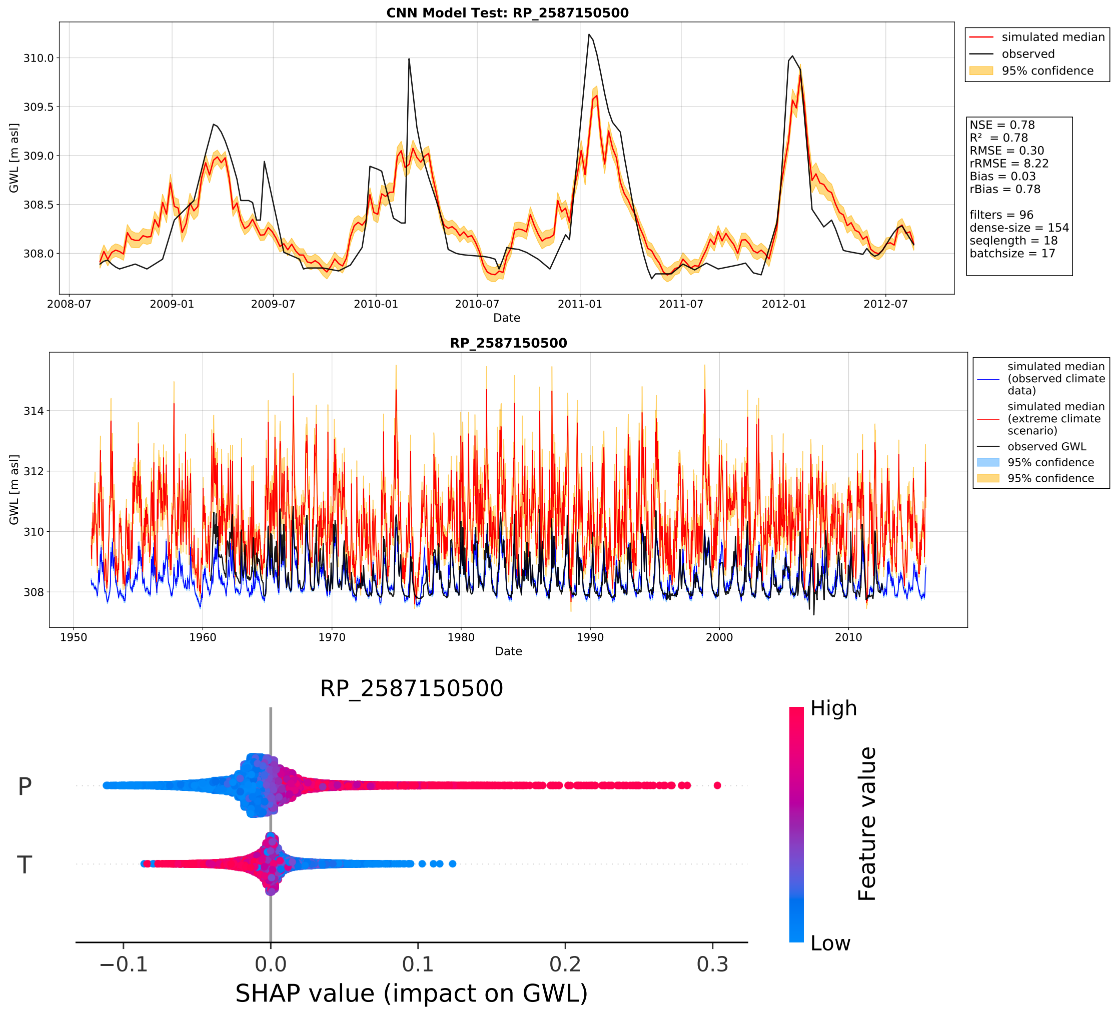

Figure S102: Evaluation of RP_2587150500 Model Performance in the past (upper), under extreme climate conditions (middle) and SHAP Summary plot (lower)

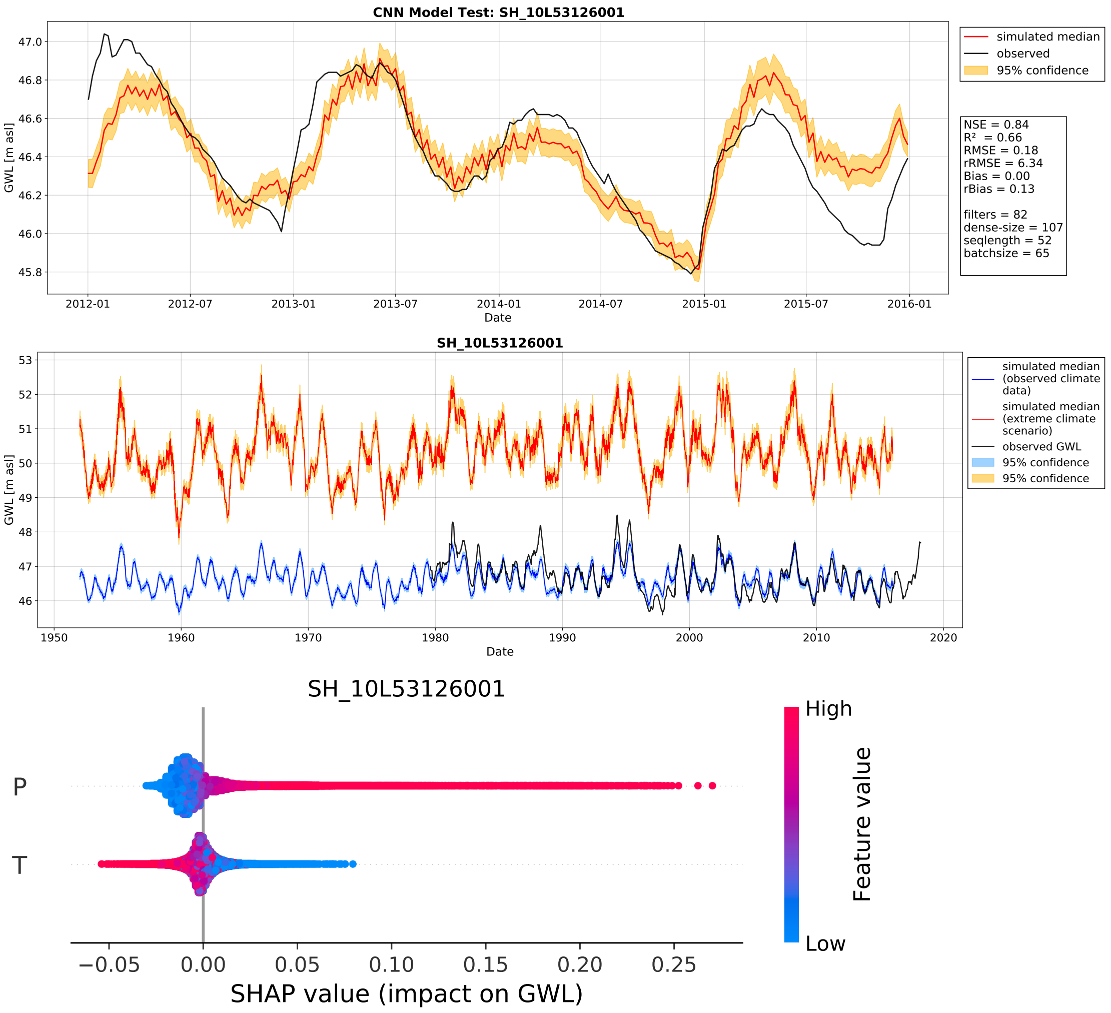

Figure S103: Evaluation of SH_10L53126001 Model Performance in the past (upper), under extreme climate conditions (middle) and SHAP Summary plot (lower)

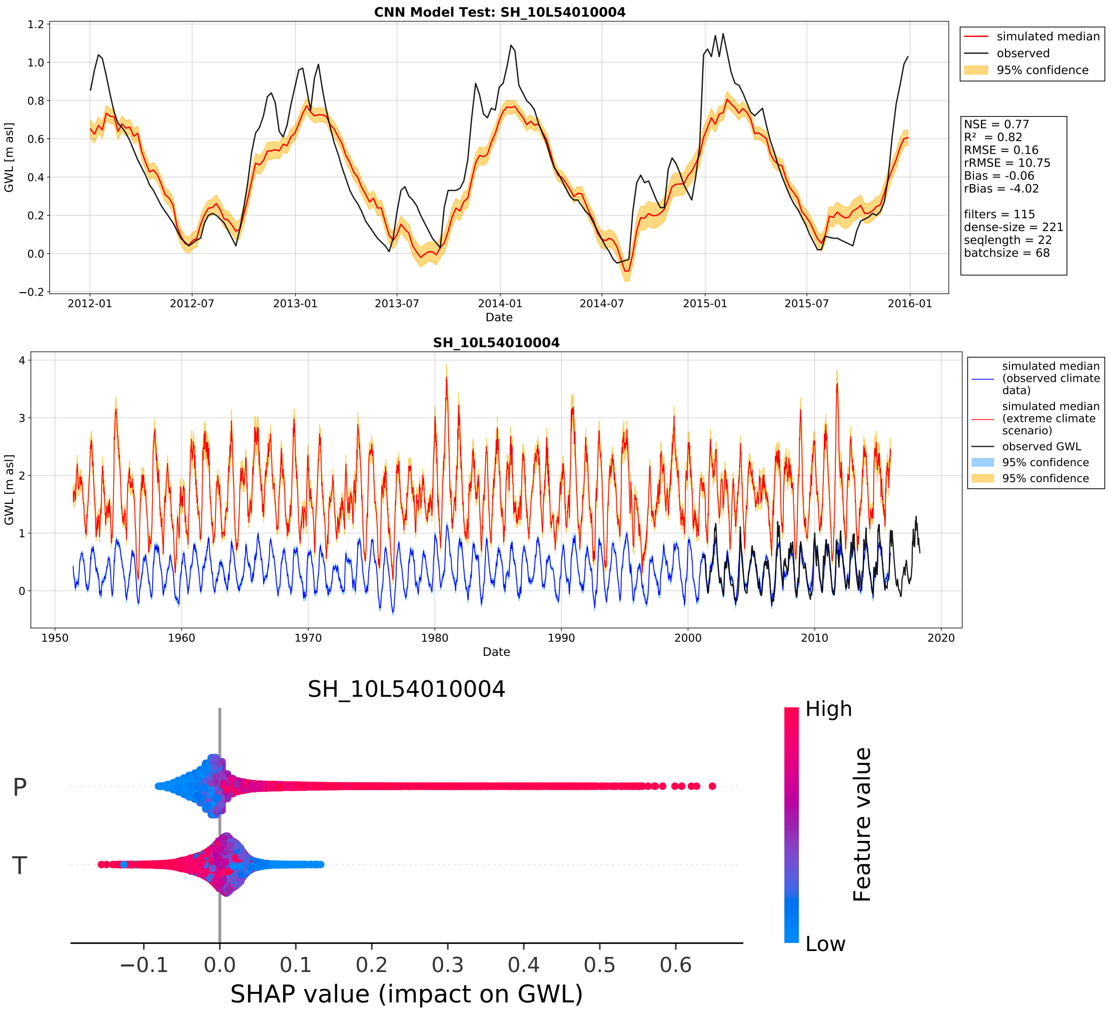

Figure S104: Evaluation of SH_10L54010004 Model Performance in the past (upper), under extreme climate conditions (middle) and SHAP Summary plot (lower)

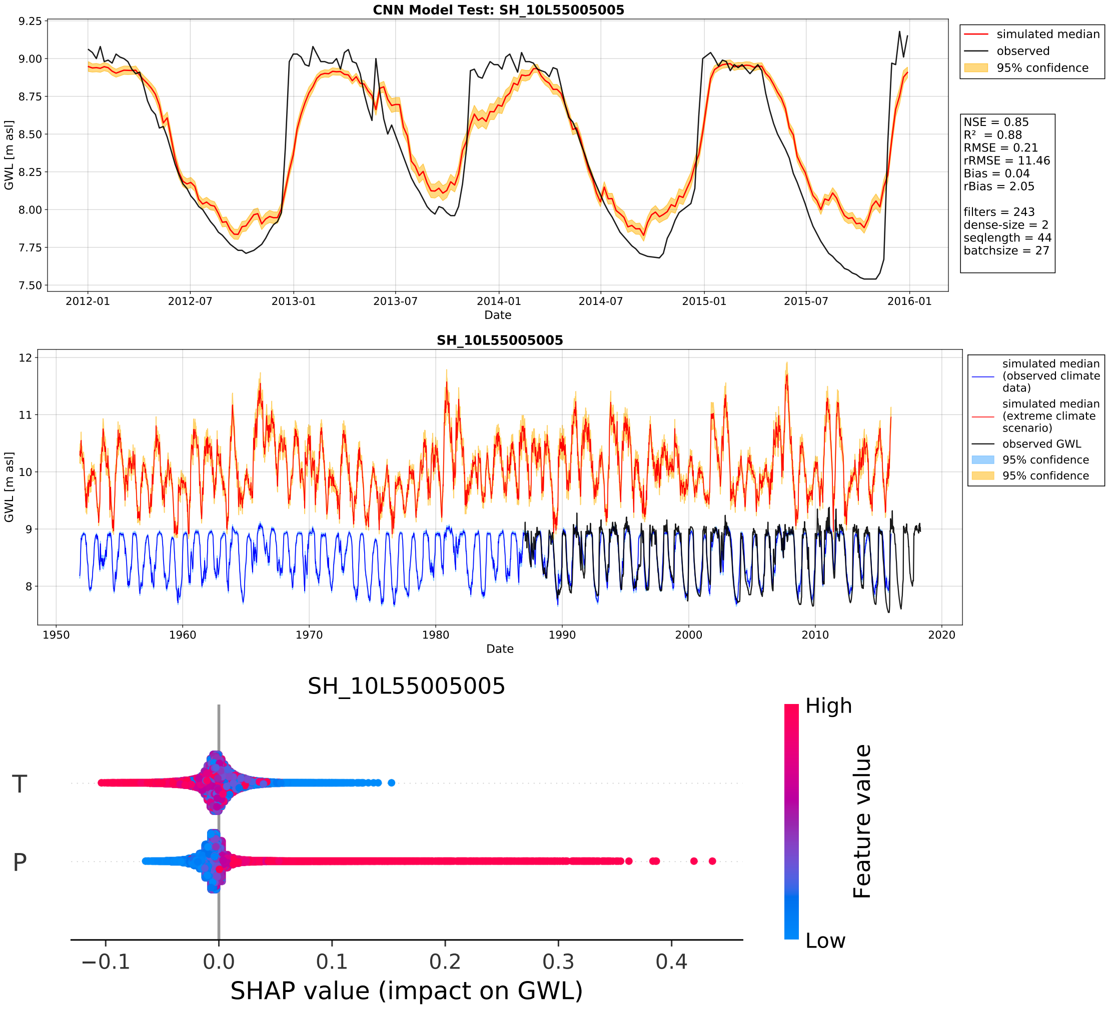

Figure S105: Evaluation of SH_10L55005005 Model Performance in the past (upper), under extreme climate conditions (middle) and SHAP Summary plot (lower)

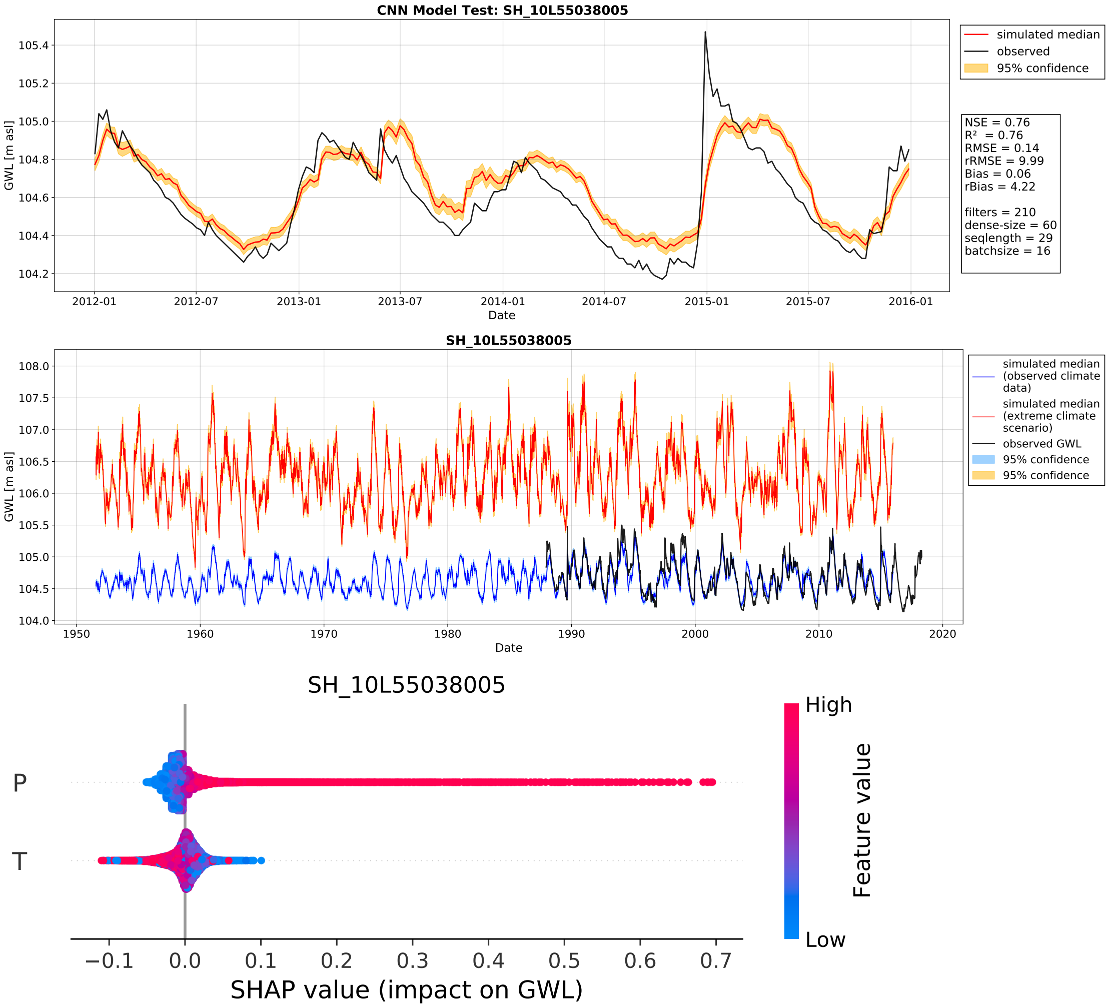

Figure S106: Evaluation of SH_10L55038005 Model Performance in the past (upper), under extreme climate conditions (middle) and SHAP Summary plot (lower)

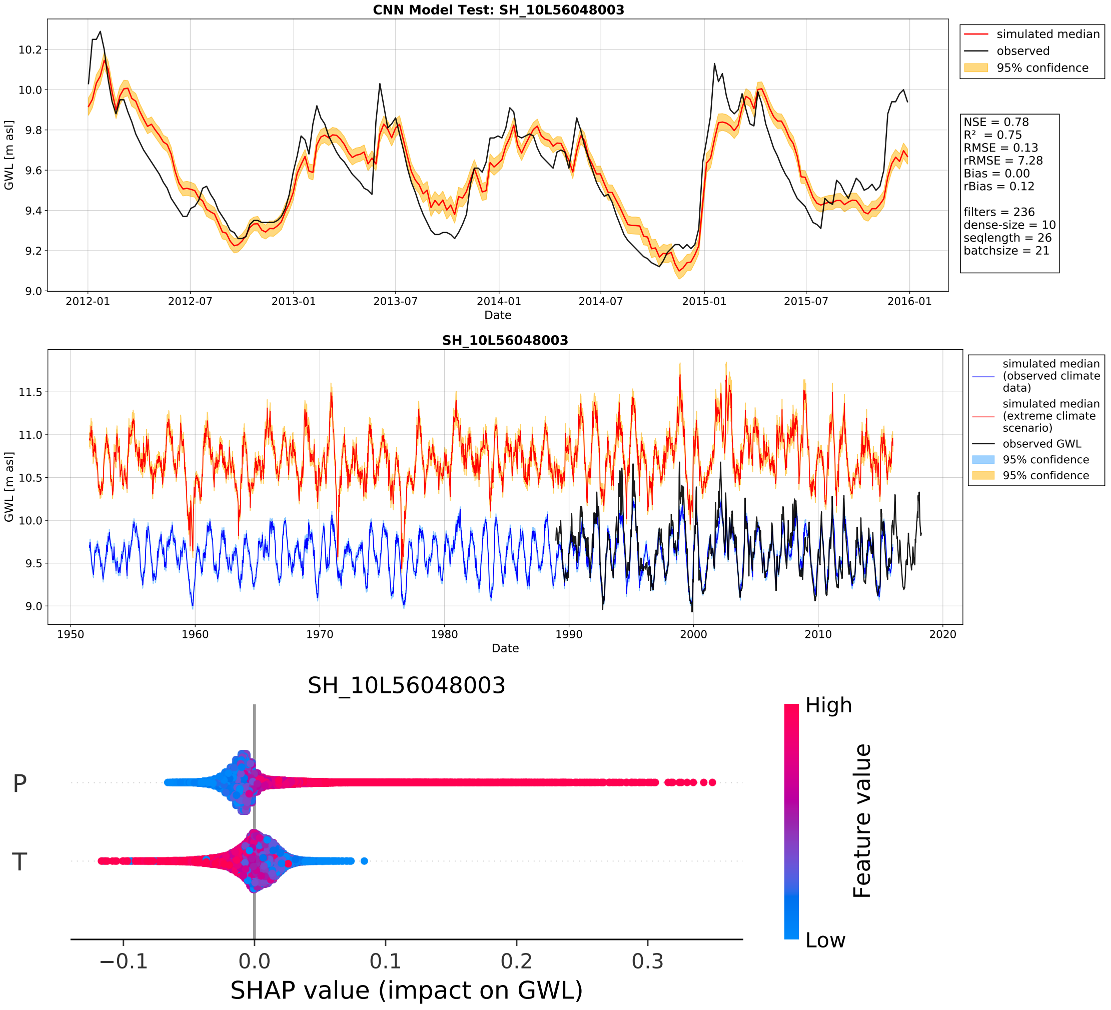

Figure S107: Evaluation of SH_10L56048003 Model Performance in the past (upper), under extreme climate conditions (middle) and SHAP Summary plot (lower)

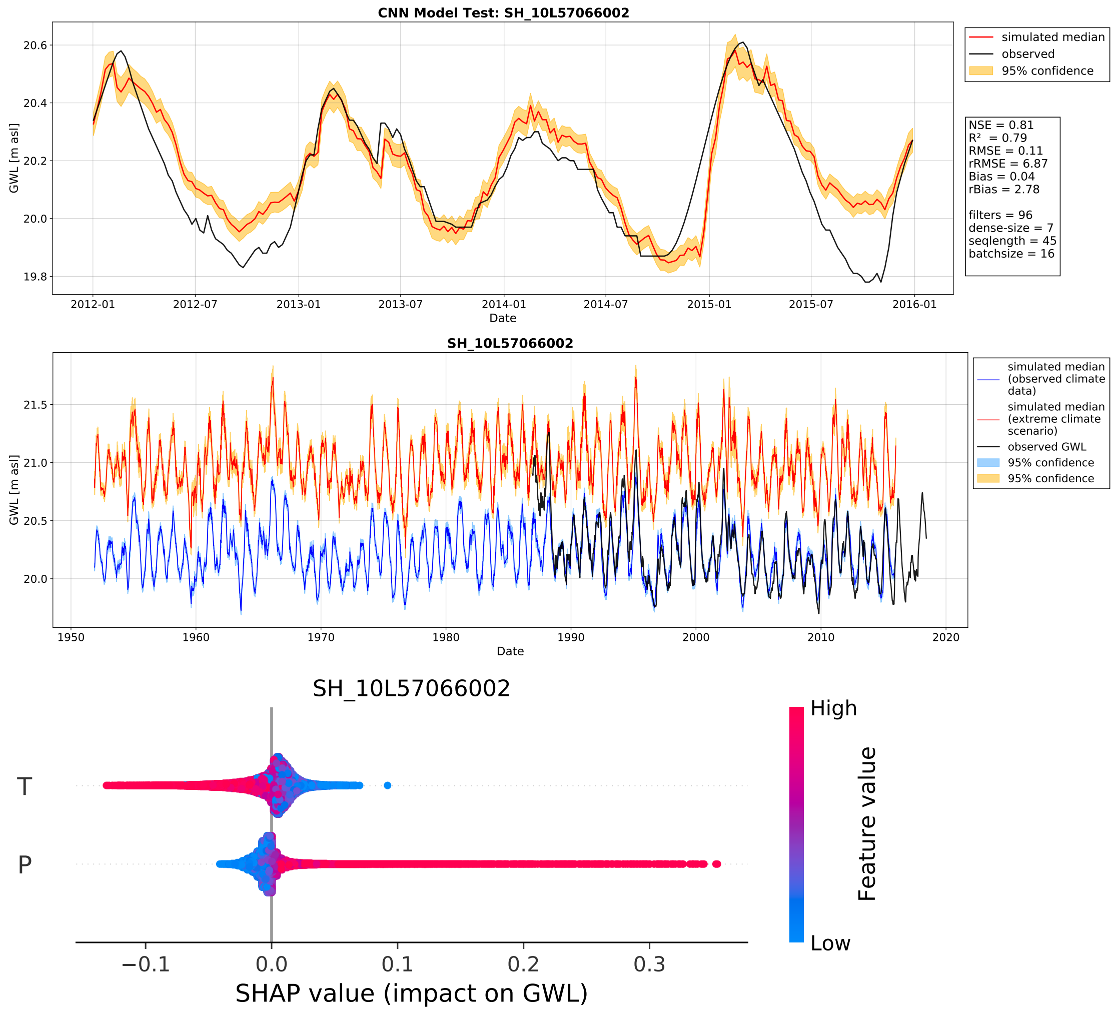

Figure S108: Evaluation of SH_10L57066002 Model Performance in the past (upper), under extreme climate conditions (middle) and SHAP Summary plot (lower)

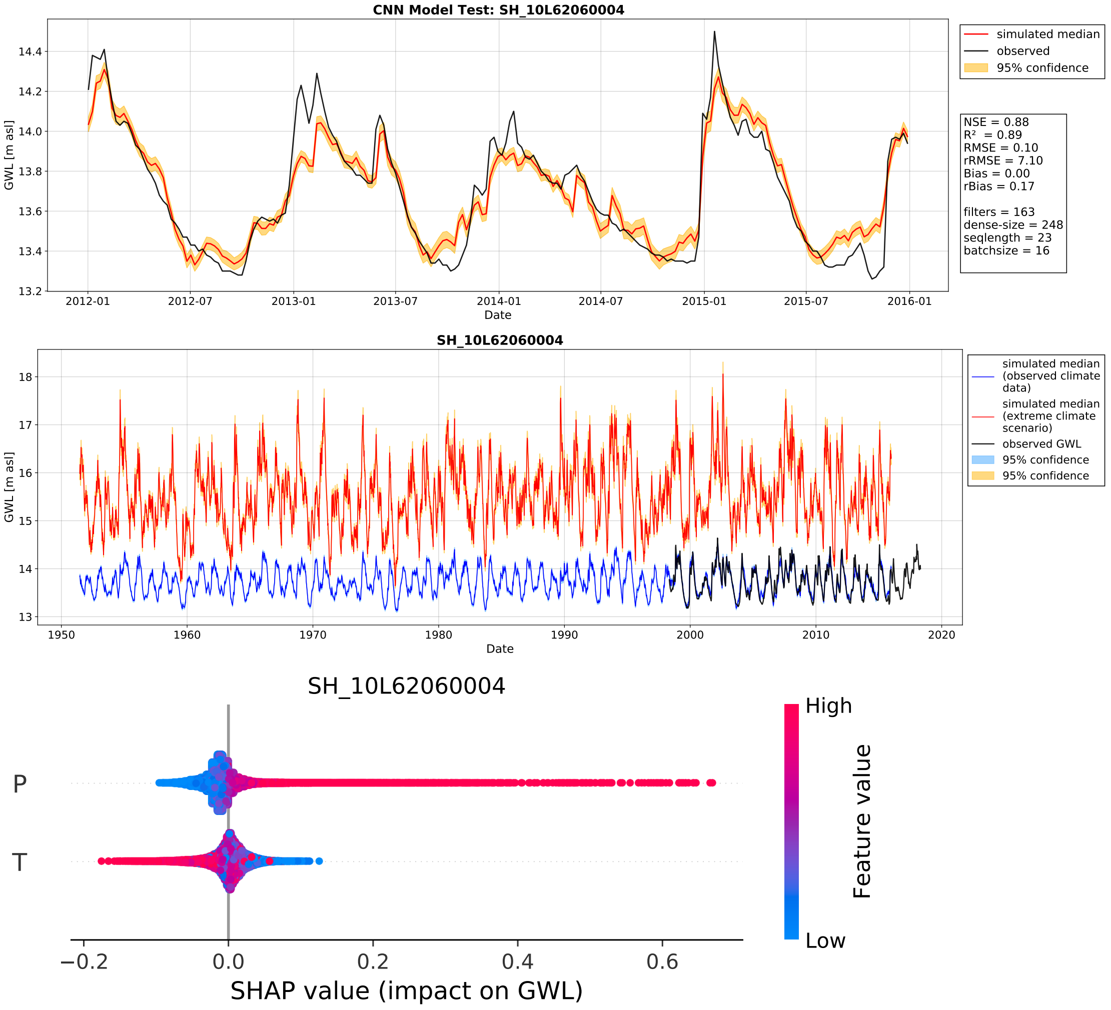

Figure S109: Evaluation of SH_10L62060004 Model Performance in the past (upper), under extreme climate conditions (middle) and SHAP Summary plot (lower)

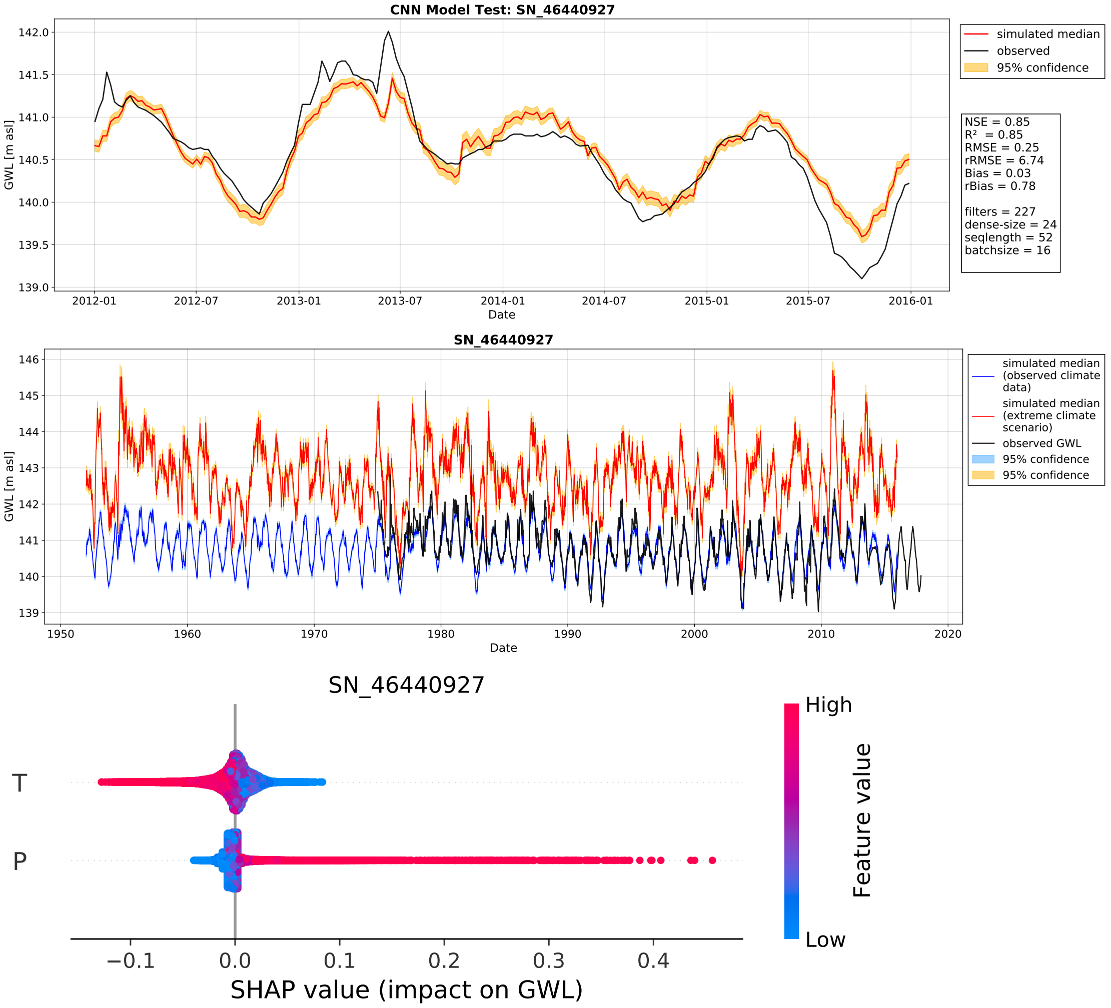

Figure S110: Evaluation of SN_46440927 Model Performance in the past (upper), under extreme climate conditions (middle) and SHAP Summary plot (lower)

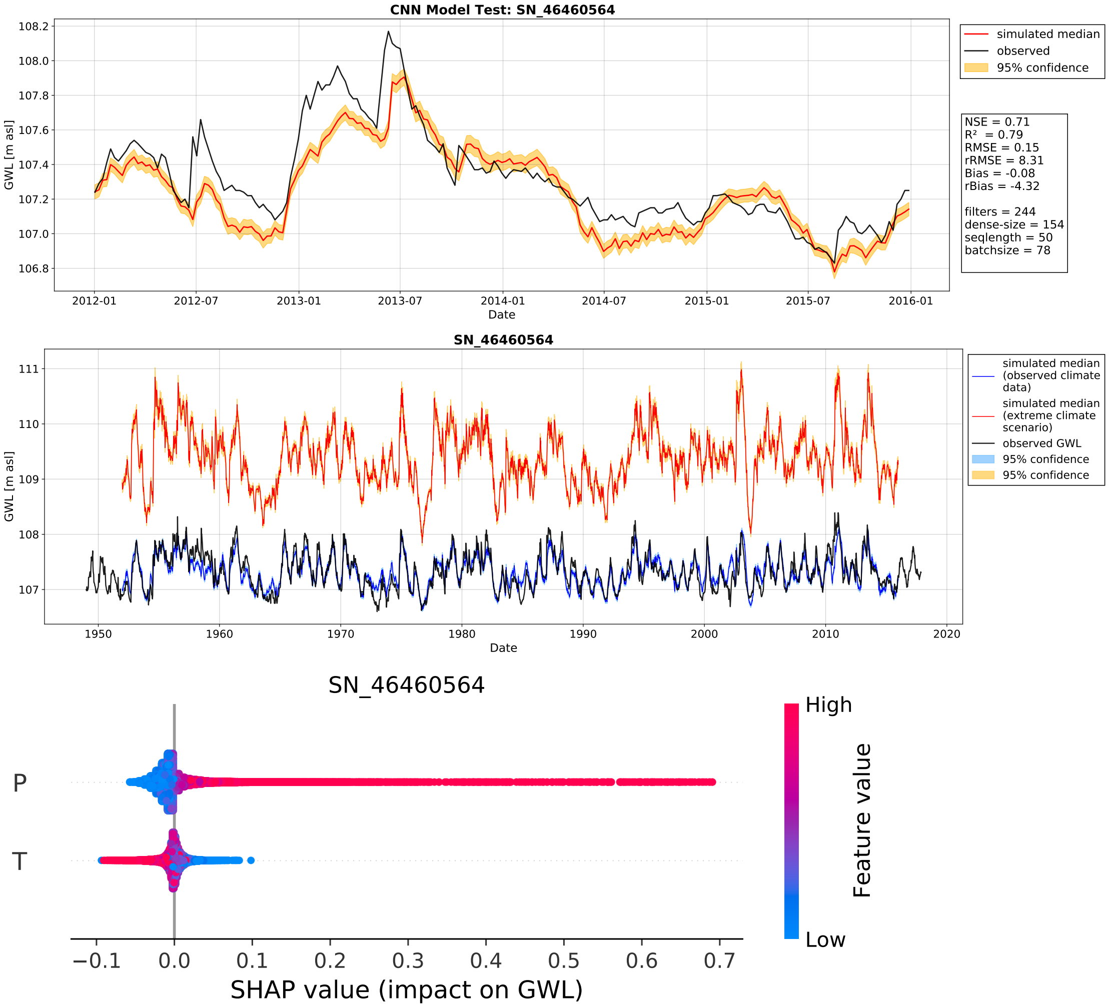

Figure S111: Evaluation of SN_46460564 Model Performance in the past (upper), under extreme climate conditions (middle) and SHAP Summary plot (lower)

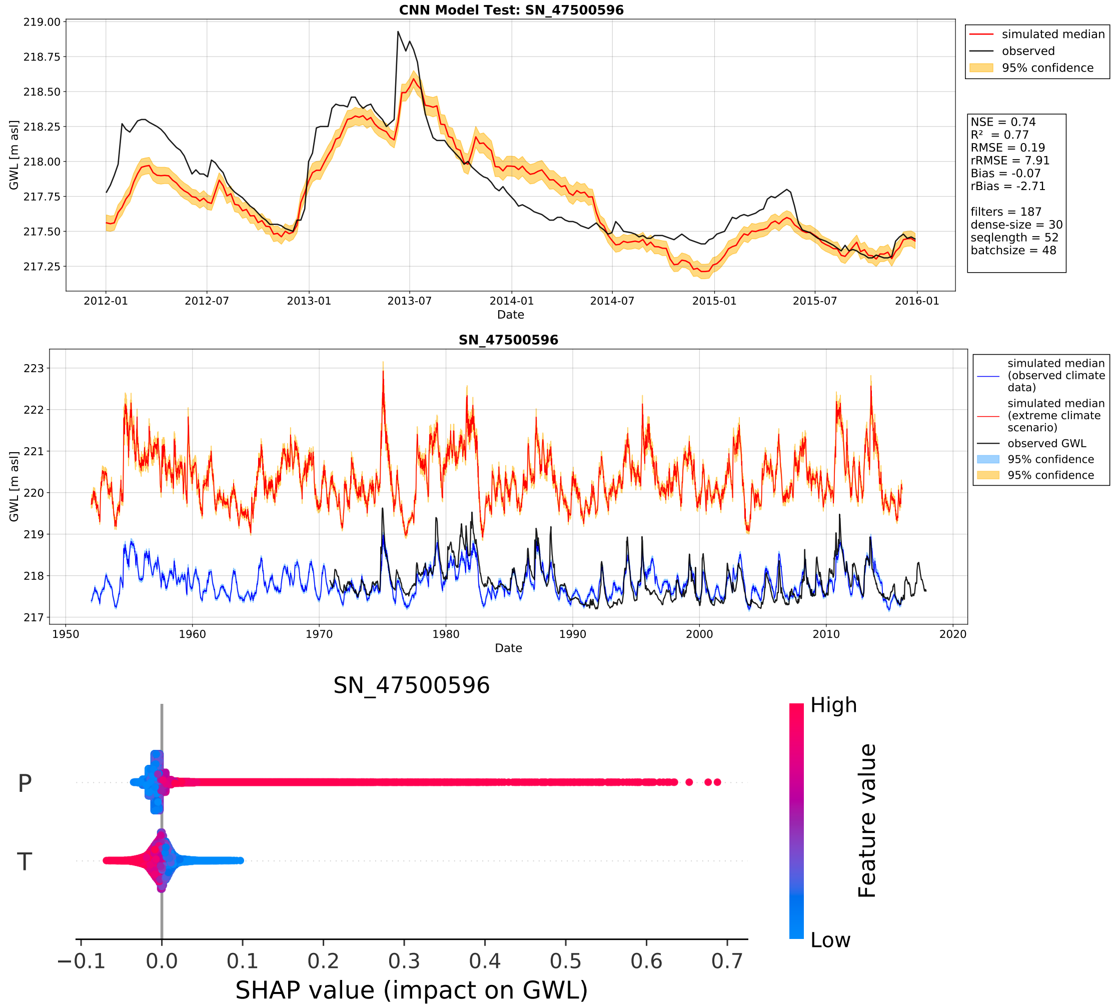

Figure S112: Evaluation of SN_47500596 Model Performance in the past (upper), under extreme climate conditions (middle) and SHAP Summary plot (lower)

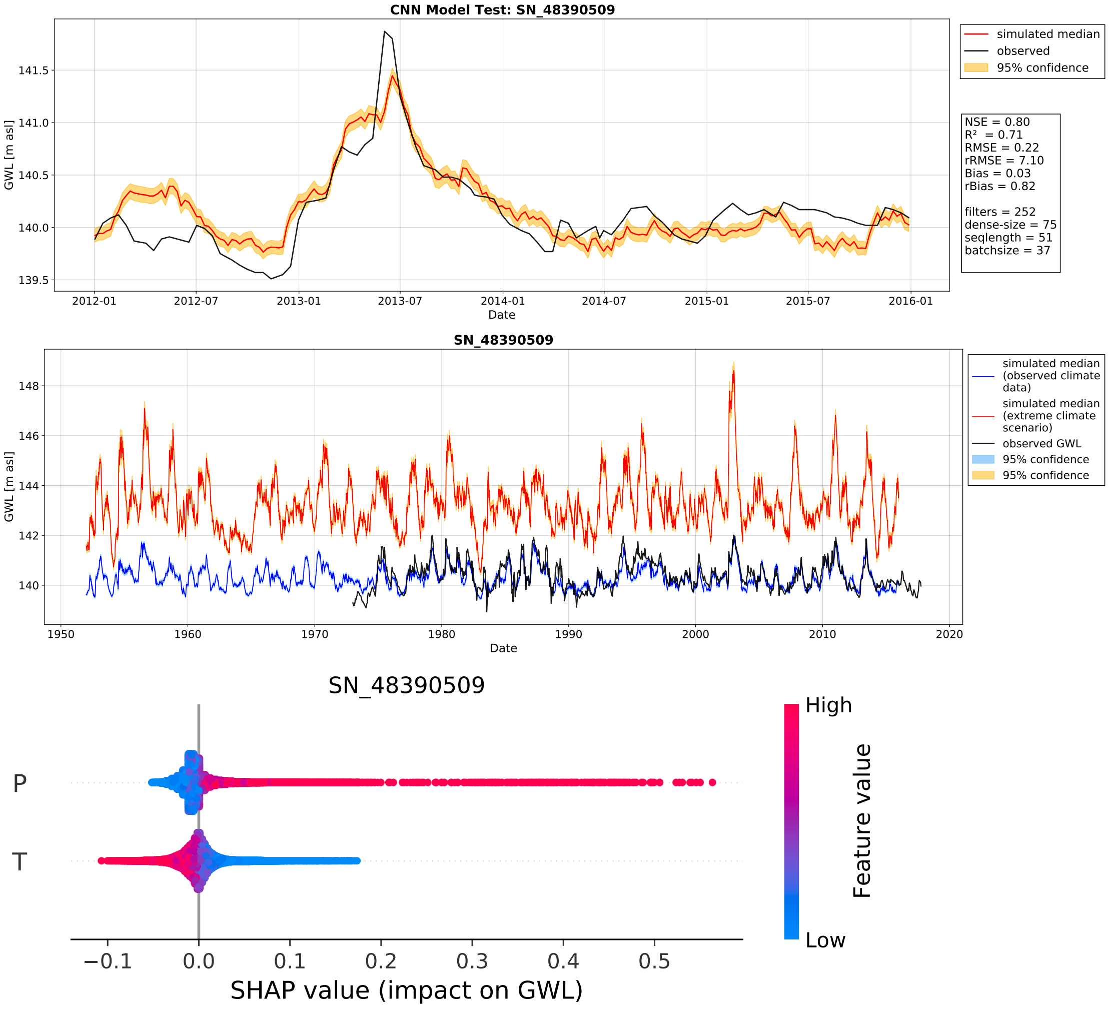

Figure S113: Evaluation of SN_48390509 Model Performance in the past (upper), under extreme climate conditions (middle) and SHAP Summary plot (lower)

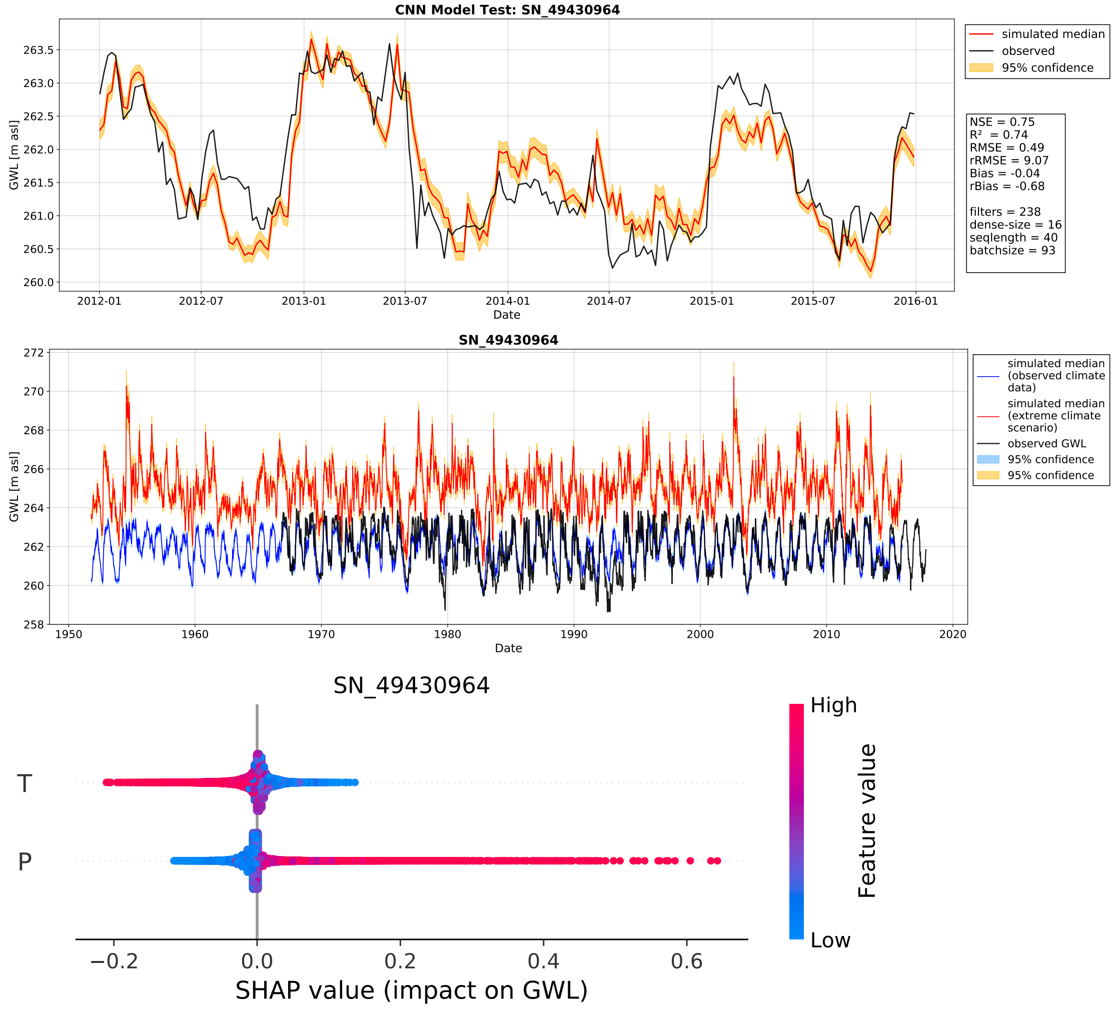

Figure S114: Evaluation of SN_49430964 Model Performance in the past (upper), under extreme climate conditions (middle) and SHAP Summary plot (lower)

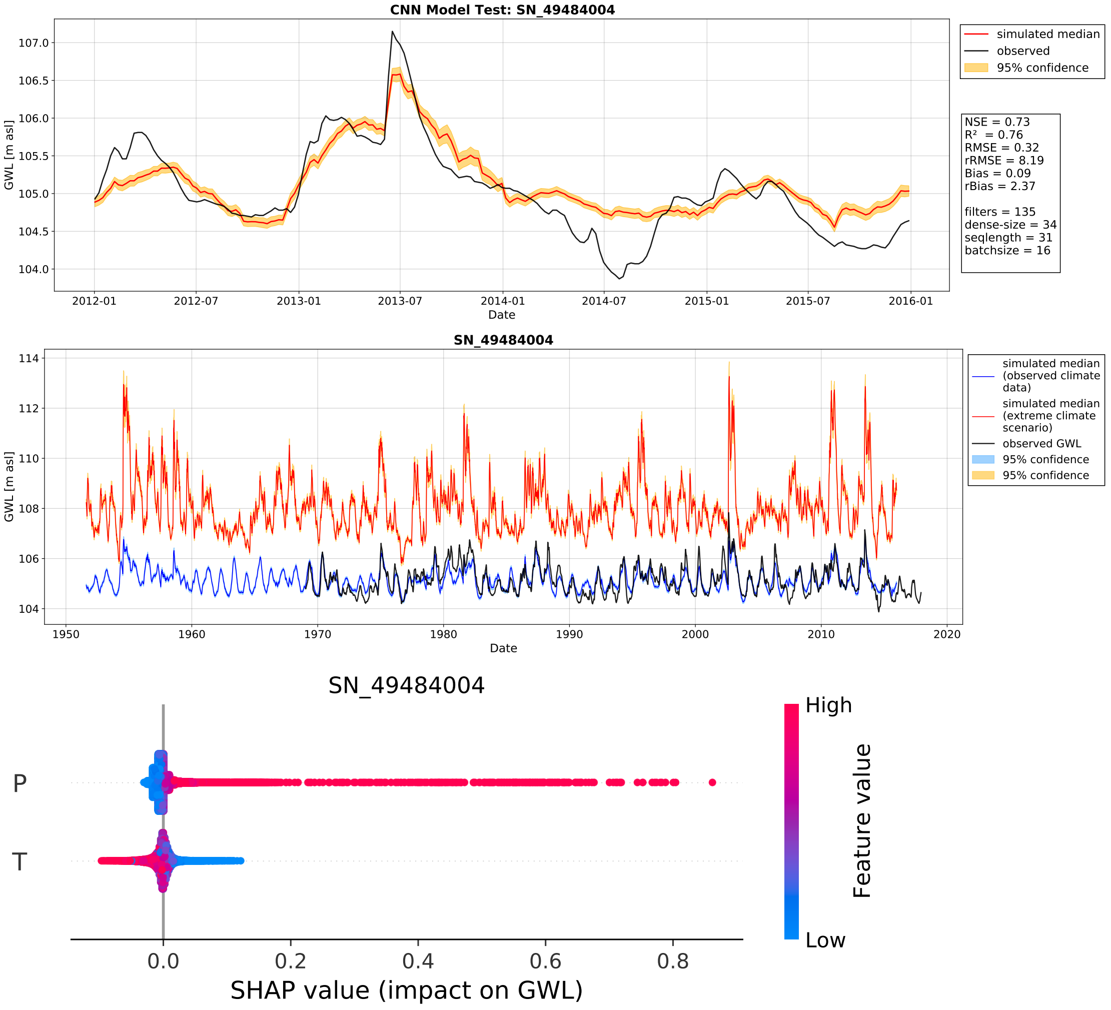

Figure S115: Evaluation of SN_49484004 Model Performance in the past (upper), under extreme climate conditions (middle) and SHAP Summary plot (lower)

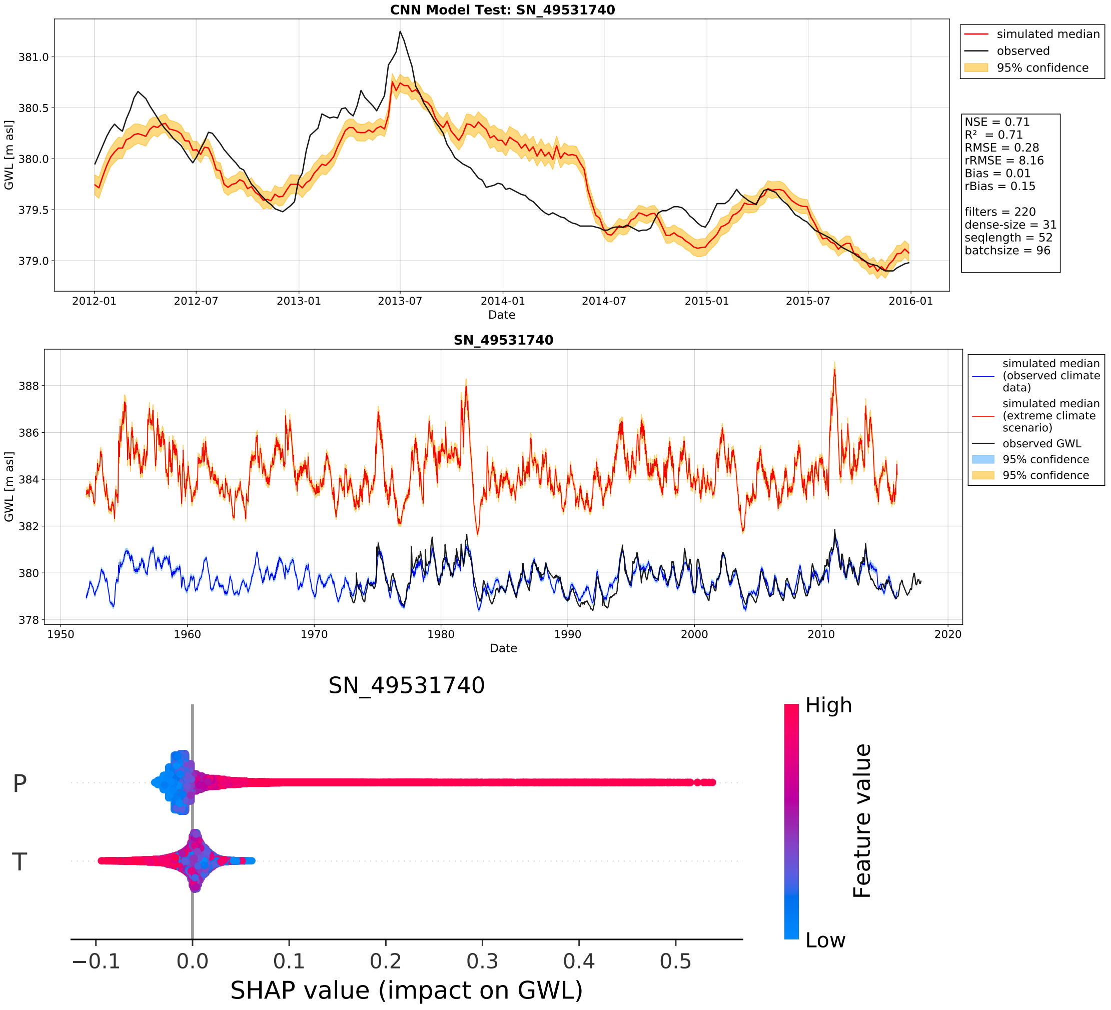

Figure S116: Evaluation of SN_49531740 Model Performance in the past (upper), under extreme climate conditions (middle) and SHAP Summary plot (lower)

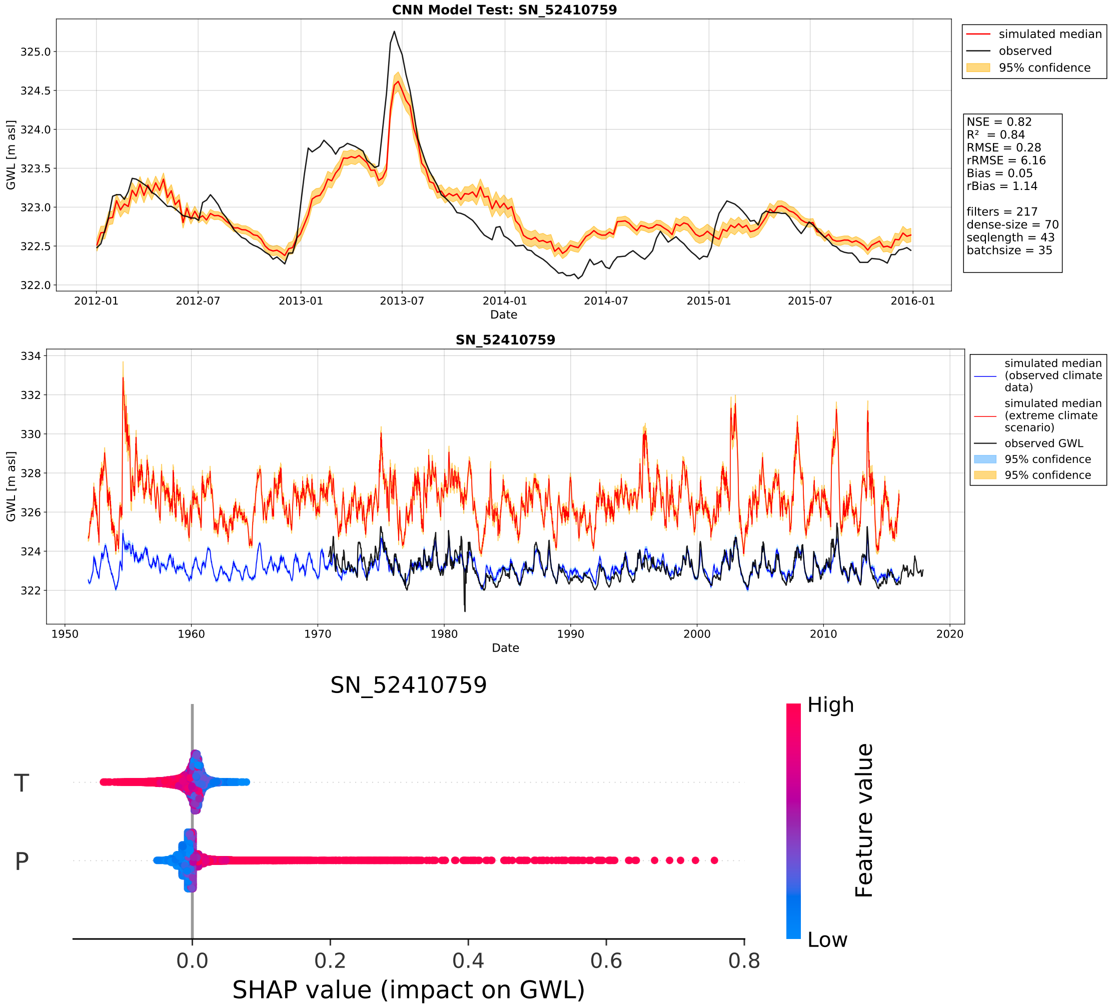

Figure S117: Evaluation of SN_52410759 Model Performance in the past (upper), under extreme climate conditions (middle) and SHAP Summary plot (lower)

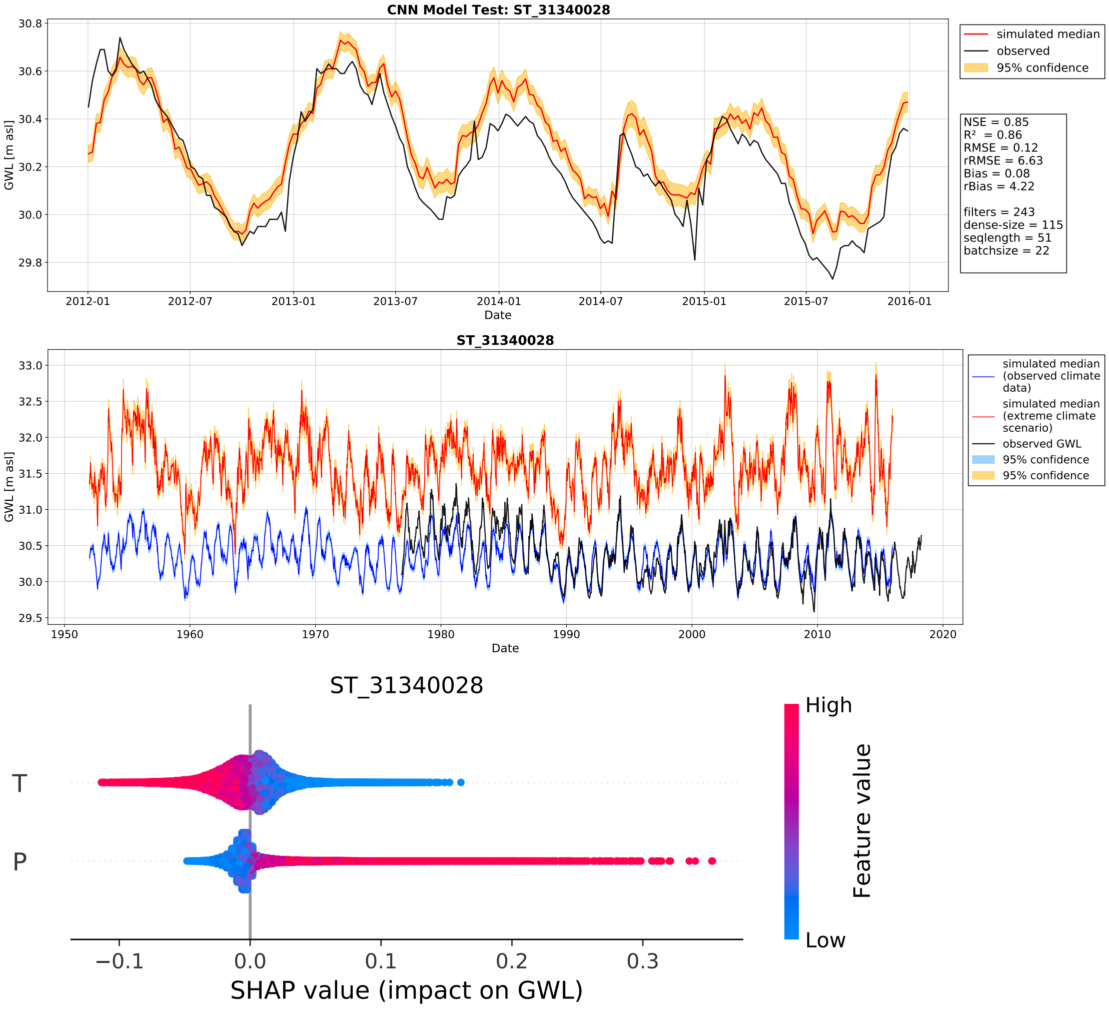

Figure S118: Evaluation of ST_31340028 Model Performance in the past (upper), under extreme climate conditions (middle) and SHAP Summary plot (lower)

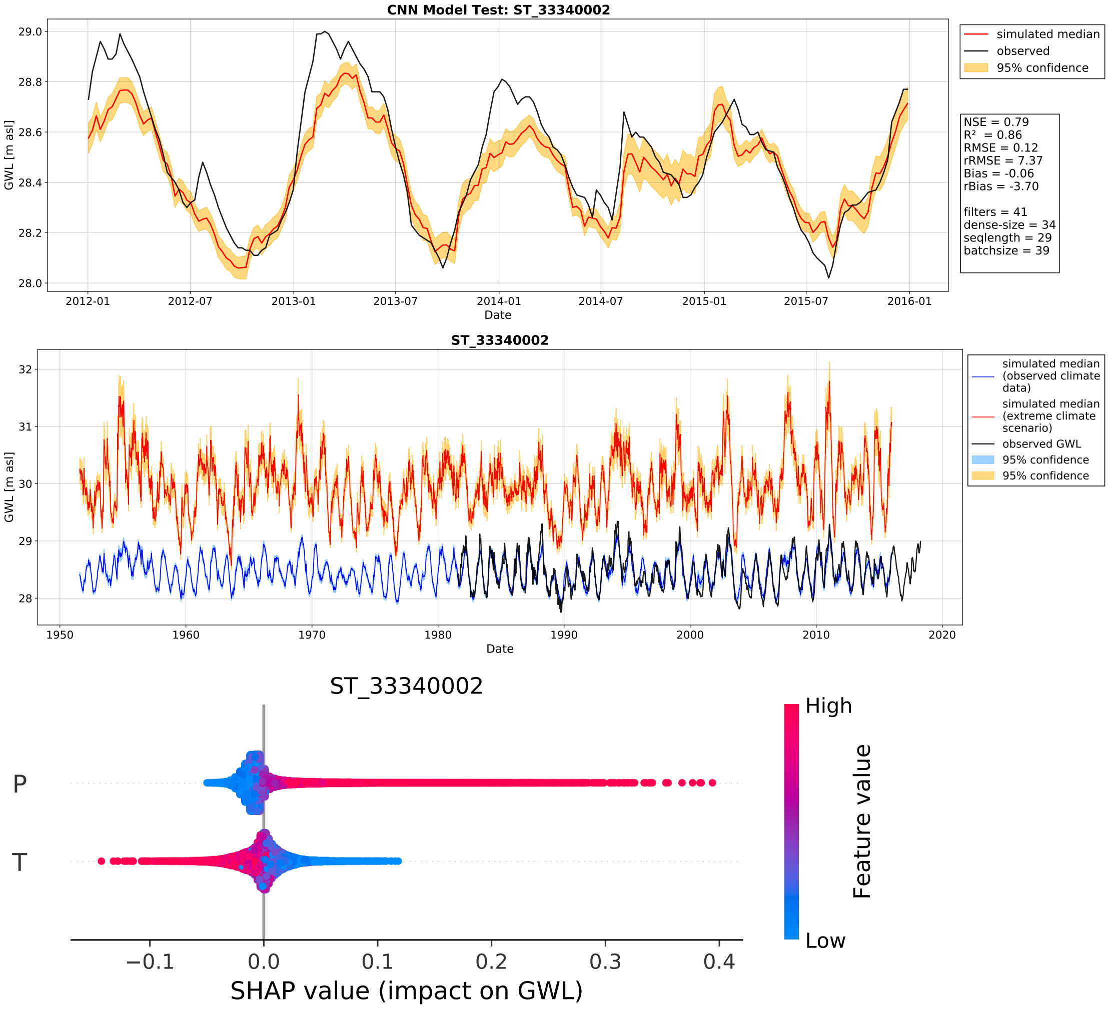

Figure S119: Evaluation of ST_33340002 Model Performance in the past (upper), under extreme climate conditions (middle) and SHAP Summary plot (lower)

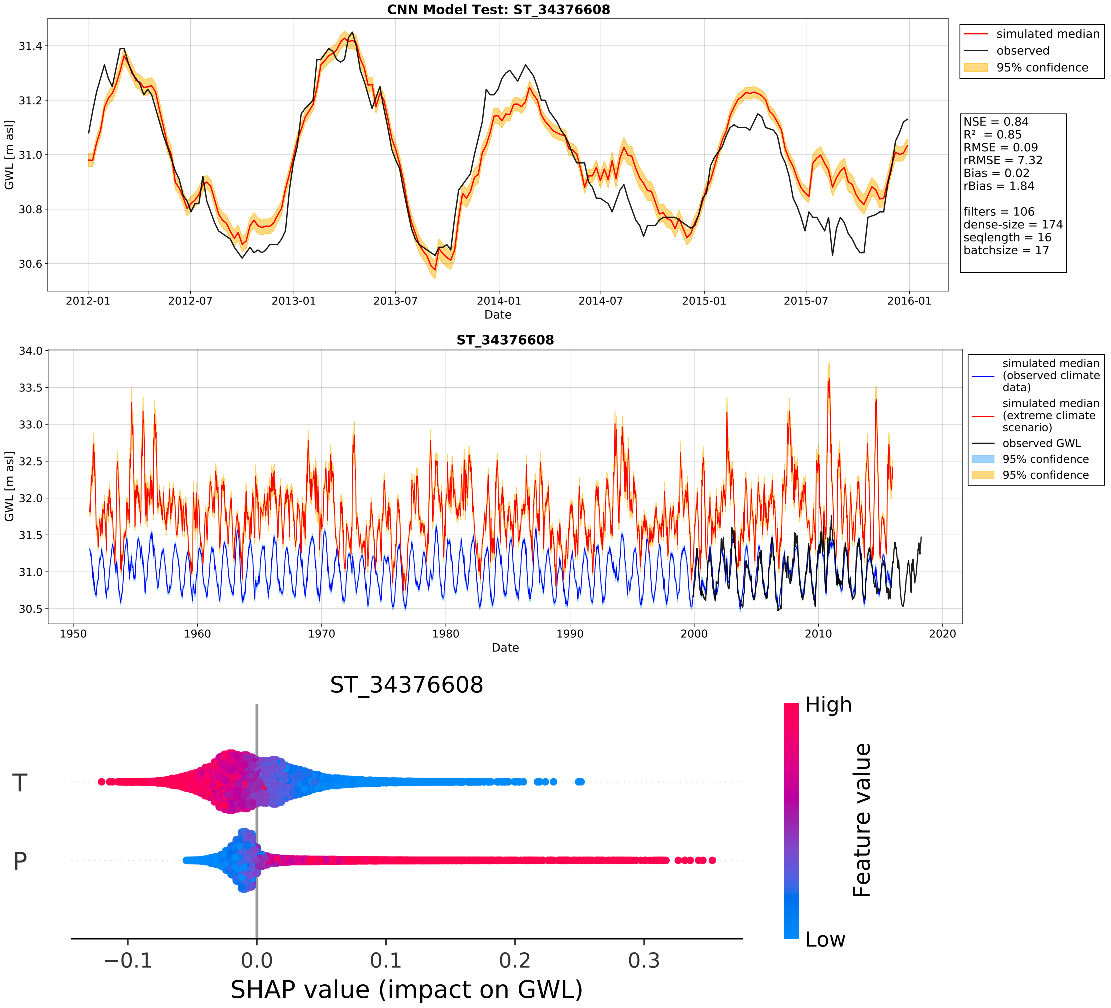

Figure S120: Evaluation of ST_34376608 Model Performance in the past (upper), under extreme climate conditions (middle) and SHAP Summary plot (lower)

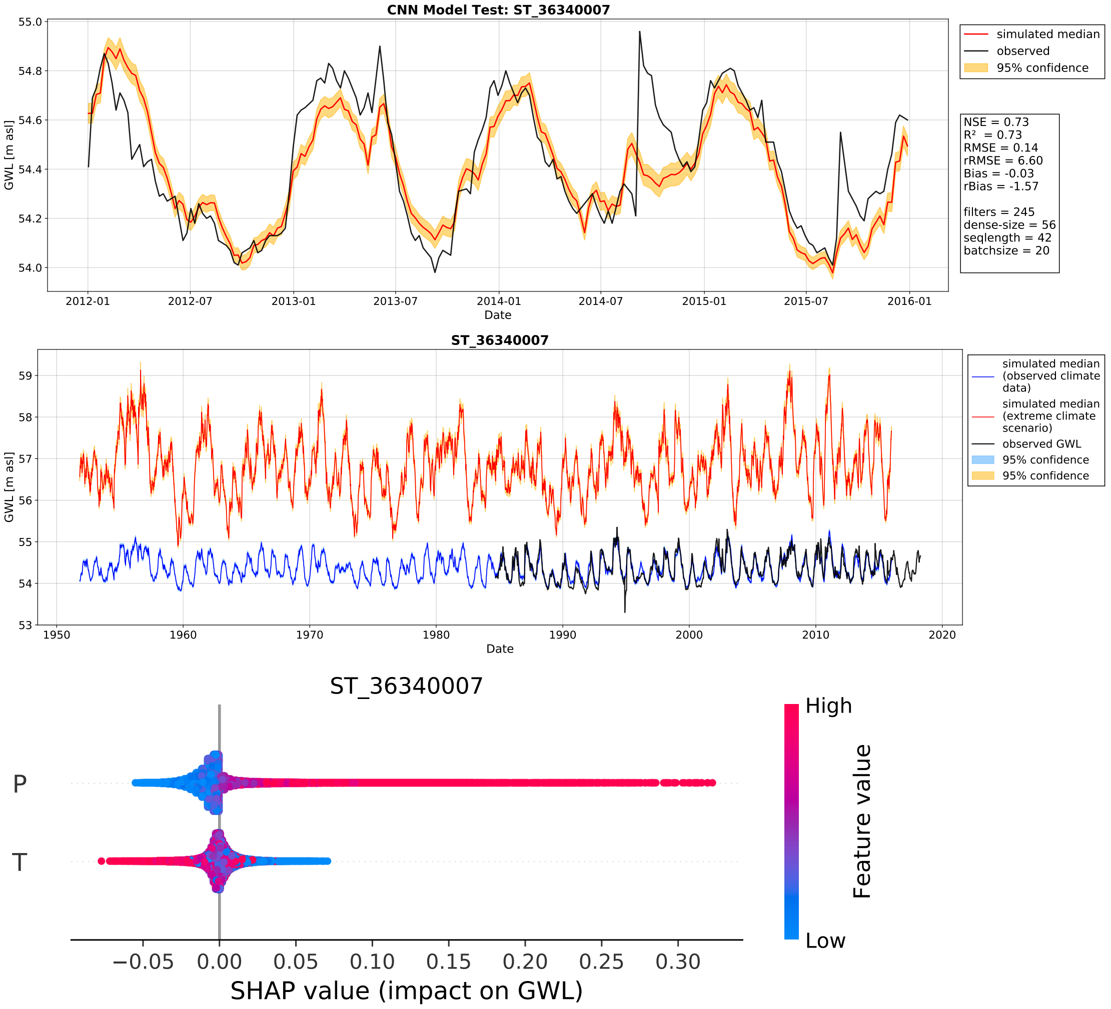

Figure S121: Evaluation of ST_36340007 Model Performance in the past (upper), under extreme climate conditions (middle) and SHAP Summary plot (lower)

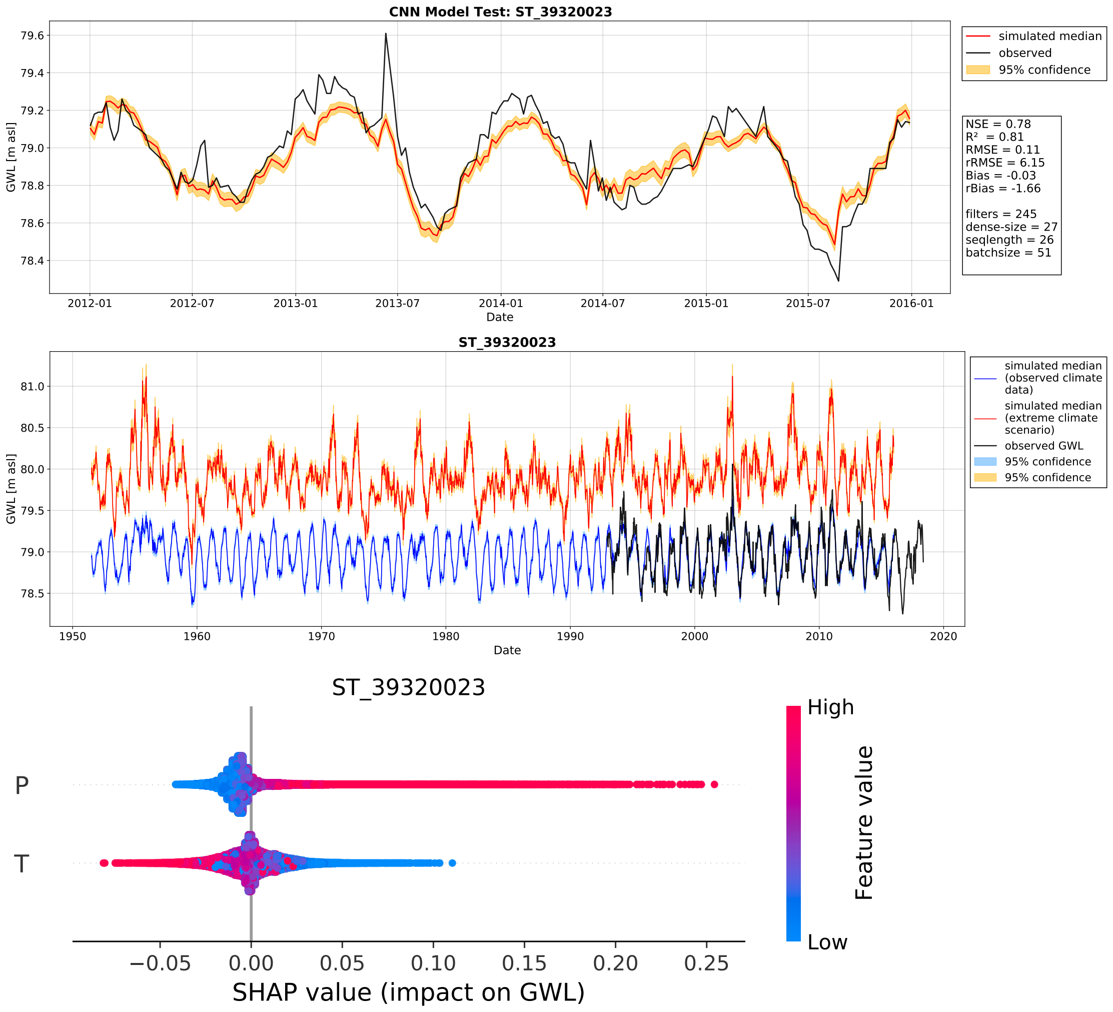

Figure S122: Evaluation of ST_39320023 Model Performance in the past (upper), under extreme climate conditions (middle) and SHAP Summary plot (lower)

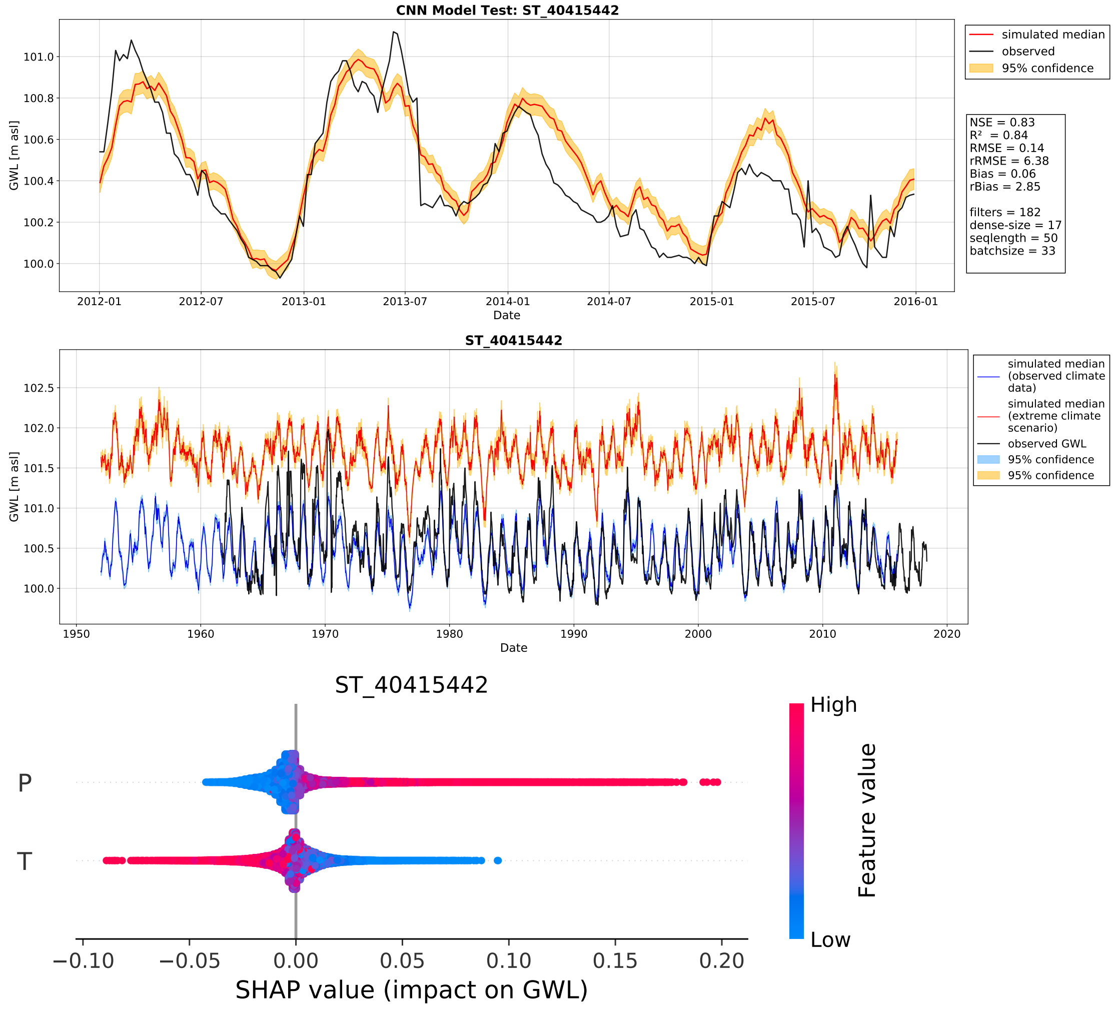

Figure S123: Evaluation of ST_40415442 Model Performance in the past (upper), under extreme climate conditions (middle) and SHAP Summary plot (lower)

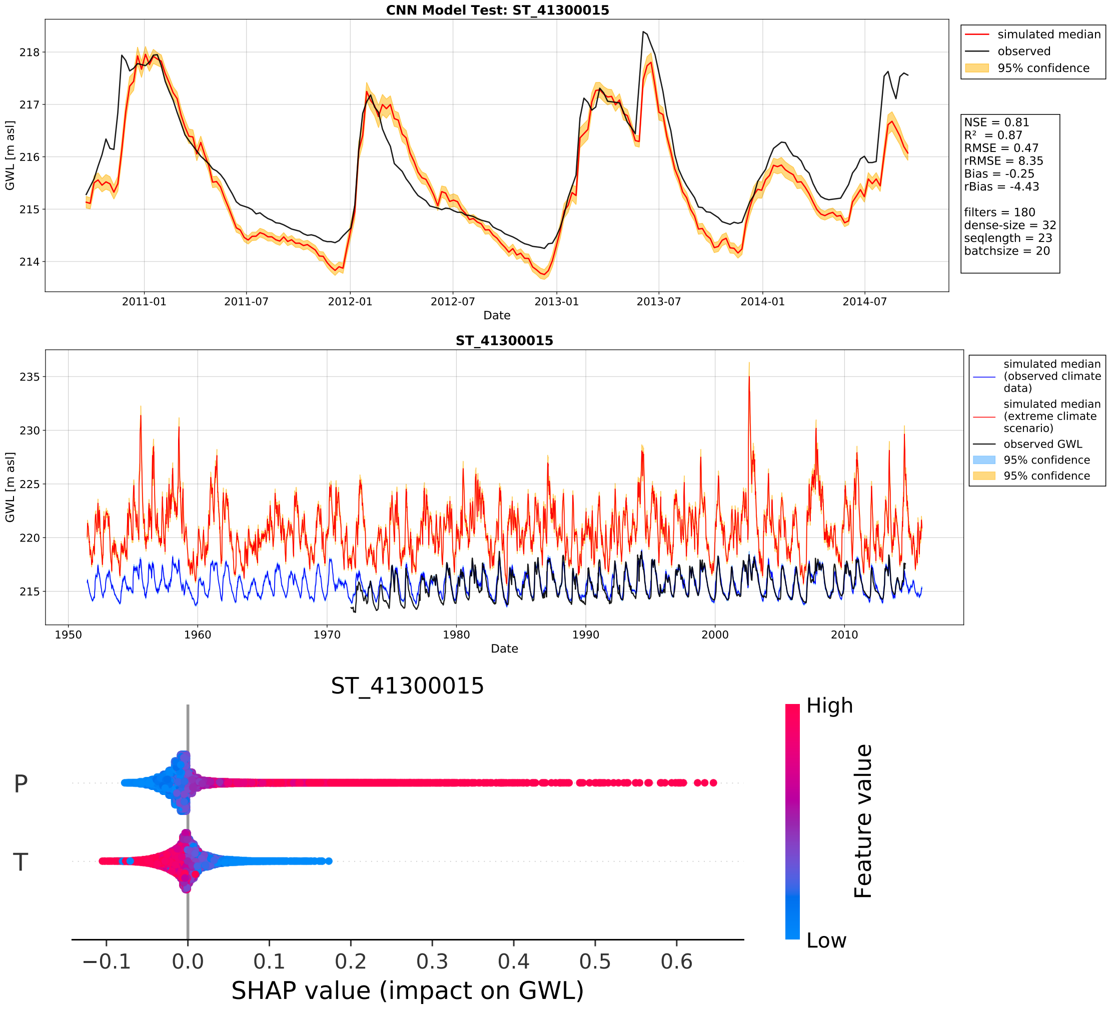

Figure S124: Evaluation of ST_41300015 Model Performance in the past (upper), under extreme climate conditions (middle) and SHAP Summary plot (lower)

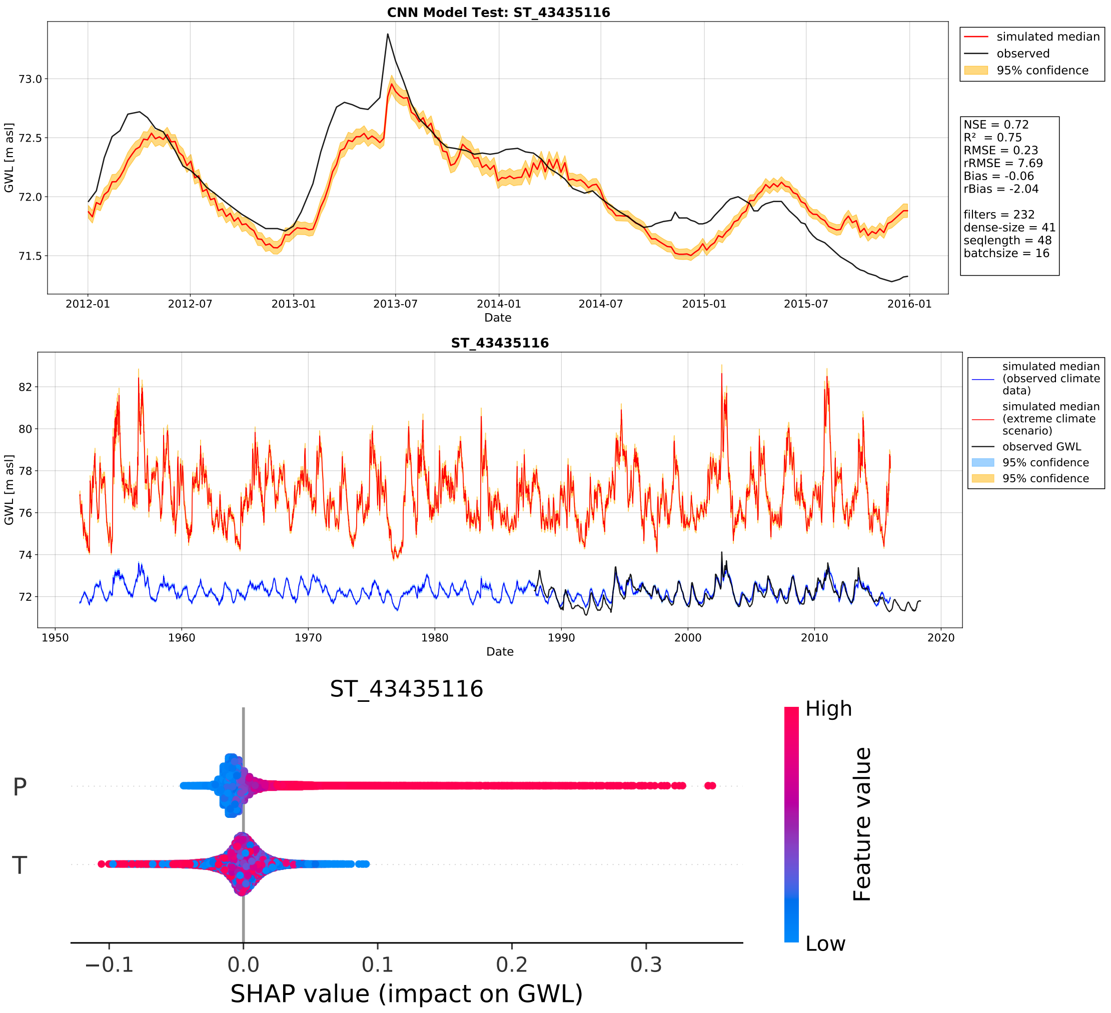

Figure S125: Evaluation of ST_43435116 Model Performance in the past (upper), under extreme climate conditions (middle) and SHAP Summary plot (lower)

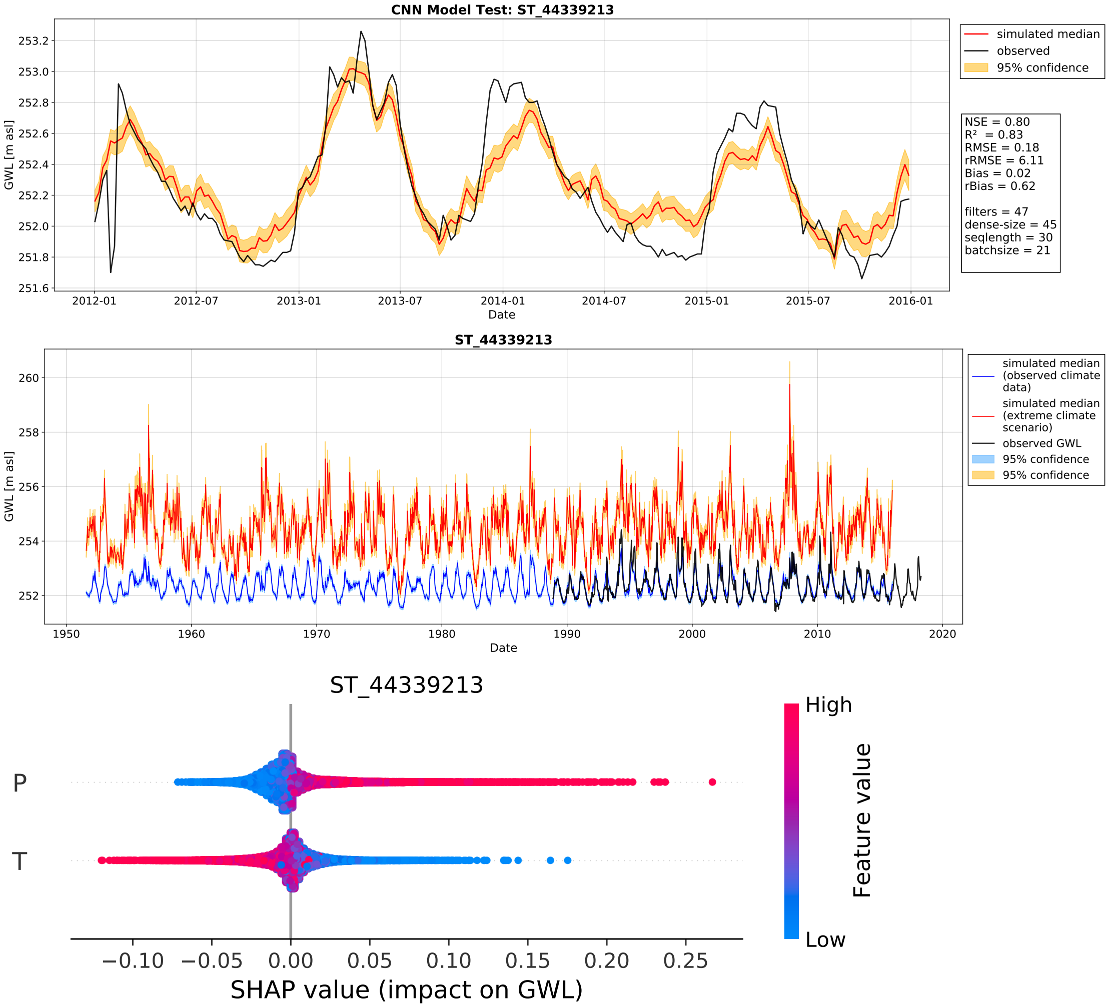

Figure S126: Evaluation of ST_44339213 Model Performance in the past (upper), under extreme climate conditions (middle) and SHAP Summary plot (lower)
